# Access to cyclopropanes with geminal trifluoromethyl and difluoromethylphosphonate groups

**DOI:** 10.3762/bjoc.19.39

**Published:** 2023-04-25

**Authors:** Ita Hajdin, Romana Pajkert, Mira Keßler, Jianlin Han, Haibo Mei, Gerd-Volker Röschenthaler

**Affiliations:** 1 School of Science, Constructor University Bremen gGmbH, Campus Ring 1, Bremen 28759, Germany; 2 Center for Molecular Materials, Bielefeld University, Universitätsstraße 25, 33615 Bielefeld, Germany; 3 Jiangsu Co-Innovation Center of Efficient Processing and Utilization of Forest Resources College of Chemical Engineering, Nanjing Forestry University, Nanjing 210037, China

**Keywords:** alkenes, cyclopropanation, diazo compounds, difluoromethylphosphonate, DFT calculations

## Abstract

A synthetic route to the bench-stable fluorinated masked carbene reagent diethyl 2-diazo-1,1,3,3,3-pentafluoropropylphosphonate, bearing a trifluoromethyl and a difluoromethyl group is reported for the first time. Its application in CuI-catalyzed cyclopropanation reactions with aromatic and aliphatic terminal alkenes under mild reaction conditions is demonstrated. In total, sixteen new cyclopropanes were synthesized in good to very good yields.

## Introduction

**Scheme 1:**
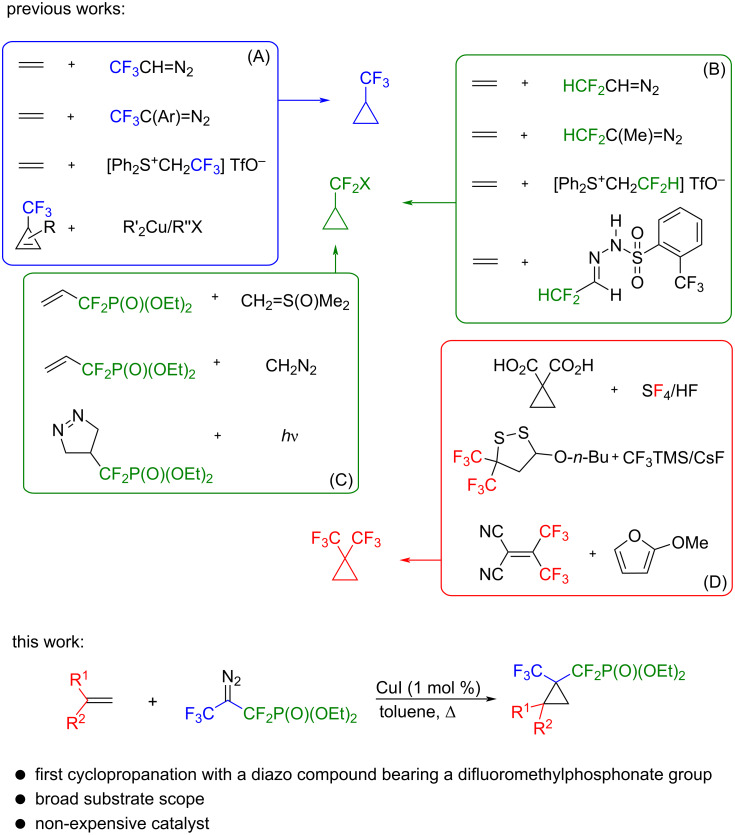
Previous works (A–D) and the extension (this work).

Cyclopropanes constitute a fascinating class of organic compounds due to their unique structure and bond properties [1]. However, their synthetic utility is closely linked to the substitution pattern of the cyclopropane unit [2]. The prevalence of the biologically active cyclopropyl derivatives, either isolated from natural sources or rationally designed as pharmaceutical agents, has inspired chemists to find efficient methods for their preparation. Among them, trifluoromethyl- and difluoromethyl-substituted cyclopropanes are of great interest in pharmaceutical, materials and agricultural chemistry [3]. Due to the unique properties of fluorine, such as highest electronegativity, small atomic radius, or low polarizability, the strategic placement of fluorinated moieties within cyclopropyl rings imparts useful properties to these molecules. Thus, it is not surprising that difluoromethyl- or trifluoromethyl-substituted cyclopropanes serve as important structural motifs in many biologically active molecules as well as in special materials [4–6]. Therefore, as relevant building blocks, tremendous efforts have been made to develop reliable methods for their synthesis. Transition-metal-catalyzed cyclopropanation of alkenes with trifluoromethyldiazoalkanes is a commonly used synthetic strategy for the construction of trifluoromethylcyclopropanes. Recently, also regio- and diastereoselective carbometalation of easily accessible trifluoromethyl-substituted cyclopropenes to access trifluoromethylcyclopropanes has been reported (Scheme 1A) [7–31]. In contrast, the synthesis of difluoromethylcyclopropanes utilizing difluoromethyldiazo reagents remains rather unexplored. Nevertheless, some difluorocarbene reagents, including HCF_2_CH(N_2_) [32], Ph_2_S^+^CH_2_CF_2_H TfO^−^ [33], and difluoroacetaldehyde *N*-triftosylhydrazone (DFHZTfs) [34], have been developed (Scheme 1B). In addition, few examples of difluoromethylphosphonate containing cyclopropanes have been reported to date. These compounds were synthesized either by cyclopropanation of CF_2_P(O)(OEt)_2_-containing alkenoates using a Corey–Chaykovsky reagent (sulfaniumyl or oxosulfaniumyl methanides) and diazomethane or by photolysis of pyrazolines bearing difluoromethylphosphonate moieties (Scheme 1C) [35–38]. Notably, among the various CF_2_-containing functionalities, difluoromethylphosphonates represent a distinct class of compounds whose potential as nonhydrolyzable phosphate mimics is unquestionable. Phosphonates containing CF_2_ groups can sterically and electronically mimic oxygen, enabling the second dissociation constant, p*K*_a2_, to closer mirror those of the phosphates due to the electron-withdrawing effect of fluorine [39]. As a result, improved lipophilicity, metabolic stability or bioavailability of the difluoromethylphosphonate derivatives relative to their nonfluorinated analogues have been observed [40].

However, the extension of this chemistry to highly fluorinated diazo derivatives has not been reported to date. Instead, cyclopropanes with geminal trifluoromethyl substituents were mostly obtained by the deoxofluorination of the corresponding dicarboxylic acids with sulfur tetrafluoride, thiophilic ring-opening reactions with nucleophiles or reactions of donor-substituted furans with bis(trifluoromethyl)-substituted ethylenes (Scheme 1D) [41–43]. The lack of application of highly fluorinated diazo compounds might be rationalized by their low accessibility and high volatility [44].

As a part of our continuing investigations in fluorinated carbene chemistry [45–49], we hypothesized that the synthesis of cyclopropanes with geminal trifluoromethyl and difluoromethylphosphonate groups at the ring might be possible by using our newly developed bench-stable diazo reagent CF_3_C(N_2_)CF_2_P(O)(OEt)_2_. Therefore, we report herein our preliminary results toward this goal via copper iodide-catalyzed cyclopropanation reaction of an acceptor carbene precursor with selected terminal alkenes. We believe that the synergism of both fluorinated substituents could open new synthetic possibilities in the field of fluorinated organic materials.

## Results and Discussion

**Scheme 2:**
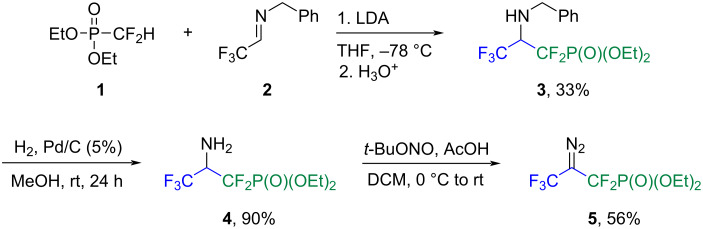
Synthesis of diethyl 2-diazo-1,1,3,3,3-pentafluoropropylphosphonate (**5**).

We began our studies by preparing the fluorinated carbene precursor, diethyl 2-diazo-1,1,3,3,3-pentafluoropropylphosphonate (**5**). This compound was synthesized smoothly within three steps in 16.6% overall yield. The synthetic route commenced from the preparation of *N*-protected amine **3**, followed by the deprotection of benzylamine **3** to furnish **4** and ended with the diazotization of **4** using *tert*-butyl nitrite. As a result, the target diazo reagent **5**, which contains both a trifluoromethyl and a difluoromethylphosphonate moiety, was isolated as a stable, non-volatile liquid (Scheme 2).

To highlight the synthetic utility of the novel carbene precursor **5**, subsequent [2 + 1] cycloaddition reactions with selected terminal aromatic and aliphatic olefins were examined. The reaction conditions were first optimized using 4-methylstyrene as a model substrate. The results are summarized in Table 1.

**Table 1:** Optimization of the reaction conditions^a^.

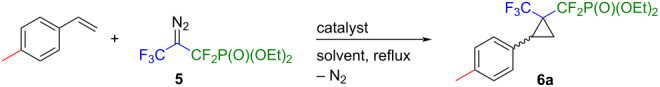					

Entry	Catalyst	*t* (h)	*T* (°C)	Solvent	Conversion rate^b^ (%)

1	Rh_2_(OAc)_4_	5	111	toluene	n.r.
2	Rh_2_(OAc)_4_	3	40	DCM	n.r.
3	CuI	3	40	DCM	n.r.
4	CuI	1	111	toluene	42
5	CuI	2	111	toluene	59
6	CuI	3.5	111	toluene	100^c^
7^d^	–	24	40	DCM	n.r.

^a^Reaction conditions: alkene (0.15 mmol), diazo compound **5** (0.1 mmol), CuI (1 mol %), dry toluene, 111 °C, Ar atmosphere; ^b^Determined by ^19^F NMR spectroscopy; ^c^Isolated yield 74%. ^d^Under UV irradiation.

Initially, dirhodium tetraacetate (Rh_2_(OAc)_4_), the most common catalyst for the preparation of cyclopropanes, was applied. To our surprise, the application of Rh_2_(OAc)_4_ did not lead to the desired product neither in dichloromethane nor in toluene (Table 1, entries 1 and 2). Switching the catalyst to copper(I) iodide in refluxing DCM, did not result in the formation of product **6a**, as well (Table 1, entry 3). However, when CuI was used in boiling toluene, 42% of the diazo reagent **5** was converted to **6a** after only 1 hour of stirring. Thus, to increase the conversion rate, the reaction time was prolonged up to 3.5 h. Indeed, after this time, complete conversion of the diazo reagent **5** was observed and cyclopropane **6a** was isolated in 74% yield (Table 1, entry 6). In addition, in a catalyst-free reaction under UV irradiation, no reaction occurred and the starting diazo compound **5** was recovered (Table 1, entry 7).

**Scheme 3:**
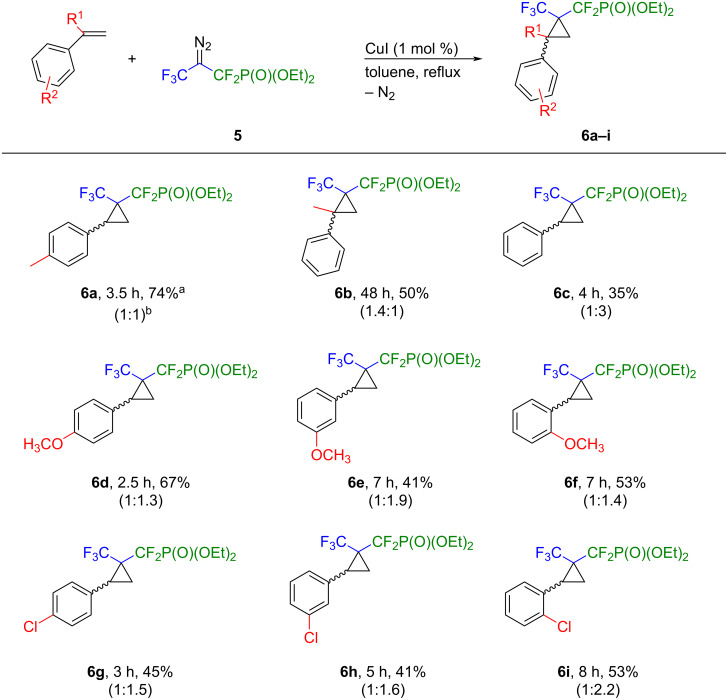
Scope of the cyclopropanation. Reaction conditions: alkene (0.15 mmol), diazo compound **5** (0.1 mmol), CuI (1 mol %), dry toluene, 111 °C, Ar atmosphere. ^a^Yields refer to isolated products; ^b^dr ratio determined by ^19^F NMR spectroscopy.

Having identified the optimal catalyst and conditions, we then examined the scope of the cyclopropanation reaction by reacting selected styrenes with the diazo reagent **5**. As evident from Scheme 3, this method is widely applicable to a range of styrenes that are either not activated or substituted with electron-donating (Me, OMe) or electron-withdrawing (Cl) groups on the aromatic ring. All reactions were carried out successfully and the corresponding cyclopropanes **6a–i** were isolated in moderate to good yields. As expected, the best results were obtained with styrenes bearing electron-donating groups in the *para*-position furnishing cyclopropanes **6a** and **6d** in 74% and 67% yield, respectively. The presence of substituents in ortho and *meta*-positions on the phenyl ring (**6e**, **6f**, **6h**, **6i**), decelerated the process. In the case of α-methylstyrene, complete conversion of diazo reagent **5** to compound **6b** was observed only after 48 hours of heating, which might result from the steric hindrance around the reaction centre.

**Figure 1:**
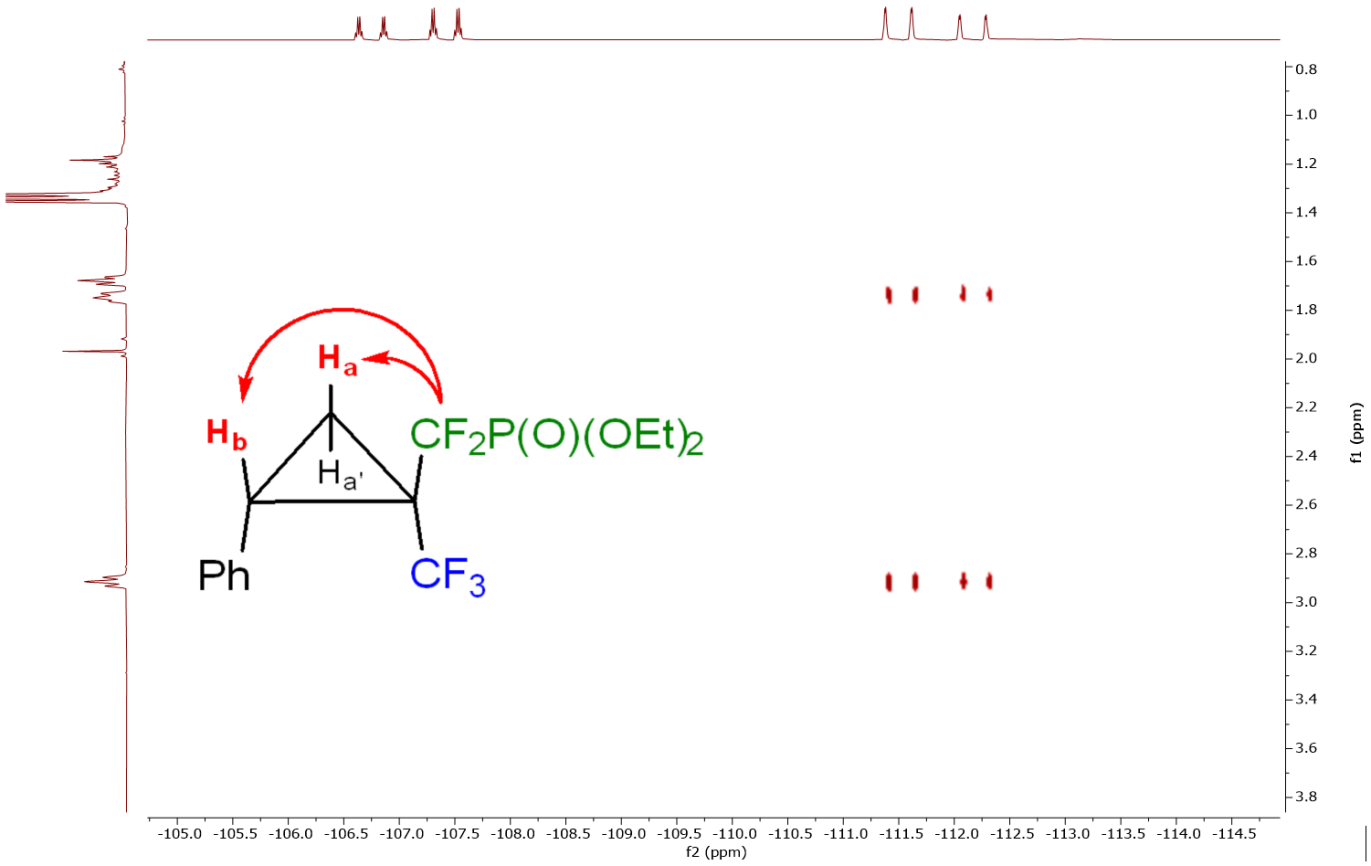
^19^F,^1^H-HOESY spectrum of compound **6c**.

In almost all cases, a mixture of two diastereoisomers was obtained, with a slight preference for one diastereoisomer for compounds **6a**, **6c–i**. Furthermore, we were able to isolate both the main isomer as well as a mixture of two isomers for cyclopropanes **6a**, **6c**, **6d**, **6g**, **6h** and **6i**. To determine the relative stereochemistry of the main isomer, the ^19^F,^1^H-HOESY NMR spectrum of compound **6c** was recorded. The spectrum shows direct correlation of one fluorine nucleus of the difluoromethyl phosphonate group with two cyclopropane protons at 1.75 and 2.91 ppm, respectively. Thus, it is conceivable that the major diastereoisomer adopts *trans* configuration (Figure 1).

**Scheme 4:**
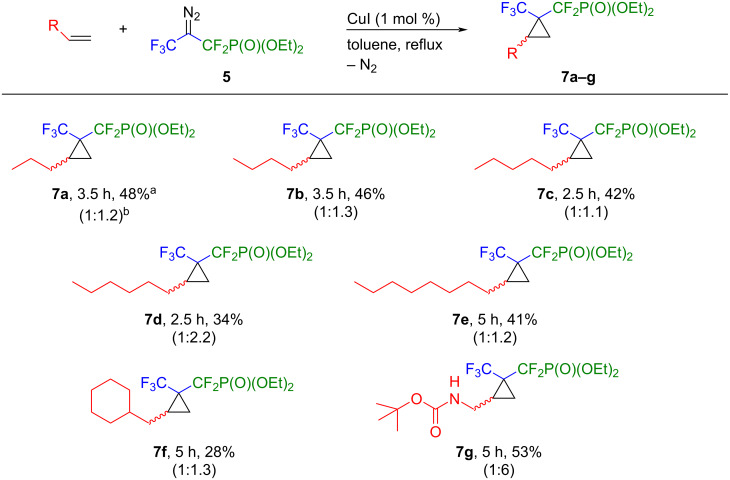
Scope of the cyclopropanation. Reaction conditions: alkene (0.15 mmol), diazo compound **5** (0.1 mmol), CuI (1 mol %), dry toluene, 111 °C, Ar atmosphere. ^a^Yields refer to isolated products; ^b^dr ratio determined by ^19^F NMR spectroscopy.

Furthermore, terminal aliphatic alkenes as well as *N*-Boc-allylamine were subjected to [2 + 1] cycloaddition reactions with the carbene precursor **5**. The results are summarized in Scheme 4. As observed, the procedure was successful with a series of terminal olefins with different aliphatic chains. The total conversion of the diazo compound **5** was achieved after 2.5–3.5 hours of heating and the corresponding cyclopropanes **7a–g** were obtained in moderate to good yield (28–53%). The highest yield (53%) as well as the best diastereoselectivity were recorded for the reaction with *tert*-butyl-*N*-allyl carbamate **7g** (1:6) while the reaction with allylcyclohexane resulted with the lowest yield (**7f**, 28%), probably due to the steric hindrance of the reagents. In the case of *N*-Boc-allylamine, improved diastereoselectivity might result from the coordination of the amino group to the copper centre of an intermediately produced metallocarbene, thus favouring the formation of one diastereoisomer of **7g** [50]. In addition, the cyclopropanes **7a–g** were always obtained as mixtures of two diastereoisomers in different ratios and all attempts to separate them using column chromatography were unsuccessful. In addition, alkenes such as allylpentafluorobenzene, diethyl allylmalonate, allylbenzene and ethyl acrylate did not react with **5**. Instead, the diazo compound **5** decomposed immediately under the reaction conditions described in Scheme 4.

To better understand the mechanism and to confirm the lack of selectivity during the cyclopropanation process with terminal alkenes, the reaction mechanism between the diazo reagent **5** and styrene as a model substrate in the presence of CuI catalyst was investigated by density functional theory (DFT) calculations (Table 2).

**Table 2:** Change in Gibbs free energy Δ*G* (kcal∙mol^−1^) from the CuI-catalyzed cyclopropanation of the diazo compound with styrene for possible stereoisomers **Pr1** to **Pr4**.

Isomer	**Int1**	**TS1**	**Int2**	**TS2** ^a^	**Int3**	**TS3**	**Int4**	**TS4**	**Pr**

**1**	5.2	16.4	−8.7		−26.4	−22.1	−52.3	−41.7	**−44.6**
**2**				8.5	−27.6	−22.5	−50.5	−42.0	−42.5
**3**					–	–	−51.1	−36.0	**−44.1**
**4**					–	–	−51.3	−35.9	−43.3

^a^Exemplarily calculated for one option due to flat potential.

**Scheme 5:**
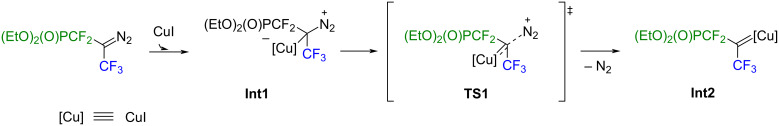
Addition of CuI to the diazo compound **5**.

In the first step, CuI adds to the diazo compound **5** under formation of zwitterionic **Int1** (Δ*G* = 5.2 kcal/mol). Afterwards, copper carbene complex **Int2** is formed after extrusion of nitrogen. The transition state of this metal carbene formation **TS1** was calculated with an activation free energy of 16.4 kcal/mol (Scheme 5).

**Scheme 6:**
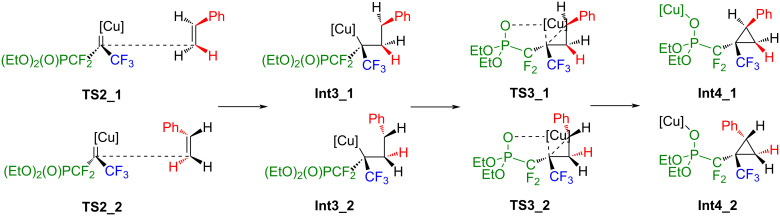
Possible addition of styrene to **Int2** yielding **Int4_1** and **Int4_2** through **Int3_1** and **Int3_2**.

**Scheme 7:**
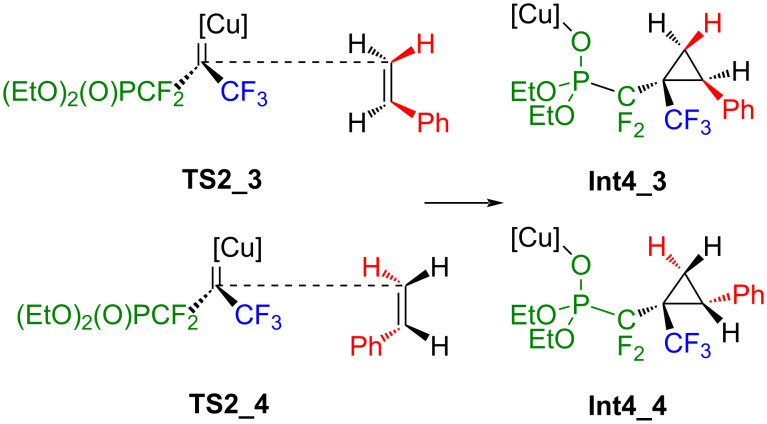
Possible addition of styrene to **Int2** yielding **Int4_3** and **Int4_4** without further intermediates.

**Int2** adds to styrene to the carbon atom in 2-position of the ethenyl group. At this point, four different orientations of the phenyl group are conceivable (Scheme 6 and Scheme 7, **TS2_1** to **TS2_4**).

**Scheme 8:**
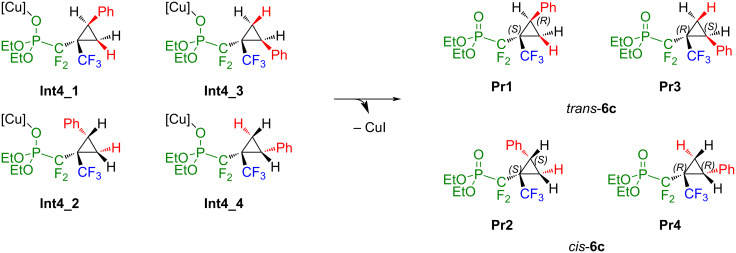
Formation of the products **Pr1** to **Pr4**.

After an early transition state with hardly any activation barrier, the addition of styrene to **Int2** proceeds in the case of **TS2_1** and **TS2_2** to the intermediates **Int3_1** and **Int3_2**. Afterwards, the Cu–C bond is broken, and a Cu–O bond is formed. CuI is transferred to the oxygen atom from the phosphonate group, yielding **Int4_1** and **Int4_2**. In the case of **TS2_3** and **TS2_4**, the addition proceeds smoothly towards **Int4_3** and **Int4_4** without further intermediates (Scheme 7 and Scheme 8). Extrusion of a catalyst yields **Pr1**, **Pr2**, **Pr3** and **Pr4** as presented in Scheme 8.

As shown in Table 2, the formation of the enantiomeric set **Pr1** and **Pr3** (Δ*G* = −44.6 and −44.1 kcal·mol^−1^) is slightly more favorable than the formation of **Pr2** and **Pr4** (Δ*G* = −42.5 and −43.3 kcal·mol^−1^; *cis*-**6c**), respectively. This is consistent with the experimental results, since two sets of signals corresponding to the diastereoisomers *trans*-**6c** and *cis*-**6c** are always observed in the ^19^F NMR spectrum of the crude mixture, with a slight preference for the *trans* isomer (diastereomeric ratio 1.4:1). Moreover, although **Int4** is the overall thermodynamic minimum of the reaction for all stereoisomers, it is still unsurprising that the reaction proceeds towards the product since the abstraction of the catalyst is the last step. Once abstracted, the catalyst is then involved in the next catalytic cycle and thus removed from the equilibrium between **Int4** and **Pr** + CuI. In addition, since **TS2** is an early transition state and the potential is concomitantly very flat, only **TS2_2** was found by means of regular optimization towards a first order saddle point. For the other three possible reaction pathways, this step was examined by relaxed potential energy scans along the indicated C–C bond (see Supporting Information File 1 for full experimental data).

## Conclusion

In summary, a convenient method for the synthesis of a highly fluorinated diazo reagent, diethyl 2-diazo-1,1,3,3,3-pentafluoropropylphosphonate (**5**), was presented for the first time. This compound is a bench-stable, non-volatile, and non-explosive liquid, which facilitated the handling of this reagent. Furthermore, our research highlights the possibility for facile and efficient syntheses of difluoromethylphosphonate-containing cyclopropanes in reactions with selected olefins, carried out under mild conditions with good to very good yields, in the presence of CuI, an inexpensive catalyst. As confirmed by quantum mechanical calculations and experimental results, the cyclopropane formation occurs always with a slight preference for one diastereoisomer. The presented results turn of a new page for the future of diazo chemistry.

## Supporting Information

File 1 Experimental section and characterization of synthesized compounds.

## References

[1] de Meijere A (1979). Angew Chem, Int Ed Engl.

[2] Faust R (2003). Fascinating Natural and Artificial Cyclopropane Architectures. Organic Synthesis Set.

[3] Pons A, Delion L, Poisson T, Charette A B, Jubault P (2021). Acc Chem Res.

[4] Bourlière M, Pietri O, Castellani P, Oules V, Adhoute X (2018). Ther Adv Gastroenterol.

[5] Abutaleb A, Kottilil S, Wilson E (2018). Hepatol Int.

[6] Davies J C, Moskowitz S M, Brown C, Horsley A, Mall M A, McKone E F, Plant B J, Prais D, Ramsey B W, Taylor-Cousar J L (2018). N Engl J Med.

[7] Le Maux P, Juillard S, Simonneaux G (2006). Synthesis.

[8] Mykhailiuk P K, Afonin S, Ulrich A S, Komarov I V (2008). Synthesis.

[9] Mykhailiuk P K, Afonin S, Palamarchuk G V, Shishkin O V, Ulrich A S, Komarov I V (2008). Angew Chem, Int Ed.

[10] Artamonov O S, Mykhailiuk P K, Voievoda N M, Volochnyuk D M, Komarov I V (2010). Synthesis.

[11] Artamonov O S, Slobodyanyuk E Y, Shishkin O V, Komarov I V, Mykhailiuk P K (2013). Synthesis.

[12] Artamonov O S, Slobodyanyuk E Y, Volochnyuk D M, Komarov I V, Tolmachev A A, Mykhailiuk P K (2014). Eur J Org Chem.

[13] Morandi B, Carreira E M (2010). Angew Chem.

[14] Morandi B, Carreira E M (2010). Angew Chem.

[15] Morandi B, Carreira E M (2011). Angew Chem, Int Ed.

[16] Morandi B, Carreira E M (2011). Org Lett.

[17] Künzi S A, Morandi B, Carreira E M (2012). Org Lett.

[18] Hamilton J Y, Morandi B, Carreira E M (2013). Synthesis.

[19] Wolf J R, Hamaker C G, Djukic J-P, Kodadek T, Woo L K (1995). J Am Chem Soc.

[20] Morandi B, Cheang J, Carreira E M (2011). Org Lett.

[21] Mori T, Ujihara K, Matsumoto O, Yanagi K, Matsuo N (2007). J Fluorine Chem.

[22] Morandi B, Mariampillai B, Carreira E M (2011). Angew Chem, Int Ed.

[23] Chen Y, Ruppel J V, Zhang X P (2007). J Am Chem Soc.

[24] Liu C-B, Meng W, Li F, Wang S, Nie J, Ma J-A (2012). Angew Chem, Int Ed.

[25] Li F, Nie J, Sun L, Zheng Y, Ma J-A (2013). Angew Chem, Int Ed.

[26] Li S, Cao W-J, Ma J-A (2017). Synlett.

[27] Bordeaux M, Tyagi V, Fasan R (2015). Angew Chem.

[28] Wang H-X, Wan Q, Low K-H, Zhou C-Y, Huang J-S, Zhang J-L, Che C-M (2020). Chem Sci.

[29] Lin J-H, Xiao J-C (2020). Acc Chem Res.

[30] Duan Y, Lin J-H, Xiao J-C, Gu Y-C (2016). Org Lett.

[31] Myronova V, Cahard D, Marek I (2022). Org Lett.

[32] Hock K J, Mertens L, Koenigs R M (2016). Chem Commun.

[33] Duan Y, Lin J-H, Xiao J-C, Gu Y-C (2017). Chem Commun.

[34] Ning Y, Zhang X, Gai Y, Dong Y, Sivaguru P, Wang Y, Reddy B R P, Zanoni G, Bi X (2020). Angew Chem, Int Ed.

[35] Yokomatsu T, Sato M, Abe H, Suemune K, Matsumoto K, Kihara T, Soeda S, Shimeno H, Shibuya S (1997). Tetrahedron.

[36] Yokomatsu T, Abe H, Sato M, Suemune K, Kihara T, Soeda S, Shimeno H, Shibuya S (1998). Bioorg Med Chem.

[37] Yokomatsu T, Abe H, Yamagishi T, Suemune K, Shibuya S (1999). J Org Chem.

[38] Yokomatsu T, Yamagishi T, Suemune K, Abe H, Kihara T, Soeda S, Shimeno H, Shibuya S (2000). Tetrahedron.

[39] Shevchuk M, Wang Q, Pajkert R, Xu J, Mei H, Röschenthaler G-V, Han J (2021). Adv Synth Catal.

[40] Romanenko V D, Kukhar V P (2006). Chem Rev.

[41] Dmowski W, Wolniewicz A (2000). J Fluorine Chem.

[42] Petrov V (2018). J Fluorine Chem.

[43] Mloston G, Huisgen R (1994). J Heterocycl Chem.

[44] Linev V V, Kolomiets A F, Fokin A V (1992). Russ Chem Bull.

[45] Mei H, Liu J, Pajkert R, Wang L, Röschenthaler G-V, Han J (2021). Org Chem Front.

[46] Mei H, Wang L, Pajkert R, Wang Q, Xu J, Liu J, Röschenthaler G-V, Han J (2021). Org Lett.

[47] Liu J, Xu J, Pajkert R, Mei H, Röschenthaler G-V, Han J (2021). Acta Chim Sin (Chin Ed).

[48] Wang Q, Wang L, Pajkert R, Hajdin I, Mei H, Röschenthaler G-V, Han J (2021). J Fluorine Chem.

[49] Liu J, Pajkert R, Wang L, Mei H, Röschenthaler G-V, Han J (2022). Chin Chem Lett.

[50] Ha T M, Guo W, Wang Q, Zhu J (2020). Adv Synth Catal.

