# Supplementary material for: Access to cyclopropanes with geminal trifluoromethyl and difluoromethylphosphonate groups
Source: Beilstein J Org Chem. 2023 Apr 25;19:541–9. doi: 10.3762/bjoc.19.39 (PMC10155617; doi:10.3762/bjoc.19.39)
Supplement: File 1 — Experimental section and characterization of synthesized compounds. [file Beilstein_J_Org_Chem-19-541-s001.pdf]

BEILSTEIN JOURNAL OF ORGANIC CHEMISTRY

# Supporting Information

for

# Access to cyclopropanes with geminal trifluoromethyl and difluoromethylphosphonate groups

Ita Hajdin, Romana Pajkert, Mira Keßler, Jianlin Han, Haibo Mei and Gerd-Volker Röschenthaler

*Beilstein J. Org. Chem.* **2023,** *19,* 541–549. doi:10.3762/bjoc.19.39

# Experimental section and characterization of synthesized compounds

License and Terms: This is a supporting information file under the terms of the Creative Commons Attribution License (https://creativecommons.org/licenses/by/4.0). Please note that the reuse, redistribution and reproduction in particular requires that the author(s) and source are credited and that individual graphics may be subject to special legal provisions.
The license is subject to the *Beilstein Journal of Organic Chemistry* terms and conditions: (https://www.beilstein-journals.org/bjoc/terms)

**Table of contents**

1. General information ...S2

2. Synthesis of diethyl 2-(benzylamino)-1,1,3,3,3-pentafluoropropylphosphonate (**3**) ...S2

3. Synthesis of diethyl 2-amino-1,1,3,3,3-pentafluoropropylphosphonate (**4**) ...S3

4. Synthesis of diethyl 2-diazo-1,1,3,3,3-pentafluoropropylphosphonate (**5**) ...S4

5. General procedure for the cyclopropanation of alkenes with diazo compound (**5**). ...S5

6. Characterization data ...S5

7. Copies of $^{1}H$ NMR, $^{13}C$ NMR, $^{19}F$ NMR, and $^{31}P$ NMR spectra for the compounds **3**, **4**, **5**, **6a–i** and **7a–g** ...S21

8. Copies of HOESY $^{19}F,^{1}H$ 2D NMR spectra ...S69

9. DFT calculations ...S71

10. References ...S88

## 1. General information

All reactions were carried out under anhydrous conditions and under argon atmosphere. THF and toluene were freshly distilled from sodium benzophenone ketyl and DCM was distilled over calcium hydride before use. All reagents were purchased from commercial sources and used without further purification. Diethyl difluoromethylphosphonate (**1**) and *N*-benzyltrifluoroacetaldimine (**2**) were prepared according to the known procedures [1,2]. The products were purified by column chromatography over silica gel 60 (230−400 mesh ASTM). Analytical TLCs were performed with silica gel 60 F254 plates. Visualization was accomplished using UV light or by spraying with $Ce(SO_4)_2$ solution in 5% $H_2SO_4$. NMR spectra were recorded on a JEOL ECX400 instrument and the frequencies for $^{1}H$, $^{13}C$, $^{19}F$ and $^{31}P$ NMR are 400, 100, 376 and 161 MHz, respectively. $^{31}P$ NMR spectra were broadband decoupled from hydrogen nuclei. All measurements were carried out in solution in $CDCl_3$. Chemical shifts (δ) are reported in ppm and coupling constants (*J*) in Hz. High-resolution mass spectra (HRMS) were recorded on an ESI-Qq-TOF mass spectrometer.

## 2. Synthesis of diethyl 2-(benzylamino)-1,1,3,3,3-pentafluoropropylphosphonate (3)

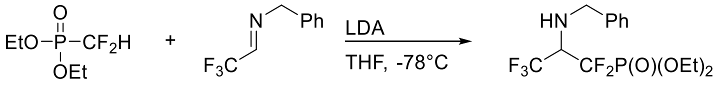


**1** **2** **3**, 33%

To a solution of diisopropylamine (24.4 mL, 17.3 mmol) and *n*-butyllithium (71.3 mL, 17.3 mmol, 2.5 M solution in hexanes) in dry THF (150 mL) at −78 °C and under argon atmosphere diethyl difluoromethylphosphonate (**1**, 22.6 mL, 14.4 mmol) was added dropwise for 15 min and the mixture was stirred for 30 min at −78 °C. Then, a solution of *N*-benzyltrifluoroacetaldimine (**2**, 27.0 g, 14.4 mmol) in dry THF (200 mL) was added dropwise and the resultant mixture was stirred at −78 °C for the next 3 h. Afterwards, the solution was

warmed to RT with the addition of saturated aqueous $NH_4Cl$ (200 mL) and it was stirred overnight. After dilution with $H_2O$ (50 mL) the solution was extracted with EtOAc (3 × 200 mL), combined organic layers were washed with brine (200 mL), dried over $MgSO_4$ and concentrated under reduced pressure. The crude product was purified via flash column chromatography on silica gel, using DCM: EtOAc (9:1 ratio) to give compound **3** in 33% yield as a yellow oil. **$^1$H NMR** ($CDCl_3$, 400 MHz): δ 7.37 (d, 2H, $^3J_{HH}$ = 8.0 Hz), 7.31-7.22 (m, 3H), 4.33-4.13 (m, 4H), 4.12-3.93 (m, 2H), 3.91-3.77 (m, 1H), 1.91 (s, 1H), 1.34 (t, 3H, $^3J_{HH}$ = 8.0 Hz), 1.25 (t, 3H, $^3J_{HH}$ = 8.0 Hz); **$^{13}$C NMR** ($CDCl_3$, 100 MHz) δ 138.6, 128.5 (d, $^1J_{CH}$ = 13.0 Hz),127.6, 123.4 (td, $^1J_{CF}$ = 285.0 Hz, $^1J_{CP}$ = 12.0 Hz), 118.7 (td, ,$^1J_{CF}$ = 269.0 Hz, $^1J_{CP}$ = 213.0 Hz), 64.9 (dt, $^2J_{CP}$ = 7.0 Hz, $^2J_{CP}$ = 2.0 Hz), 61.8-60.3 (m), 53.0, 16.3 (dd, $^3J_{CP}$ = 13.0 Hz, $^3J_{CP}$ = 6.0 Hz); **$^{19}$F NMR** ($CDCl_3$, 376 MHz): δ -68.8 (dq, 3F, $^4J_{FF}$ = 22.6 Hz, $^4J_{FP}$ = 3.8 Hz), -109.8 (ddqd, 1F, $^2J_{FF}$ = 304.6 Hz, $^2J_{FP}$ = 97.8 Hz, $^3J_{FH}$ = 15.0 Hz, $^4J_{FF}$ = 3.8 Hz), -123.1 (ddqd, 1F, $^2J_{FF}$ = 304.6 Hz, $^2J_{FP}$ = 97.8 Hz, $^3J_{FH}$ = 15.0 Hz, $^4J_{FF}$ = 3.8 Hz); **$^{31}$P {$^1$H} NMR** ($CDCl_3$, 161 MHz): δ 5.6 (tq, 1P, $^2J_{PF}$ = 99.8 Hz, $^3J_{PH}$ = 3.2 Hz). **HRMS** (ESI) *m/z*: $[M + H]^+$ calcd. for $C_{14}H_{20}F_5NO_3P$ 376.1101; found 376.1095.

### 3. Synthesis of diethyl 2-amino-1,1,3,3,3-pentafluoropropylphosphonate (4)

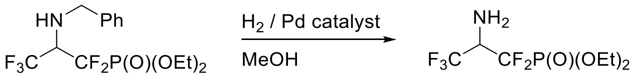


3 4, 90%

To a solution of **3** (11.6 g, 3.1 mmol) in MeOH (200 mL) Pd catalyst, Palladium 5 wt. % (dry basis) on activated carbon (2.0 g, 3.1 mmol) was added. Then, the reaction mixture was degassed for 10 min. Afterwards, $H_2$ was introduced into the reaction from a balloon and the mixture was left to stir overnight. The resulting suspension was filtered over Celite, washed with MeOH and evaporated under reduced pressure to furnish compound **4** in 90% yield as orange oil. **$^1$H NMR** ($CDCl_3$, 400 MHz): δ 4.28-4.16 (m, 4H), 3.86-3.73 (m, 1H), 1.85 (s, 2H),

1.29 (dt, 6H, ${}^{3}J_{HH}$ = 12.0, 4.0 Hz); **$^{13}$C NMR** ($CDCl_3$, 100 MHz): δ = 124.7 (qd, ${}^{1}J_{CF}$ = 282.0 Hz, ${}^{1}J_{CP}$ = 12.0 Hz), 117.8 (td, ${}^{1}J_{CF}$ = 269.0 Hz, ${}^{1}J_{CP}$ = 213.0 Hz), 65.2-65.0 (m), 56.5-55.0 (m), 16.2 (dd, ${}^{3}J_{CP}$ = 6.0, ${}^{3}J_{CP}$ = 2.0 Hz); **$^{19}$F NMR** ($CDCl_3$, 376 MHz): δ -72.3 (dq, 3F, ${}^{4}J_{FF}$ = 22.6 Hz, ${}^{4}J_{FP}$ = 7.5 Hz), -113.1 (ddqd, 1F, ${}^{2}J_{FF}$ = 308.3 Hz, ${}^{2}J_{FP}$ = 97.8 Hz, ${}^{3}J_{FH}$ = 15.0 Hz, ${}^{4}J_{FF}$ = 3.8 Hz), -125.8 (ddqd, 1F, ${}^{2}J_{FF}$ = 308.3 Hz, ${}^{2}J_{FP}$ = 97.8 Hz, ${}^{3}J_{FH}$ = 15.0 Hz, ${}^{4}J_{FF}$ = 3.8 Hz); **$^{31}$P {$^{1}$H} NMR** ($CDCl_3$, 161 MHz): δ 5.5 (t, 1P, ${}^{2}J_{PF}$ = 96.6 Hz). **HRMS** (ESI) *m/z*: $[M + Na]^+$ calcd. for $C_7H_{13}F_5NNaO_3P$ 308.0451; found 308.0446.

## 4. Synthesis of diethyl 2-diazo-1,1,3,3,3-pentafluoropropylphosphonate (5)

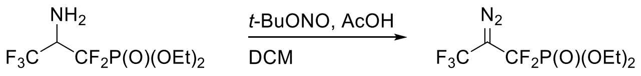


**4** **5**, 56%

A solution of **4** (7.5 g, 2.6 mmol) in dry DCM (300 mL) under argon atmosphere was cooled down to 0 °C and *t*-BuONO (4.7 mL, 3.9 mmol) and AcOH (0.6 mL, 1.1 mmol) were added in one portion. The reaction mixture was stirred overnight at RT. The solvent was evaporated, and the crude product was purified via flash column chromatography on silica gel using DCM as eluent to give compound **5** in 56% yield as yellow oil. **$^{1}$H NMR** ($CDCl_3$, 400 MHz): δ 4.33-4.20 (m, 4H), 1.32 (t, 6H, ${}^{3}J_{HH}$ = 8.0 Hz); **$^{13}$C NMR** ($CDCl_3$, 100 MHz): δ = 123.7 (qd, ${}^{1}J_{CF}$ = 286.0 Hz, ${}^{1}J_{CP}$ = 5.0 Hz), 115.3 (dt, ${}^{1}J_{CF}$ = 264.0 Hz, ${}^{1}J_{CP}$ = 230.0 Hz), 65.6 (d, ${}^{2}J_{CP}$ = 7.0 Hz), 16.2 (d, ${}^{3}J_{CP}$ = 6.0 Hz); **$^{19}$F NMR** ($CDCl_3$, 376 MHz): δ -55.1 (td, 3F, ${}^{4}J_{FF}$ = 7.5 Hz, ${}^{4}J_{FP}$ = 3.8 Hz), -104 (dq, 2F, ${}^{2}J_{FP}$ = 112.8 Hz, ${}^{4}J_{FF}$ = 7.5 Hz); **$^{31}$P {$^{1}$H} NMR** ($CDCl_3$, 161 MHz): δ 3.8 (tq, 1P, ${}^{2}J_{PF}$ = 111.1 Hz, ${}^{3}J_{PH}$ = 1.61 Hz).

## 5. General procedure for the cyclopropanation of alkenes with diazo compound (5).

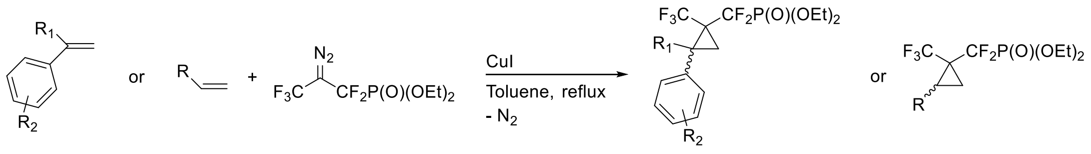


In a manner similar to Ref. 3. To a solution of alkene (2.5 mmol) and CuI (0.17 mmol) in dry toluene under argon atmosphere, **5** (1.7 mmol) was added. The reaction mixture was stirred under reflux. After the time indicated, the solvent was evaporated under reduced pressure and the crude product was purified via flash chromatography on silica gel using DCM/EtOAc (10:1 ratio) as eluent.

## 6. Characterization data

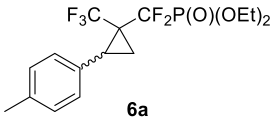


**6a**

Diethyl (2-(*p*-tolyl)-1-(trifluoromethyl)cyclopropyl)difluoromethylphosphonate (**6a**) was prepared from 0.3 g (0.1 mmol) of **5**, 0.019 g (0.01 mmol) of CuI, 0.2 mL (0.15 mmol) of 4-methylstyrene and 5 mL of dry toluene. The reaction mixture was refluxed for 3.5 h to give compound **6a** in 74% yield as yellow oil. Mixture of isomers in 1:1 ratio. *Major isomer*: **$^{1}$H NMR** ($CDCl_3$, 400 MHz): δ 7.20-7.17 (m, 2H), 7.05-7.02 (m, 2H), 4.34-4.19 (m, 4H), 2.93 (t, 1H, $^{3}J_{HH}$ = 8.0 Hz), 1.72-1.69 (m, 2H), 1.34 (t, 6H, $^{3}J_{HH}$ = 8.0 Hz), 1.18 (t, 3H, $^{4}J_{HH}$ = 8.0 Hz); **$^{13}$C NMR** ($CDCl_3$, 100 MHz): δ = 136.8, 130.3 (d, $^{3}J_{CH}$ = 11.0 Hz), 129.4, 123.3 (qd, $^{1}J_{CF}$ = 274.0 Hz, $^{1}J_{CP}$ = 4.0 Hz), 118.0 (td, $^{1}J_{CF}$ = 306.0 Hz, $^{1}J_{CP}$ = 217.0 Hz), 65.2-64.7 (m), 33.5-32.5 (m), 30.9, 29.7, 26.7, 16.3 (dt, $^{3}J_{CP}$ = 15.0 Hz, $^{3}J_{CP}$ = 6.0 Hz); **$^{19}$F NMR** ($CDCl_3$, 376 MHz): δ -59.0 (t, 3F, $^{4}J_{FF}$ = 7.5 Hz), -106.8 (ddq, 1F, $^{2}J_{FF}$ = 312.1 Hz, $^{2}J_{FP}$ = 109.0 Hz, $^{4}J_{FF}$ = 7.5 Hz) -110.9 (ddq, 1F, $^{2}J_{FF}$ = 312.1 Hz, $^{2}J_{FP}$ = 109.0 Hz, $^{4}J_{FF}$ = 7.5 Hz); **$^{31}$P {$^{1}$H} NMR**

($CDCl_3$, 161 MHz): δ 4.9 (dd, 1P, $^{2}J_{PF}$ = 111.1 Hz, $^{2}J_{PF}$ = 106.3 Hz). *Minor isomer:* **$^{1}$H NMR** ($CDCl_3$, 400 MHz): δ 7.21-7.13 (m, 2H), 7.04-7.01 (m, 2H), 4.22-4.07 (m, 4H), 2.84 (t, 1H, $^{3}J_{HH}$ = 8.0 Hz), 1.84-1.54 (m, 2H), 1.33 (t, 6H, $^{3}J_{HH}$ = 8.0 Hz), 1.17 (t, 3H, $^{4}J_{HH}$ = 8.0 Hz); **$^{13}$C NMR** ($CDCl_3$, 100 MHz): δ = 136.8, 130.3 (d, $^{3}J_{CH}$ = 11.0 Hz), 129.4, 123.3 (qd, $^{1}J_{CF}$ = 274.0 Hz, $^{1}J_{CP}$ = 4.0 Hz), 118.0 (td, $^{1}J_{CF}$ = 306.0 Hz, $^{1}J_{CP}$ = 217.0 Hz), 65.2-64.7 (m), 33.5-32.5 (m), 30.9, 29.7, 26.7, 16.3 (dt, $^{3}J_{CP}$ = 15.0 Hz, $^{3}J_{CP}$ = 6.0 Hz); **$^{19}$F NMR** ($CDCl_3$, 376 MHz): δ -64.8 (t, 3F, $^{4}J_{FF}$ = 7.5 Hz), -105.1 (ddq, 1F, $^{2}J_{FF}$ = 312.1 Hz, $^{2}J_{FP}$ = 109.0 Hz, $^{4}J_{FF}$ = 7.5 Hz) -106.2 (ddq, 1F, $^{2}J_{FF}$ = 312.1 Hz, $^{2}J_{FP}$ = 109.0 Hz, $^{4}J_{FF}$ = 7.5 Hz); **$^{31}$P {$^{1}$H} NMR** ($CDCl_3$, 161 MHz): δ 4.8 (t, 1P, $^{2}J_{PF}$ = 107.9 Hz). **HRMS** (ESI) *m/z*: $[M + H]^+$ calcd. for $C_{16}H_{21}F_5O_3P$ 387.1148; found 387.1141.

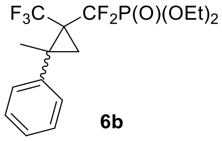

**6b**

Diethyl (2-methyl-2-phenyl-1-(trifluoromethyl)cyclopropyl)difluoromethylphosphonate (**6b**) was prepared from 0.5 g (0.2 mmol) of **5**, 0.032 g (0.02 mmol) of CuI, 0.3 mL (0.25 mmol) of α-methylstyrene and 7 mL of dry toluene. The reaction mixture was refluxed for 48 h to give compound **6b** in 50% yield as yellow oil. Mixture of isomers in 1.4:1 ratio. *Major isomer*: **$^{1}$H NMR** ($CDCl_3$, 400 MHz): δ 7.48 (d, 1H, $^{3}J_{HH}$ = 8.0 Hz), 7.23-7.04 (m, 4H), 4.31-4.14 (m, 4H), 1.95-1.61 (m, 2H), 1.56-1.48 (m, 3H), 1.31 (t, 6H, $^{3}J_{HH}$ = 8.0 Hz); **$^{13}$C NMR** ($CDCl_3$, 100 MHz): δ = 142.2, 129.9, 128.4, 126.9, 121.2 (qd, $^{1}J_{CF}$ = 275.0 Hz, $^{1}J_{CP}$ = 6.0 Hz), 118.4 (td, $^{1}J_{CF}$ = 288.0 Hz, $^{1}J_{CP}$ = 216.0 Hz), 65.1 (qd, $^{2}J_{CP}$ = 7.0 Hz, $^{2}J_{CP}$ = 2.0 Hz), 37.6-35.6 (m), 25.0 (q, $^{1}J_{CH}$ = 3.0 Hz), 19.6-19.5 (m), 16.3 (qd, $^{3}J_{CP}$ = 8.0 Hz, $^{3}J_{CP}$ = 6.0 Hz); **$^{19}$F NMR** ($CDCl_3$, 376 MHz): δ -58.1 (dd, 3F, $^{4}J_{FF}$ = 15.0 Hz, $^{4}J_{FP}$ = 7.5 Hz), -98.3 (ddq, 1F, $^{2}J_{FF}$ = 319.6 Hz, $^{2}J_{FP}$ = 109.0 Hz, $^{4}J_{FF}$ = 15.0 Hz), -101.5 (ddq, 1F, $^{2}J_{FF}$ = 319.6 Hz, $^{2}J_{FP}$ = 109.0 Hz, $^{4}J_{FF}$ = 15.0 Hz); **$^{31}$P {$^{1}$H} NMR** ($CDCl_3$, 161 MHz): δ 5.5 (dd, 1P, $^{2}J_{PF}$ = 107.8 Hz, $^{2}J_{PF}$ = 106.3 Hz).

*Minor isomer:* **$^1$H NMR** ($CDCl_3$, 400 MHz): δ 7.48 (d, 1H, $^3J_{HH}$ = 8.0 Hz), 7.23-7.04 (m, 4H), 4.14-3.88 (m, 4H), 1.95-1.61 (m, 2H), 1.56-1.48 (m, 3H), 1.18 (dt, 6H, $^3J_{HH}$ = 16.0 Hz, $^3J_{HH}$ = 8.0 Hz); **$^{13}$C NMR** ($CDCl_3$, 100 MHz): δ = 142.1, 129.6, 128.1, 126.7, 121.2 (qd, $^1J_{CF}$ = 275.0 Hz, $^1J_{CP}$ = 6.0 Hz), 118.4 (td, $^1J_{CF}$ = 288.0 Hz, $^1J_{CP}$ = 216.0 Hz), 64.7 (qd, $^2J_{CP}$ = 7.0 Hz, $^2J_{CP}$ = 2.0 Hz), 37.6-35.6 (m), 24.7 (dd, $^1J_{CH}$ = 10.0 Hz, $^1J_{CH}$ = 3.0 Hz), 18.0-17.8 (m), 16.3 (qd, $^3J_{CP}$ = 8.0 Hz, $^3J_{CP}$ = 6.0 Hz); **$^{19}$F NMR** ($CDCl_3$, 376 MHz): δ -55.8 (td, 3F, $^4J_{FF}$ = 7.5 Hz, $^4J_{FP}$ = 3.8 Hz), -99.4 (ddq, 1F, $^2J_{FF}$ = 319.6 Hz, $^2J_{FP}$ = 109.0 Hz, $^4J_{FF}$ = 15.0 Hz) -104.8 (ddq, 1F, $^2J_{FF}$ = 319.6 Hz, $^2J_{FP}$ = 109.0 Hz, $^4J_{FF}$ = 15.0 Hz); **$^{31}$P {$^1$H} NMR** ($CDCl_3$, 161 MHz): δ 6.1 (t, 1P, $^2J_{PF}$ = 109.5 Hz). **HRMS** (ESI) *m/z*: $[M + H]^+$ calcd. for $C_{16}H_{21}F_5O_3P$ 387.1149; found 387.1141.

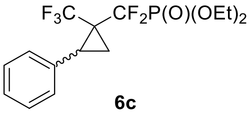


**6c**

Diethyl (2-phenyl-1-(trifluoromethyl)cyclopropyl)difluoromethylphosphonate (**6c**) was prepared from 0.84 g (0.3 mmol) of **5**, 0.053 g (0.03 mmol) of CuI, 0.49 mL (0.4 mmol) of styrene and 11 mL of dry toluene. The reaction mixture was refluxed for 4 h to give compound **6c** in 35% yield as yellow oil. Mixture of isomers in 1:3 ratio. *Major isomer:* **$^1$H NMR** ($CDCl_3$, 400 MHz): δ 7.30 (d, 2H, $^3J_{HH}$ = 8.0 Hz), 7.25-7.16 (m, 3H), 4.34-4.12 (m, 4H), 2.98 (t, 1H, $^4J_{HF}$ = 8.0 Hz), 1.76-1.69 (m, 2H), 1.33 (t, 6H, $^3J_{HH}$ = 8.0 Hz); **$^{13}$C NMR** ($CDCl_3$, 100 MHz): δ = 133.3, 129.4, 128.2, 127.5, 124.4 (qd, $^1J_{CF}$ = 275.0 Hz, $^1J_{CP}$ = 6.0 Hz), 117.4 (td, $^1J_{CF}$ = 260.0 Hz, $^1J_{CP}$ = 218.0 Hz), 65.2 (dd, $^2J_{CP}$ = 18.0 Hz, $^2J_{CP}$ = 8.0 Hz), 33.5-32.4 (m), 26.1 (t, $^1J_{CH}$ = 5.0 Hz), 24.7, 16.4 (dd, $^3J_{CP}$ = 8.0 Hz, $^3J_{CP}$ = 6.0 Hz); **$^{19}$F NMR** ($CDCl_3$, 376 MHz): δ -59.1 (t, 3F, $^4J_{FF}$ = 7.5 Hz), -106.9 (ddq, 1F, $^2J_{FF}$ = 315.8 Hz, $^2J_{FP}$ = 105.3 Hz, $^4J_{FF}$ = 7.5 Hz), -111.0 (ddq, 1F, $^2J_{FF}$ = 315.8 Hz, $^2J_{FP}$ = 105.3 Hz, $^4J_{FF}$ = 7.5 Hz); **$^{31}$P {$^1$H} NMR** ($CDCl_3$, 161 MHz): δ 4.9 (dd, 1P, $^2J_{PF}$ = 109.5 Hz, $^2J_{PF}$ = 106.3 Hz). *Minor isomer:* **$^1$H NMR** ($CDCl_3$, 400 MHz): δ 7.30 (d, 2H, $^3J_{HH}$ = 8.0 Hz), 7.25-7.16 (m, 3H), 4.34-4.12 (m, 4H), 2.98 (t, 1H, $^4J_{HF}$ =

8.0 Hz), 1.76-1.69 (m, 2H), 1.33 (t, 6H, $^3J_{HH}$ = 8.0 Hz); **$^{13}$C NMR** ($CDCl_3$, 100 MHz): δ = 133.3, 129.4, 128.2, 127.5, 124.4 (qd, $^1J_{CF}$ = 275.0 Hz, $^1J_{CP}$ = 6.0 Hz), 117.4 (dd, $^1J_{CF}$ = 260.0 Hz, $^1J_{CP}$ = 218.0 Hz), 65.2 (dd, $^2J_{CP}$ = 18.0 Hz, $^2J_{CP}$ = 8.0 Hz), 33.5-32.4 (m), 26.1 (t, $^1J_{CH}$ = 5.0 Hz), 24.7, 16.4 (dd, $^3J_{CP}$ = 8.0 Hz, $^3J_{CP}$ = 6.0 Hz); **$^{19}$F NMR** ($CDCl_3$, 376 MHz): δ -64.8 (t, 3F, $^4J_{FF}$ = 7.5 Hz), -104.6 (ddq, 1F, $^2J_{FF}$ = 315.8 Hz, $^2J_{FP}$ = 105.3 Hz, $^4J_{FF}$ = 7.5 Hz), -106.5 (ddq, 1F, $^2J_{FF}$ = 315.8 Hz, $^2J_{FP}$ = 105.3 Hz, $^4J_{FF}$ = 7.5 Hz); **$^{31}$P {$^1$H} NMR** ($CDCl_3$, 161 MHz): δ 4.7 (t, 1P, $^2J_{PF}$ = 106.3 Hz). **HRMS** (ESI) *m/z*: $[M + H]^+$ calcd. for $C_{15}H_{19}F_5O_3P$ 373.0992; found 373.0991.

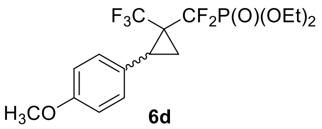

**6d**

Diethyl (2-(4-methoxyphenyl)-1-(trifluoromethyl)cyclopropyl)difluoromethylphosphonate (**6d**) was prepared from 0.3 g (0.1 mmol) of **5**, 0.019 g (0.01 mmol) of CuI, 0.2 mL (0.15 mmol) of 4-methoxystyrene and 5 mL of dry toluene. The reaction mixture was refluxed for 2.5 h to give compound **6d** in 67% yield as yellow oil. Mixture of isomers in 1:1.3 ratio. *Major isomer:* **$^1$H NMR** ($CDCl_3$, 400 MHz): δ 7.22-7.19 (m, 2H), 6.78-6.74 (m, 2H), 4.34-4.18 (m, 4H), 3.71 (s, 3H), 2.91 (t, 1H, $^4J_{HF}$ = 8.0 Hz), 1.73-1.65 (m, 2H), 1.33 (t, 6H, $^3J_{HH}$ = 8.0 Hz); **$^{13}$C NMR** ($CDCl_3$, 100 MHz): δ = 158.9, 130.5, 128.6, 124.3 (qd, $^1J_{CF}$ = 274.0 Hz, $^1J_{CP}$ = 5.0 Hz), 117.3 (dt, $^1J_{CF}$ = 260.0 Hz, $^1J_{CP}$ = 219.0 Hz), 65.2 (dd, $^2J_{CP}$ = 20.0 Hz, $^2J_{CP}$ = 7.0 Hz), 55.2, 39.9-39.3 (m), 33.3-32.3 (m), 25.4 (t, $^1J_{CH}$ = 4.0 Hz), 16.4 (dd, $^3J_{CP}$ = 9.0 Hz, $^3J_{CP}$ = 6.0 Hz); **$^{19}$F NMR** ($CDCl_3$, 376 MHz): δ -59.0 (t, 3F, $^4J_{FF}$ = 7.5 Hz), -106.7 (ddq, 1F, $^2J_{FF}$ = 315.8 Hz, $^2J_{FP}$ = 105.3 Hz, $^4J_{FF}$ = 7.5 Hz), -111.1 (ddq, 1F, $^2J_{FF}$ = 315.8 Hz, $^2J_{FP}$ = 105.3 Hz, $^4J_{FF}$ = 7.5 Hz); **$^{31}$P {$^1$H} NMR** ($CDCl_3$, 161 MHz): δ 4.9 (dd, 1P, $^2J_{PF}$ = 111.1 Hz, $^2J_{PF}$ = 106.3 Hz). *Minor isomer:* **$^1$H NMR** ($CDCl_3$, 400 MHz): δ 7.23-7.16 (m, 2H), 6.78-6.74 (m, 2H), 4.19-3.98 (m, 4H), 3.71 (s, 3H), 2.83 (t, 1H, $^4J_{HF}$ = 8.0 Hz), 1.80-1.55 (m, 2H), 1.23 (tdd, 6H, $^3J_{HH}$ = 8.0 Hz,

${}^{4}J_{HP}$ = 4.0 Hz, ${}^{4}J_{HP}$ = 4.0 Hz); **$^{13}$C NMR** ($CDCl_3$, 100 MHz): δ = 158.9, 130.5, 128.6, 124.3 (qd, ${}^{1}J_{CF}$ = 274.0 Hz, ${}^{1}J_{CP}$ = 5.0 Hz), 117.3 (td, ${}^{1}J_{CF}$ = 260.0 Hz, ${}^{1}J_{CP}$ = 219.0 Hz), 65.2 (dd, ${}^{2}J_{CP}$ = 20.0 Hz, ${}^{2}J_{CP}$ = 7.0 Hz), 55.2, 39.9-39.3 (m), 33.3-32.3 (m), 25.4 (t, ${}^{1}J_{CH}$ = 4.0 Hz), 16.4 (dd, ${}^{3}J_{CP}$ = 9.0 Hz, ${}^{3}J_{CP}$ = 6.0 Hz); **$^{19}$F NMR** ($CDCl_3$, 376 MHz): δ -64.7 (t, 3F, ${}^{4}J_{FF}$ = 7.5 Hz), -104.8 (ddq, 1F, ${}^{2}J_{FF}$ = 315.8 Hz, ${}^{2}J_{FP}$ = 105.3 Hz, ${}^{4}J_{FF}$ = 7.5 Hz), -106.1 (ddq, 1F, ${}^{2}J_{FF}$ = 315.8 Hz, ${}^{2}J_{FP}$ = 105.3 Hz, ${}^{4}J_{FF}$ = 7.5 Hz); **$^{31}$P {$^{1}$H} NMR** ($CDCl_3$, 161 MHz): δ 4.8 (t, 1P, ${}^{2}J_{PF}$ = 106.3 Hz). **HRMS** (ESI) *m/z*: $[M + H]^+$ calcd. for $C_{16}H_{21}F_5O_4P$ 403.1097; found 403.1092.

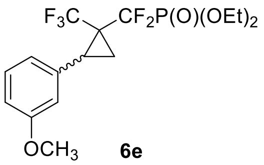

**6e**

Diethyl (2-(3-methoxyphenyl)-1-(trifluoromethyl)cyclopropyl)difluoromethylphosphonate (**6e**) was prepared from 0.3 g (0.1 mmol) of **5**, 0.019 g (0.01 mmol) of CuI, 0.2 mL (0.15 mmol) of 3-methoxystyrene and 5 mL of dry toluene. The reaction mixture was refluxed for 7 h to give compound **6e** in 41% yield as yellow oil. Mixture of isomers in 1:1.9 ratio. *Major isomer:* **$^{1}$H NMR** ($CDCl_3$, 400 MHz): δ 7.16-7.11 (m, 1H), 6.90-6.84 (m, 2H), 6.74-6.70 (m, 1H), 4.32-4.20 (m, 4H), 3.71 (s, 3H), 2.96 (t, 1H, ${}^{4}J_{HF}$ = 8.0 Hz), 1.80-1.66 (m, 2H), 1.33 (t, 6H, ${}^{3}J_{HH}$ = 8.0 Hz); **$^{13}$C NMR** ($CDCl_3$, 100 MHz): δ = 159.5, 135.2, 129.2, 120.7 (qd, ${}^{1}J_{CF}$ = 274.0 Hz, ${}^{1}J_{CP}$ = 5.0 Hz), 117.3 (td, ${}^{1}J_{CF}$ = 260.0 Hz, ${}^{1}J_{CP}$ = 213.0 Hz), 65.2 (dd, ${}^{2}J_{CP}$ = 15.0 Hz, ${}^{2}J_{CP}$ = 7.0 Hz), 55.2 (d, ${}^{5}J_{CH}$ = 3.0 Hz), 33.3-32.5 (m), 26.1 (t, ${}^{1}J_{CH}$ = 4.0 Hz), 16.4 (dd, ${}^{3}J_{CP}$ = 7.0 Hz, ${}^{3}J_{CP}$ = 5.0 Hz); **$^{19}$F NMR** ($CDCl_3$, 376 MHz): δ -59.2 (t, 3F, ${}^{4}J_{FF}$ = 7.5 Hz), -106.9 (ddq, 1F, ${}^{2}J_{FF}$ = 315.8 Hz, ${}^{2}J_{FP}$ = 105.3 Hz, ${}^{4}J_{FF}$ = 7.5 Hz), -110.8 (ddq, 1F, ${}^{2}J_{FF}$ = 315.8 Hz, ${}^{2}J_{FP}$ = 105.3 Hz, ${}^{4}J_{FF}$ = 7.5 Hz); **$^{31}$P {$^{1}$H} NMR** ($CDCl_3$, 161 MHz): δ 4.9 (dd, 1P, ${}^{2}J_{PF}$ = 109.5 Hz, ${}^{2}J_{PF}$ = 106.3 Hz). *Minor isomer:* **$^{1}$H NMR** ($CDCl_3$, 400 MHz): δ 7.16-7.11 (m, 1H), 6.90-6.84 (m, 2H), 6.74-6.70 (m, 1H), 4.19-4.00 (m, 4H), 3.72 (s, 3H), 2.87 (t, 1H, ${}^{4}J_{HF}$ = 8.0 Hz), 1.80-1.66 (m, 2H), 1.23 (td, 6H, ${}^{3}J_{HH}$ = 8.0 Hz, ${}^{3}J_{HH}$ = 4.0 Hz); **$^{13}$C NMR** ($CDCl_3$, 100 MHz): δ = 159.4,

134.9, 129.0, 120.7 (qd, $^{1}J_{CF}$ = 274.0 Hz, $^{1}J_{CP}$ = 5.0 Hz), 117.5 (td, $^{1}J_{CF}$ = 260.0 Hz, $^{1}J_{CP}$ = 213.0 Hz), 64.8 (t, $^{2}J_{CP}$ = 6.0 Hz), 55.3 (d, $^{5}J_{CH}$ = 4.0 Hz), 33.3-32.5 (m), 27.1,16.2 (dd, $^{3}J_{CP}$ = 11.0 Hz, $^{3}J_{CP}$ = 5.0 Hz); **$^{19}$F NMR** ($CDCl_3$, 376 MHz): δ -64.8 (t, 3F, $^{4}J_{FF}$ = 3.8 Hz), -104.7 (ddq, 1F, $^{2}J_{FF}$ = 315.8 Hz, $^{2}J_{FP}$ = 105.3 Hz, $^{4}J_{FF}$ = 7.5 Hz), -106.6 (ddq, 1F, $^{2}J_{FF}$ = 315.8 Hz, $^{2}J_{FP}$ = 105.3 Hz, $^{4}J_{FF}$ = 7.5 Hz); **$^{31}$P {$^{1}$H} NMR** ($CDCl_3$, 161 MHz): δ 4.8 (t, 1P, $^{2}J_{PF}$ = 106.3 Hz). **HRMS** (ESI) *m/z*: $[M + H]^+$ calcd. for $C_{16}H_{21}F_5O_4P$ 403.1097; found 403.1092.

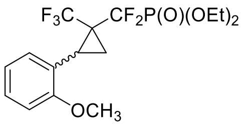


**6f**

Diethyl (2-(2-methoxyphenyl)-1-(trifluoromethyl)cyclopropyl)difluoromethylphosphonate (**6f**) was prepared from 0.3 g (0.1 mmol) of **5**, 0.019 g (0.01 mmol) of CuI, 0.2 mL (0.15 mmol) of 2-methoxystyrene and 5 mL of dry toluene. The reaction mixture was refluxed for 7 h to give compound **6f** in 53% yield as yellow oil. Mixture of isomers in 1:1.4 ratio. *Major isomer:* **$^{1}$H NMR** ($CDCl_3$, 400 MHz): δ 7.19-7.14 (m, 1H), 7.07-7.02 (m, 1H), 6.82-6.76 (m, 2H), 4.31-4.17 (m, 4H), 3.75 (s, 3H), 2.97 (t, 1H, $^{4}J_{HF}$ = 8.0 Hz), 1.83-1.70 (m, 2H), 1.31 (td, 6H, $^{3}J_{HH}$ = 8.0 Hz, $^{3}J_{HH}$ = 4.0 Hz); **$^{13}$C NMR** ($CDCl_3$, 100 MHz): δ = 159.2, 129.5, 128.9, 124.0 (qd, $^{1}J_{CF}$ = 273.0 Hz, $^{1}J_{CP}$ = 4.0 Hz),118.4 (dt, $^{1}J_{CF}$ = 296.0 Hz, $^{1}J_{CP}$ = 269.0 Hz), 65.2-65.0 (m), 55.3, 33.1-33.7 (m), 29.8.29.2 (m), 23.2, 16.4 (dq, $^{3}J_{CP}$ = 6.0 Hz, $^{3}J_{CP}$ = 2.0 Hz); **$^{19}$F NMR** ($CDCl_3$, 376 MHz): δ -60.1 (t, 3F, $^{4}J_{FF}$ = 7.5 Hz), -106.0 (ddq, 1F, $^{2}J_{FF}$ = 315.8 Hz, $^{2}J_{FP}$ = 105.3 Hz, $^{4}J_{FF}$ = 7.5 Hz), -108.2 (ddq, 1F, $^{2}J_{FF}$ = 315.8 Hz, $^{2}J_{FP}$ = 105.3 Hz, $^{4}J_{FF}$ = 7.5 Hz); **$^{31}$P {$^{1}$H} NMR** ($CDCl_3$, 161 MHz): δ 4.8 (t, 1P, $^{2}J_{PF}$ = 109.5 Hz). *Minor isomer:* **$^{1}$H NMR** ($CDCl_3$, 400 MHz): δ 7.19-7.14 (m, 1H), 7.07-7.02 (m, 1H), 6.82-6.76 (m, 2H), 4.15-3.97 (m, 4H), 3.77 (s, 3H), 2.77 (t, 1H, $^{4}J_{HF}$ = 8.0 Hz), 1.83-1.70 (m, 2H), 1.21 (dt, 6H, $^{3}J_{HH}$ = 12.0 Hz, $^{3}J_{HH}$ = 8.0 Hz); **$^{13}$C NMR** ($CDCl_3$, 100 MHz): δ = 159.1, 129.5, 128.6, 124.0 (qd, $^{1}J_{CF}$ = 273.0 Hz, $^{1}J_{CP}$ = 4.0 Hz), 118.1 (dt, $^{1}J_{CF}$ = 296.0 Hz, $^{1}J_{CP}$ = 269.0 Hz), 64.8 (dd, $^{2}J_{CP}$ = 14.0 Hz, $^{2}J_{CP}$ = 7.0 Hz),

55.2, 33.1-33.7 (m), 29.8-29.2 (m), 22.8, 16.2 (dd, ${}^3J_{CP}$ = 6.0 Hz, ${}^3J_{CP}$ = 1.0 Hz); **$^{19}$F NMR** ($CDCl_3$, 376 MHz): δ -64.9 (t, 3F, ${}^4J_{FF}$ = 7.5 Hz), -107.0 (ddq, 1F, ${}^2J_{FF}$ = 315.8 Hz, ${}^2J_{FP}$ = 105.3 Hz, ${}^4J_{FF}$ = 7.5 Hz), -109.2 (ddq, 1F, ${}^2J_{FF}$ = 315.8 Hz, ${}^2J_{FP}$ = 105.3 Hz, ${}^4J_{FF}$ = 7.5 Hz); **$^{31}$P {$^1$H} NMR** ($CDCl_3$, 161 MHz): δ 5.0 (t, 1P, ${}^2J_{PF}$ = 109.5 Hz). **HRMS** (ESI) *m/z*: $[M + H]^+$ calcd. for $C_{16}H_{21}F_5O_4P$ 403.1097; found 403.1092.

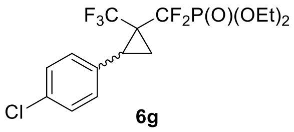

**6g**

Diethyl (2-(4-chlorophenyl)-1-(trifluoromethyl)cyclopropyl)difluoromethylphosphonate (**6g**) was prepared from 0.3 g (0.1 mmol) of **5**, 0.019 g (0.01 mmol) of CuI, 0.2 mL (0.15 mmol) of 4-chlorstyrene and 5 mL of dry toluene. The reaction mixture was refluxed for 3 h to give compound **6g** in 45% yield as yellow oil. Mixture of isomers in 1:1.5 ratio. *Major isomer:* **$^1$H NMR** ($CDCl_3$, 400 MHz): δ 7.24-7.19 (m, 4H), 4.33-4.21 (m, 4H), 2.92 (t, 1H, ${}^4J_{HF}$ = 8.0 Hz), 1.77-1.66 (m, 2H), 1.35 (tdd, 6H, ${}^3J_{HH}$ = 8.0 Hz, ${}^3J_{HH}$ = 4.0 Hz, ${}^3J_{HH}$ = 4.0 Hz); **$^{13}$C NMR** ($CDCl_3$, 100 MHz): δ = 133.4, 131.9, 130.8, 123.6 (qd, ${}^1J_{CF}$ = 275.0 Hz, ${}^1J_{CP}$ = 5.0 Hz), 117.2 (td, ${}^1J_{CF}$ = 260.0 Hz, ${}^1J_{CP}$ = 217.0 Hz), 65.3 (dd, ${}^2J_{CP}$ = 20.0 Hz, ${}^2J_{CP}$ = 7.0 Hz), 33.5-32.5 (m), 30.3-29.4 (m), 25.4 (t, ${}^1J_{CH}$ = 5.0 Hz), 16.4 (dd, ${}^3J_{CP}$ = 12.0 Hz, ${}^3J_{CP}$ = 6.0 Hz); **$^{19}$F NMR** ($CDCl_3$, 376 MHz): δ -59.1 (t, 3F, ${}^4J_{FF}$ = 7.5 Hz), -107.0 (ddq, 1F, ${}^2J_{FF}$ = 315.8 Hz, ${}^2J_{FP}$ = 105.3 Hz, ${}^4J_{FF}$ = 7.5 Hz), -111.7 (ddq, 1F, ${}^2J_{FF}$ = 315.8 Hz, ${}^2J_{FP}$ = 105.3 Hz, ${}^4J_{FF}$ = 7.5 Hz); **$^{31}$P {$^1$H} NMR** ($CDCl_3$, 161 MHz): δ 4.7 (dd, 1P, ${}^2J_{PF}$ = 111.1 Hz, ${}^2J_{PF}$ = 104.7 Hz). *Minor isomer:* **$^1$H NMR** ($CDCl_3$, 400 MHz): δ 7.26-7.19 (m, 4H), 4.20-3.99 (m, 4H), 2.83 (t, 1H, ${}^4J_{HF}$ = 8.0 Hz), 1.81-1.54 (m, 2H), 1.24 (td, 6H, ${}^3J_{HH}$ = 8.0 Hz, ${}^3J_{HH}$ = 4.0 Hz); **$^{13}$C NMR** ($CDCl_3$, 100 MHz): δ = 133.4, 131.9, 130.8, 123.6 (qd, ${}^1J_{CF}$ = 275.0 Hz, ${}^1J_{CP}$ = 5.0 Hz), 117.2 (td, ${}^1J_{CF}$ = 260.0 Hz, ${}^1J_{CP}$ = 217.0 Hz), 65.3 (dd, ${}^2J_{CP}$ = 20.0 Hz, ${}^2J_{CP}$ = 7.0 Hz), 33.5-32.5 (m), 30.3-29.4 (m), 25.4 (t, ${}^1J_{CH}$ = 5.0 Hz), 16.4 (dd, ${}^3J_{CP}$ = 12.0 Hz, ${}^3J_{CP}$ = 6.0 Hz); **$^{19}$F NMR** ($CDCl_3$,

376 MHz): δ -64.9 (t, 3F, $^4J_{FF}$ = 7.5 Hz), -104.3 (ddq, 1F, $^2J_{FF}$ = 315.8 Hz, $^2J_{FP}$ = 105.3 Hz, $^4J_{FF}$ = 7.5 Hz), -106.3 (ddq, 1F, $^2J_{FF}$ = 315.8 Hz, $^2J_{FP}$ = 105.3 Hz, $^4J_{FF}$ = 7.5 Hz); **$^{31}$P {$^1$H} NMR** ($CDCl_3$, 161 MHz): δ 4.5 (t, 1P, $^2J_{PF}$ = 106.3 Hz). **HRMS** (ESI) *m/z*: $[M + H]^+$ calcd. for $C_{15}H_{18}ClF_5O_3P$ 407.0602; found 407.0596.

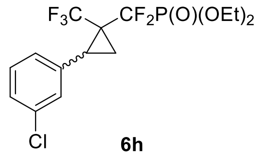


**6h**

Diethyl (2-(3-chlorophenyl)-1-(trifluoromethyl)cyclopropyl)difluoromethylphosphonate (**6h**) was prepared from 0.3 g (0.1 mmol) of **5**, 0.019 g (0.01 mmol) of CuI, 0.2 mL (0.15 mmol) of 3-chlorstyrene and 5 mL of dry toluene. The reaction mixture was refluxed for 5 h to give compound **6h** in 41% yield as yellow oil. *Major isomer:* **$^1$H NMR** ($CDCl_3$, 400 MHz): δ 7.24-7.08 (m, 4H), 4.35-4.22 (m, 4H), 2.93 (t, 1H, $^4J_{HF}$ = 8.0 Hz), 1.78-1.68 (m, 2H), 1.35 (t, 6H, $^3J_{HH}$ = 8.0 Hz); **$^{13}$C NMR** ($CDCl_3$, 100 MHz): δ = 135.5, 134.1, 129.5, 123.7 (qd, $^1J_{CF}$ = 274.0 Hz, $^1J_{CP}$ = 6.0 Hz), 117.1 (td, $^1J_{CF}$ = 261.0 Hz, $^1J_{CP}$ = 217.0 Hz), 65.3 (dd, $^2J_{CP}$ = 17.0 Hz, $^2J_{CP}$ = 8.0 Hz), 33.5-32.6 (m), 29.8, 25.6 (t, $^1J_{CH}$ = 4.0 Hz), 16.4 (dd, $^3J_{CP}$ = 9.0 Hz, $^3J_{CP}$ = 6.0 Hz); **$^{19}$F NMR** ($CDCl_3$, 376 MHz): δ -59.2 (t, 3F, $^4J_{FF}$ = 7.5 Hz), -107.2 (ddq, 1F, $^2J_{FF}$ = 315.8 Hz, $^2J_{FP}$ = 105.3 Hz, $^4J_{FF}$ = 7.5 Hz), -111.4 (ddq, 1F, $^2J_{FF}$ = 315.8 Hz, $^2J_{FP}$ = 105.3 Hz, $^4J_{FF}$ = 7.5 Hz); **$^{31}$P {$^1$H} NMR** ($CDCl_3$, 161 MHz): δ 4.7 (dd, 1P, $^2J_{PF}$ = 109.5 Hz, $^2J_{PF}$ = 106.3 Hz). *Minor isomer:* **$^1$H NMR** ($CDCl_3$, 400 MHz): δ 7.29-7.27 (m, 2H), 7.18-7.16 (m, 2H), 4.20-4.03 (m, 4H), 2.85 (t, 1H, $^4J_{HF}$ = 8.0 Hz), 1.82-1.59 (m, 2H), 1.25 (tdd, 6H, $^3J_{HH}$ = 8.0 Hz, $^3J_{HH}$ = 4.0 Hz, $^3J_{HH}$ = 4.0 Hz); **$^{13}$C NMR** ($CDCl_3$, 100 MHz): δ = 135.5, 134.1, 129.5, 123.7 (qd, $^1J_{CF}$ = 274.0 Hz, $^1J_{CP}$ = 6.0 Hz), 117.1 (td, $^1J_{CF}$ = 261.0 Hz, $^1J_{CP}$ = 217.0 Hz), 65.3 (dd, $^2J_{CP}$ = 17.0 Hz, $^2J_{CP}$ = 8.0 Hz), 33.5-32.6 (m), 29.8, 25.6 (t, $^1J_{CH}$ = 4.0 Hz), 16.4 (dd, $^3J_{CP}$ = 9.0 Hz, $^3J_{CP}$ = 6.0 Hz); **$^{19}$F NMR** ($CDCl_3$, 376 MHz): δ -64.9 (t, 3F, $^4J_{FF}$ = 7.5 Hz), -104.3 (ddq, 1F, $^2J_{FF}$ = 315.8 Hz, $^2J_{FP}$ = 105.3 Hz, $^4J_{FF}$ = 7.5 Hz), -106.5 (ddq, 1F, $^2J_{FF}$ = 315.8 Hz, $^2J_{FP}$ = 105.3 Hz,

$^{4}J_{FF}$ = 7.5 Hz); **$^{31}$P {$^{1}$H} NMR** ($CDCl_3$, 161 MHz): δ 4.6 (t, 1P, $^{2}J_{PF}$ = 106.3 Hz). **HRMS** (ESI) *m/z*: $[M + H]^+$ calcd. for $C_{15}H_{18}ClF_5O_3P$ 407.0602; found 407.0596.

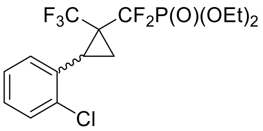


**6i**

Diethyl (2-(2-chlorophenyl)-1-(trifluoromethyl)cyclopropyl)difluoromethylphosphonate (**6i**) was prepared from 0.3 g (0.1 mmol) of **5**, 0.019 g (0.01 mmol) of CuI, 0.2 mL (0.15 mmol) of 2-chlorstyrene and 5 mL of dry toluene. The reaction mixture was refluxed for 8 h to give compound **6i** in 53% yield as yellow oil. Mixture of isomers in 1:2.2 ratio. *Major isomer:* **$^{1}$H NMR** ($CDCl_3$, 400 MHz): δ 7.24-7.14 (m, 4H), 4.30-4.20 (m, 4H), 3.09 (t, 1H, $^{4}J_{HF}$ = 8.0 Hz), 1.93-1.80 (m, 2H), 1.33 (td, 6H, $^{3}J_{HH}$ = 8.0 Hz, $^{3}J_{HH}$ = 4.0 Hz); **$^{13}$C NMR** ($CDCl_3$, 100 MHz): δ = 136.2, 131.6, 130.3, 123.8 (qd, $^{1}J_{CF}$ = 275.0 Hz, $^{1}J_{CP}$ = 5.0 Hz), 117.8 (td, $^{1}J_{CF}$ = 262.0 Hz, $^{1}J_{CP}$ = 217.0 Hz), 65.0 (dd, $^{2}J_{CP}$ = 11.0 Hz, $^{2}J_{CP}$ = 6.0 Hz), 33.9-32.7 (m), 29.5, 24.4 (t, $^{1}J_{CH}$ = 4.0 Hz), 16.3 (dd, $^{3}J_{CP}$ = 11.0 Hz, $^{3}J_{CP}$ = 6.0 Hz); **$^{19}$F NMR** ($CDCl_3$, 376 MHz): δ -59.8 (t, 3F, $^{4}J_{FF}$ = 7.5 Hz), -105.2 (ddq, 1F, $^{2}J_{FF}$ = 315.8 Hz, $^{2}J_{FP}$ = 109.0 Hz, $^{4}J_{FF}$ = 7.5 Hz), -109.0 (ddq, 1F, $^{2}J_{FF}$ = 315.8 Hz, $^{2}J_{FP}$ = 109.0 Hz, $^{4}J_{FF}$ = 7.5 Hz); **$^{31}$P {$^{1}$H} NMR** ($CDCl_3$, 161 MHz): δ 4.5 (t, 1P, $^{2}J_{PF}$ = 106.3 Hz). *Minor isomer:* **$^{1}$H NMR** ($CDCl_3$, 400 MHz): δ 7.34-7.31 (m, 1H), 7.20-7.10 (m, 3H), 4.20-4.01 (m, 4H), 2.88 (t, 1H, $^{4}J_{HF}$ = 8.0 Hz), 1.95-1.64 (m, 2H), δ 1.25 (dt, 6H, $^{3}J_{HH}$ = 16.0 Hz, $^{3}J_{HH}$ = 8.0 Hz); **$^{13}$C NMR** ($CDCl_3$, 100 MHz): δ = 136.2, 131.6, 130.3, 123.8 (qd, $^{1}J_{CF}$ = 275.0 Hz, $^{1}J_{CP}$ = 5.0 Hz), 117.8 (td, $^{1}J_{CF}$ = 262.0 Hz, $^{1}J_{CP}$ = 217.0 Hz), 65.0 (dd, $^{2}J_{CP}$ = 11.0 Hz, $^{2}J_{CP}$ = 6.0 Hz), 33.9-32.7 (m), 29.5, 24.4 (t, $^{1}J_{CH}$ = 4.0 Hz), 16.3 (dd, $^{3}J_{CP}$ = 11.0 Hz, $^{3}J_{CP}$ = 6.0 Hz); **$^{19}$F NMR** ($CDCl_3$, 376 MHz): δ -64.8 (t, 3F, $^{4}J_{FF}$ = 7.5 Hz), -105.6 (ddq, 1F, $^{2}J_{FF}$ = 315.8 Hz, $^{2}J_{FP}$ = 109.0 Hz, $^{4}J_{FF}$ = 7.5 Hz), -108.5 (ddq, 1F, $^{2}J_{FF}$ = 315.8 Hz, $^{2}J_{FP}$ = 109.0 Hz, $^{4}J_{FF}$ = 7.5 Hz); **$^{31}$P {$^{1}$H} NMR** ($CDCl_3$, 161 MHz): δ 4.5 (t, 1P, $^{2}J_{PF}$ = 107.9 Hz). **HRMS** (ESI) *m/z*: $[M + H]^+$ calcd. for $C_{15}H_{18}ClF_5O_3P$ 407.0602; found 407.0596.

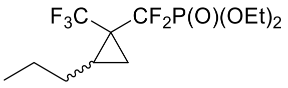

**7a**

Diethyl (2-propyl-1-(trifluoromethyl)cyclopropyl)difluoromethylphosphonate (**7a**) was prepared from 0.3 g (0.1 mmol) of **5**, 0.019 g (0.01 mmol) of CuI, 0.16 mL (0.15 mmol) of 1-pentene and 5 mL of dry toluene. The reaction mixture was refluxed for 3.5 h to give compound **7a** in 48% yield as yellow oil. Mixture of isomers in 1:1.2 ratio. *Major isomer:* **$^{1}$H NMR** ($CDCl_3$, 400 MHz): δ 4.29-4.15 (m, 4H), 2.31-1.54 (m, 2H), 1.47-1.21 (m, 10H), 1.16-1.11 (3, 1H), 0.89 (t, 3H, $^{3}J_{HH}$ = 8.0 Hz); **$^{13}$C NMR** ($CDCl_3$, 100 MHz): δ = 124.0 (qd, $^{1}J_{CF}$ = 274.0 Hz, $^{1}J_{CP}$ = 6.0 Hz), 117.9 (td, $^{1}J_{CF}$ = 309.0 Hz, $^{1}J_{CP}$ = 216.0 Hz), 65.1-64.8 (m), 31.0-30.5 (m), 28.7 (t, $^{2}J_{CF}$ = 5.0 Hz), 23.5 (q, $^{3}J_{CH}$ = 2.0 Hz), 23.0, 22.6, 22.2 (t, $^{3}J_{CH}$ = 4.0 Hz), 16.4 (dd, $^{3}J_{CP}$ = 10.0 Hz, $^{3}J_{CP}$ = 4.0 Hz); **$^{19}$F NMR** ($CDCl_3$, 376 MHz): δ -57.8 (s, 3F), -106.8 (ddq, 1F, $^{2}J_{FF}$ = 323.4 Hz, $^{2}J_{FP}$ = 109.0 Hz, $^{4}J_{FF}$ = 7.5 Hz), -110.3 (ddq, 1F, $^{2}J_{FF}$ = 323.4 Hz, $^{2}J_{FP}$ = 109.0 Hz, $^{4}J_{FF}$ = 7.5 Hz); **$^{31}$P {$^{1}$H} NMR** ($CDCl_3$, 161 MHz): δ 5.1 (t, 1P, $^{2}J_{PF}$ = 109.5 Hz). *Minor isomer:* **$^{1}$H NMR** ($CDCl_3$, 400 MHz): δ 4.29-4.15 (m, 4H), 2.31-1.54 (m, 2H), 1.47-1.21 (m, 10H), 1.03 (t, 1H, $^{4}J_{HF}$ = 8.0 Hz), 0.89 (t, 3H, $^{3}J_{HH}$ = 8.0 Hz); **$^{13}$C NMR** ($CDCl_3$, 100 MHz): δ = 124.0 (qd, $^{1}J_{CF}$ = 274.0 Hz, $^{1}J_{CP}$ = 6.0 Hz), 117.9 (td, $^{1}J_{CF}$ = 309.0 Hz, $^{1}J_{CP}$ = 216.0 Hz), 65.1-64.8 (m), 31.0-30.5 (m), 28.7 (t, $^{2}J_{CF}$ = 5.0 Hz), 23.5 (q, $^{3}J_{CH}$ = 2.0 Hz), 23.0, 22.6, 22.2 (t, $^{3}J_{CH}$ = 4.0 Hz), 16.4 (dd, $^{3}J_{CP}$ = 10.0 Hz, $^{3}J_{CP}$ = 4.0 Hz); **$^{19}$F NMR** ($CDCl_3$, 376 MHz): δ -64.8 (t, 3F, $^{4}J_{FF}$ = 3.8 Hz), -102.1, (ddq, 1F, $^{2}J_{FF}$ = 323.4 Hz, $^{2}J_{FP}$ = 109.0 Hz, $^{4}J_{FF}$ = 7.5 Hz), -104.2 (ddq, 1F, $^{2}J_{FF}$ = 323.4 Hz, $^{2}J_{FP}$ = 109.0 Hz, $^{4}J_{FF}$ = 7.5 Hz); **$^{31}$P {$^{1}$H} NMR** ($CDCl_3$, 161 MHz): δ 5.2 (dd, 1P, $^{2}J_{PF}$ = 111.1 Hz, $^{2}J_{PF}$ = 109.5 Hz). **HRMS** (ESI) *m/z*: $[M + H]^+$ calcd. for $C_{12}H_{21}F_5O_3P$ 339.1148; found 339.1143.

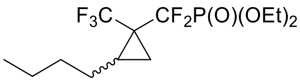

**7b**

Diethyl (2-butyl-1-(trifluoromethyl)cyclopropyl)difluoromethylphosphonate (**7b**) was prepared from 0.3 g (0.1 mmol) of **5**, 0.019 g (0.01 mmol) of CuI, 0.19 mL (0.15 mmol) of 1-hexene and 5 mL of dry toluene. The reaction mixture was refluxed for 3.5 h to give compound **7b** in 46% yield as yellow oil. Mixture of isomers in 1:1.3 ratio. *Major isomer:* **$^{1}$H NMR** ($CDCl_3$, 400 MHz): δ 4.29-4.15 (m, 4H), 2.31-1.48 (m, 2H), 1.47-1.21 (m, 12H), 1.21-1.11 (m, 1H), 0.84 (t, 3H, $^{3}J_{HH}$ = 8.0 Hz); **$^{13}$C NMR** ($CDCl_3$, 100 MHz): δ = 124.3 (qd, $^{1}J_{CF}$ = 277.0 Hz, $^{1}J_{CP}$ = 6.0 Hz), 118.5 (td, $^{1}J_{CF}$ = 263.0 Hz, $^{1}J_{CP}$ = 218.0 Hz), 65.1-64.8 (m), 32.0, 31.6, 31.1-30.5 (m), 26.3 (t, $^{2}J_{CF}$ = 4.0 Hz), 23.7 (q, $^{3}J_{CH}$ = 2.0 Hz), 22.4 (t, $^{3}J_{CH}$ = 3.0 Hz), 16.3 (dd, $^{3}J_{CP}$ = 6.0 Hz, $^{3}J_{CP}$ = 4.0 Hz), 14.25; **$^{19}$F NMR** ($CDCl_3$, 376 MHz): δ -57.7 (t, 3F, $^{4}J_{FF}$ = 7.5 Hz), -106.8 (ddq, 1F, $^{2}J_{FF}$ = 312.1 Hz, $^{2}J_{FP}$ = 109.0 Hz, $^{4}J_{FF}$ = 7.5 Hz) -110.2 (ddq, 1F, $^{2}J_{FF}$ = 312.1 Hz, $^{2}J_{FP}$ = 109.0 Hz, $^{4}J_{FF}$ = 7.5 Hz); **$^{31}$P {$^{1}$H} NMR** ($CDCl_3$, 161 MHz): δ 5.1 (t, 1P, $^{2}J_{PF}$ = 109.5 Hz). *Minor isomer:* **$^{1}$H NMR** ($CDCl_3$, 400 MHz): δ 4.29-4.15 (m, 4H), 2.31-1.48 (m, 2H), 1.47-1.21 (m, 12H), 1.02 (t, 1H, $^{4}J_{HF}$ = 8.0 Hz), 0.84 (t, 3H, $^{3}J_{HH}$ = 8.0 Hz); **$^{13}$C NMR** ($CDCl_3$, 100 MHz): δ = 124.3 (qd, $^{1}J_{CF}$ = 277.0 Hz, $^{1}J_{CP}$ = 6.0 Hz), 118.5 (td, $^{1}J_{CF}$ = 263.0 Hz, $^{1}J_{CP}$ = 218.0 Hz), 65.1-64.8 (m), 32.0, 31.6, 31.1-30.5 (m), 26.3 (t, $^{2}J_{CF}$ = 4.0 Hz), 23.7 (q, $^{3}J_{CH}$ = 2.0 Hz), 22.4 (t, $^{3}J_{CH}$ = 3.0 Hz), 16.3 (dd, $^{3}J_{CP}$ = 6.0 Hz, $^{3}J_{CP}$ = 4.0 Hz), 14.25; **$^{19}$F NMR** ($CDCl_3$, 376 MHz): δ -64.7 (t, 3F, $^{4}J_{FF}$ = 7.5 Hz), -102.2 (ddq, 1F, $^{2}J_{FF}$ = 312.1 Hz, $^{2}J_{FP}$ = 109.0 Hz, $^{4}J_{FF}$ = 7.5 Hz) -104.1 (ddq, 1F, $^{2}J_{FF}$ = 312.1 Hz, $^{2}J_{FP}$ = 109.0 Hz, $^{4}J_{FF}$ = 7.5 Hz); **$^{31}$P {$^{1}$H} NMR** ($CDCl_3$, 161 MHz): δ 5.3 (dd, 1P, $^{2}J_{PF}$ = 111.1 Hz, $^{2}J_{PF}$ = 109.5 Hz). **HRMS** (ESI) *m/z*: $[M + H]^+$ calcd. for $C_{13}H_{23}F_5O_3P$ 353.1304; found 353.1298.

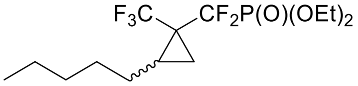


**7c**

Diethyl (2-pentyl-1-(trifluoromethyl)cyclopropyl)difluoromethylphosphonate (**7c**) was prepared from 0.5 g (0.2 mmol) of **5**, 0.032 g (0.02 mmol) of CuI, 0.33 mL (0.25 mmol) of 1-

hepten and 10 mL of dry toluene. The reaction mixture was refluxed for 2.5 h to give compound **7c** in 42% yield as yellow oil. Mixture of isomers in 1:1.1 ratio. *Major isomer:* **$^{1}$H NMR** ($CDCl_3$, 400 MHz): δ 4.29-4.15 (m, 4H), 1.95-1.52 (m, 2H), 1.47-1.19 (m, 14H), 1.13 (t, 1H, $^{4}J_{HF}$ = 8.0 Hz), 0.82 (t, 3H, $^{3}J_{HH}$ = 8.0 Hz); **$^{13}$C NMR** ($CDCl_3$, 100 MHz): δ = 124.7 (qd, $^{1}J_{CF}$ = 274.0 Hz, $^{1}J_{CP}$ = 5.0 Hz), 118.5 (td, $^{1}J_{CF}$ = 263.0 Hz, $^{1}J_{CP}$ = 218.0 Hz), 65.2-64.8 (m), 31.5 (d, $^{2}J_{CH}$ = 3.0 Hz), 31.0-30.5 (m), 29.5, 29.1, 26.6 (q, $^{2}J_{CF}$ = 2.0 Hz), 23.7 (q, $^{3}J_{CH}$ = 2.0 Hz), 22.6 (d, $^{3}J_{CH}$ = 4.0 Hz), 22.5 (t, $^{3}J_{CH}$ = 4.0 Hz), 16.3 (dd, $^{3}J_{CP}$ = 6.0 Hz, $^{3}J_{CP}$ = 4.0 Hz), 14.1; **$^{19}$F NMR** ($CDCl_3$, 376 MHz): δ -57.8 (t, 3F, $^{4}J_{FF}$ = 7.5 Hz), -106.8, (ddq, 1F, $^{2}J_{FF}$ = 312.1 Hz, $^{2}J_{FP}$ = 109.0 Hz, $^{4}J_{FF}$ = 7.5 Hz) -110.2 (ddq, 1F, $^{2}J_{FF}$ = 312.1 Hz, $^{2}J_{FP}$ = 109.0 Hz, $^{4}J_{FF}$ = 7.5 Hz); **$^{31}$P {$^{1}$H} NMR** ($CDCl_3$, 161 MHz): δ 5.1 (t, 1P, $^{2}J_{PF}$ = 109.5 Hz). *Minor isomer:* **$^{1}$H NMR** ($CDCl_3$, 400 MHz): δ 4.29-4.15 (m, 4H), 1.95-1.52 (m, 2H), 1.47-1.19 (m, 14H), 1.02 (t, 1H, $^{4}J_{HF}$ = 8.0 Hz), 0.82 (t, 3H, $^{3}J_{HH}$ = 8.0 Hz); **$^{13}$C NMR** ($CDCl_3$, 100 MHz): δ = 124.3 (qd, $^{1}J_{CF}$ = 273.0 Hz, $^{1}J_{CP}$ = 6.0 Hz), 117.7 (td, $^{1}J_{CF}$ = 260.0 Hz, $^{1}J_{CP}$ = 218.0 Hz), 65.2-64.8 (m), 31.5 (d, $^{2}J_{CH}$ = 3.0 Hz), 31.0-30.5 (m), 29.5, 29.1, 26.6 (q, $^{2}J_{CF}$ = 2.0 Hz), 23.7 (q, $^{3}J_{CH}$ = 2.0 Hz), 22.6 (d, $^{3}J_{CH}$ = 4.0 Hz), 22.5 (t, $^{3}J_{CH}$ = 4.0 Hz), 16.3 (dd, $^{3}J_{CP}$ = 6.0 Hz, $^{3}J_{CP}$ = 4.0 Hz), 14.1; **$^{19}$F NMR** ($CDCl_3$, 376 MHz): δ -64.8 (t, 3F, $^{4}J_{FF}$ = 7.5 Hz), -102.1 (ddq, 1F, $^{2}J_{FF}$ = 312.1 Hz, $^{2}J_{FP}$ = 109.0 Hz, $^{4}J_{FF}$ = 7.5 Hz) -104.1 (ddq, 1F, $^{2}J_{FF}$ = 312.1 Hz, $^{2}J_{FP}$ = 109.0 Hz, $^{4}J_{FF}$ = 7.5 Hz); **$^{31}$P {$^{1}$H} NMR** ($CDCl_3$, 161 MHz): δ 5.2 (dd, 1P, $^{2}J_{PF}$ = 111.1 Hz, $^{2}J_{PF}$ = 109.5 Hz). **HRMS** (ESI) *m/z*: $[M + H]^+$ calcd. for $C_{14}H_{25}F_5O_3P$ 367.1461; found 367.1455.

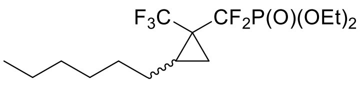


**7d**

Diethyl (2-hexyl-1-(trifluoromethyl)cyclopropyl)difluoromethylphosphonate (**7d**) was prepared from 0.2 g (0.07 mmol) of **5**, 0.012 g (0.007 mmol) of CuI, 0.16 mL (0.1 mmol) of octene-1 and 4 mL of dry toluene. The reaction mixture was refluxed for 2.5 h to give compound **7d** in 34% yield as yellow oil. Mixture of isomers in 1:2.2 ratio. *Major isomer:* **$^{1}$H**

**NMR** ($CDCl_3$, 400 MHz): δ 4.29-4.14 (m, 4H), 1.96-1.47 (m, 2H), 1.43-1.28 (m, 10H), 1.28-1.17 (m, 6H), 1.15-1.01 (m, 1H), 0.81 (t, 3H, $^3J_{HH}$ = 4.0 Hz); **$^{13}$C NMR** ($CDCl_3$, 100 MHz): δ = 124.7 (qd, $^1J_{CF}$ = 275.0 Hz, $^1J_{CP}$ = 5.0 Hz), 117.7 (td, $^1J_{CF}$ = 266.0 Hz, $^1J_{CP}$ = 219.0 Hz), 64.9 (m), 31.8 (d, $^2J_{CH}$ = 6.0 Hz), 29.8, 29.4, 29.0 (d, $^2J_{CH}$ = 5.0 Hz), 26.7, 23.7, 22.7, 22.5 (t, $^3J_{CH}$ = 4.0 Hz), 16.4 (dd, $^3J_{CP}$ = 6.0 Hz, $^3J_{CP}$ = 4.0 Hz), 14.1; **$^{19}$F NMR** ($CDCl_3$, 376 MHz): δ -57.7 (t, 3F, $^4J_{FF}$ = 7.5 Hz), -106.8 (ddq, 1F, $^2J_{FF}$ = 312.1 Hz, $^2J_{FP}$ = 109.0 Hz, $^4J_{FF}$ = 7.5 Hz) -110.2 (ddq, 1F, $^2J_{FF}$ = 312.1 Hz, $^2J_{FP}$ = 109.0 Hz, $^4J_{FF}$ = 7.5 Hz); **$^{31}$P {$^1$H} NMR** ($CDCl_3$, 161 MHz): δ 5.2 (t, 1P, $^2J_{PF}$ = 109.5 Hz). *Minor isomer:* **$^1$H NMR** ($CDCl_3$, 400 MHz): δ 4.29-4.14 (m, 4H), 1.96-1.47 (m, 2H), 1.43-1.28 (m, 10H), 1.28-1.17 (m, 6H), 1.15-1.01 (m, 1H), 0.81 (t, 3H, $^3J_{HH}$ = 4.0 Hz); **$^{13}$C NMR** ($CDCl_3$, 100 MHz): 124.7 (qd, $^1J_{CF}$ = 275.0 Hz, $^1J_{CP}$ = 5.0 Hz), 117.7 (td, $^1J_{CF}$ = 266.0 Hz, $^1J_{CP}$ = 219.0 Hz), 64.9 (m), 31.8 (d, $^2J_{CH}$ = 6.0 Hz), 29.8, 29.4, 29.0 (d, $^2J_{CH}$ = 5.0 Hz), 26.7, 23.7, 22.7, 22.5 (t, $^3J_{CH}$ = 4.0 Hz), 16.4 (dd, $^3J_{CP}$ = 6.0 Hz, $^3J_{CP}$ = 4.0 Hz), 14.1; **$^{19}$F NMR** ($CDCl_3$, 376 MHz): δ -64.7 (t, 3F, $^4J_{FF}$ = 7.5 Hz), -102.1 (ddq, 1F, $^2J_{FF}$ = 312.1 Hz, $^2J_{FP}$ = 109.0 Hz, $^4J_{FF}$ = 7.5 Hz) -104.1 (ddq, 1F, $^2J_{FF}$ = 312.1 Hz, $^2J_{FP}$ = 109.0 Hz, $^4J_{FF}$ = 7.5 Hz); **$^{31}$P {$^1$H} NMR** ($CDCl_3$, 161 MHz): δ 5.3 (dd, 1P, $^2J_{PF}$ = 109.5 Hz, $^2J_{PF}$ = 107.9 Hz). **HRMS** (ESI) *m/z*: $[M + H]^+$ calcd. for $C_{15}H_{27}F_5O_3P$ 381.1617; found 381.1613.

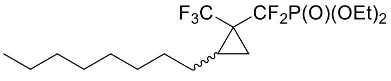


**7e**

Diethyl (2-octyl-1-(trifluoromethyl)cyclopropyl)difluoromethylphosphonate (**7e**) was prepared from 0.3 g (0.1 mmol) of **5**, 0.019 g (0.01 mmol) of CuI, 0.3 mL (0.15 mmol) of 1-decene and 5 mL of dry toluene. The reaction mixture was refluxed for 5 h to give compound **7e** in 41% yield as yellow oil. Mixture of isomers in 1:1.2 ratio. *Major isomer:* **$^1$H NMR** ($CDCl_3$, 400 MHz): δ 4.30-4.13 (m, 4H), 1.82-1.54 (m, 1H), 1.52-1.01 (m, 22H), 0.80 (t, 3H, $^3J_{HH}$ = 8.0 Hz); **$^{13}$C NMR** ($CDCl_3$, 100 MHz): δ = 124.3 (qd, $^1J_{CF}$ = 272.0 Hz, $^1J_{CP}$ = 6.0 Hz), 117.6 (td, $^1J_{CF}$ = 261.0 Hz, $^1J_{CP}$ = 218.0 Hz), 65.0-64.8 (m), 31.9, 31.1-30.5 (m), 29.8, 29.5 (d,

$^2J_{CH}$ = 7.0 Hz), 29.4 (d, $^3J_{CH}$ = 4.0 Hz), 29.3 (d, $^3J_{CH}$ = 4.0 Hz), 26.7 (dd, $^3J_{CH}$ = 6.0 Hz, $^3J_{CH}$ = 3.0 Hz), 23.7, 22.7, 22.5 (t, $^3J_{CH}$ = 4.0 Hz), 16.3 (dd, $^3J_{CP}$ = 7.0 Hz, $^3J_{CP}$ = 4.0 Hz), 14.1; **$^{19}$F NMR** ($CDCl_3$, 376 MHz): δ -57.8 (t, 3F, $^4J_{FF}$ = 7.5 Hz), -106.8 (ddq, 1F, $^2J_{FF}$ = 312.1 Hz, $^2J_{FP}$ = 109.0 Hz, $^4J_{FF}$ = 7.5 Hz) -110.1 (ddq, 1F, $^2J_{FF}$ = 312.1 Hz, $^2J_{FP}$ = 109.0 Hz, $^4J_{FF}$ = 7.5 Hz); **$^{31}$P {$^1$H} NMR** ($CDCl_3$, 161 MHz): δ 5.1 (t, 1P, $^2J_{PF}$ = 109.5 Hz). *Minor isomer:* **$^1$H NMR** ($CDCl_3$, 400 MHz): δ 4.30-4.13 (m, 4H), 1.82-1.54 (m, 1H), 1.52-1.01 (m, 22H), 0.80 (t, 3H, $^3J_{HH}$ = 8.0 Hz); **$^{13}$C NMR** ($CDCl_3$, 100 MHz): δ = 124.3 (qd, $^1J_{CF}$ = 272.0 Hz, $^1J_{CP}$ = 6.0 Hz), 117.6 (td, $^1J_{CF}$ = 261.0 Hz, $^1J_{CP}$ = 218.0 Hz), 65.0-64.8 (m), 31.9, 31.1-30.5 (m), 29.8, 29.5 (d, $^2J_{CH}$ = 7.0 Hz), 29.4 (d, $^3J_{CH}$ = 4.0 Hz), 29.3 (d, $^3J_{CH}$ = 4.0 Hz), 26.7 (dd, $^3J_{CH}$ = 6.0 Hz, $^3J_{CH}$ = 3.0 Hz), 23.7, 22.7, 22.5 (t, $^3J_{CH}$ = 4.0 Hz), 16.3 (dd, $^3J_{CP}$ = 7.0 Hz, $^3J_{CP}$ = 4.0 Hz), 14.1; **$^{19}$F NMR** ($CDCl_3$, 376 MHz): δ -64.7 (t, 3F, $^4J_{FF}$ = 7.5 Hz), -102.1 (ddq, 1F, $^2J_{FF}$ = 312.1 Hz, $^2J_{FP}$ = 109.0 Hz, $^4J_{FF}$ = 7.5 Hz) -103.9 (ddq, 1F, $^2J_{FF}$ = 312.1 Hz, $^2J_{FP}$ = 109.0 Hz, $^4J_{FF}$ = 7.5 Hz); **$^{31}$P {$^1$H} NMR** ($CDCl_3$, 161 MHz): δ 5.3 (dd, 1P, $^2J_{PF}$ = 111.1 Hz, $^2J_{PF}$ = 109.5 Hz). **HRMS** (ESI) *m/z*: $[M + H]^+$ calcd. for $C_{17}H_{31}F_5O_3P$ 409.1930; found 409.1924.

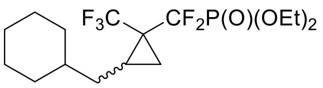


**7f**

Diethyl (2-(cyclohexylmethyl)-1-(trifluoromethyl)cyclopropyl)difluoromethylphosphonate (**7f**) was prepared from 0.5 g (0.2 mmol) of **5**, 0.032 g (0.02 mmol) of CuI, 0.4 mL (0.25 mmol) of allylcyclohexane and 10 mL of dry toluene. The reaction mixture was refluxed for 5 h to give compound **7f** in 28% yield as yellow oil. Mixture of isomers in 1:1.3 ratio. *Major isomer:* **$^1$H NMR** ($CDCl_3$, 400 MHz): δ 4.30-4.13 (m, 4H), 2.10-1.69 (m, 2H), 1.65-1.06 (m, 17H), 1.05-0.97 (m, 1H), 0.92-0.81 (m, 2H); **$^{13}$C NMR** ($CDCl_3$, 100 MHz): δ = 124.7 (qd, $^1J_{CF}$ = 276.0 Hz, $^1J_{CP}$ = 4.0 Hz), 118.5 (td, $^1J_{CF}$ = 264.0 Hz, $^1J_{CP}$ = 218.0 Hz), 65.0-64.8 (m), 38.5, 38.1, 33.4, 33.0, 30.9-29.7 (m), 26.6 (d, $^3J_{CH}$ = 2.0 Hz), 26.3 (t, $^3J_{CH}$ = 3.0 Hz), 24.1, 21.9, 20.6

(t, $^{3}J_{CH}$ = 3.0 Hz), 16.4 (dd, $^{3}J_{CP}$ = 6.0 Hz, $^{3}J_{CP}$ = 4.0 Hz); **$^{19}$F NMR** ($CDCl_3$, 376 MHz): δ -57.7 (t, 3F, $^{4}J_{FF}$ = 7.5 Hz), -106.7 (ddq, 1F, $^{2}J_{FF}$ = 312.1 Hz, $^{2}J_{FP}$ = 109.0 Hz, $^{4}J_{FF}$ = 7.5 Hz) -109.9 (ddq, 1F, $^{2}J_{FF}$ = 312.1 Hz, $^{2}J_{FP}$ = 109.0 Hz, $^{4}J_{FF}$ = 7.5 Hz); **$^{31}$P {$^{1}$H} NMR** ($CDCl_3$, 161 MHz): δ 5.1 (t, 1P, $^{2}J_{PF}$ = 109.5 Hz). *Minor isomer:* **$^{1}$H NMR** ($CDCl_3$, 400 MHz): δ 4.30-4.13 (m, 4H), 2.10-1.69 (m, 2H), 1.65-1.06 (m, 17H), 1.05-0.97 (m, 1H), 0.92-0.81 (m, 2H); **$^{13}$C NMR** ($CDCl_3$, 100 MHz): δ = 124.4 (qd, $^{1}J_{CF}$ = 273.0 Hz, $^{1}J_{CP}$ = 6.0 Hz), 117.7 (td, $^{1}J_{CF}$ = 266.0 Hz, $^{1}J_{CP}$ = 219.0 Hz), 65.9-65.7 (m), 38.5, 38.1, 33.4, 33.0, 30.9-29.7 (m), 26.6 (d, $^{3}J_{CH}$ = 2.0 Hz), 26.3 (t, $^{3}J_{CH}$ = 3.0 Hz), 24.1, 21.9, 20.6 (t, $^{3}J_{CH}$ = 3.0 Hz), 16.4 (dd, $^{3}J_{CP}$ = 6.0 Hz, $^{3}J_{CP}$ = 4.0 Hz); **$^{19}$F NMR** ($CDCl_3$, 376 MHz): δ -64.6 (t, 3F, $^{4}J_{FF}$ = 7.5 Hz), -101.9 (ddq, 1F, $^{2}J_{FF}$ = 312.1 Hz, $^{2}J_{FP}$ = 109.0 Hz, $^{4}J_{FF}$ = 7.5 Hz), -104.2 (ddq, 1F, $^{2}J_{FF}$ = 312.1 Hz, $^{2}J_{FP}$ = 109.0 Hz, $^{4}J_{FF}$ = 7.5 Hz); **$^{31}$P {$^{1}$H} NMR** ($CDCl_3$, 161 MHz): δ 5.3 (dd, 1P, $^{2}J_{PF}$ = 111.1Hz, $^{2}J_{PF}$ = 109.5 Hz). **HRMS** (ESI) *m/z*: $[M + H]^+$ calcd. for $C_{16}H_{27}F_5O_3P$ 393.1617; found 393.1613.

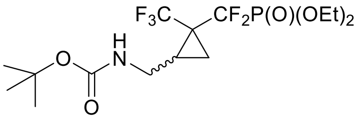


**7g**

*tert*-Butyl ((2-((diethoxyphosphoryl)difluoromethyl)-2-(trifluoromethyl)cyclopropyl)methyl)carbamate (**7g**) was prepared from 0.5 g (0.2 mmol) of **5**, 0.032 g (0.02 mmol) of CuI, 0.4 g (0.25 mmol) of *tert*-butyl-*N*-allylcarbamate and 10 mL of dry toluene. The reaction mixture was refluxed for 5 h to give compound **7g** in 53% yield as yellow oil. Mixture of isomers in 1:6 ratio. *Major isomer:* **$^{1}$H NMR** ($CDCl_3$, 400 MHz): δ 4.27-4.16 (m, 8H), 1.97 (d, 1H, $^{4}J_{HF}$ = 4.0 Hz), 1.38 (s, 3H), 1.34-1.30 (m, 12H); **$^{13}$C NMR** ($CDCl_3$, 100 MHz): δ = 151.9, 120.6 (td, $^{1}J_{CF}$ = 282.0 Hz, $^{1}J_{CP}$ = 11.0 Hz), 114.3 (td, $^{1}J_{CF}$ = 265.0 Hz, $^{1}J_{CP}$ = 215.0 Hz), 67.6-66.4 (m), 64.4 (dd, $^{2}J_{CH}$ = 13.0, $^{2}J_{CH}$ = 7.0 Hz), 42.8, 40.6, 36.5-36.4 (m), 28.7, 27.4, 27.2, 22.9, 15.3 (dd, $^{3}J_{CP}$ = 5.0, $^{3}J_{CP}$ = 2.0 Hz); **$^{19}$F NMR** ($CDCl_3$, 376 MHz): δ -71.4 (q, 3F, $^{4}J_{FF}$ = 11.3 Hz), -116.5 (ddtd, 1F, $^{2}J_{FF}$ = 319.6 Hz, $^{2}J_{FP}$ = 90.2 Hz, 11.3 Hz, $^{4}J_{FF}$

= 7.6 Hz), -123.6 (ddtd, 1F, $^2J_{FF}$ = 319.6 Hz, $^2J_{FP}$ = 90.2 Hz, 11.3 Hz, $^4J_{FF}$ = 7.6 Hz); **$^{31}$P {$^1$H} NMR** ($CDCl_3$, 161 MHz): δ 3.6 (ddq, 1P, $^2J_{PF}$ = 103.0 Hz, $^2J_{PF}$ = 91.8 Hz, $^4J_{PF}$ = 3.2 Hz). *Minor isomer:* **$^1$H NMR** ($CDCl_3$, 400 MHz): δ 4.27-4.16 (m, 8H), 1.98 (d, 1H, $^4J_{HF}$ = 4.0 Hz), 1.38 (s, 3H), 1.34-1.30 (m, 12H); **$^{13}$C NMR** ($CDCl_3$, 100 MHz): δ = 151.9, 120.6 (td, $^1J_{CF}$ = 282.0 Hz, $^1J_{CP}$ = 11.0 Hz), 114.3 (td, $^1J_{CF}$ = 265.0 Hz, $^1J_{CP}$ = 215.0 Hz), 67.6-66.4 (m), 64.4 (dd, $^2J_{CH}$ = 13.0, $^2J_{CH}$ = 7.0 Hz), 42.8, 40.6, 36.5-36.4 (m), 28.7, 27.4, 27.2, 22.9, 15.3 (dd, $^3J_{CP}$ = 5.0, $^3J_{CP}$ = 2.0 Hz); **$^{19}$F NMR** ($CDCl_3$, 376 MHz): δ -64.9 (d, 3F, $^4J_{FF}$ = 3.8 Hz), -98.6 (ddtd, 1F, $^2J_{FF}$ = 319.6 Hz, $^2J_{FP}$ = 90.2 Hz, $^4J_{FF}$ = 11.3 Hz, $^4J_{FH}$ = 3.8 Hz), -106.2 (ddtd, 1F, $^2J_{FF}$ = 319.6 Hz, $^2J_{FP}$ = 90.2 Hz, $^4J_{FF}$ = 11.3 Hz, $^4J_{FH}$ = 3.8 Hz); **$^{31}$P {$^1$H} NMR** ($CDCl_3$, 161 MHz): δ 4.8 (dd, 1P, $^2J_{PF}$ = 111.1 Hz, $^2J_{PF}$ = 103.0 Hz). **HRMS** (ESI) *m/z*: $[M + H]^+$ calcd. for $C_{15}H_{26}F_5NO_5P$ 426.1468; found 426.1464.

## 7. Copies of $^{1}H$ NMR, $^{13}C$ NMR, $^{19}F$ NMR, and $^{31}P$ NMR spectra for the compounds 3, 4, 5, 6a–i and 7a–g

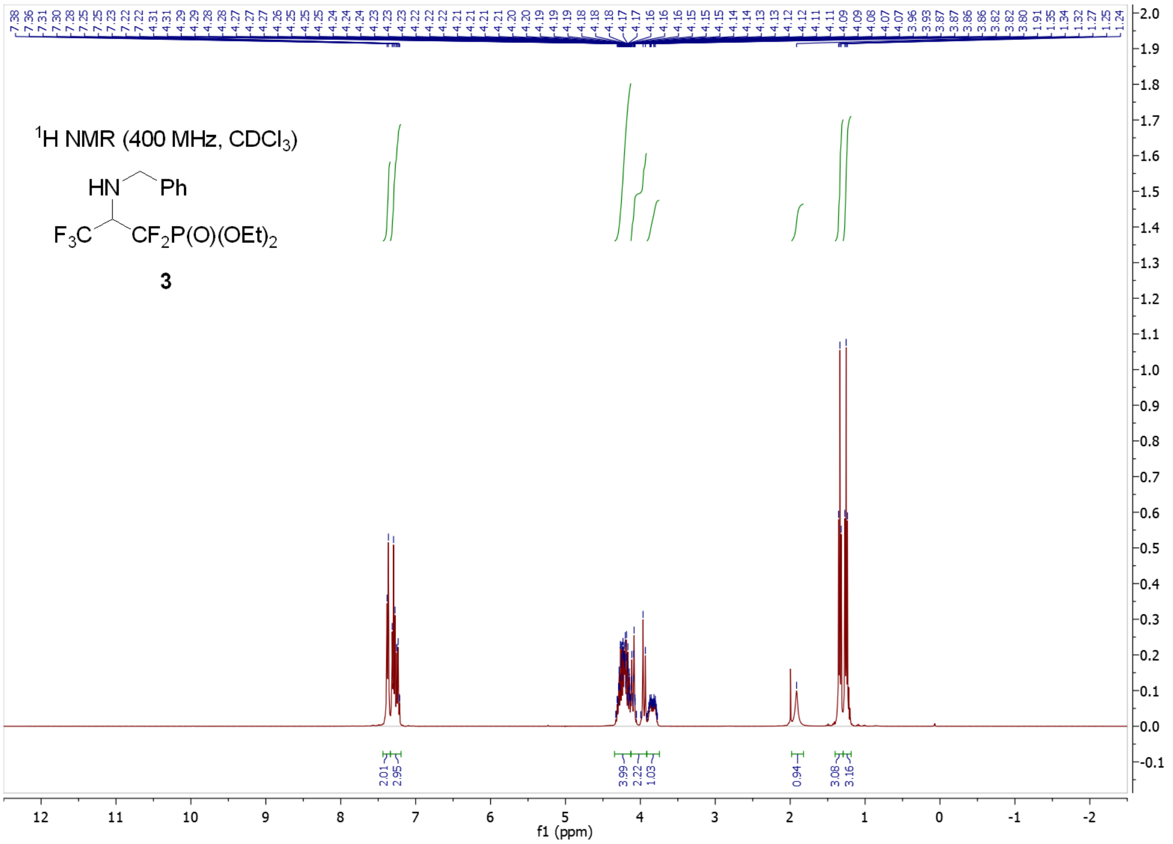

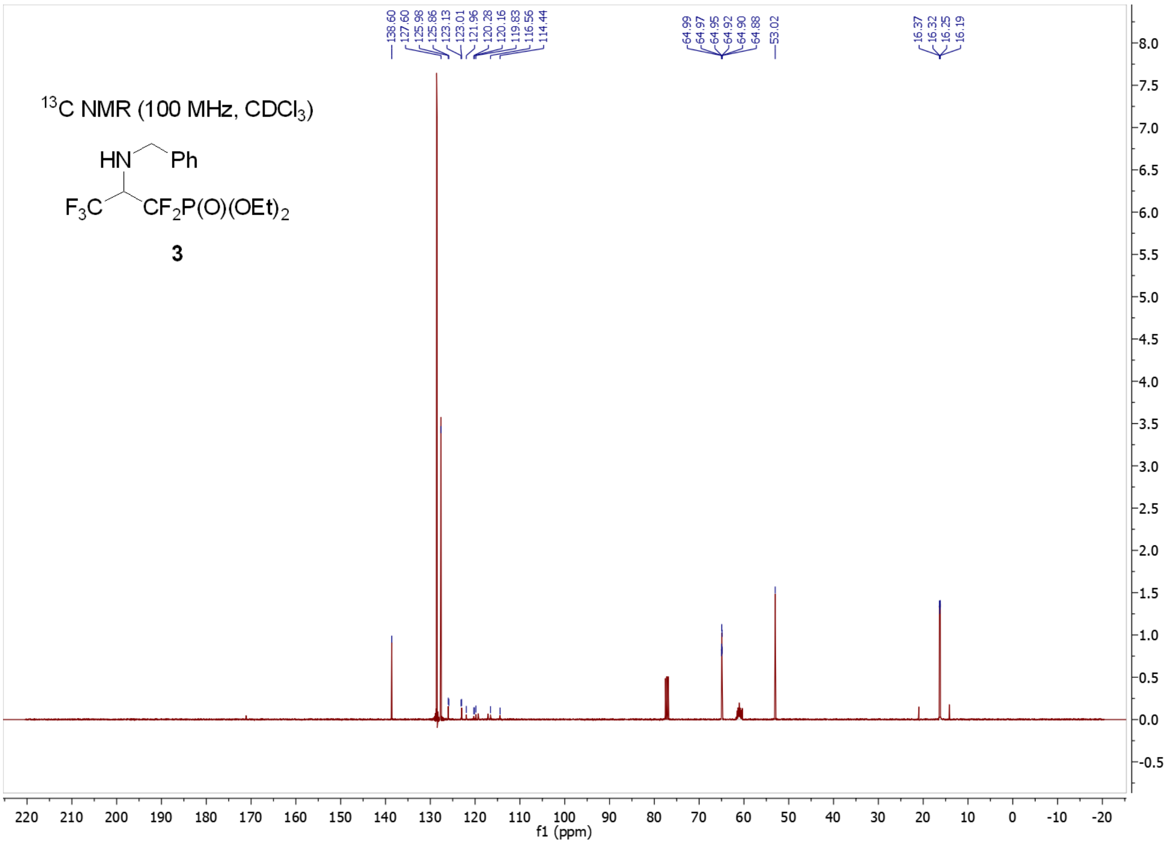

$^{13}$C NMR (100 MHz, CDCl$_3$)
HN Ph
F$_3$C CF$_2$P(O)(OEt)$_2$
3
138.60 127.60 125.98 125.86 123.13 123.01 121.96 120.28 120.16 119.83 116.56 114.44
64.99 64.97 64.95 64.92 64.90 64.88 53.02
16.37 16.32 16.25 16.19
f1 (ppm)


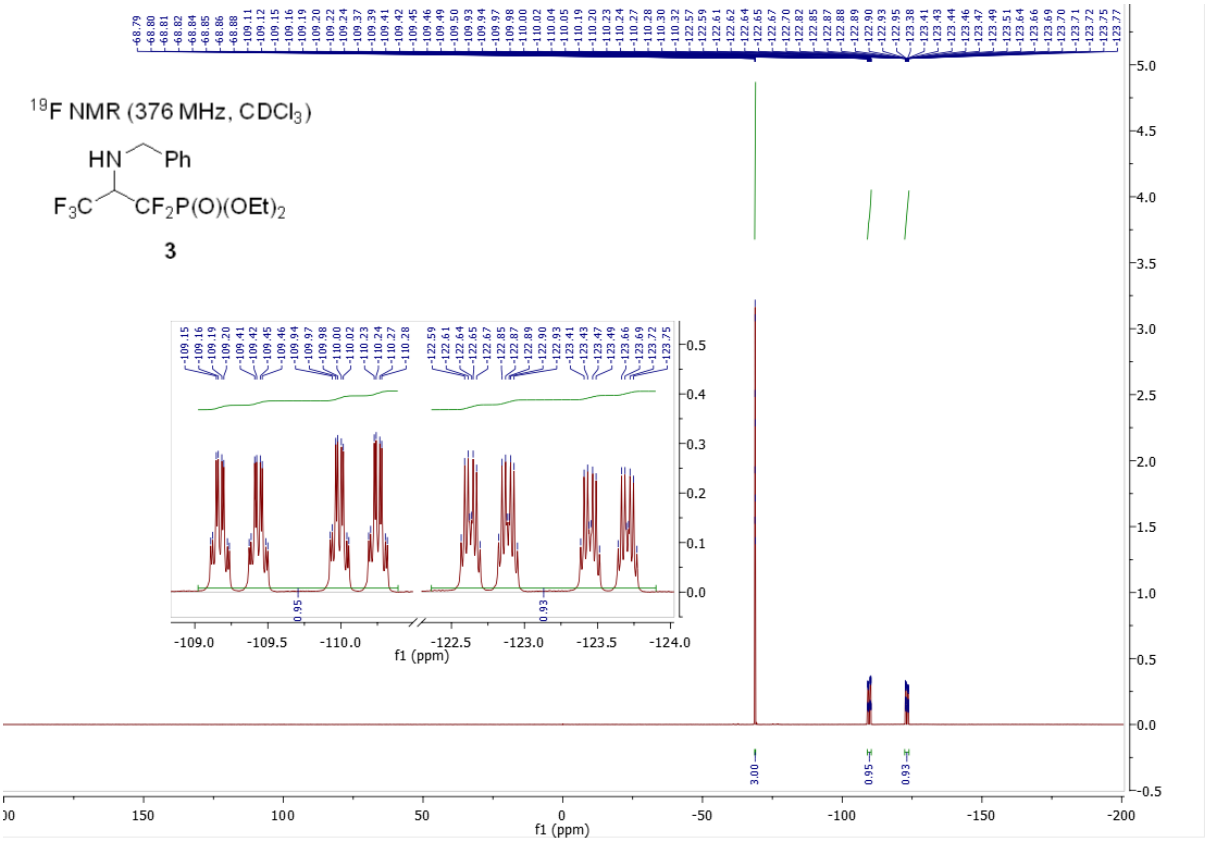

$^{19}$F NMR (376 MHz, CDCl$_3$)
HN Ph
F$_3$C CF$_2$P(O)(OEt)$_2$
3
3.00
0.95
0.93
f1 (ppm)

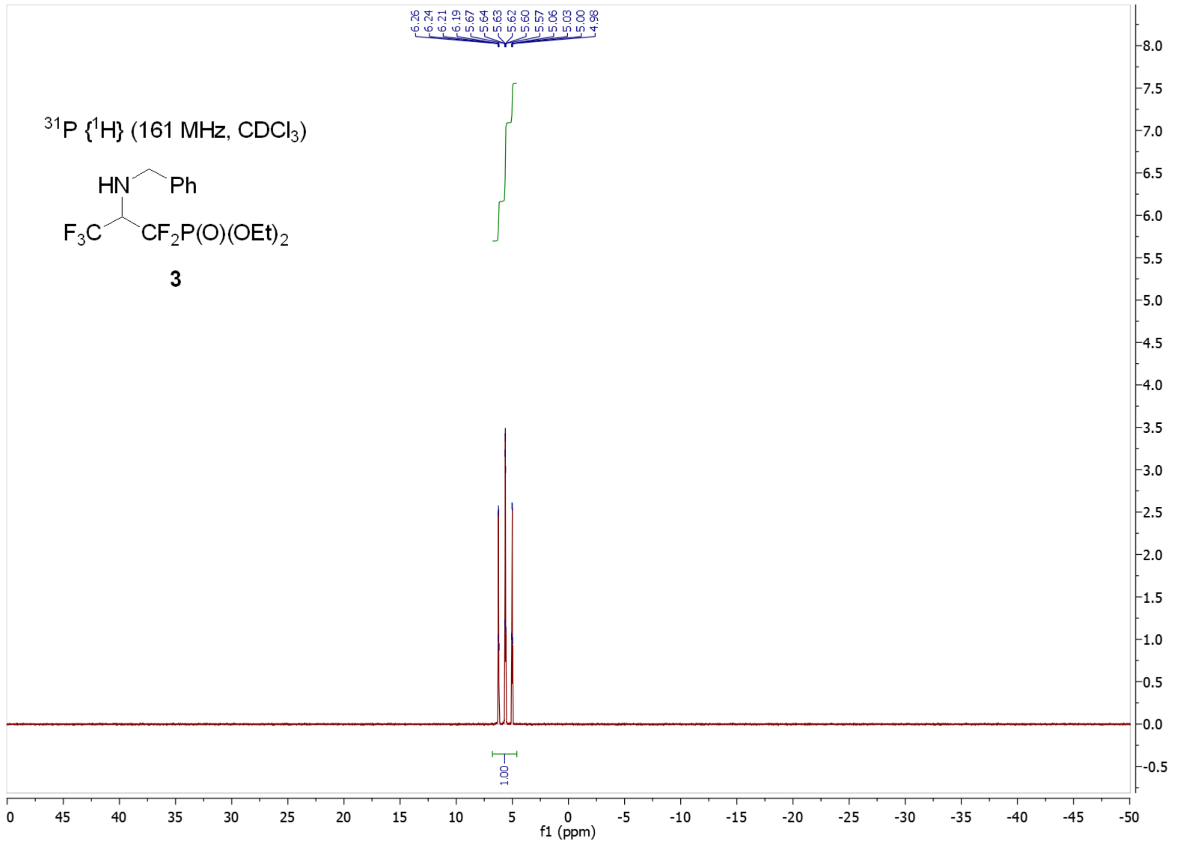

6.26
6.24
6.21
6.19
5.67
5.64
5.63
5.62
5.60
5.57
5.06
5.03
5.00
4.98
31P {1H} (161 MHz, CDCl3)
HN
Ph
F3C
CF2P(O)(OEt)2
3
1.00
f1 (ppm)


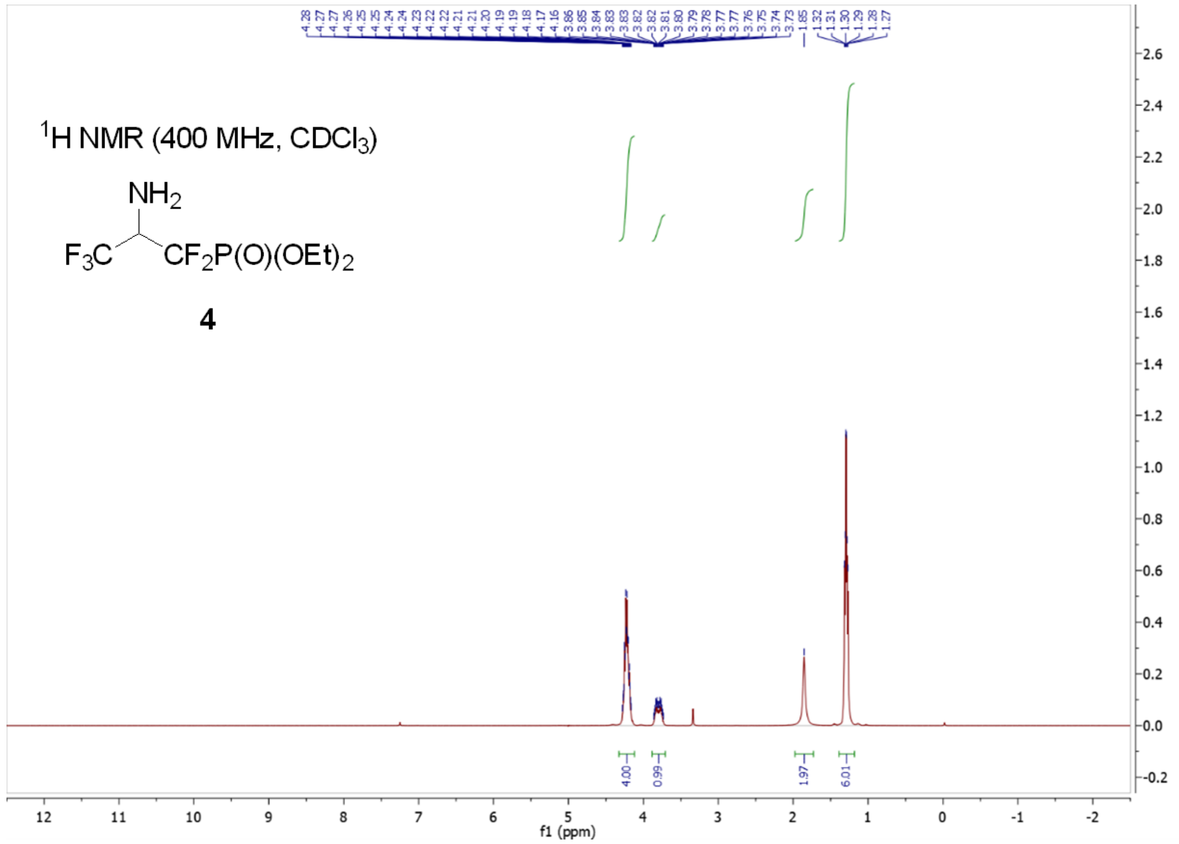

1H NMR (400 MHz, CDCl3)
NH2
F3C
CF2P(O)(OEt)2
4
4.00
0.99
1.97
6.01
f1 (ppm)

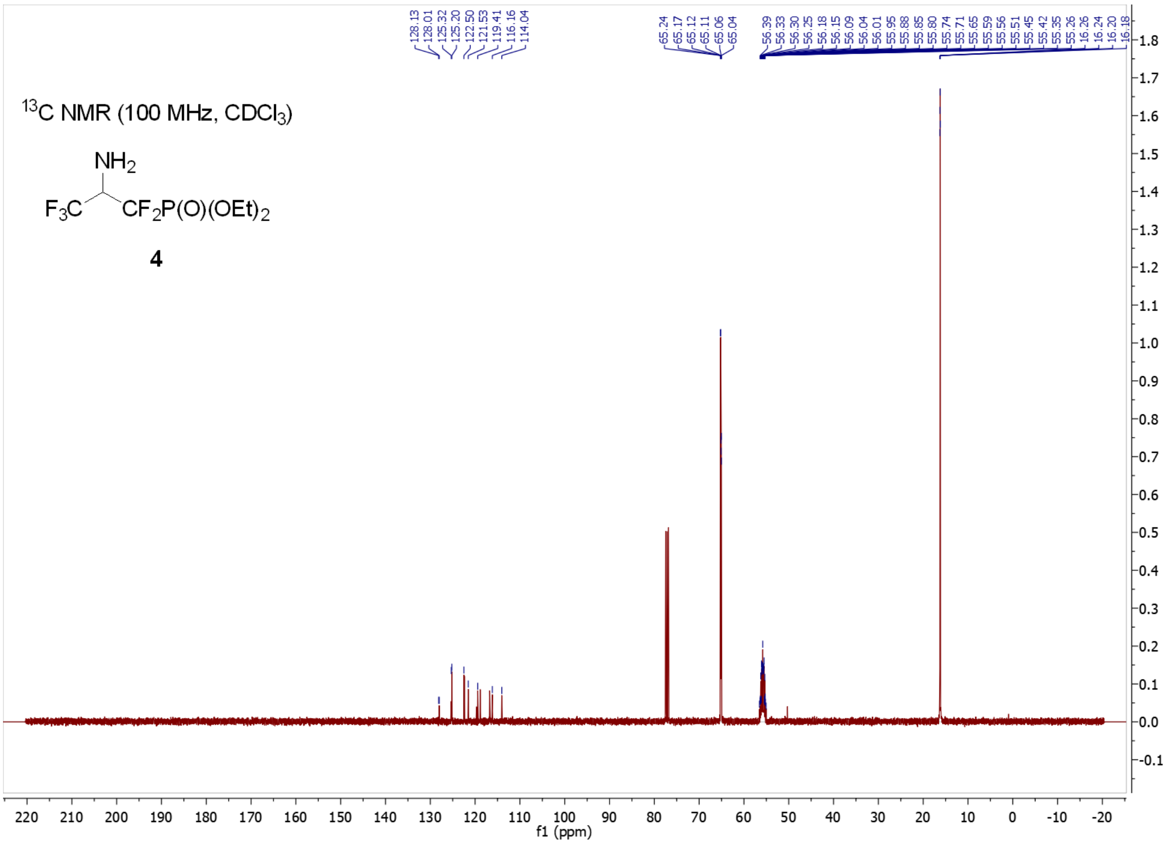

$^{13}$C NMR (100 MHz, $CDCl_3$)
4
f1 (ppm)


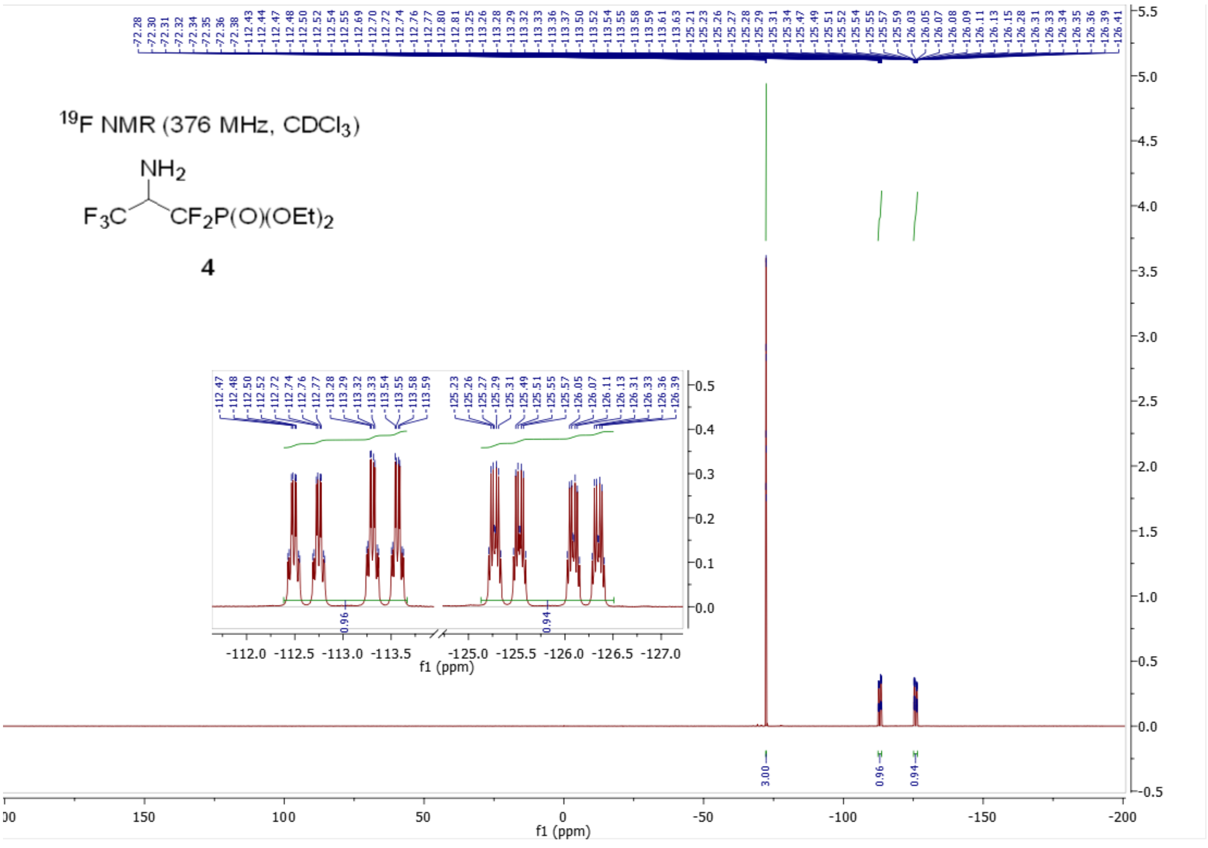

$^{19}$F NMR (376 MHz, $CDCl_3$)
4
f1 (ppm)

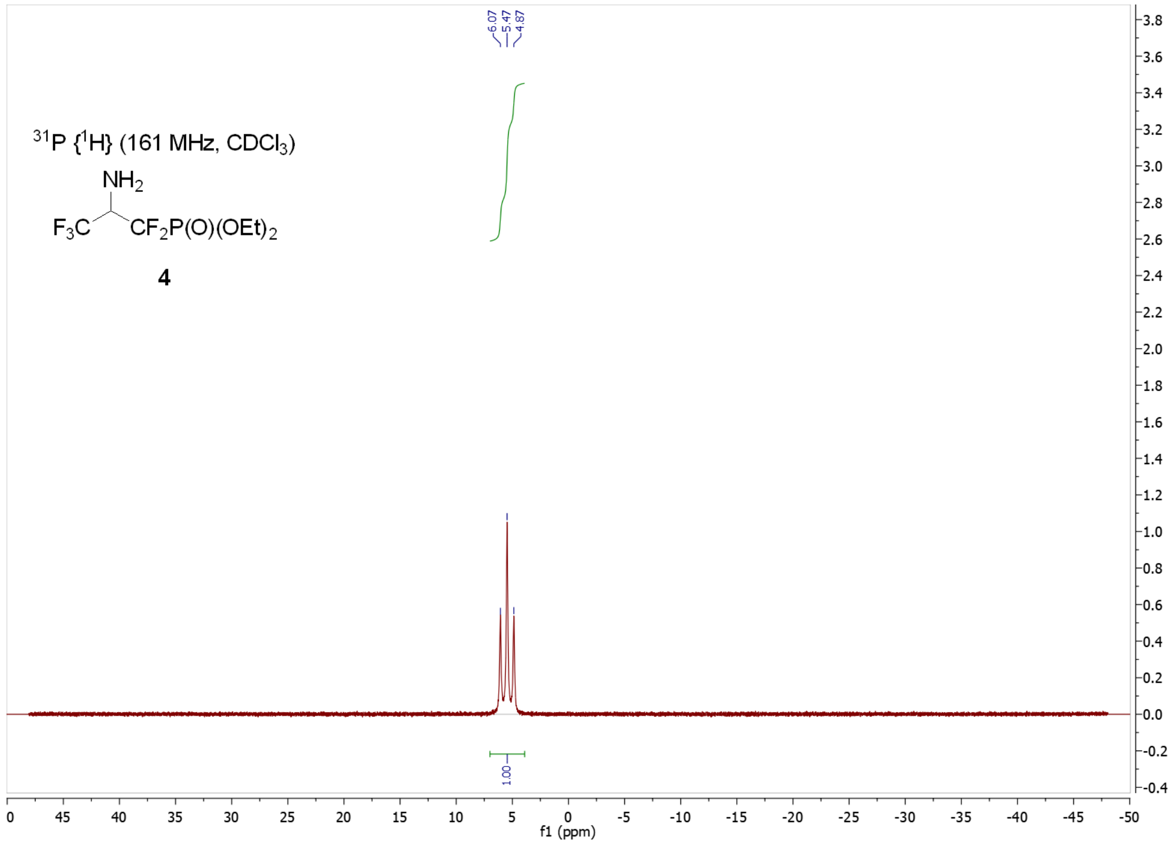

$^{31}P$ {$^1H$} (161 MHz, $CDCl_3$)
NH2
F3C CF2P(O)(OEt)2
4
6.07
5.47
4.87
1.00
f1 (ppm)


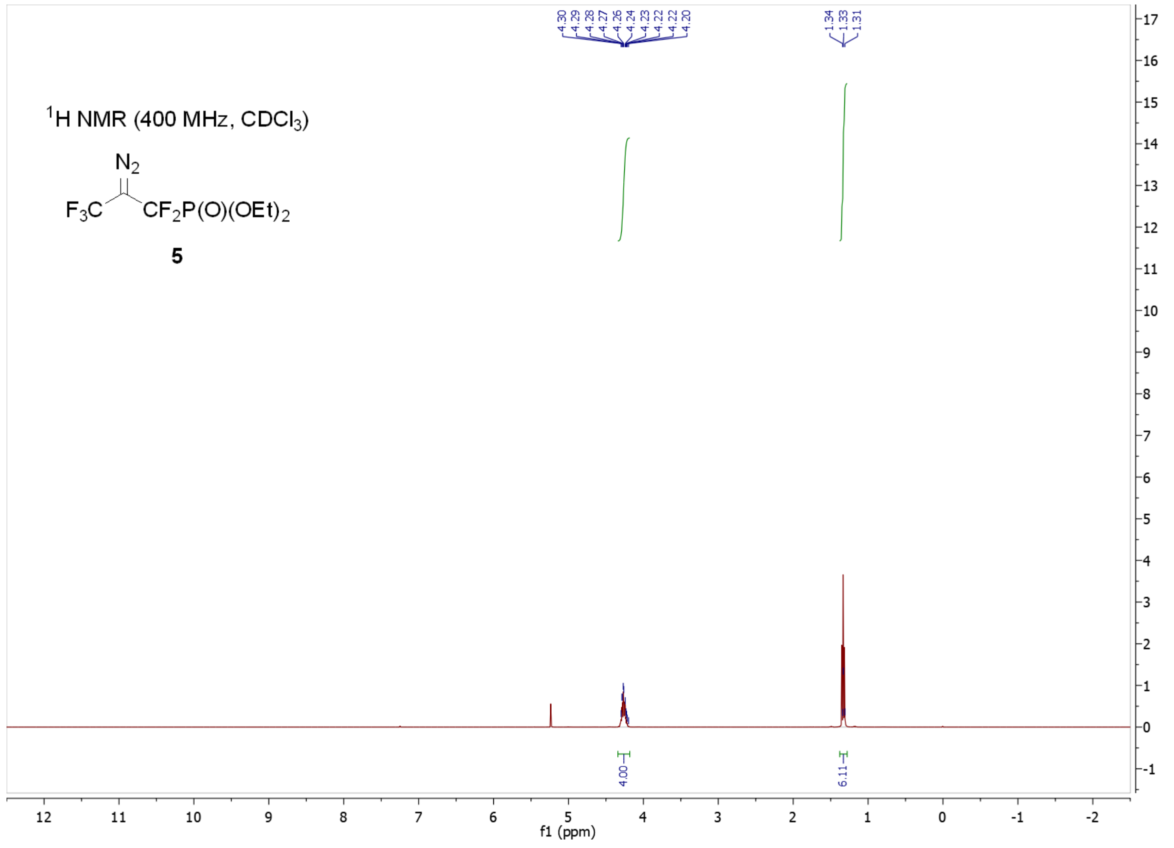

$^1H$ NMR (400 MHz, $CDCl_3$)
N2
F3C CF2P(O)(OEt)2
5
4.30
4.29
4.28
4.27
4.26
4.24
4.23
4.22
4.22
4.20
1.34
1.33
1.31
4.00
6.11
f1 (ppm)

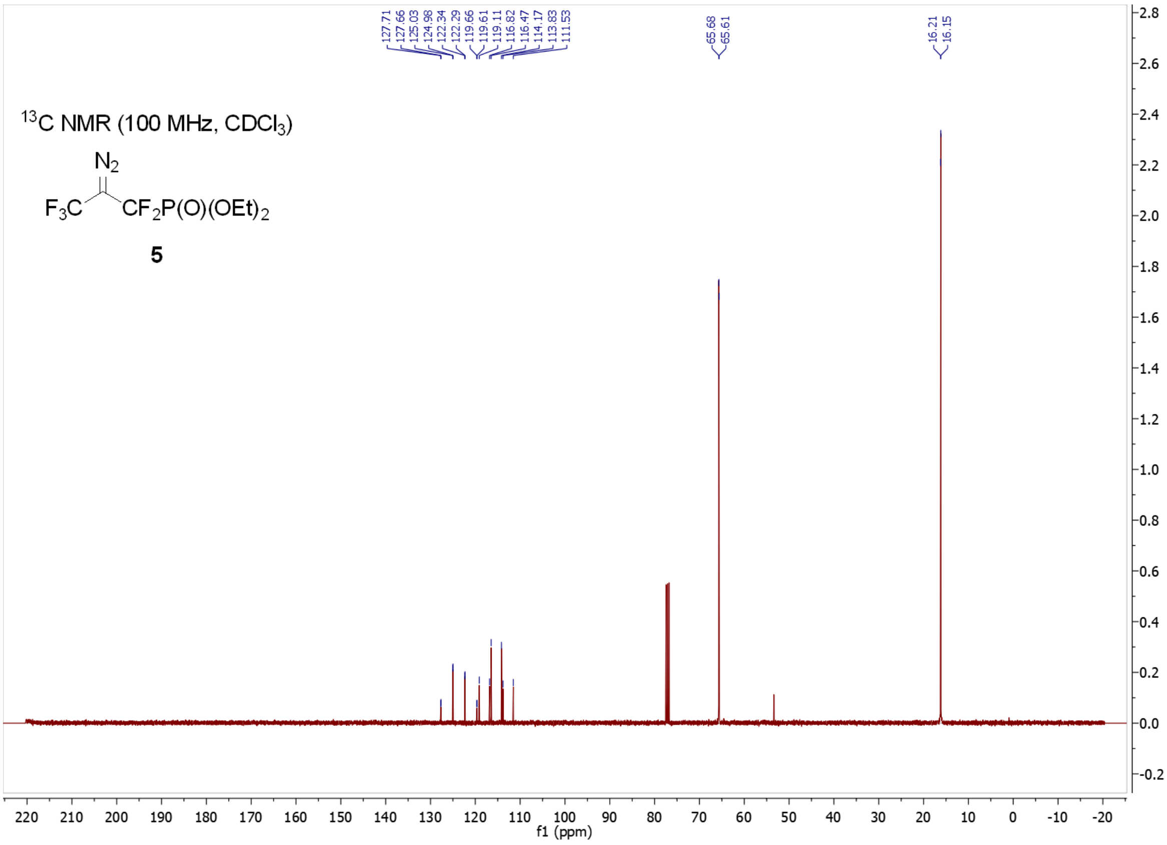

13C NMR (100 MHz, CDCl3)
N2
F3C
CF2P(O)(OEt)2
5
f1 (ppm)


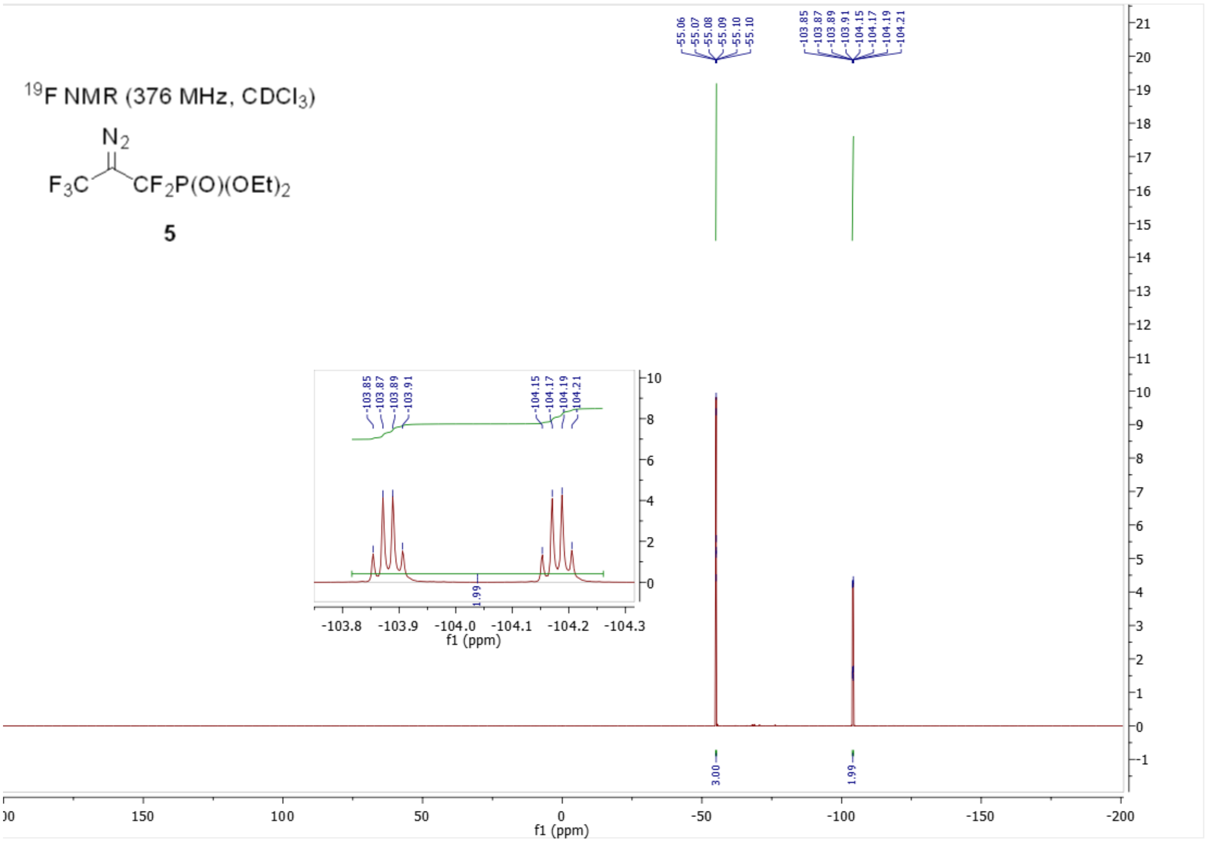

19F NMR (376 MHz, CDCl3)
N2
F3C
CF2P(O)(OEt)2
5
f1 (ppm)

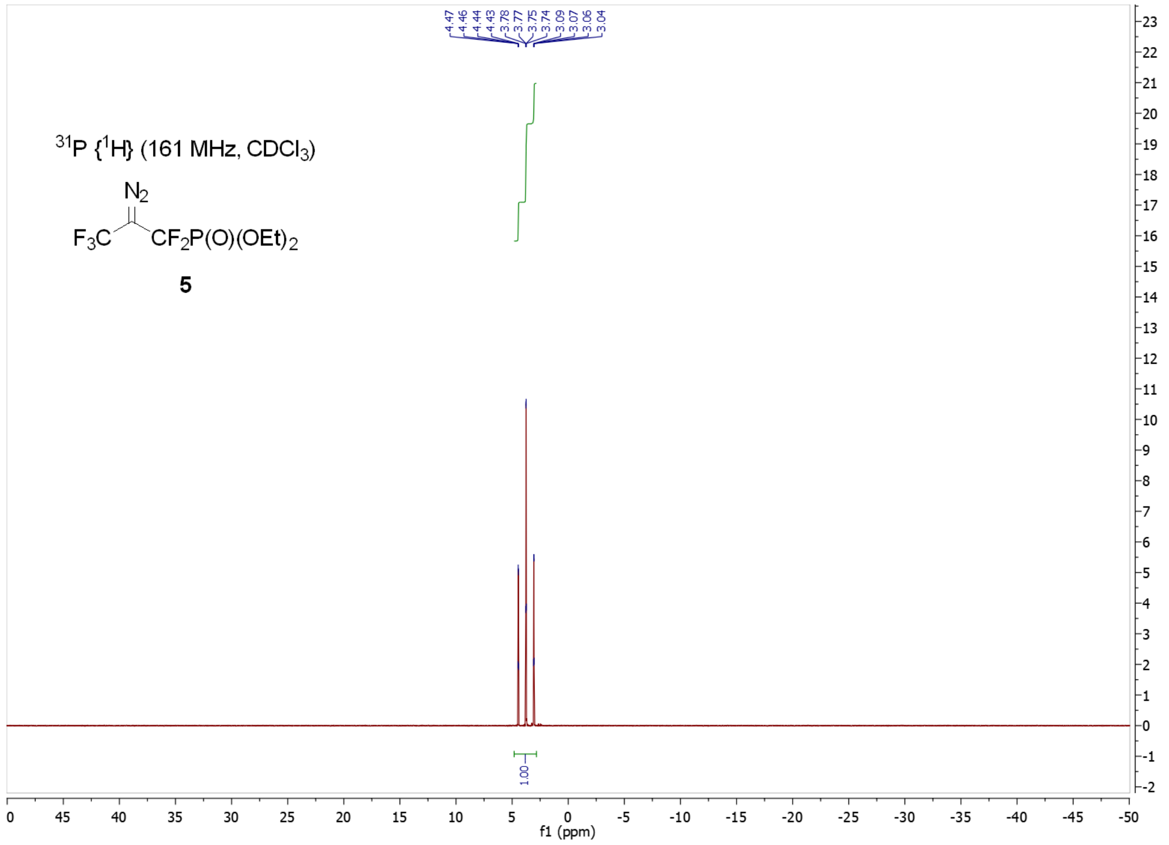
31P {1H} (161 MHz, CDCl3)
N2
F3C CF2P(O)(OEt)2
5
f1 (ppm)

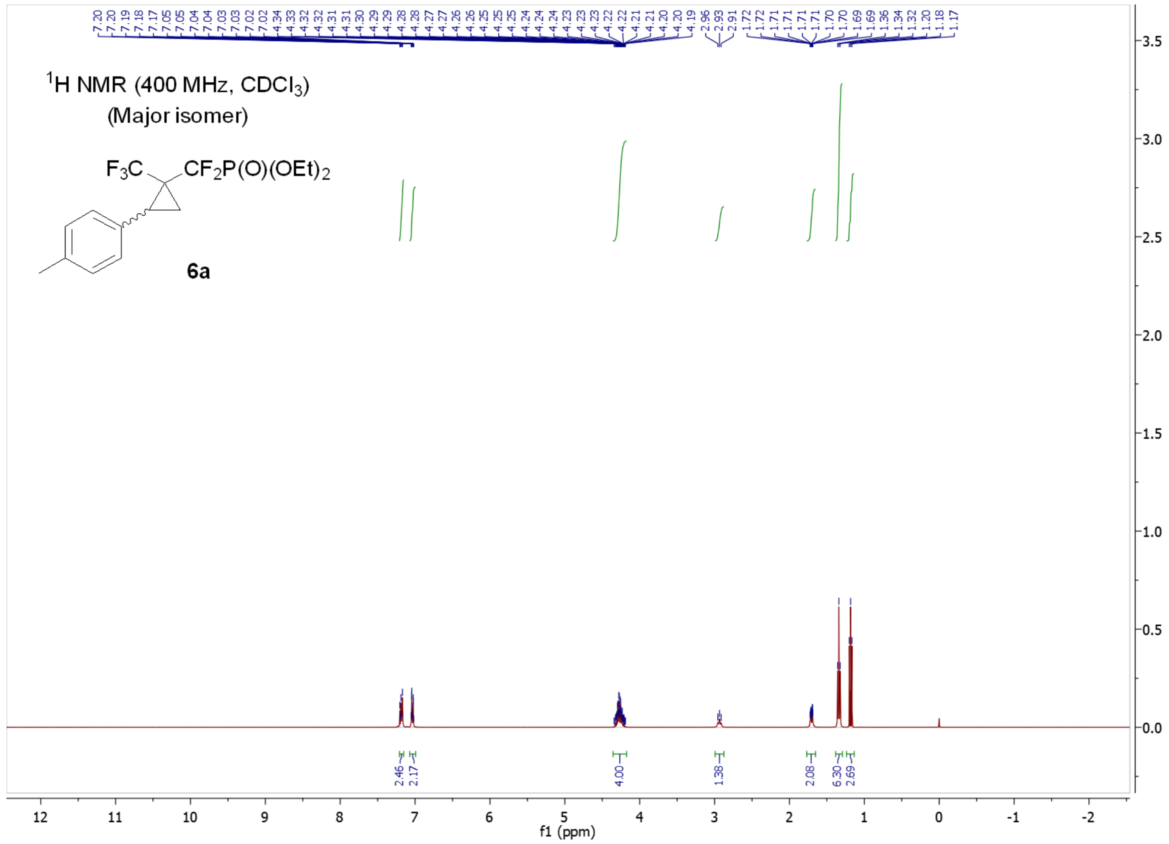
1H NMR (400 MHz, CDCl3)
(Major isomer)
F3C CF2P(O)(OEt)2
6a
f1 (ppm)

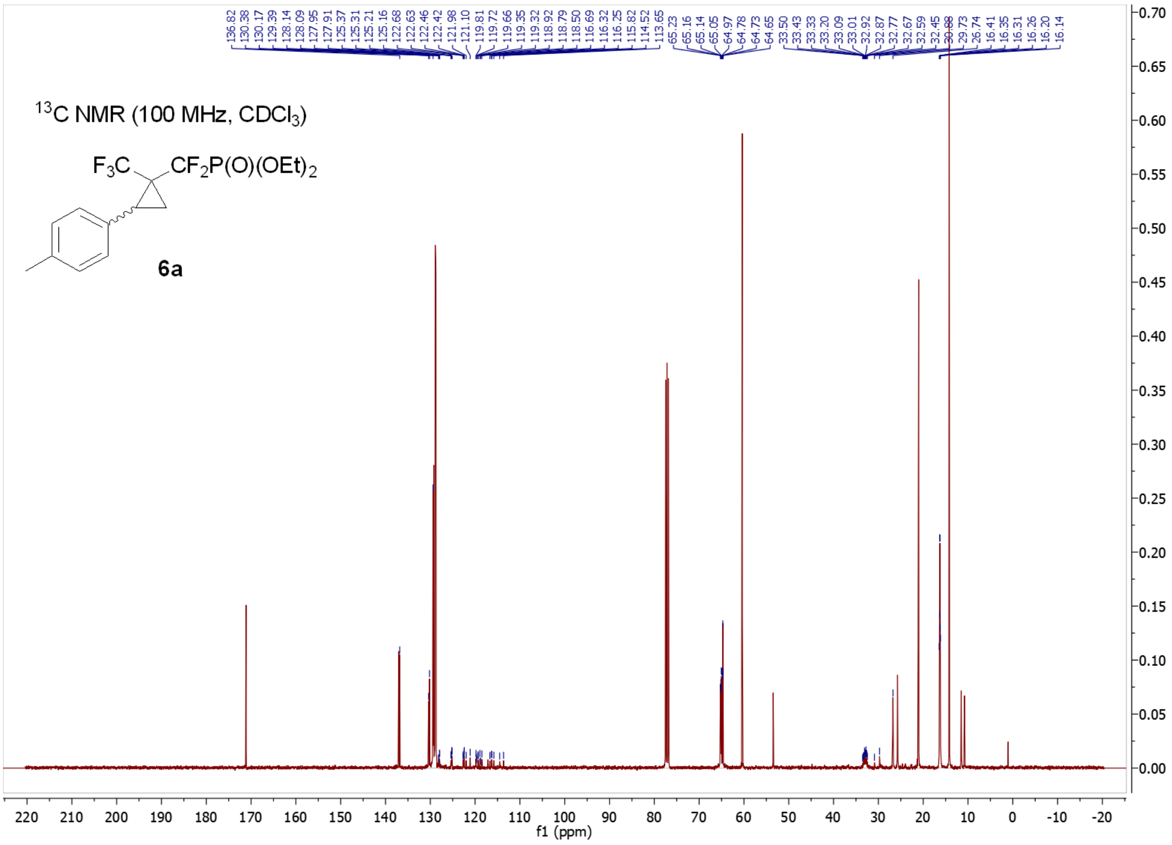

$^{13}$C NMR (100 MHz, CDCl$_3$)
F$_3$C CF$_2$P(O)(OEt)$_2$
6a
f1 (ppm)


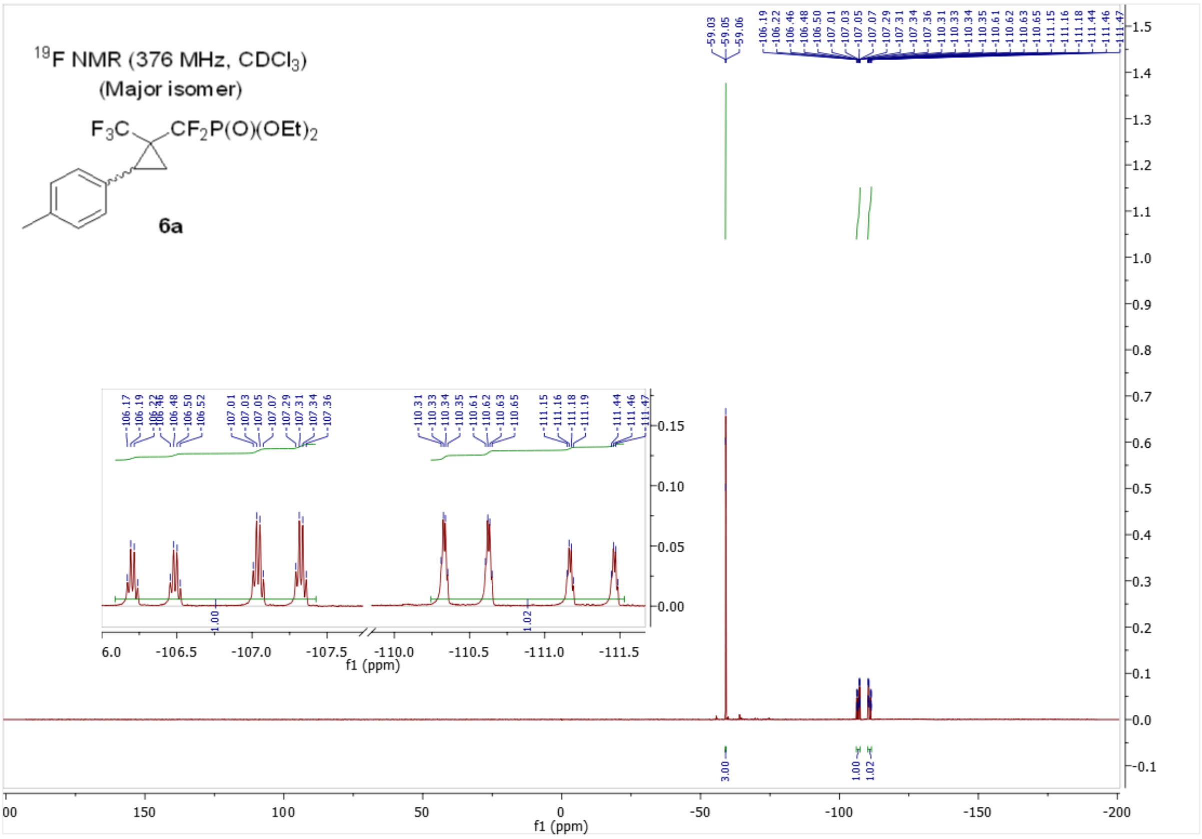

$^{19}$F NMR (376 MHz, CDCl$_3$)
(Major isomer)
F$_3$C CF$_2$P(O)(OEt)$_2$
6a
f1 (ppm)

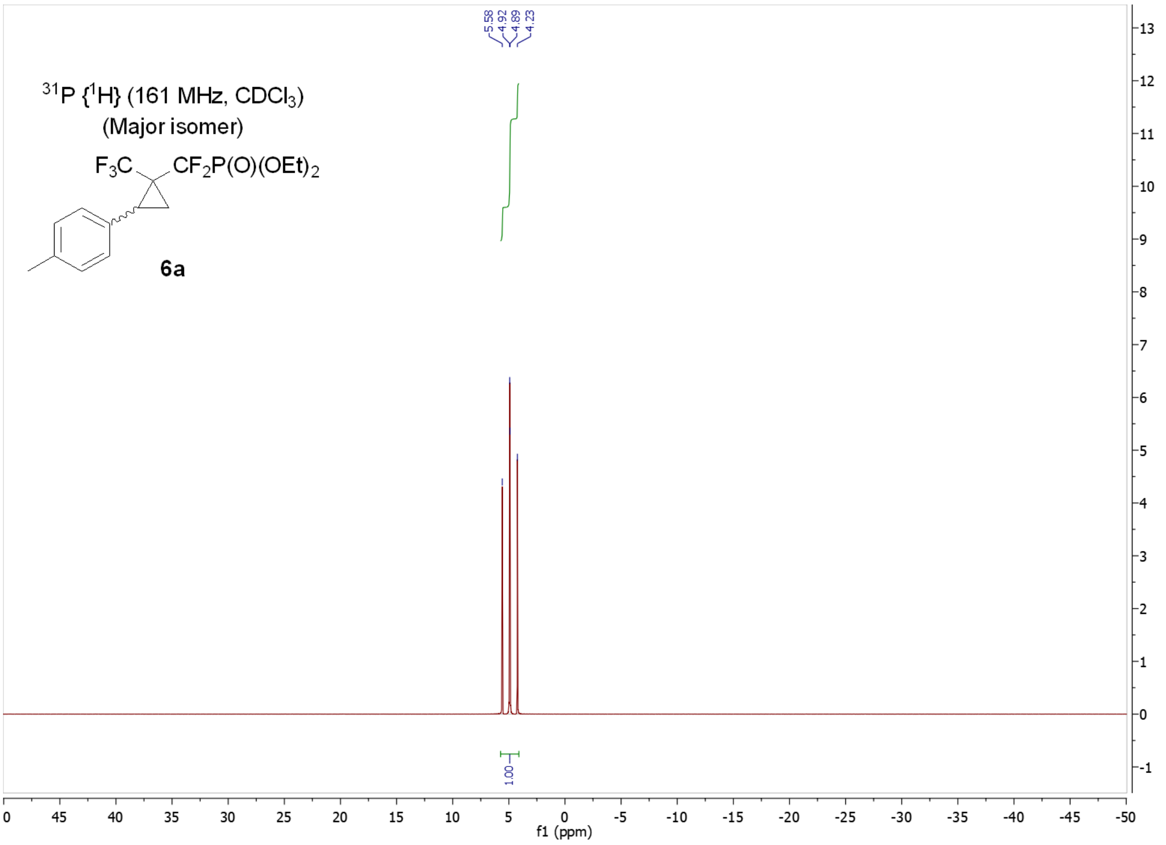

$^{31}$P {$^{1}$H} (161 MHz, CDCl$_3$)
(Major isomer)
F$_3$C
CF$_2$P(O)(OEt)$_2$
6a
5.58
4.92
4.89
4.23
1.00
f1 (ppm)


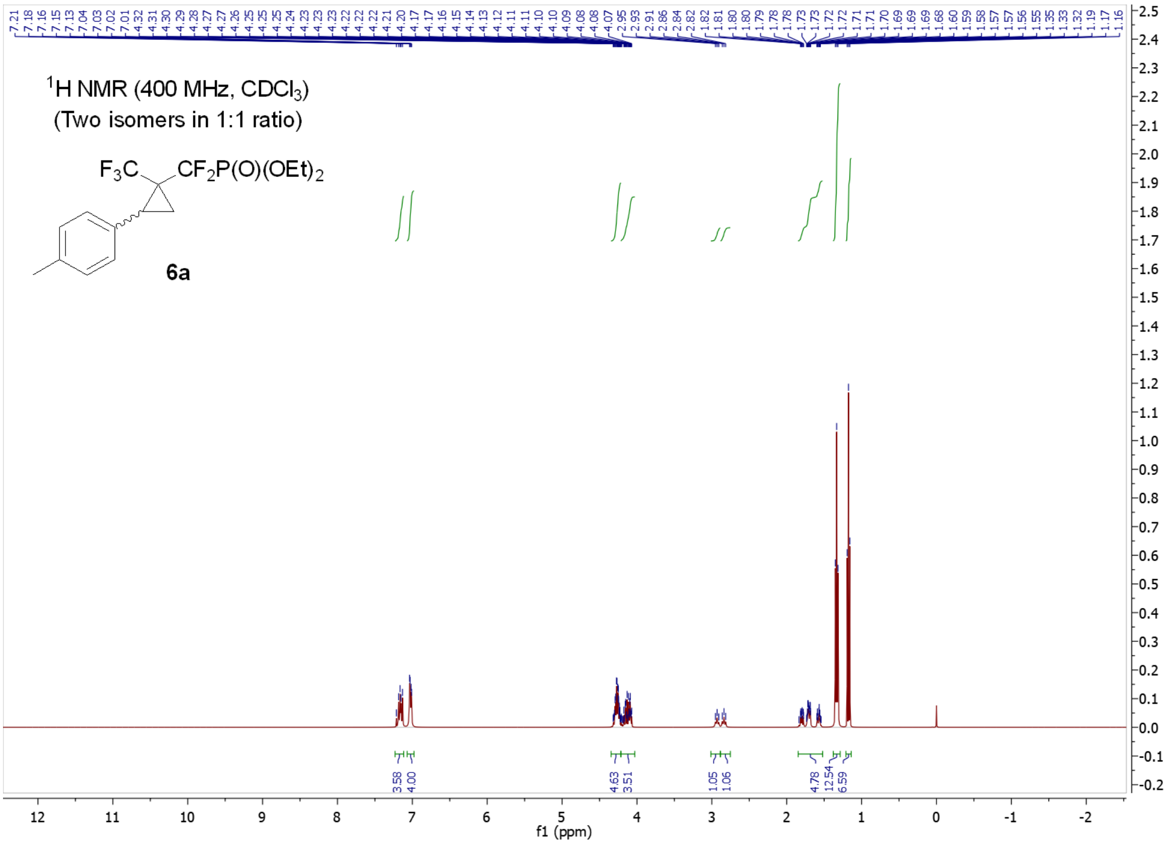

$^{1}$H NMR (400 MHz, CDCl$_3$)
(Two isomers in 1:1 ratio)
F$_3$C
CF$_2$P(O)(OEt)$_2$
6a
3.58
4.00
4.63
3.51
1.05
1.06
4.78
12.54
6.59
f1 (ppm)

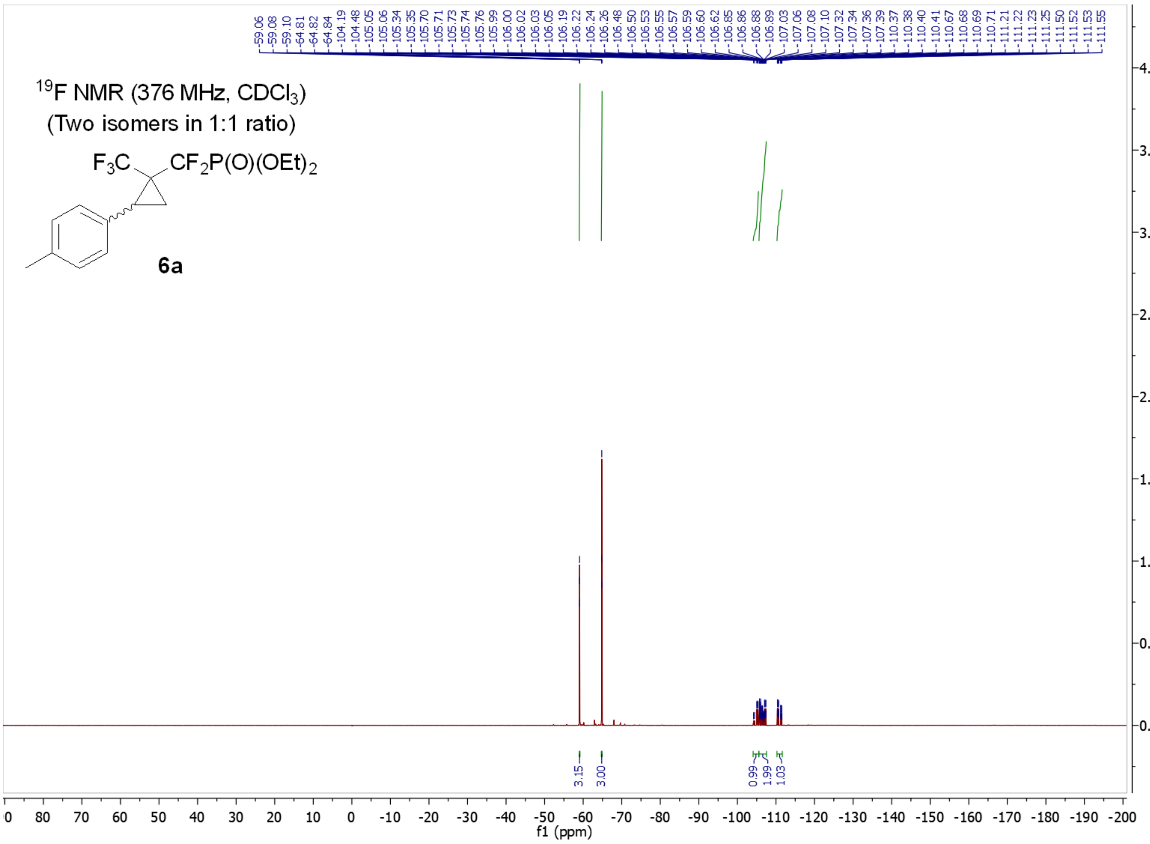
19F NMR (376 MHz, CDCl3)
(Two isomers in 1:1 ratio)
F3C CF2P(O)(OEt)2
6a
f1 (ppm)

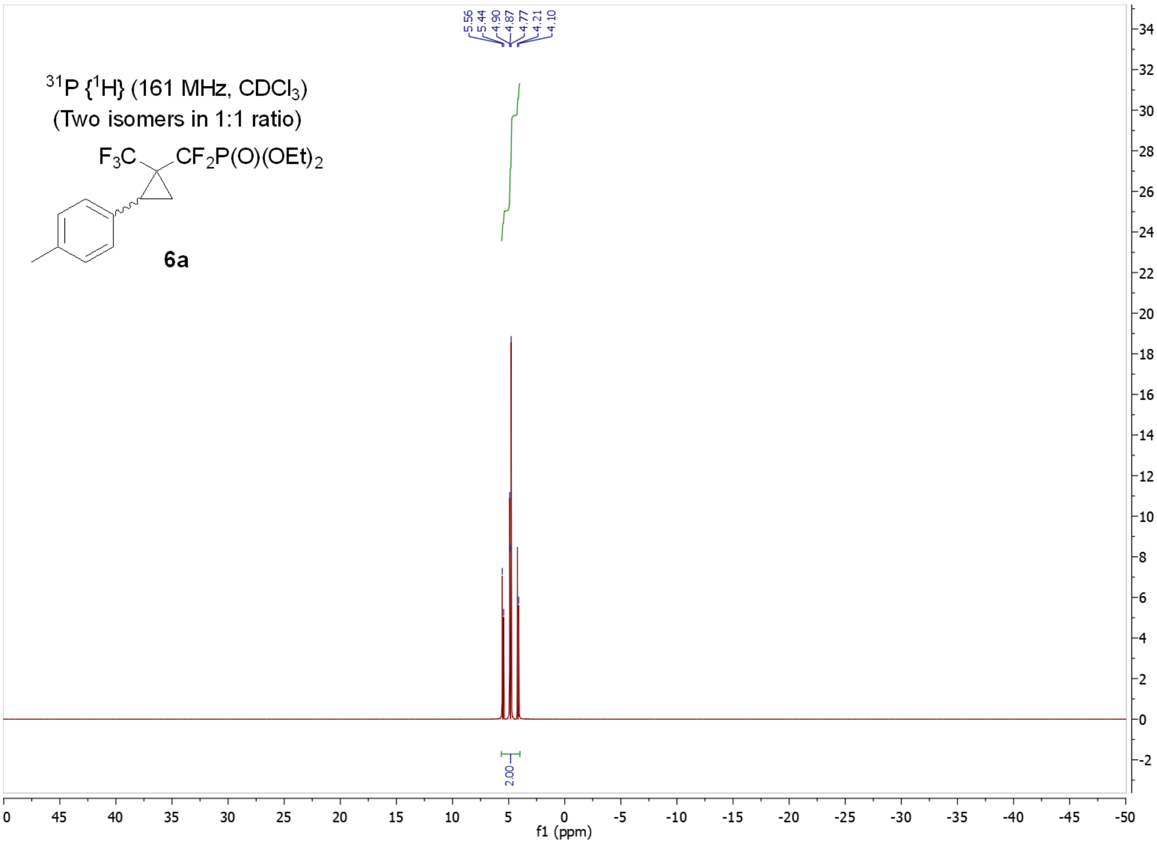
31P {1H} (161 MHz, CDCl3)
(Two isomers in 1:1 ratio)
F3C CF2P(O)(OEt)2
6a
f1 (ppm)

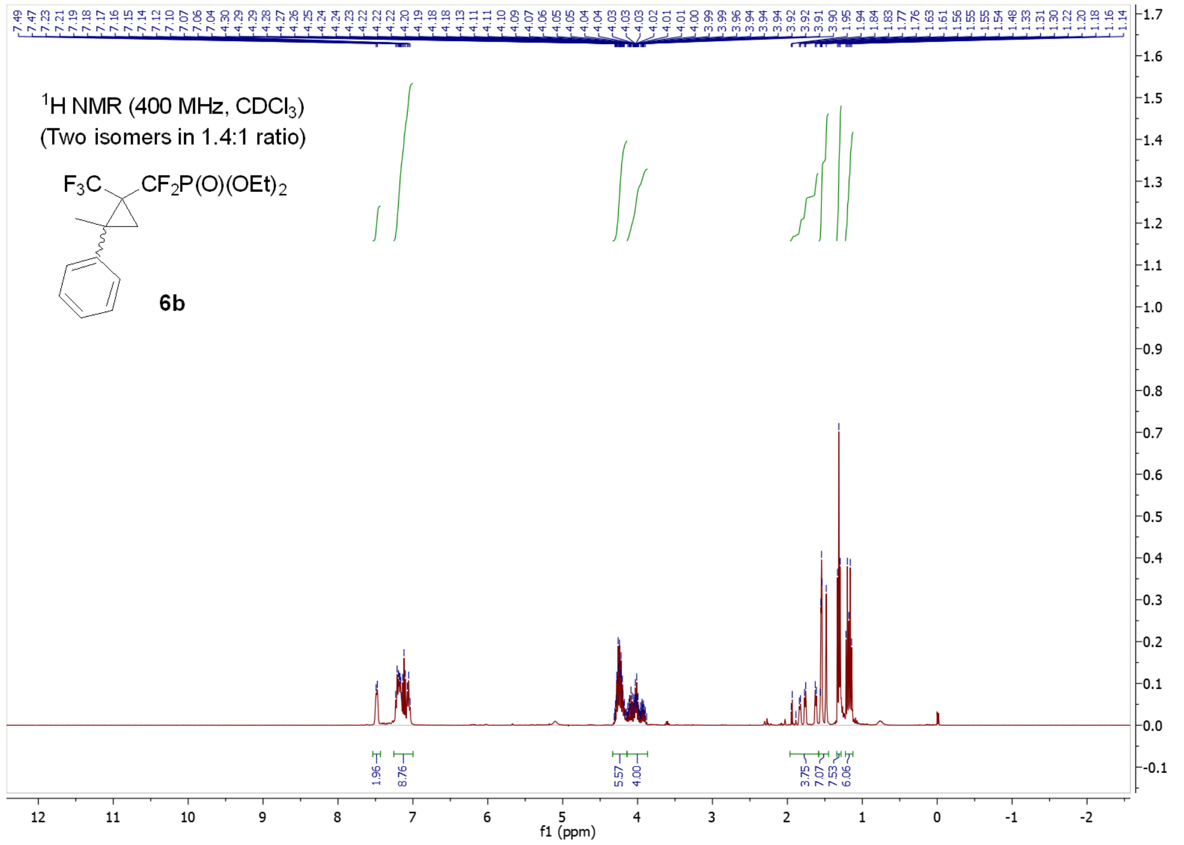

$^{1}H$ NMR (400 MHz, $CDCl_3$)
(Two isomers in 1.4:1 ratio)
$F_3C$
$CF_2P(O)(OEt)_2$
6b
f1 (ppm)


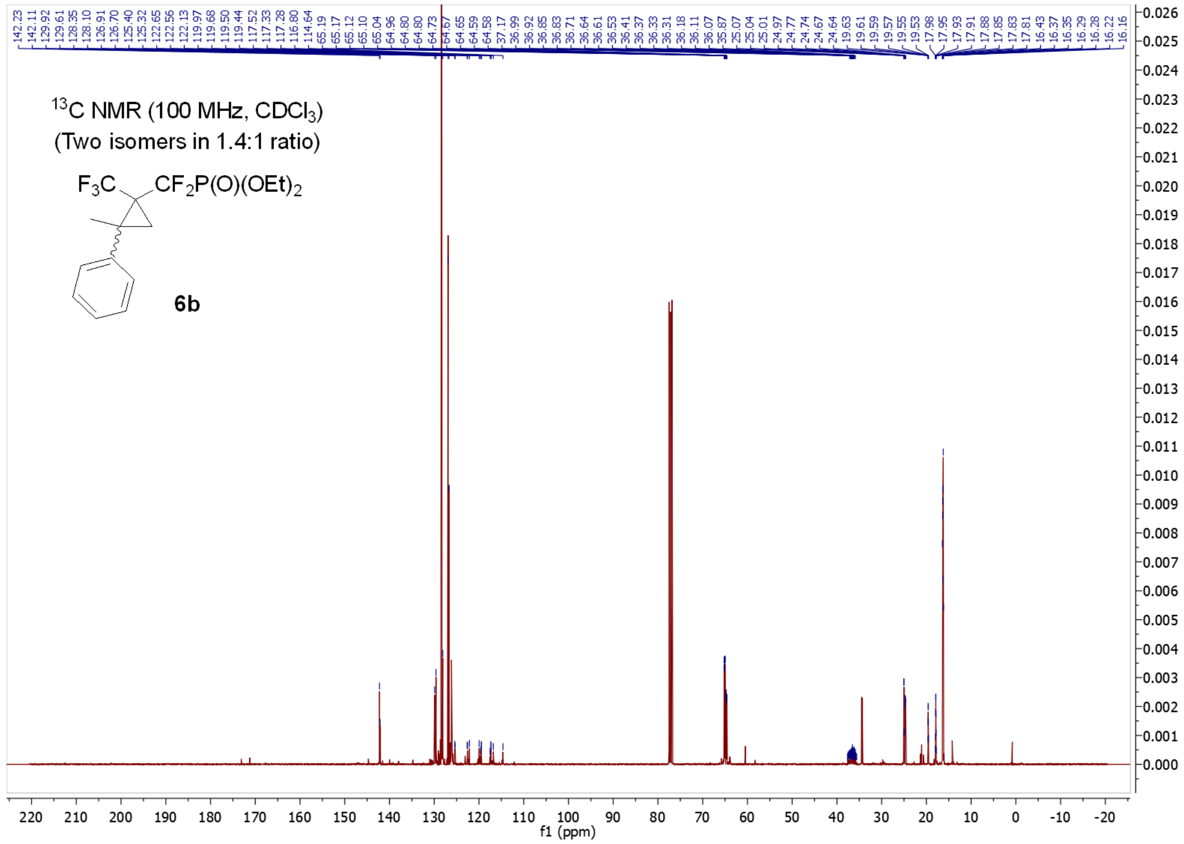

$^{13}C$ NMR (100 MHz, $CDCl_3$)
(Two isomers in 1.4:1 ratio)
$F_3C$
$CF_2P(O)(OEt)_2$
6b
f1 (ppm)

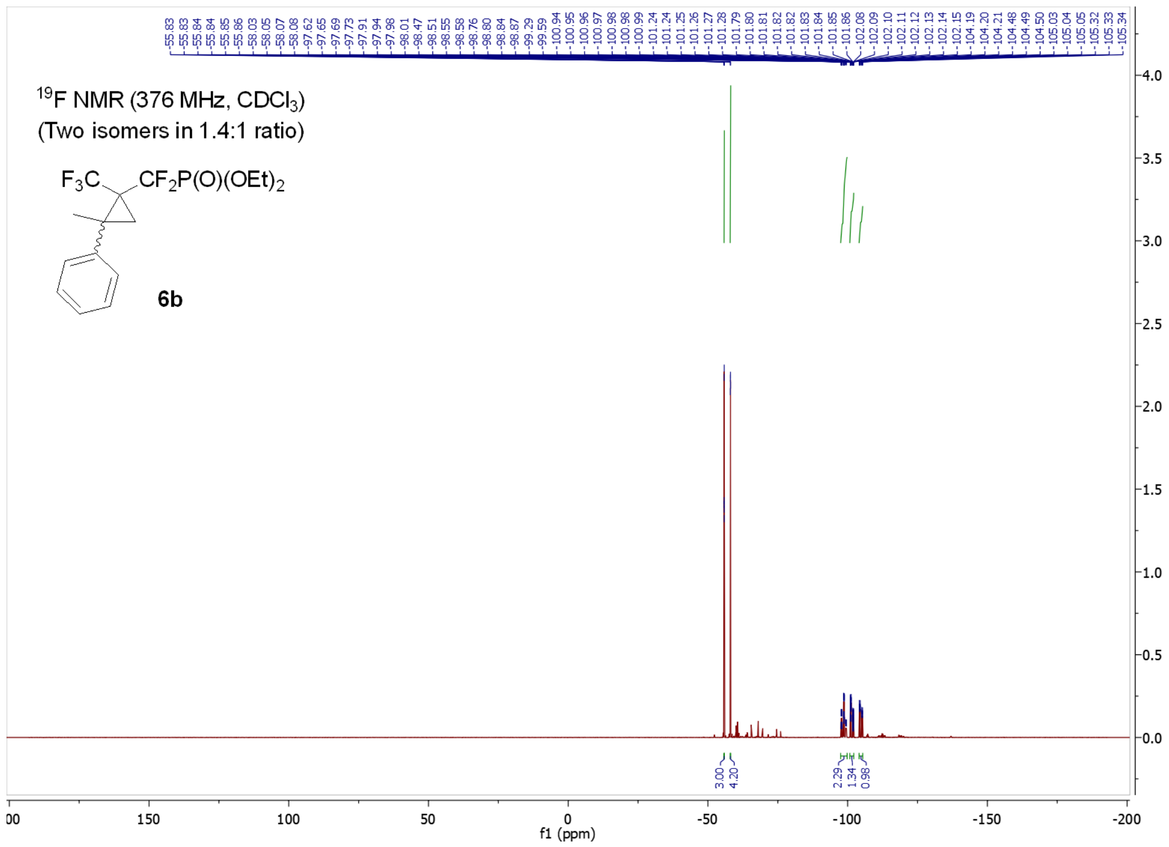

19F NMR (376 MHz, CDCl3)
(Two isomers in 1.4:1 ratio)
F3C
CF2P(O)(OEt)2
6b
f1 (ppm)


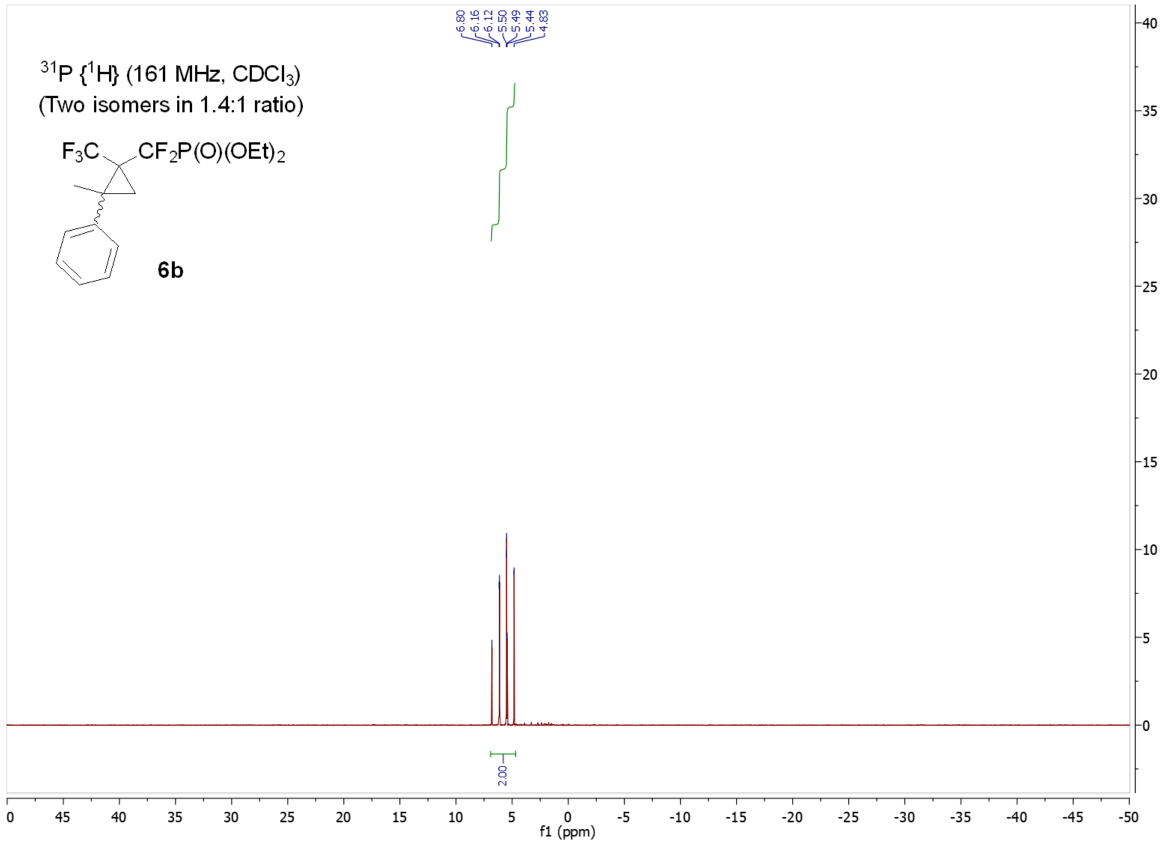

31P {1H} (161 MHz, CDCl3)
(Two isomers in 1.4:1 ratio)
F3C
CF2P(O)(OEt)2
6b
f1 (ppm)

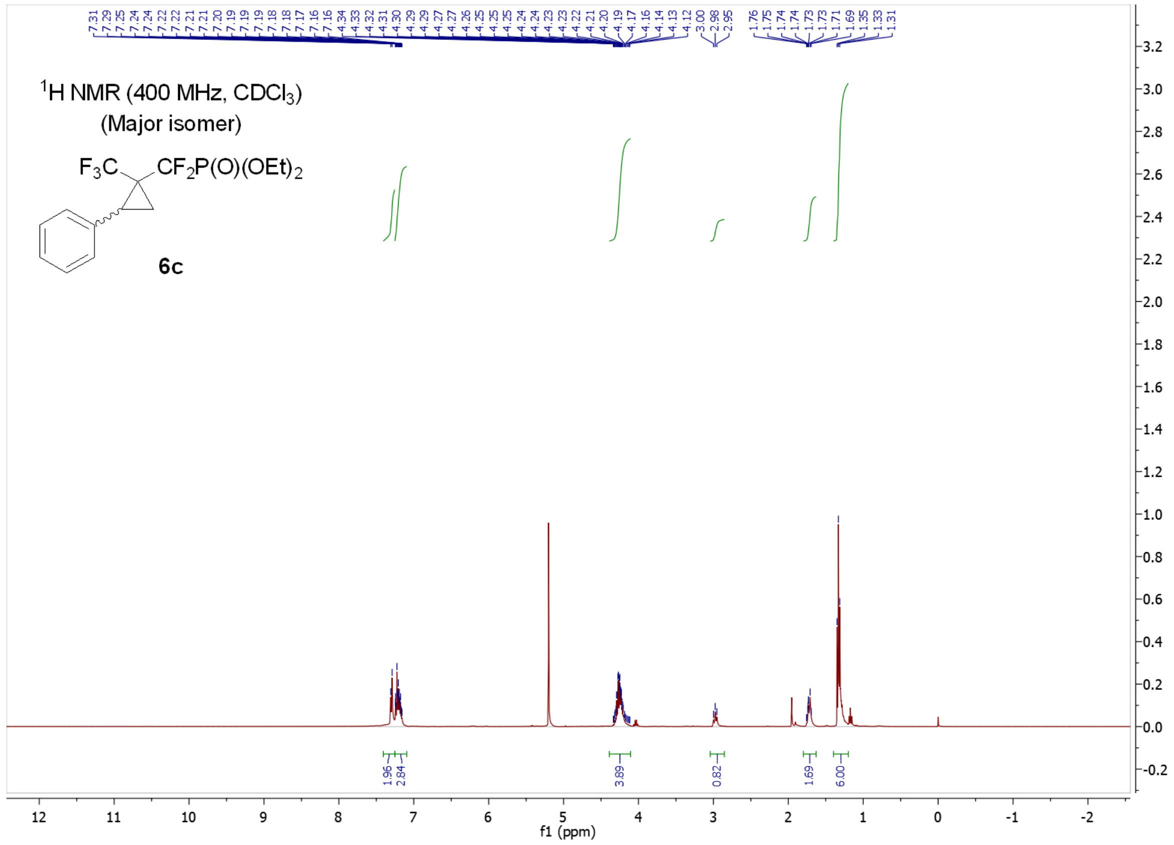
$^1$H NMR (400 MHz, CDCl$_3$)
(Major isomer)
F$_3$C
CF$_2$P(O)(OEt)$_2$
6c
f1 (ppm)

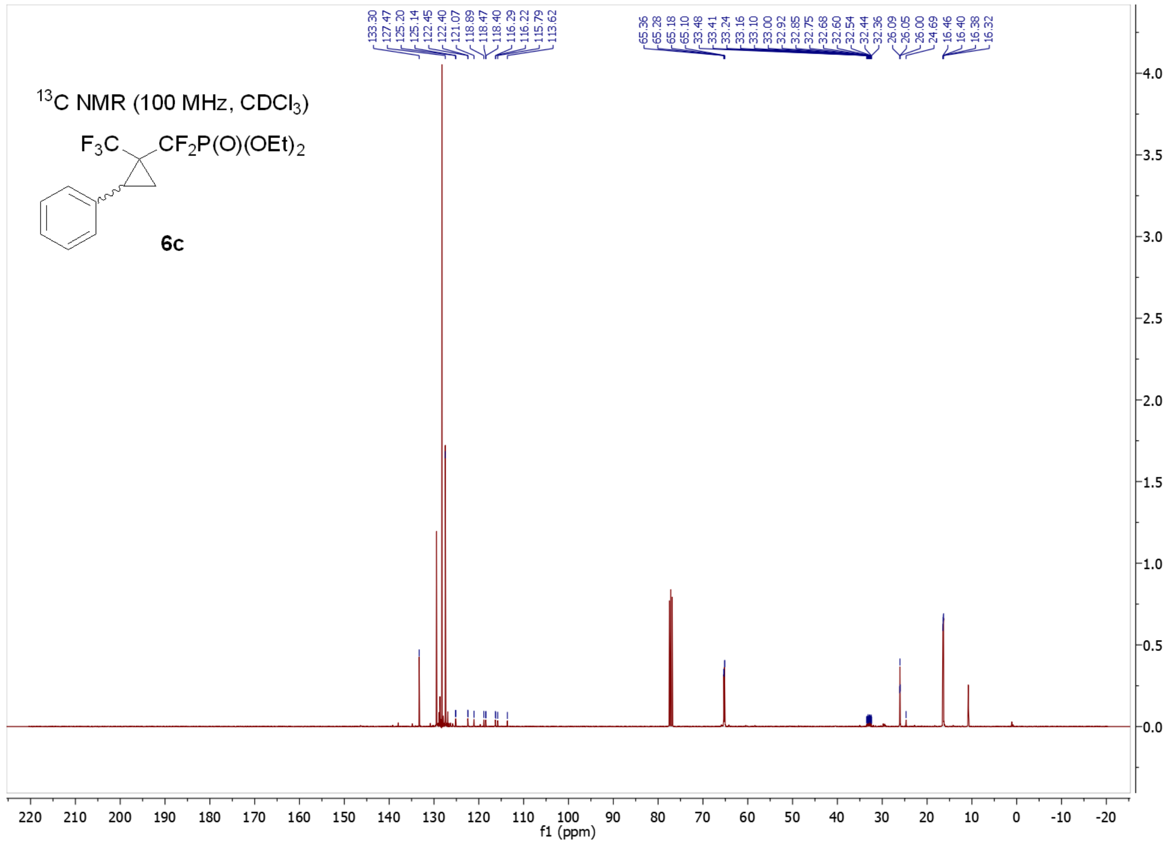
$^{13}$C NMR (100 MHz, CDCl$_3$)
F$_3$C
CF$_2$P(O)(OEt)$_2$
6c
f1 (ppm)

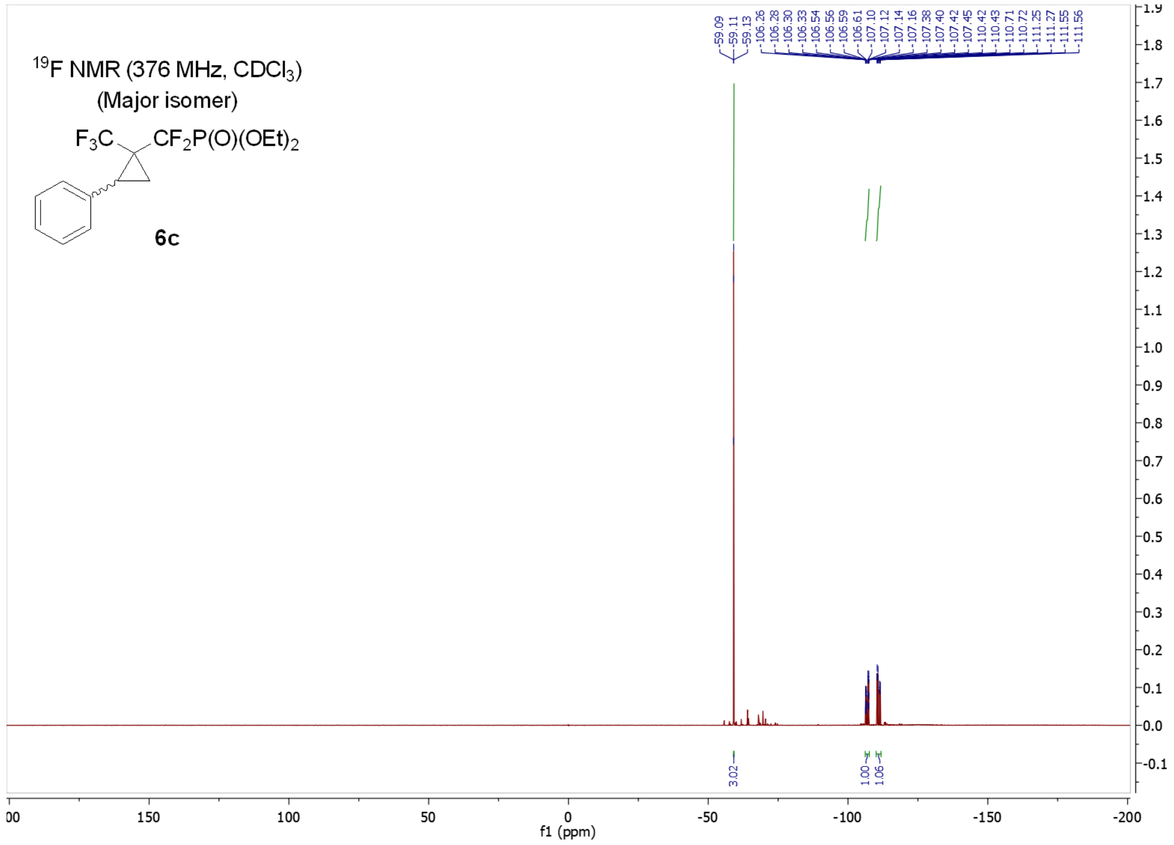
19F NMR (376 MHz, CDCl3)
(Major isomer)
F3C
CF2P(O)(OEt)2
6c
f1 (ppm)

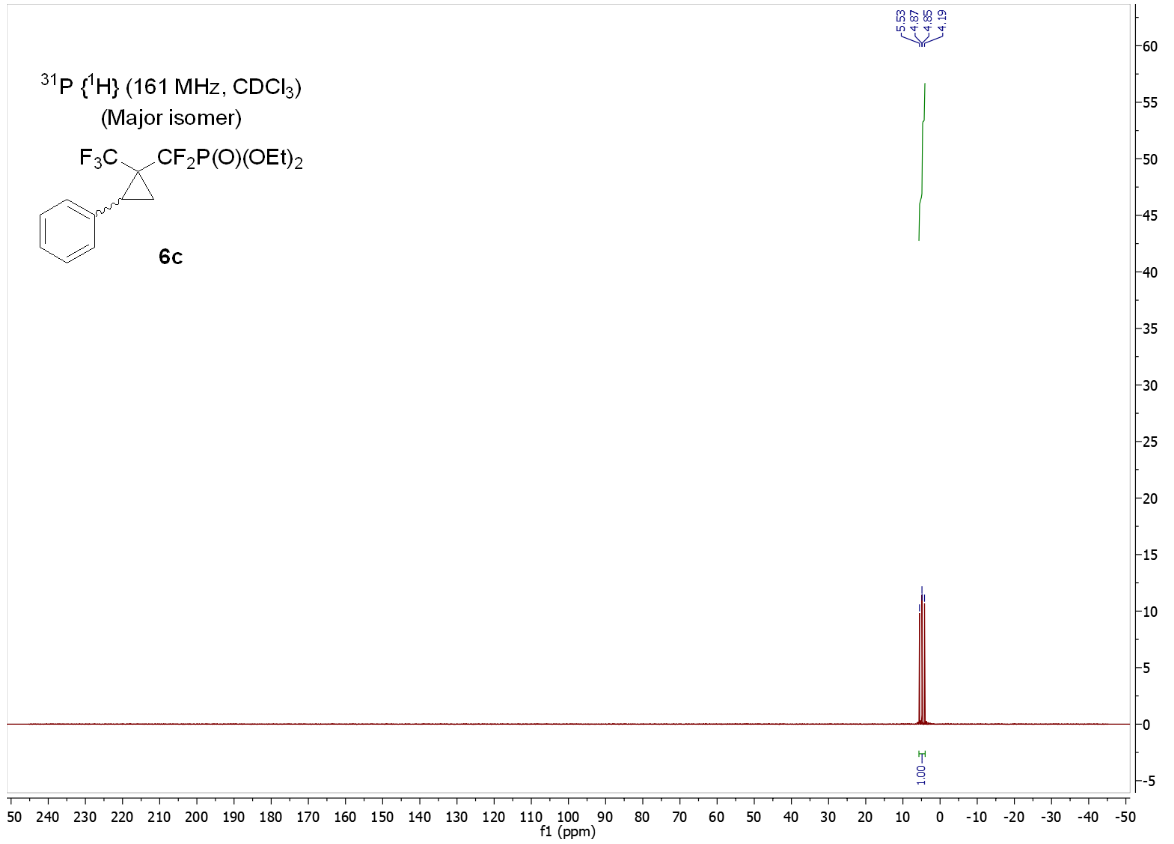
31P {1H} (161 MHz, CDCl3)
(Major isomer)
F3C
CF2P(O)(OEt)2
6c
f1 (ppm)

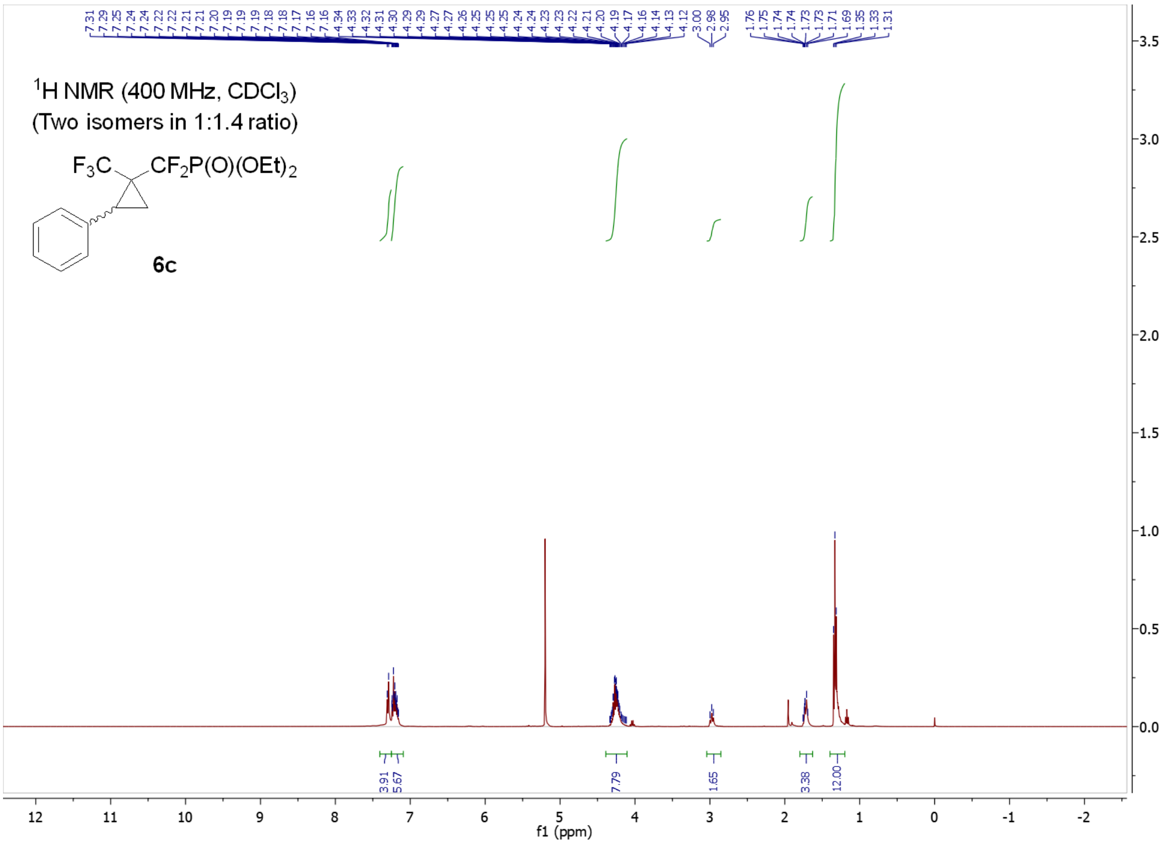
1H NMR (400 MHz, CDCl3)
(Two isomers in 1:1.4 ratio)
F3C
CF2P(O)(OEt)2
6c
f1 (ppm)

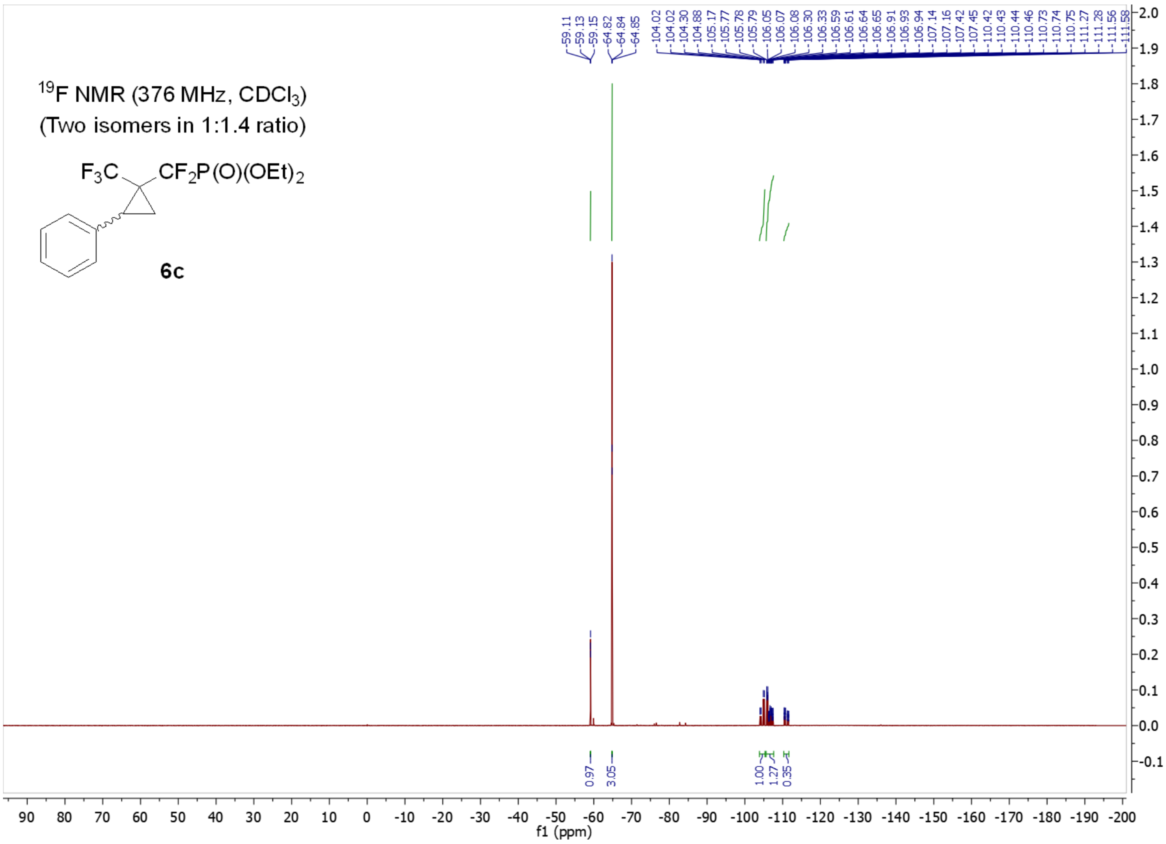
19F NMR (376 MHz, CDCl3)
(Two isomers in 1:1.4 ratio)
F3C
CF2P(O)(OEt)2
6c
f1 (ppm)

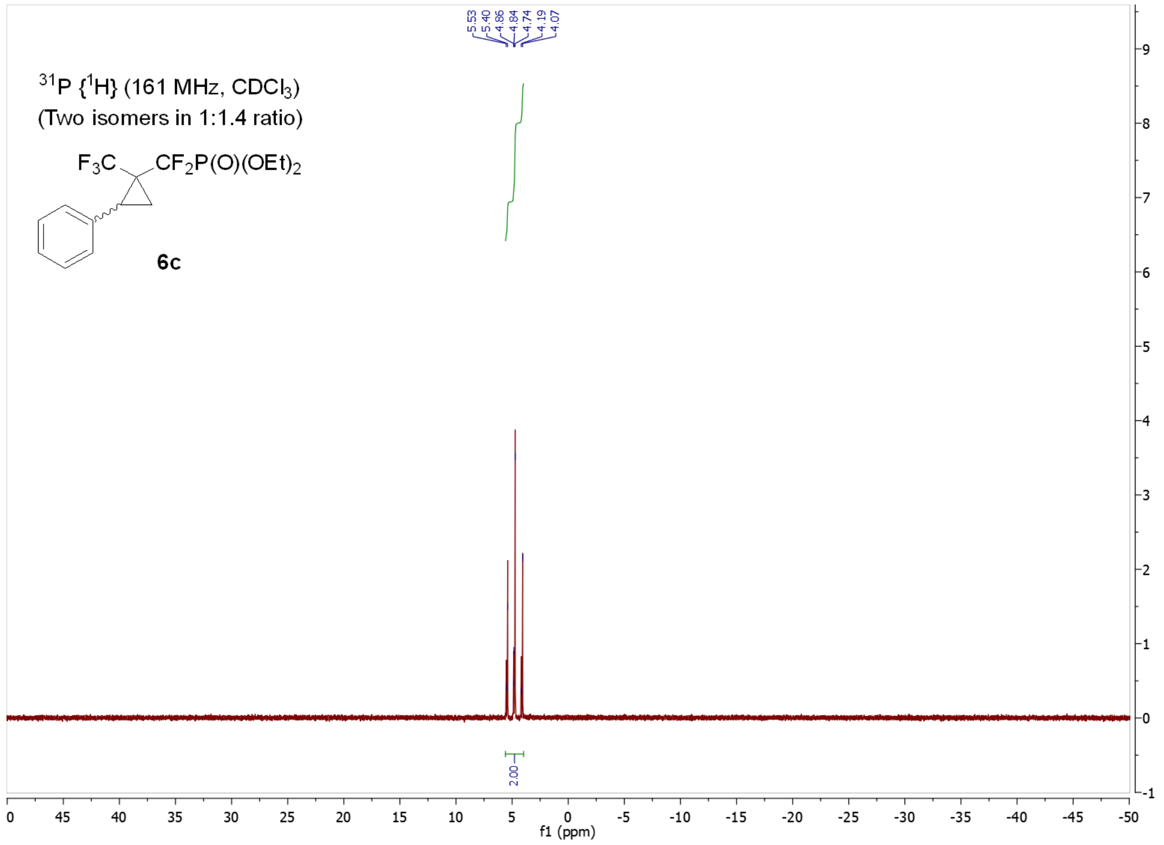
³¹P {¹H} (161 MHz, CDCl₃)
(Two isomers in 1:1.4 ratio)
F3C
CF2P(O)(OEt)2
6c
f1 (ppm)

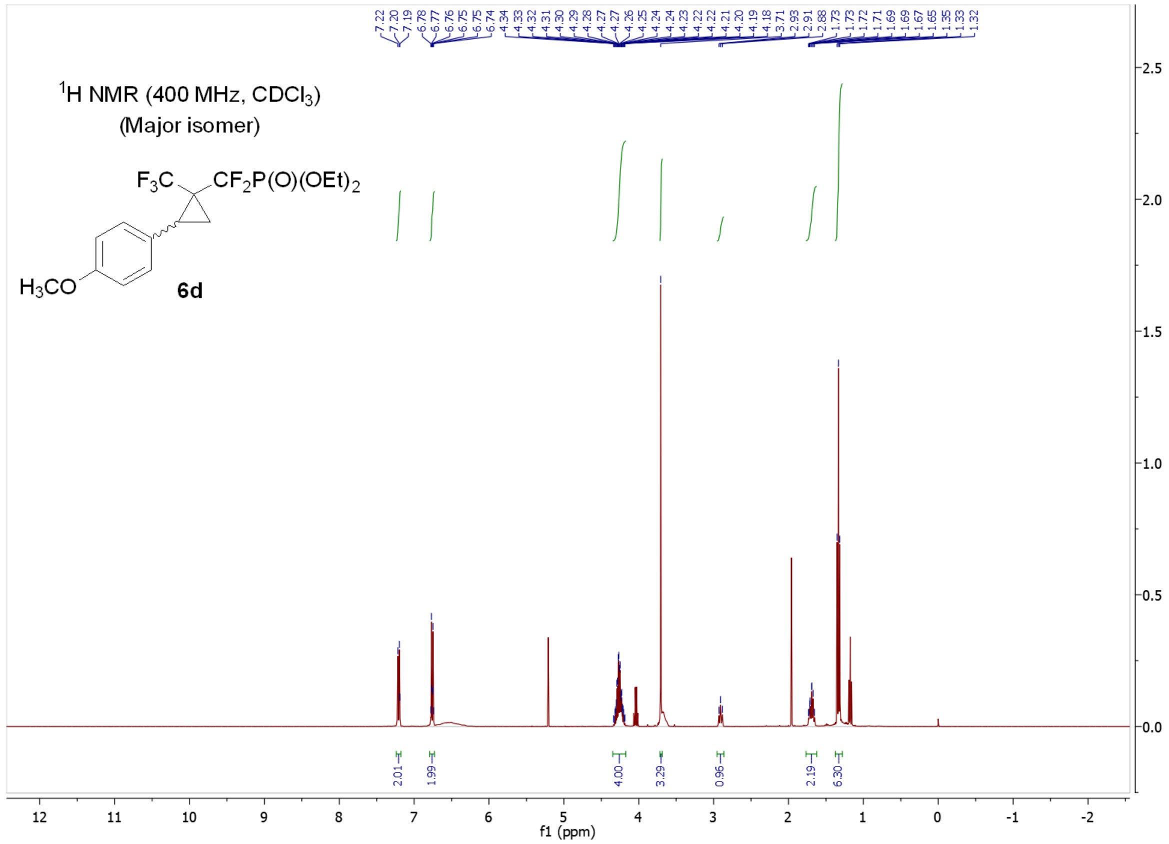
¹H NMR (400 MHz, CDCl₃)
(Major isomer)
F3C
CF2P(O)(OEt)2
H3CO
6d
f1 (ppm)

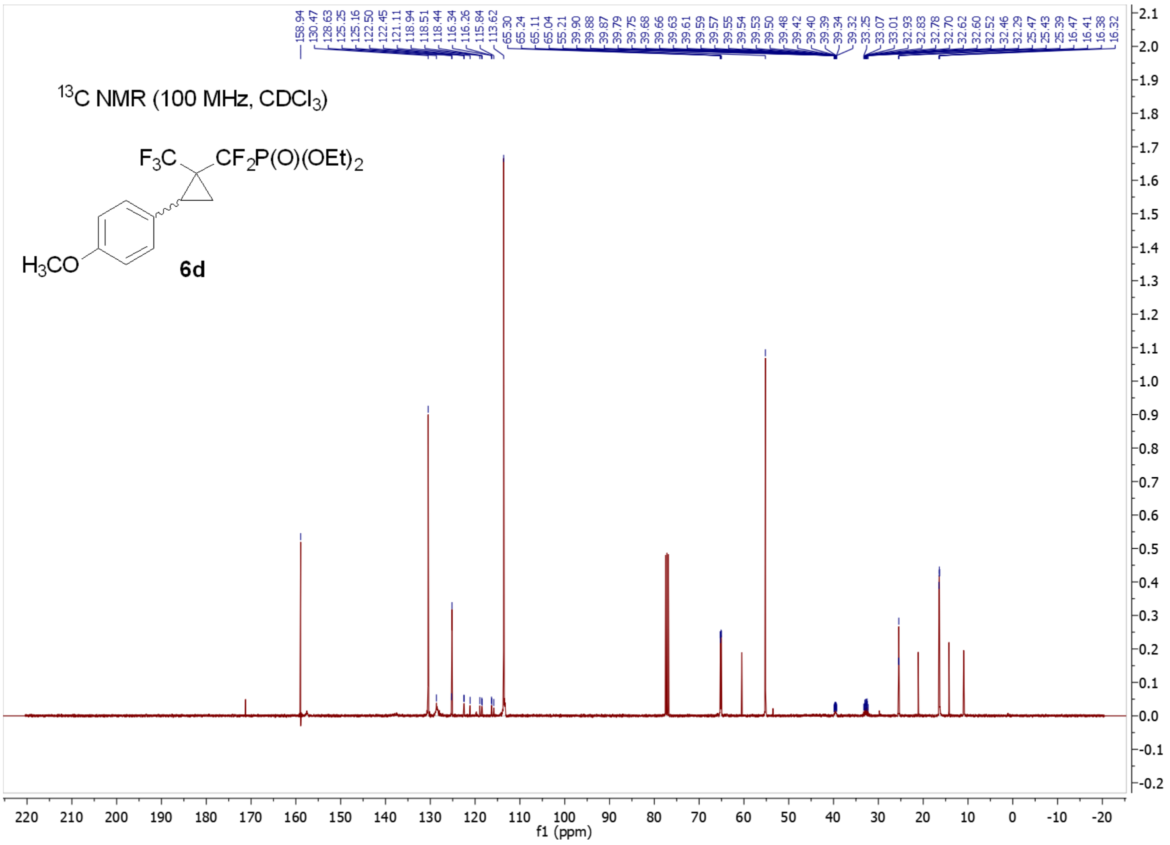
13C NMR (100 MHz, CDCl3)
F3C CF2P(O)(OEt)2
H3CO
6d
f1 (ppm)

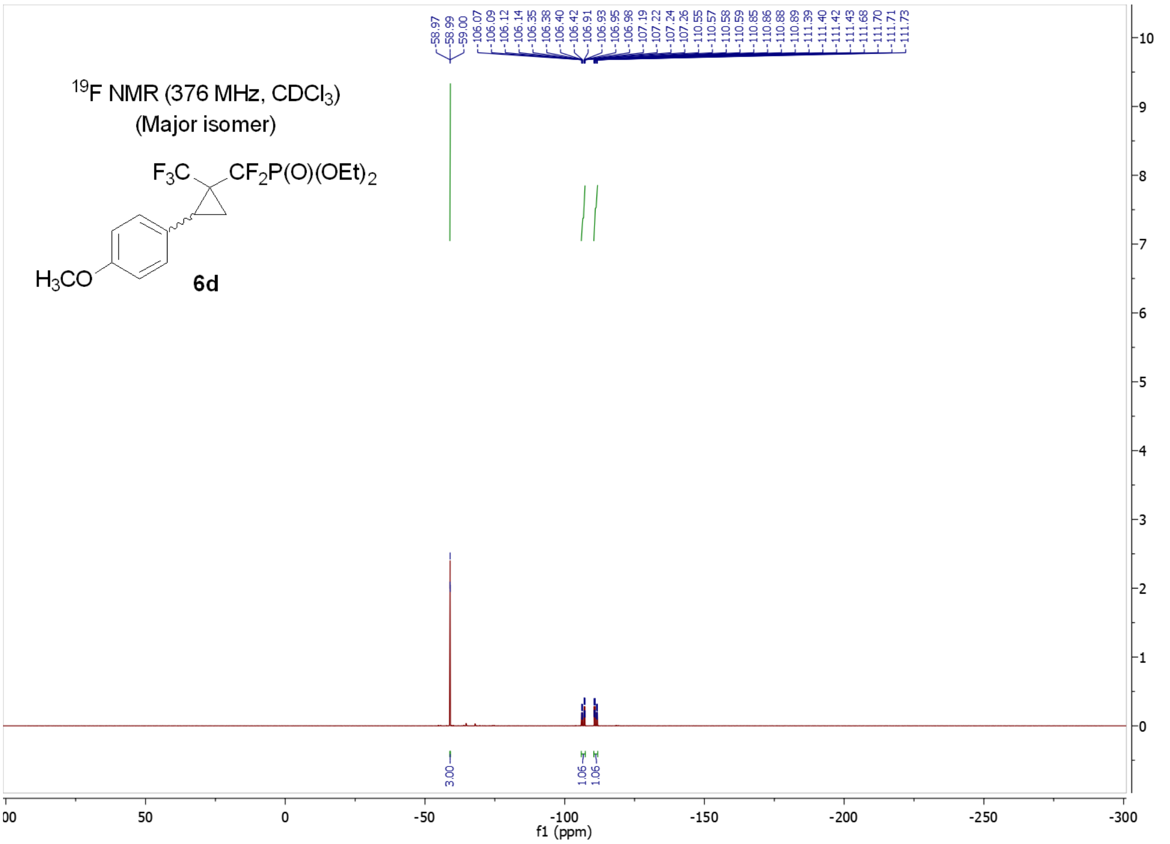
19F NMR (376 MHz, CDCl3)
(Major isomer)
F3C CF2P(O)(OEt)2
H3CO
6d
f1 (ppm)

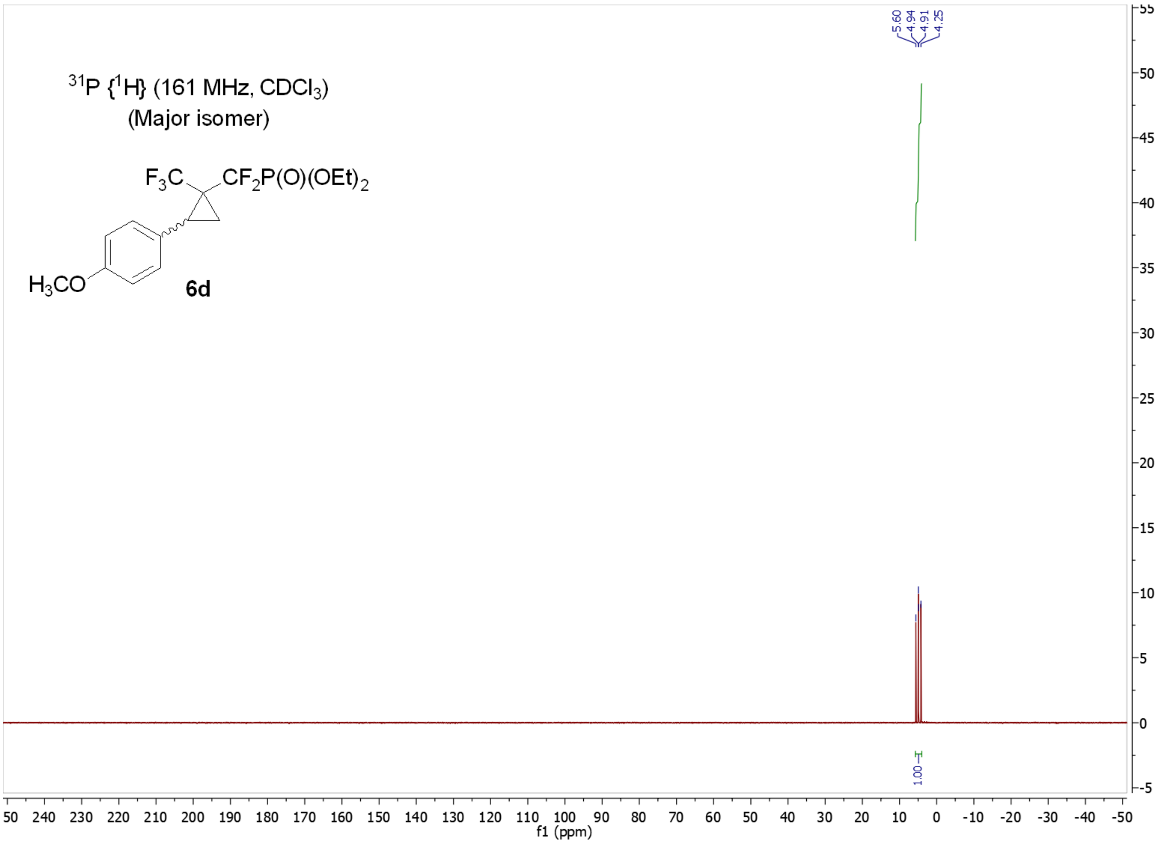

$^{31}P\ \{^1H\}$ (161 MHz, $CDCl_3$)
(Major isomer)
$F_3C$
$CF_2P(O)(OEt)_2$
$H_3CO$
6d
f1 (ppm)


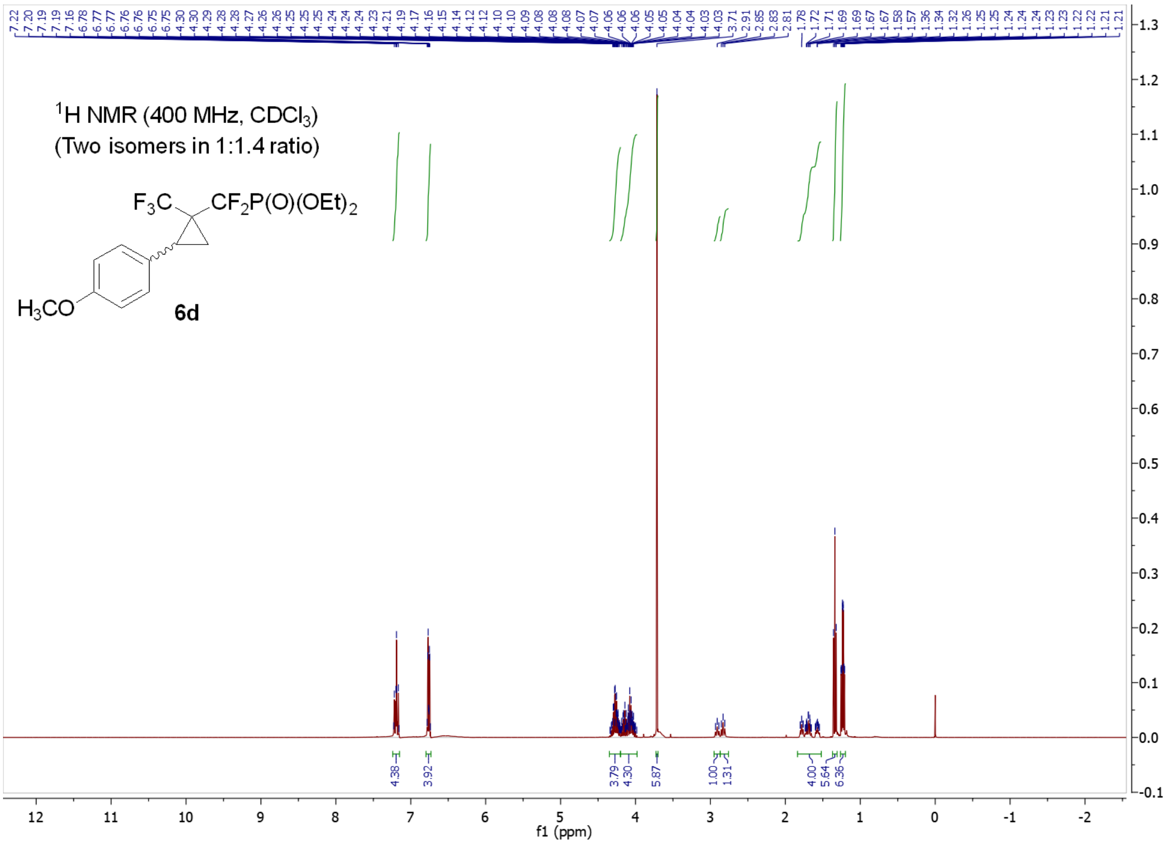

$^1H$ NMR (400 MHz, $CDCl_3$)
(Two isomers in 1:1.4 ratio)
$F_3C$
$CF_2P(O)(OEt)_2$
$H_3CO$
6d
f1 (ppm)

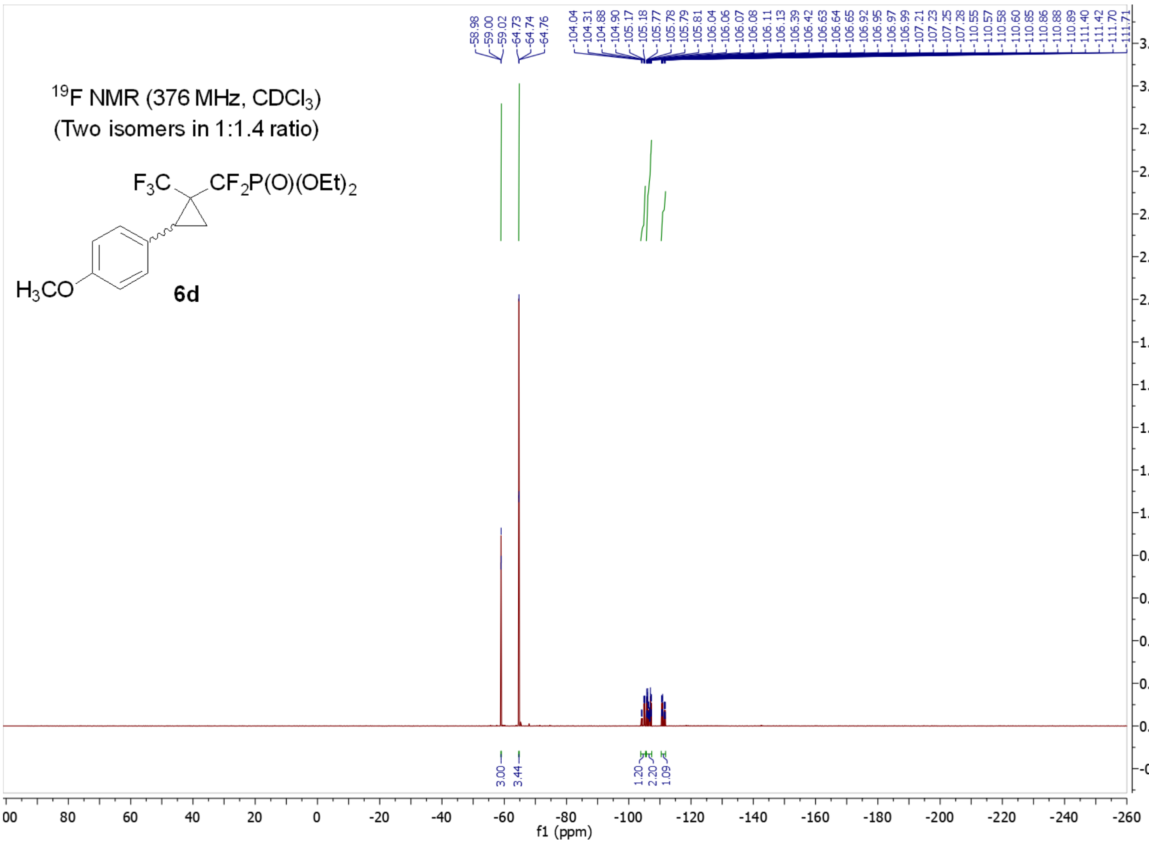
19F NMR (376 MHz, CDCl3)
(Two isomers in 1:1.4 ratio)
F3C
CF2P(O)(OEt)2
H3CO
6d
f1 (ppm)

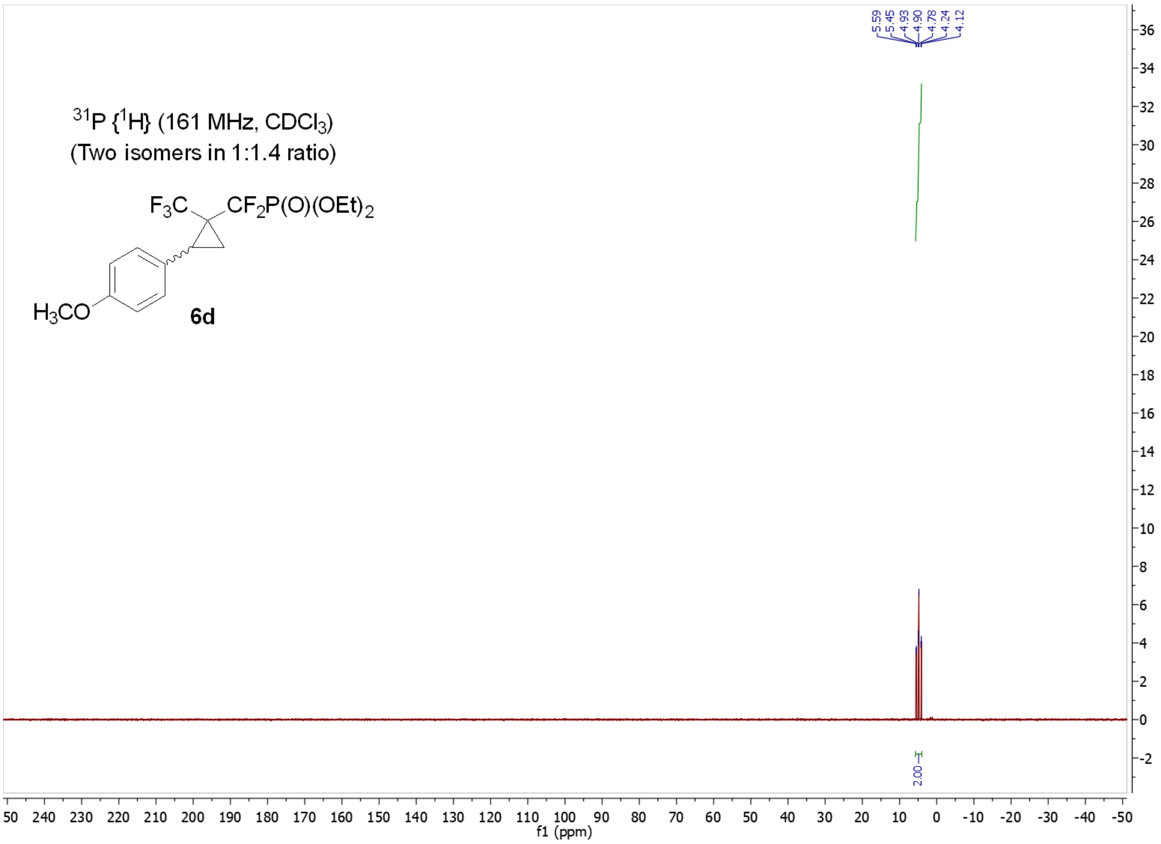
31P {1H} (161 MHz, CDCl3)
(Two isomers in 1:1.4 ratio)
F3C
CF2P(O)(OEt)2
H3CO
6d
f1 (ppm)

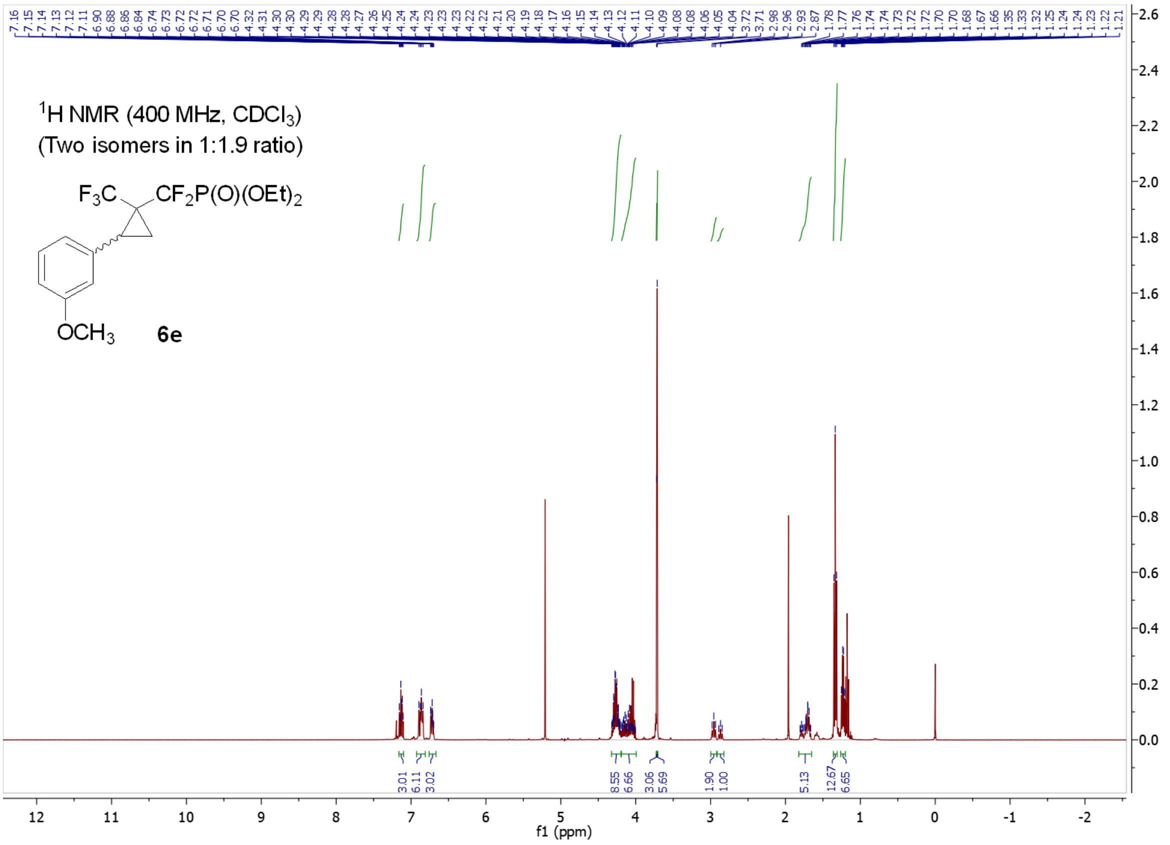
$^{1}$H NMR (400 MHz, $CDCl_3$)
(Two isomers in 1:1.9 ratio)
6e
f1 (ppm)

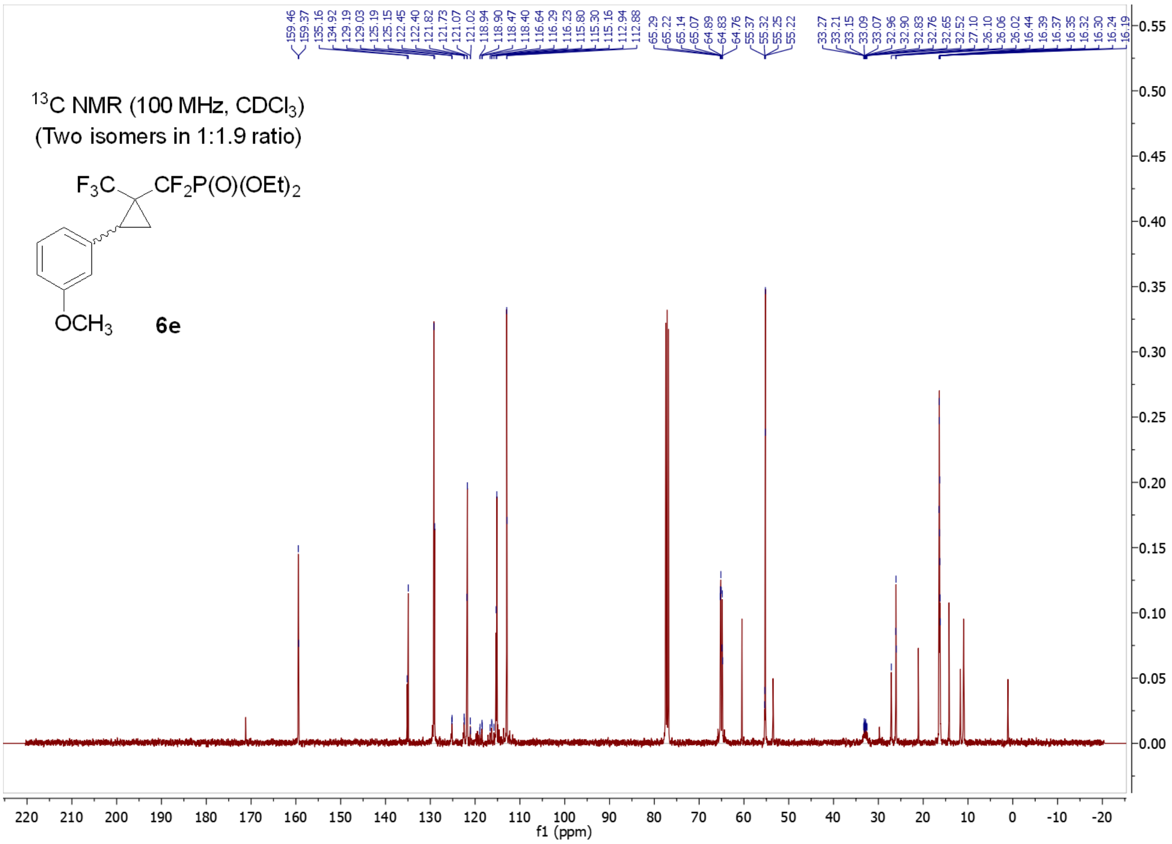
$^{13}$C NMR (100 MHz, $CDCl_3$)
(Two isomers in 1:1.9 ratio)
6e
f1 (ppm)

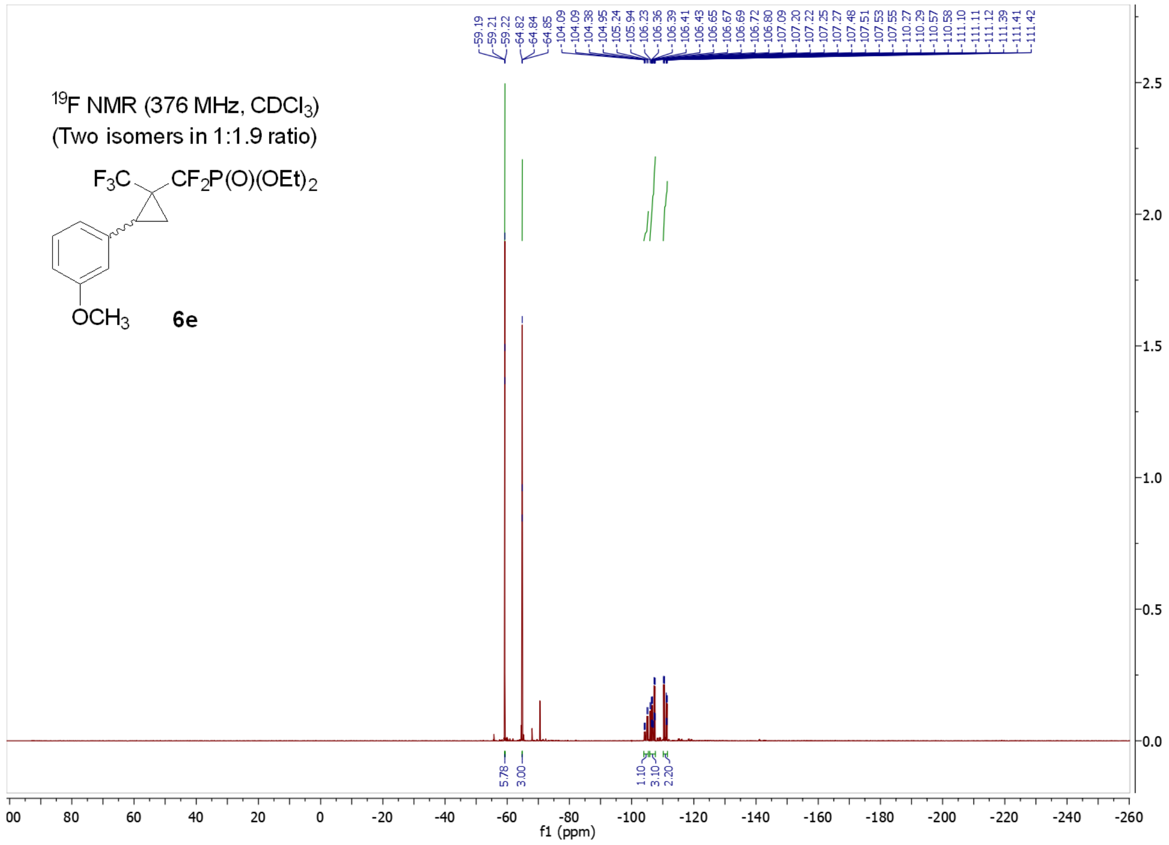

19F NMR (376 MHz, CDCl3)
(Two isomers in 1:1.9 ratio)
F3C
CF2P(O)(OEt)2
OCH3
6e
f1 (ppm)


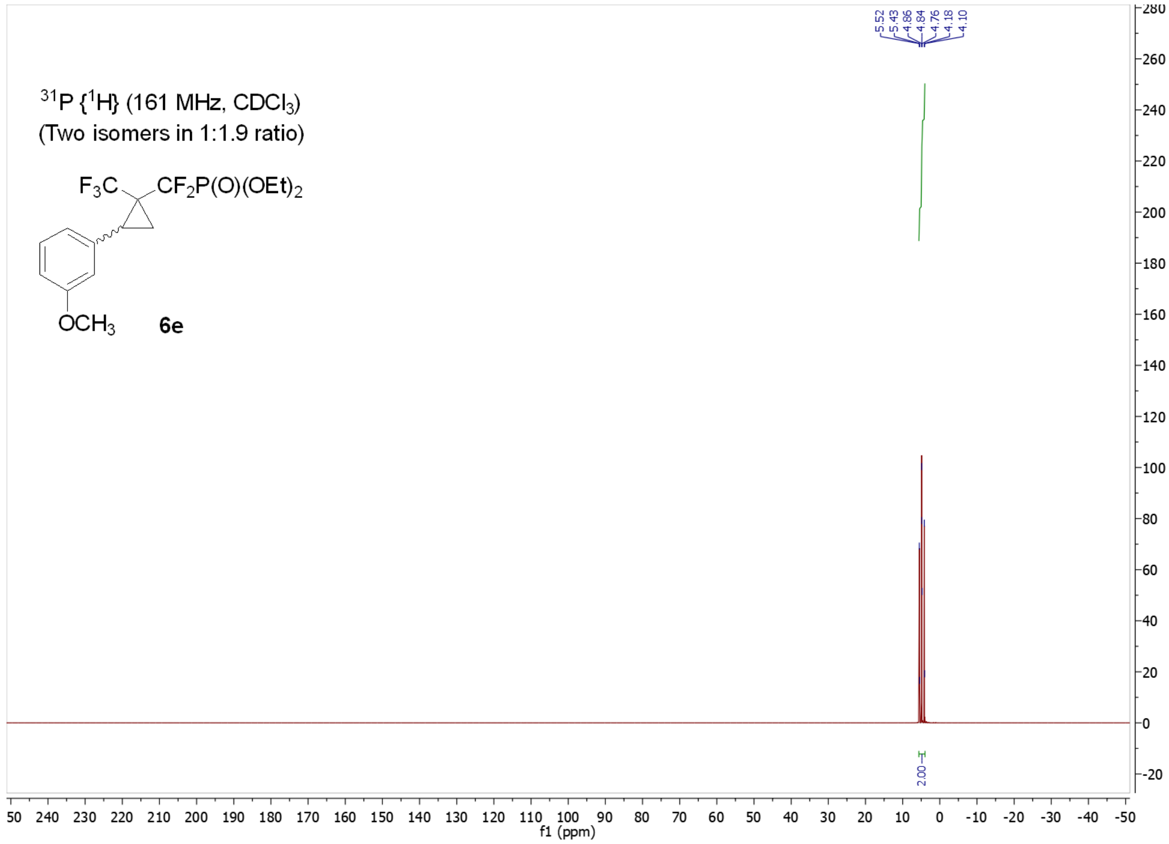

31P {1H} (161 MHz, CDCl3)
(Two isomers in 1:1.9 ratio)
F3C
CF2P(O)(OEt)2
OCH3
6e
f1 (ppm)

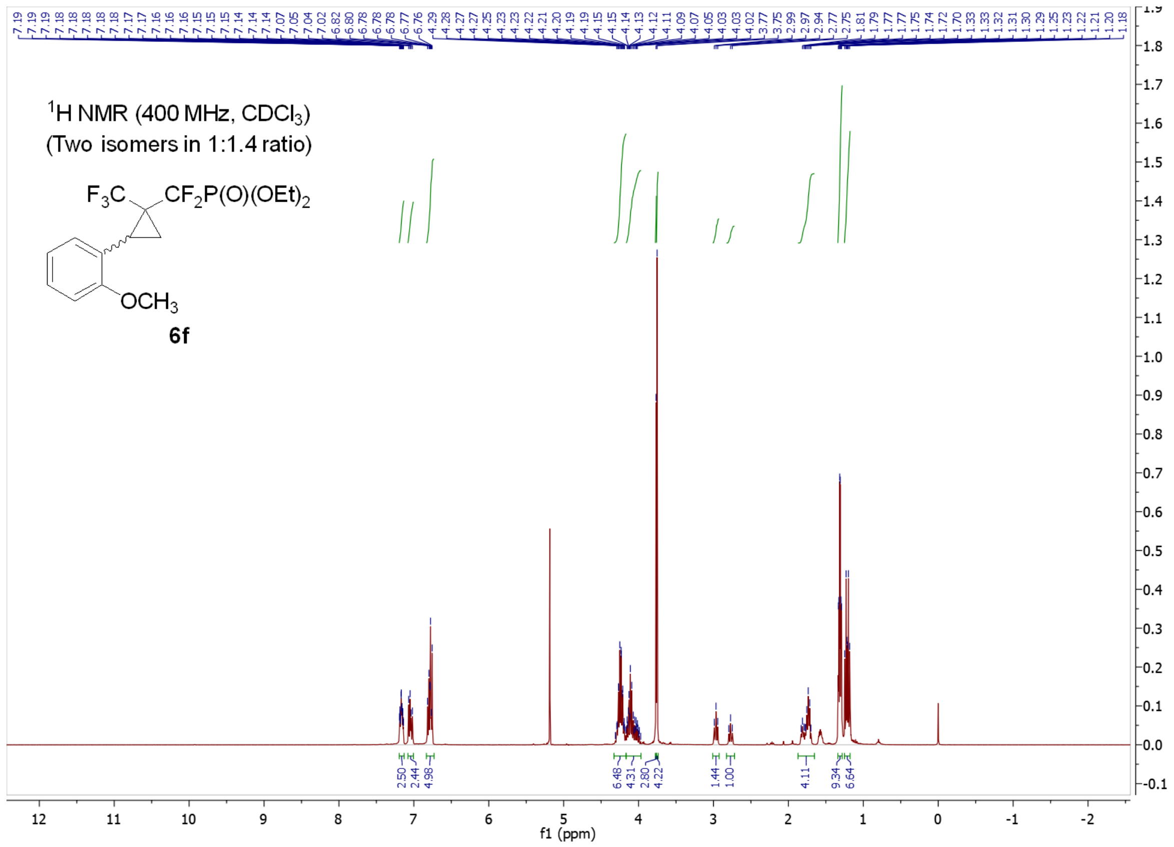

$^{1}$H NMR (400 MHz, CDCl$_3$)
(Two isomers in 1:1.4 ratio)
F$_3$C CF$_2$P(O)(OEt)$_2$
OCH$_3$
6f
f1 (ppm)


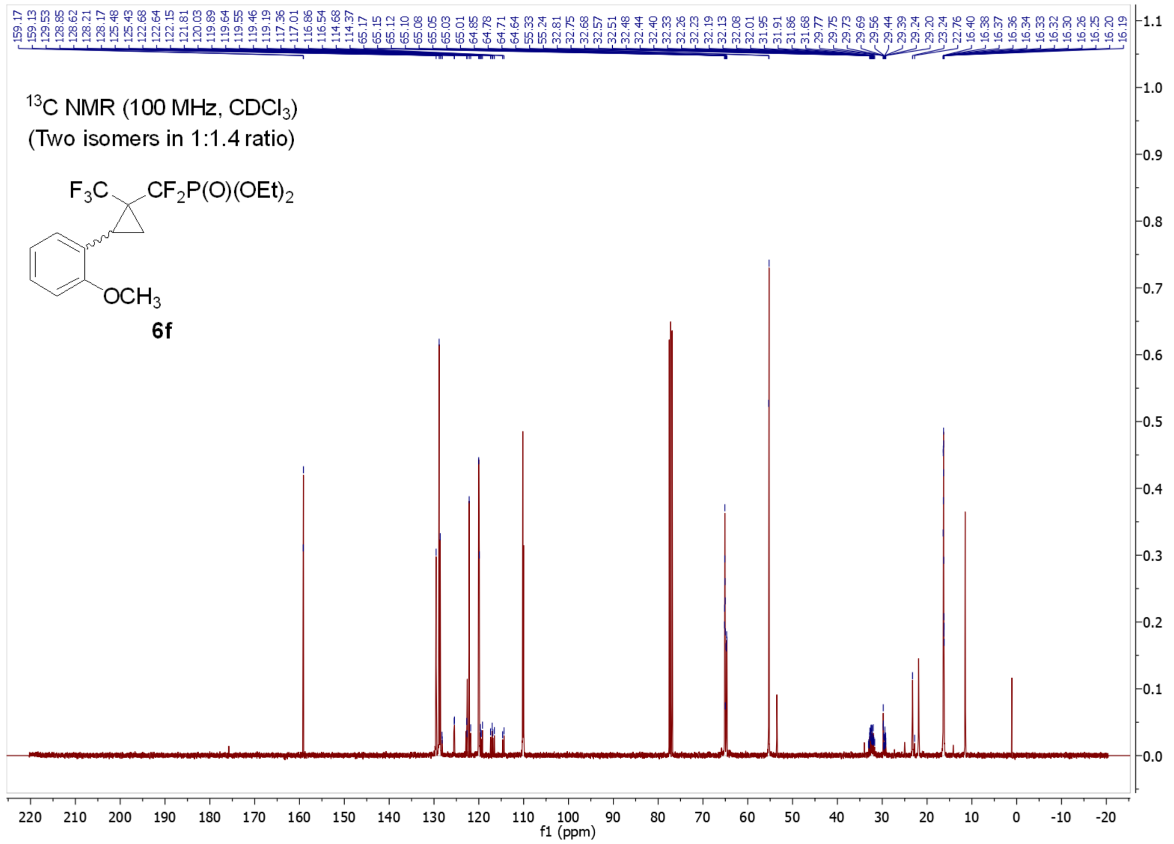

$^{13}$C NMR (100 MHz, CDCl$_3$)
(Two isomers in 1:1.4 ratio)
F$_3$C CF$_2$P(O)(OEt)$_2$
OCH$_3$
6f
f1 (ppm)

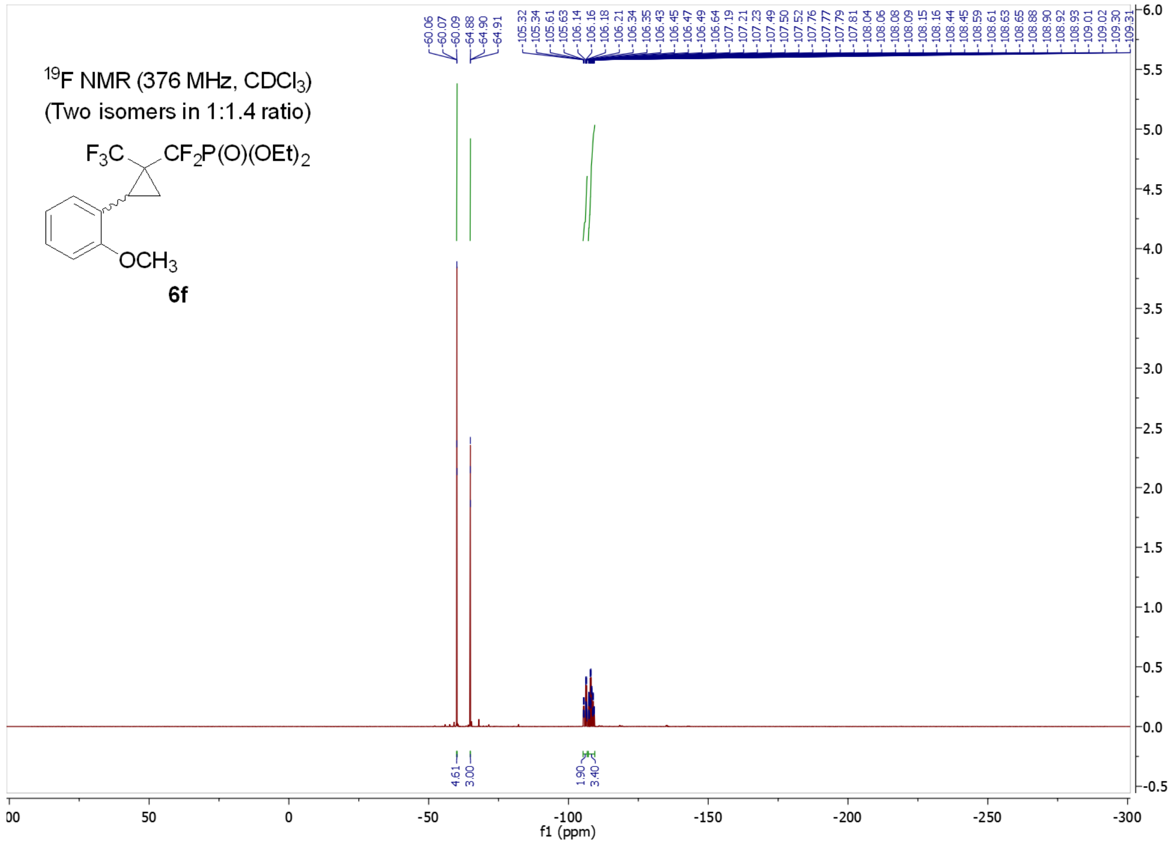

19F NMR (376 MHz, CDCl3)
(Two isomers in 1:1.4 ratio)
F3C
CF2P(O)(OEt)2
OCH3
6f
f1 (ppm)


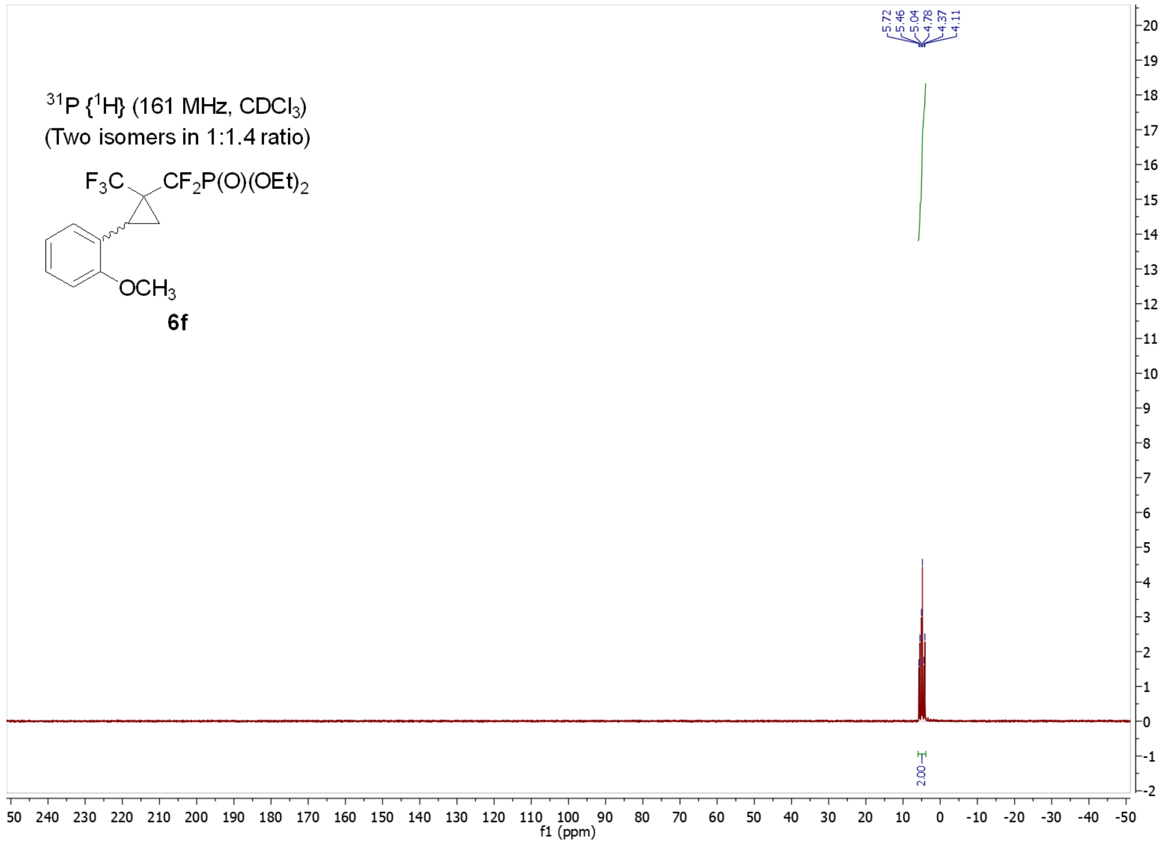

31P {1H} (161 MHz, CDCl3)
(Two isomers in 1:1.4 ratio)
F3C
CF2P(O)(OEt)2
OCH3
6f
f1 (ppm)

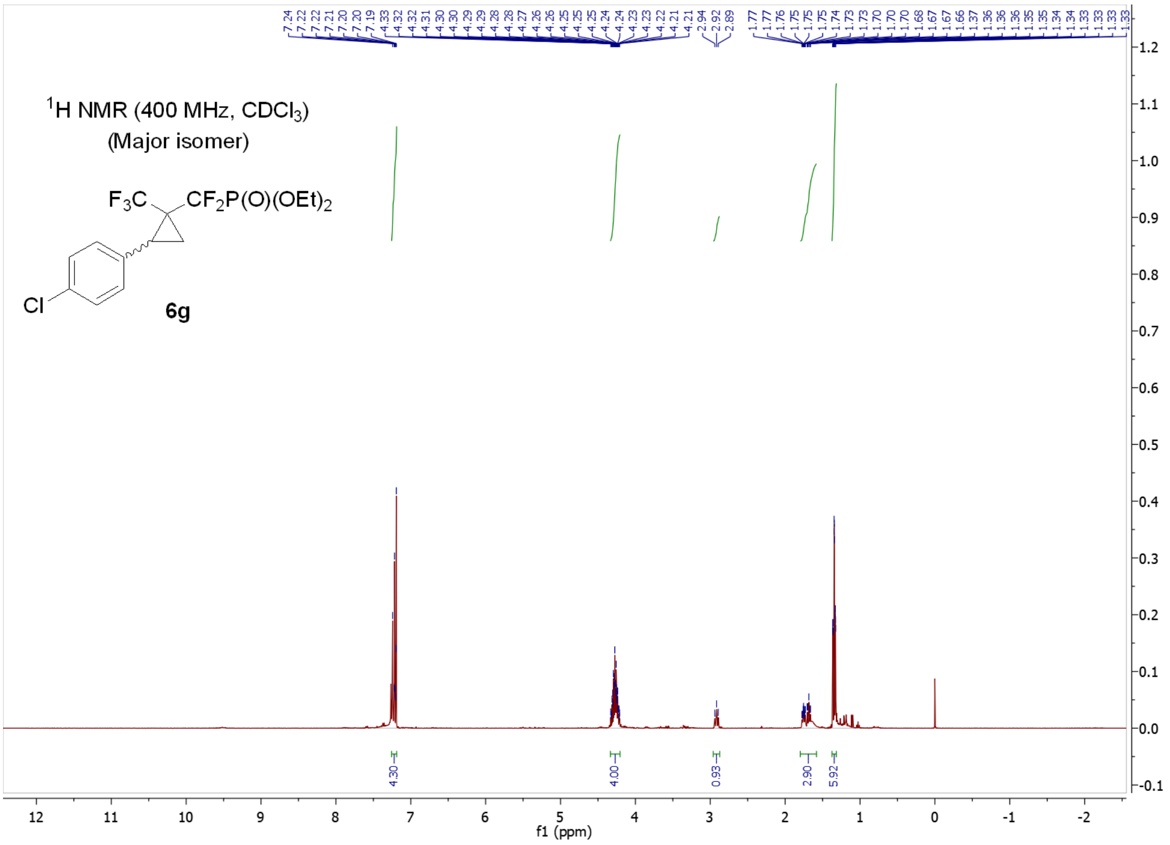
1H NMR (400 MHz, CDCl3)
(Major isomer)
F3C
CF2P(O)(OEt)2
Cl
6g
4.30
4.00
0.93
2.90
5.92
f1 (ppm)

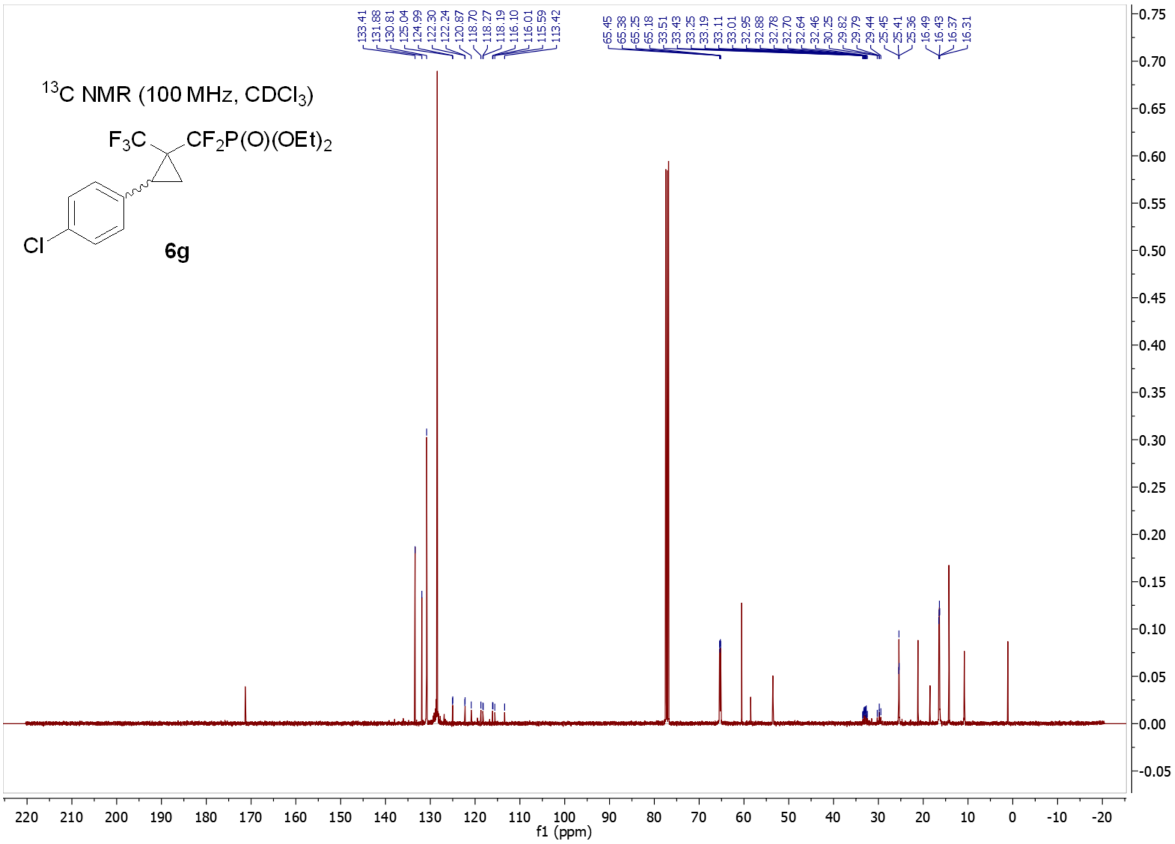
13C NMR (100 MHz, CDCl3)
F3C
CF2P(O)(OEt)2
Cl
6g
f1 (ppm)

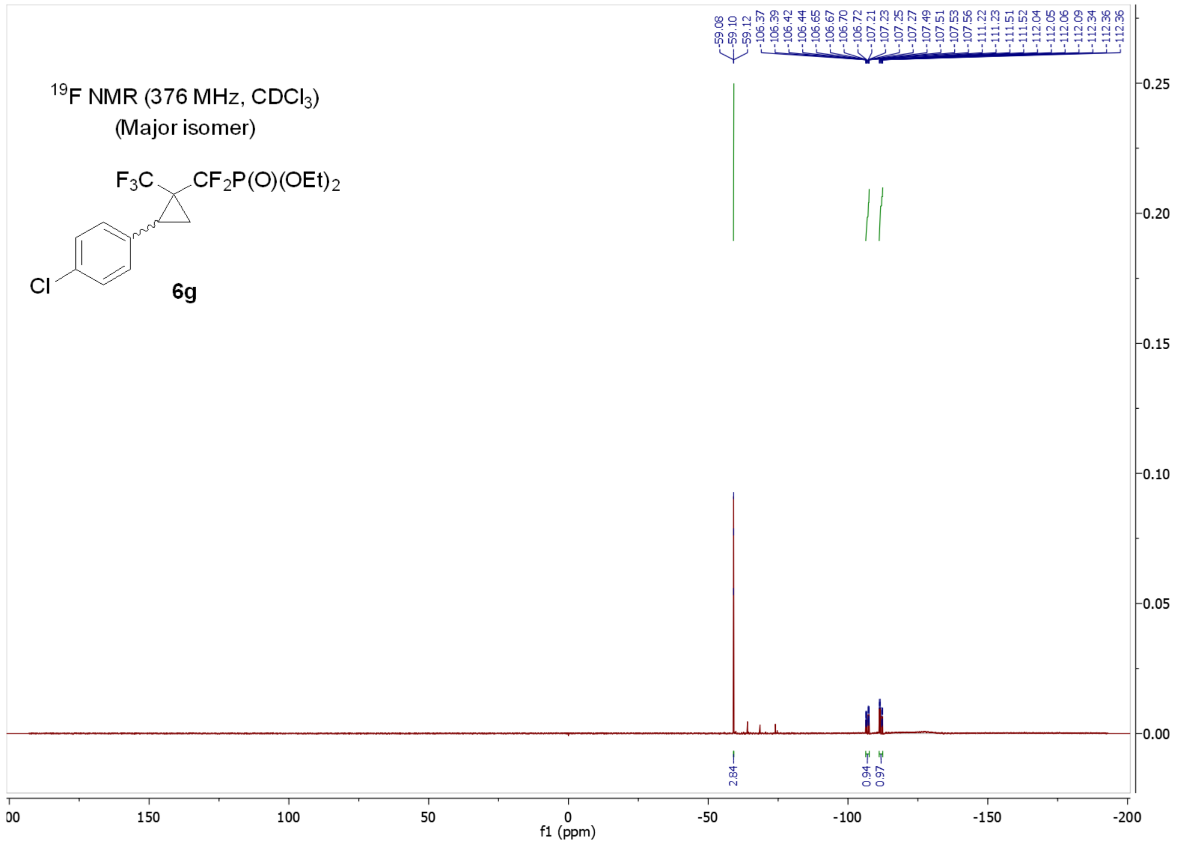
19F NMR (376 MHz, CDCl3)
(Major isomer)
F3C
CF2P(O)(OEt)2
Cl
6g
f1 (ppm)

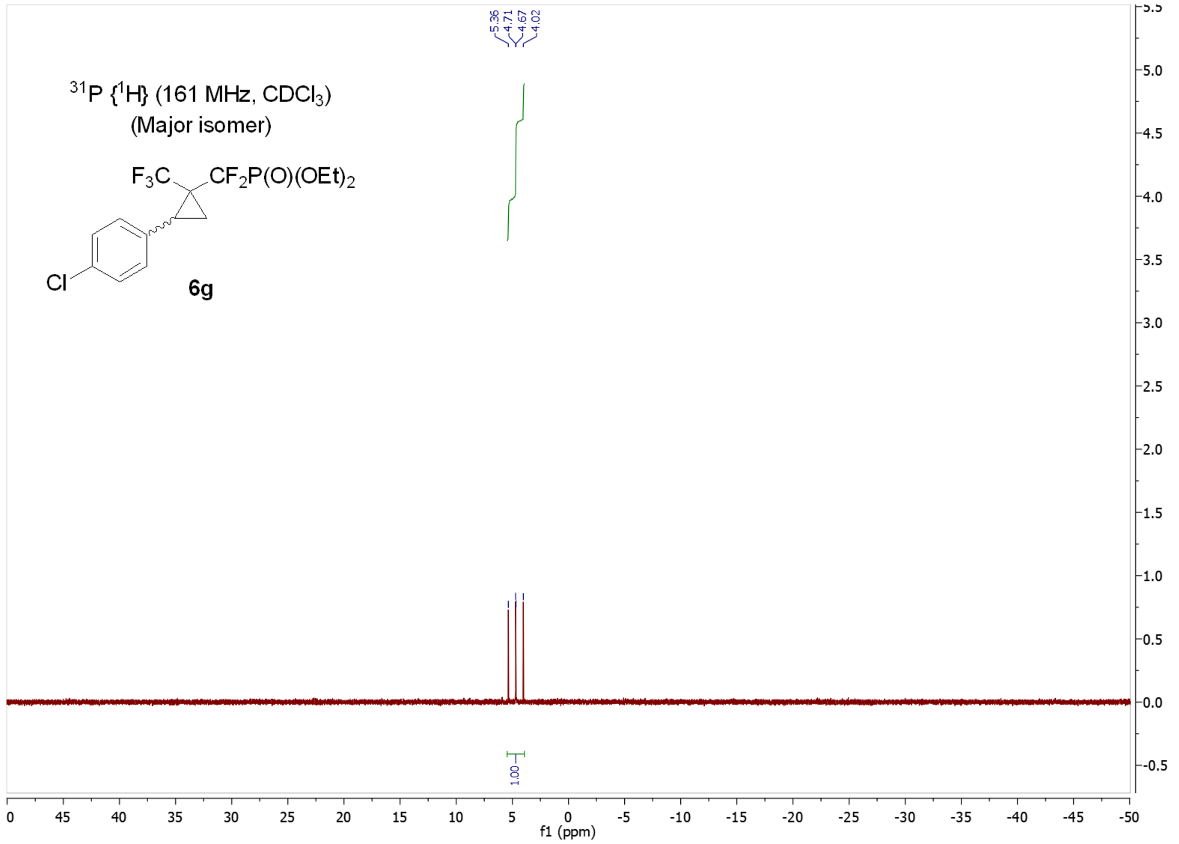
31P {1H} (161 MHz, CDCl3)
(Major isomer)
F3C
CF2P(O)(OEt)2
Cl
6g
f1 (ppm)

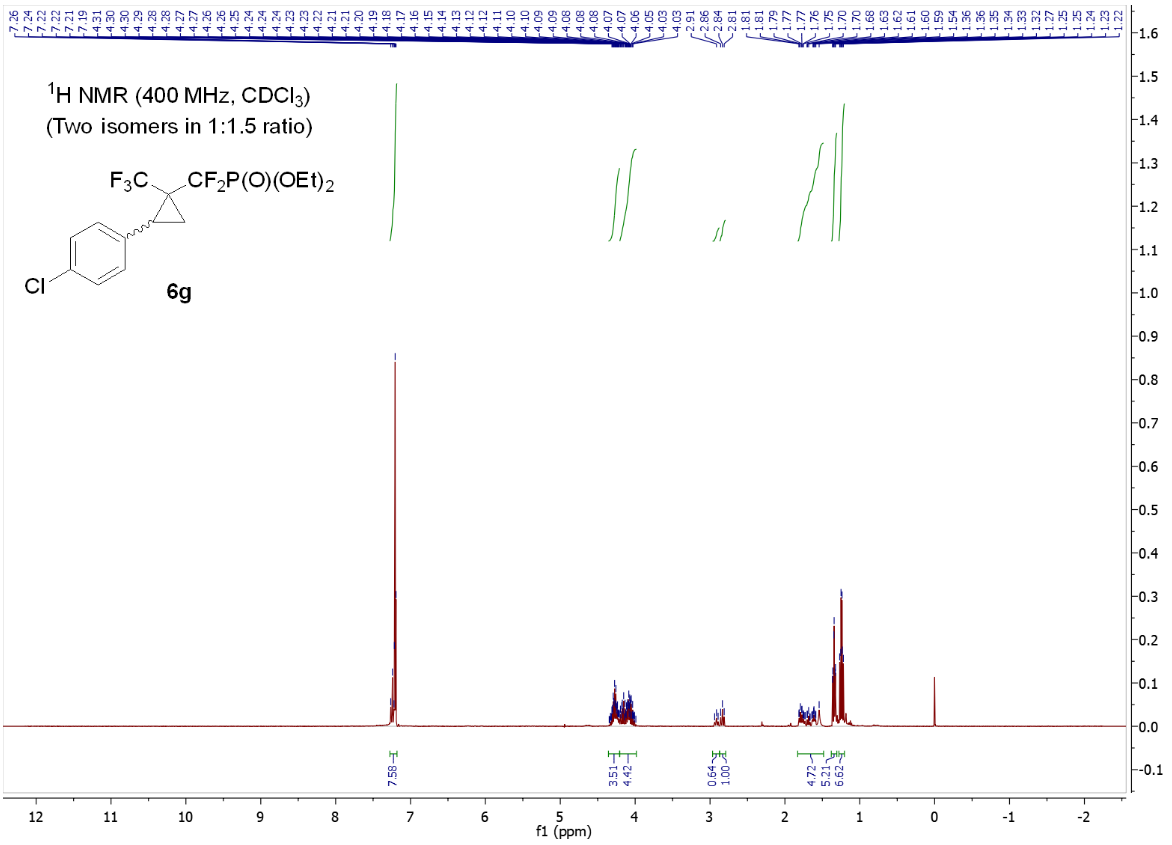
1H NMR (400 MHz, CDCl3)
(Two isomers in 1:1.5 ratio)
F3C
CF2P(O)(OEt)2
Cl
6g
f1 (ppm)

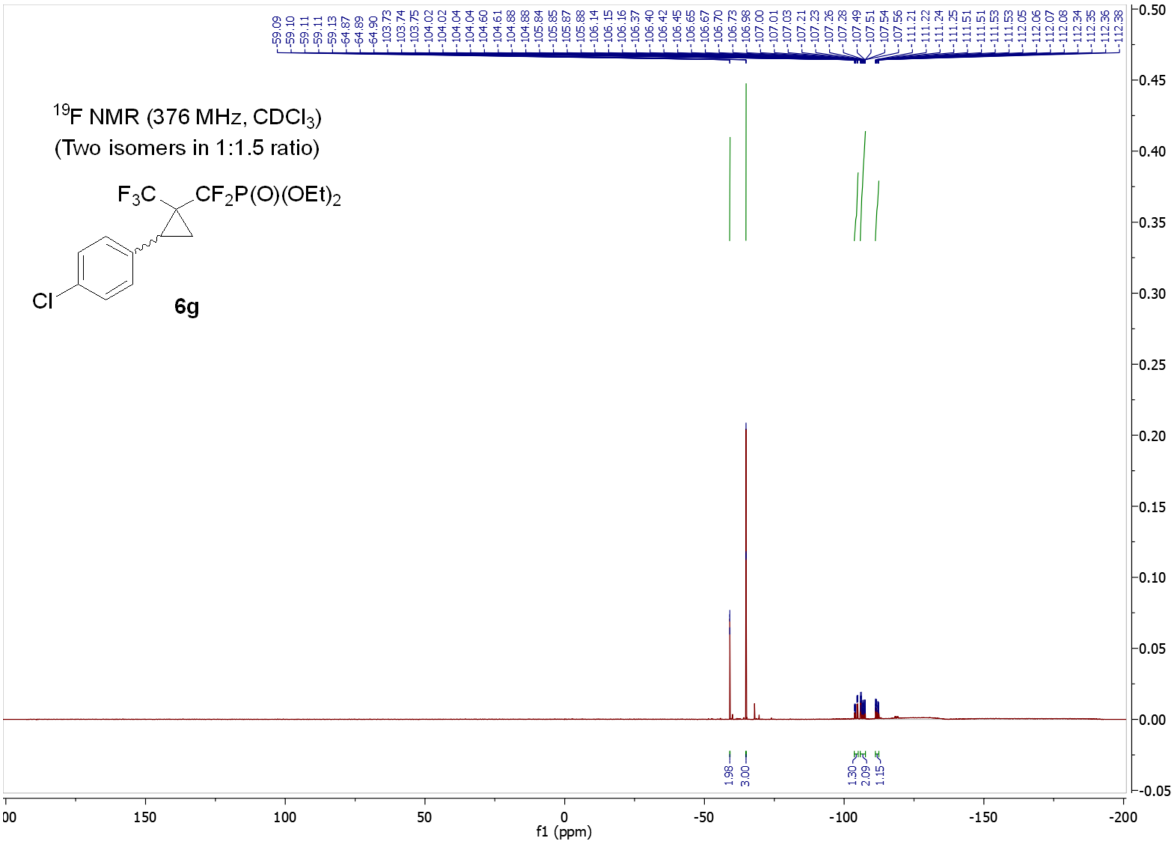
19F NMR (376 MHz, CDCl3)
(Two isomers in 1:1.5 ratio)
F3C
CF2P(O)(OEt)2
Cl
6g
f1 (ppm)

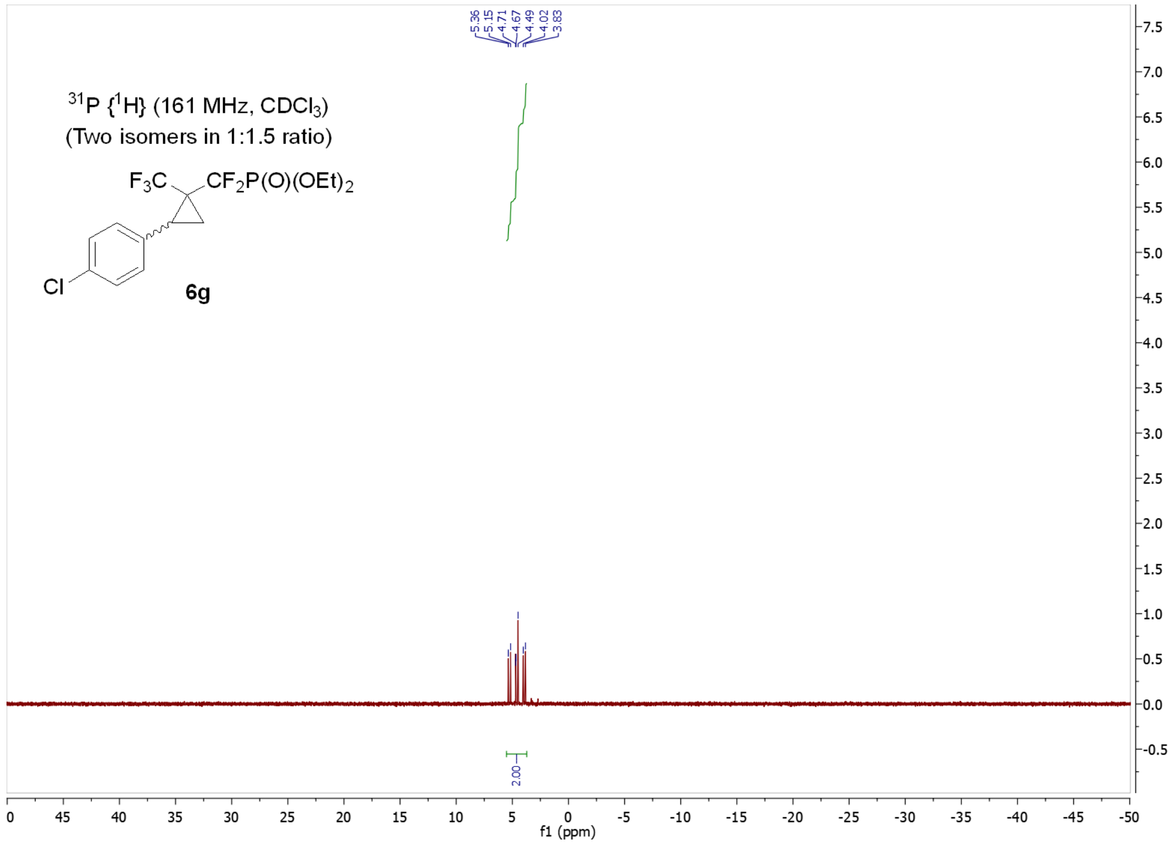

$^{31}P\ \{^{1}H\}$ (161 MHz, CDCl$_3$)
(Two isomers in 1:1.5 ratio)
F$_3$C
CF$_2$P(O)(OEt)$_2$
Cl
6g
f1 (ppm)


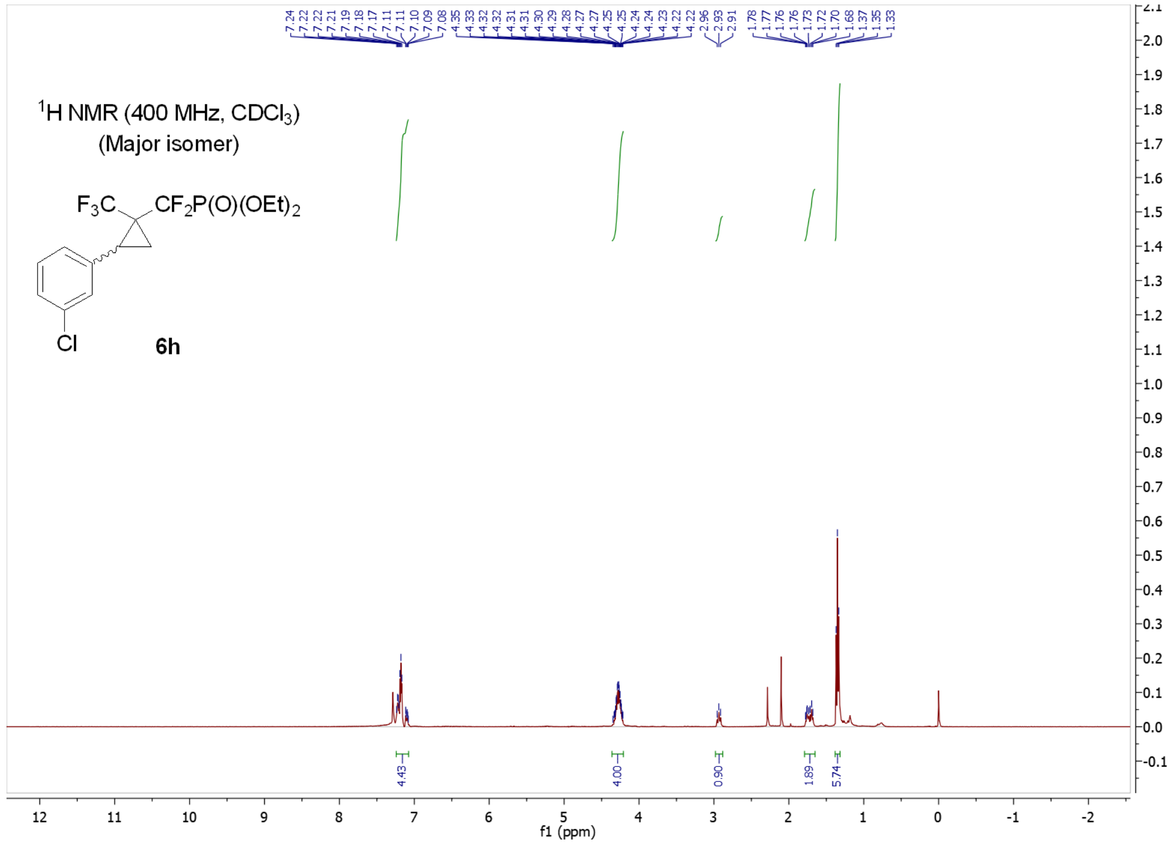

$^{1}H$ NMR (400 MHz, CDCl$_3$)
(Major isomer)
F$_3$C
CF$_2$P(O)(OEt)$_2$
Cl
6h
f1 (ppm)

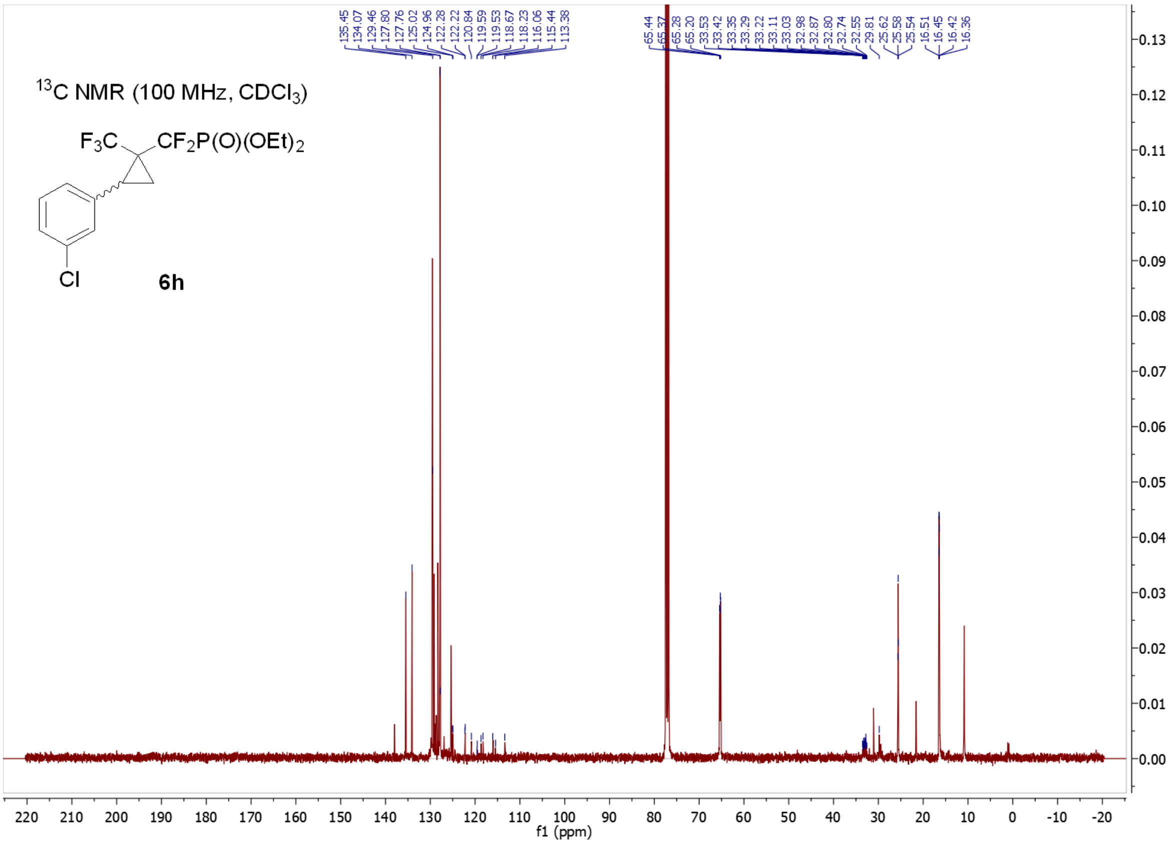

$^{13}C$ NMR (100 MHz, $CDCl_3$)
$F_3C$ $CF_2P(O)(OEt)_2$
Cl
6h
135.45 134.07 129.46 127.80 127.76 125.02 124.96 122.28 122.22 120.84 119.59 119.53 118.67 118.23 116.06 115.44 113.38
65.44 65.37 65.28 65.20 33.53 33.42 33.35 33.29 33.22 33.11 33.03 32.98 32.87 32.80 32.74 32.55 29.81 25.62 25.58 25.54 16.51 16.45 16.42 16.36
f1 (ppm)


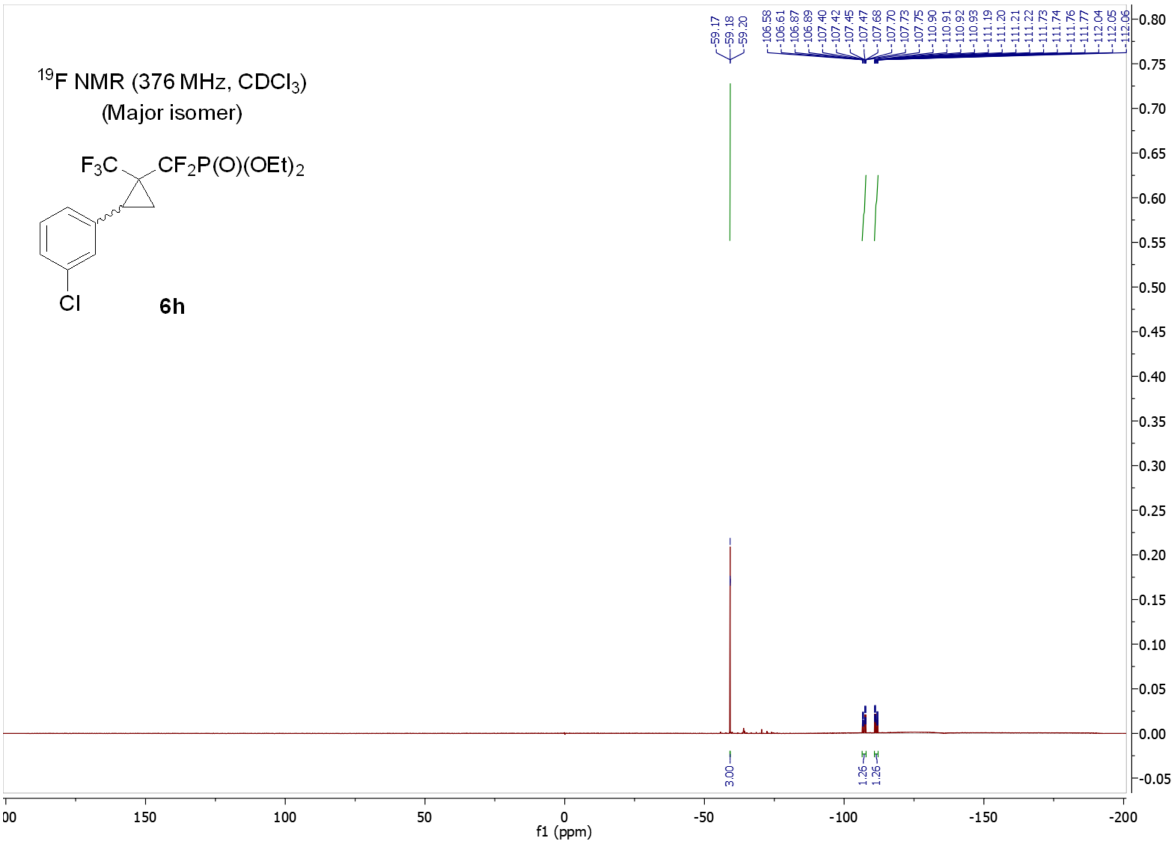

$^{19}F$ NMR (376 MHz, $CDCl_3$)
(Major isomer)
$F_3C$ $CF_2P(O)(OEt)_2$
Cl
6h
-59.17 -59.18 -59.20
-106.58 -106.61 -106.87 -106.89 -107.40 -107.42 -107.45 -107.47 -107.68 -107.70 -107.73 -107.75 -110.90 -110.91 -110.92 -110.93 -111.19 -111.20 -111.21 -111.22 -111.73 -111.74 -111.76 -111.77 -112.04 -112.05 -112.06
3.00
1.26 1.26
f1 (ppm)

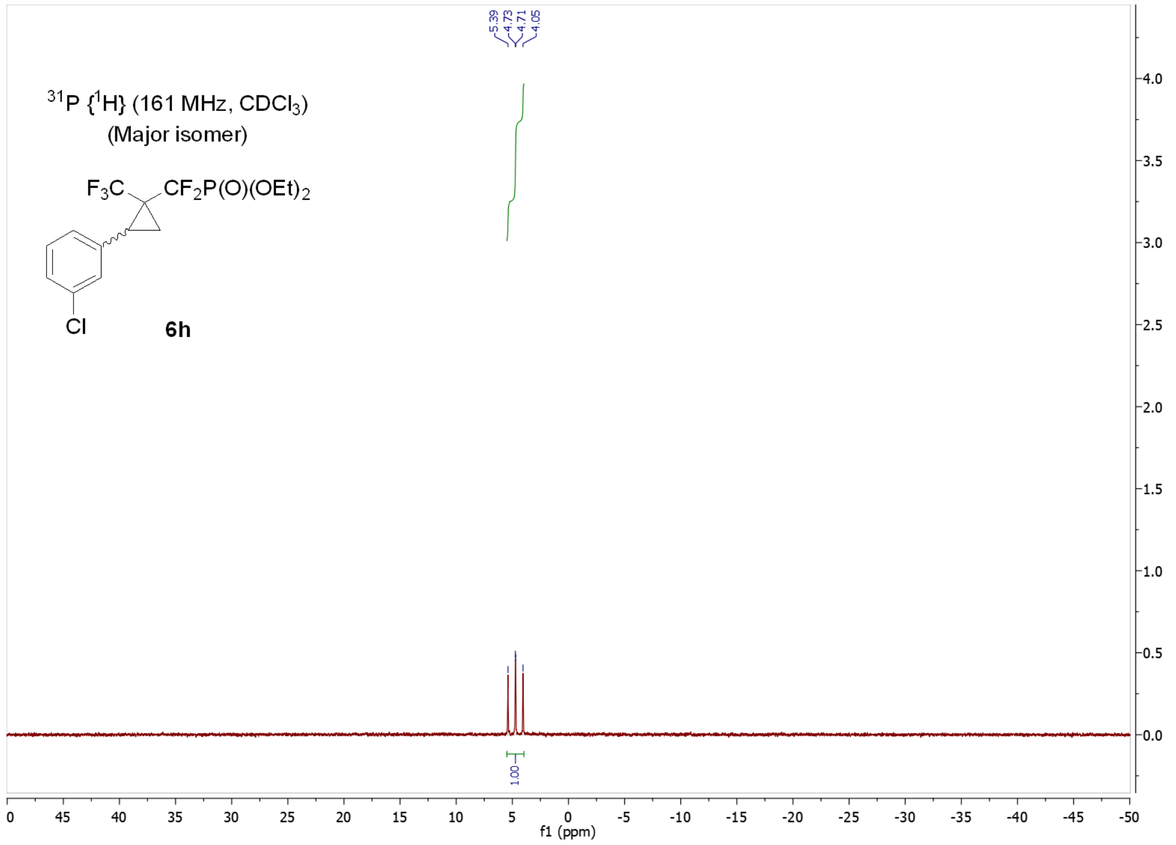
31P {1H} (161 MHz, CDCl3)
(Major isomer)
F3C
CF2P(O)(OEt)2
Cl
6h
5.39
4.73
4.71
4.05
1.00
f1 (ppm)

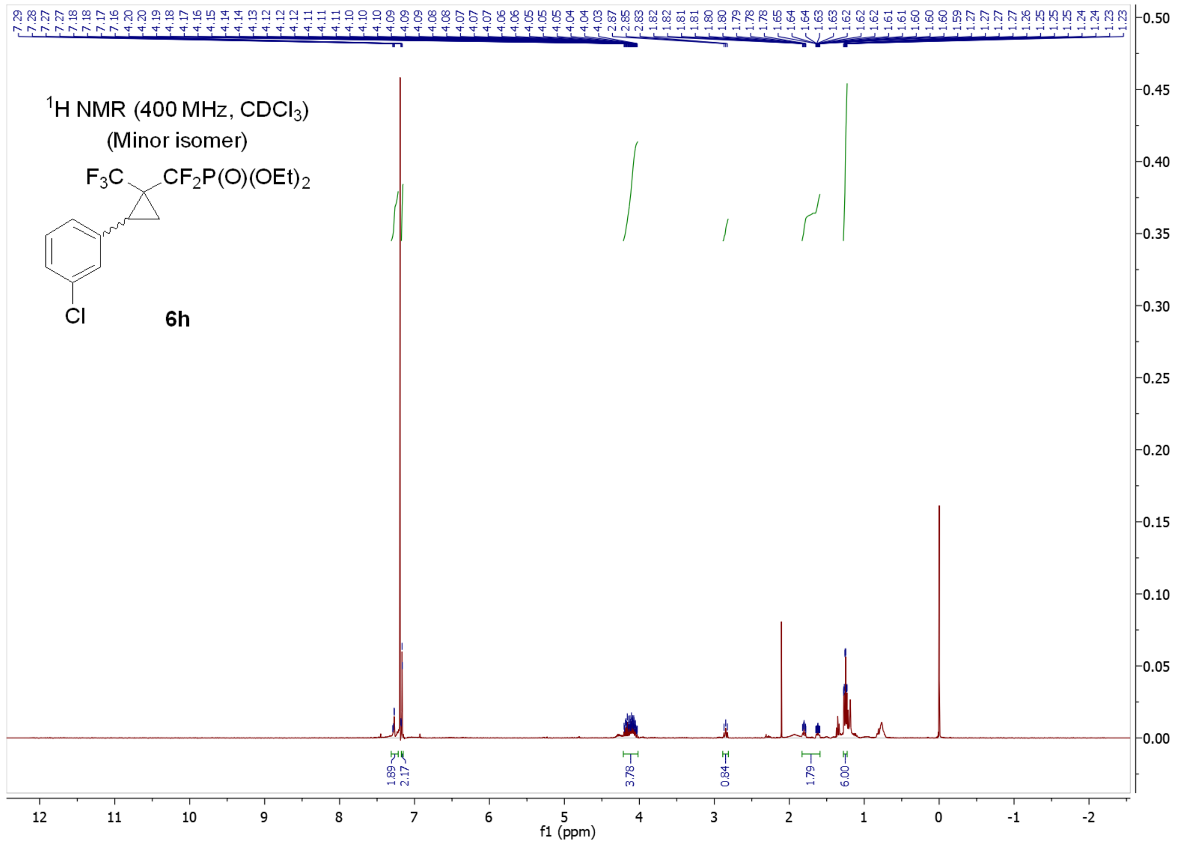
1H NMR (400 MHz, CDCl3)
(Minor isomer)
F3C
CF2P(O)(OEt)2
Cl
6h
1.89
2.17
3.78
0.84
1.79
6.00
f1 (ppm)

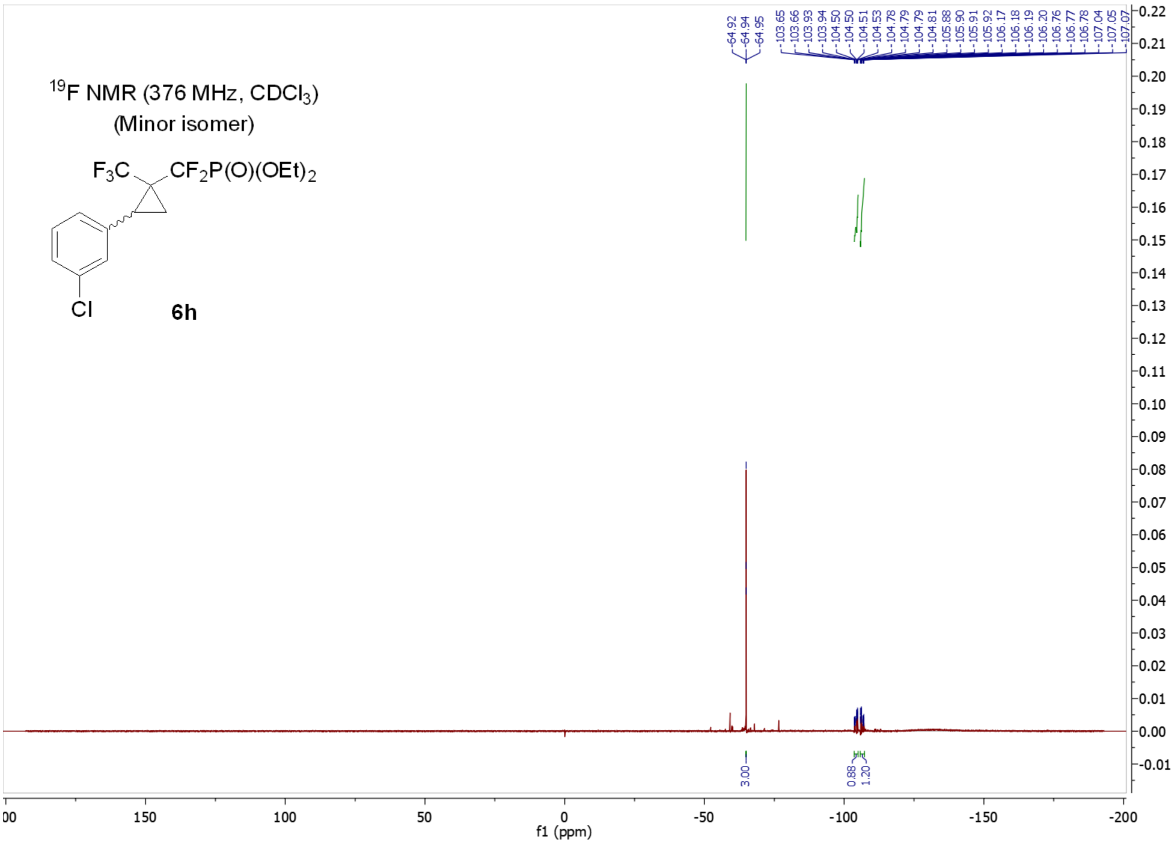
$^{19}$F NMR (376 MHz, CDCl$_3$)
(Minor isomer)
F$_3$C
CF$_2$P(O)(OEt)$_2$
Cl
6h
f1 (ppm)

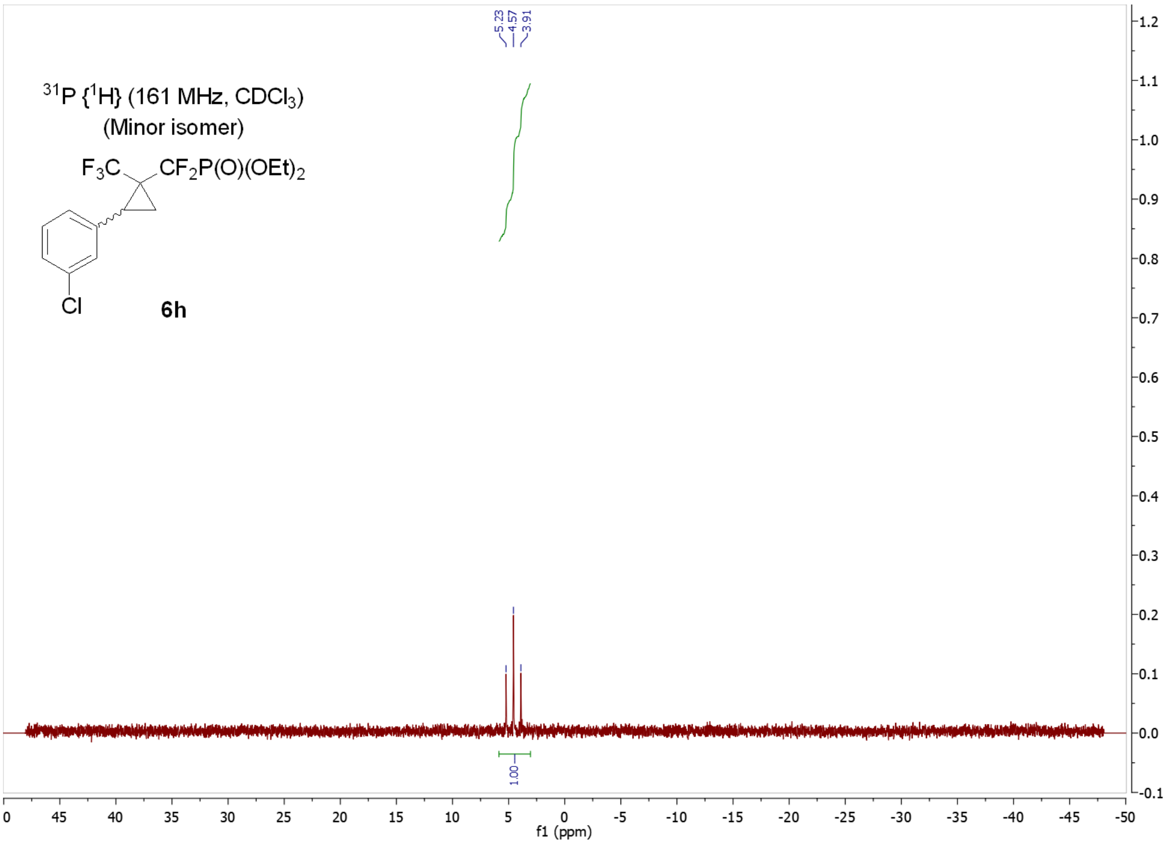
$^{31}$P {$^1$H} (161 MHz, CDCl$_3$)
(Minor isomer)
F$_3$C
CF$_2$P(O)(OEt)$_2$
Cl
6h
5.23
4.57
3.91
1.00
f1 (ppm)

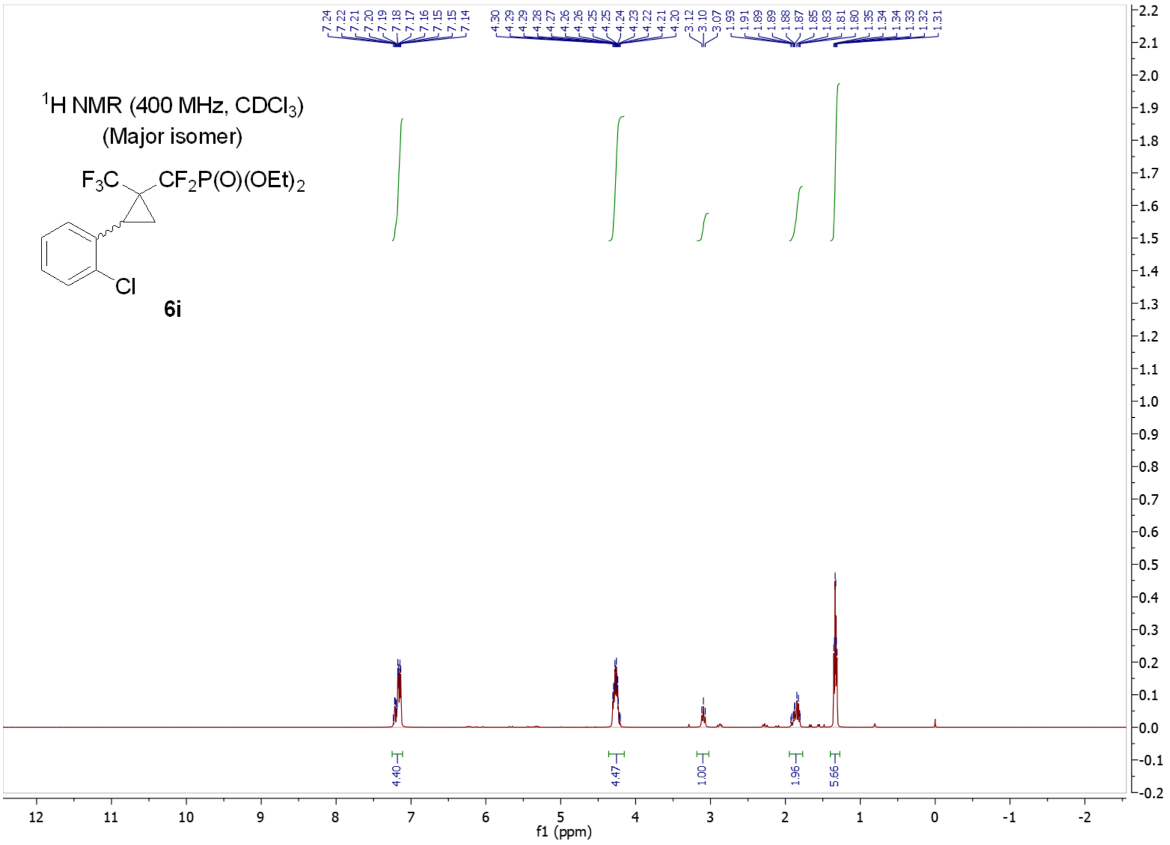
$^{1}$H NMR (400 MHz, $CDCl_3$)
(Major isomer)
$F_3C$
$CF_2P(O)(OEt)_2$
Cl
6i
4.40
4.47
1.00
1.96
5.66
f1 (ppm)

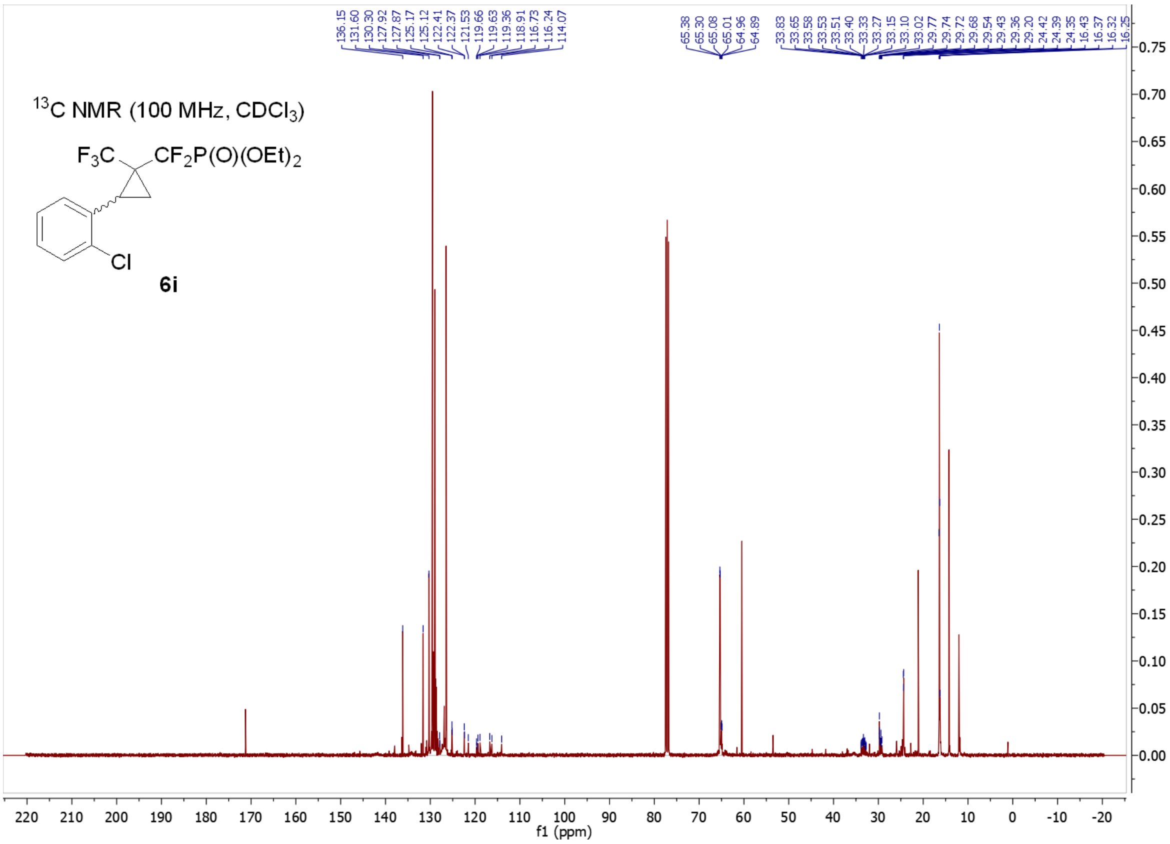
$^{13}$C NMR (100 MHz, $CDCl_3$)
$F_3C$
$CF_2P(O)(OEt)_2$
Cl
6i
f1 (ppm)

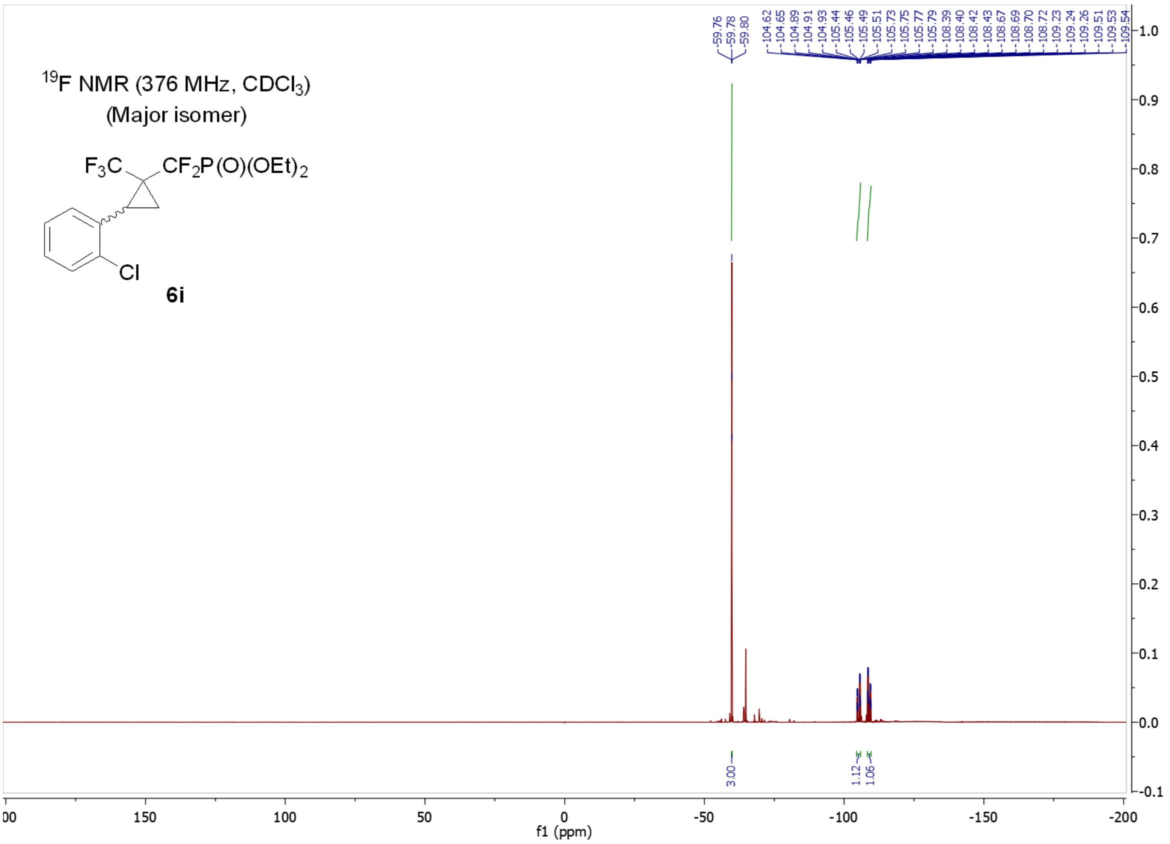

¹⁹F NMR (376 MHz, CDCl₃)
(Major isomer)
F₃C
CF₂P(O)(OEt)₂
Cl
6i
f1 (ppm)


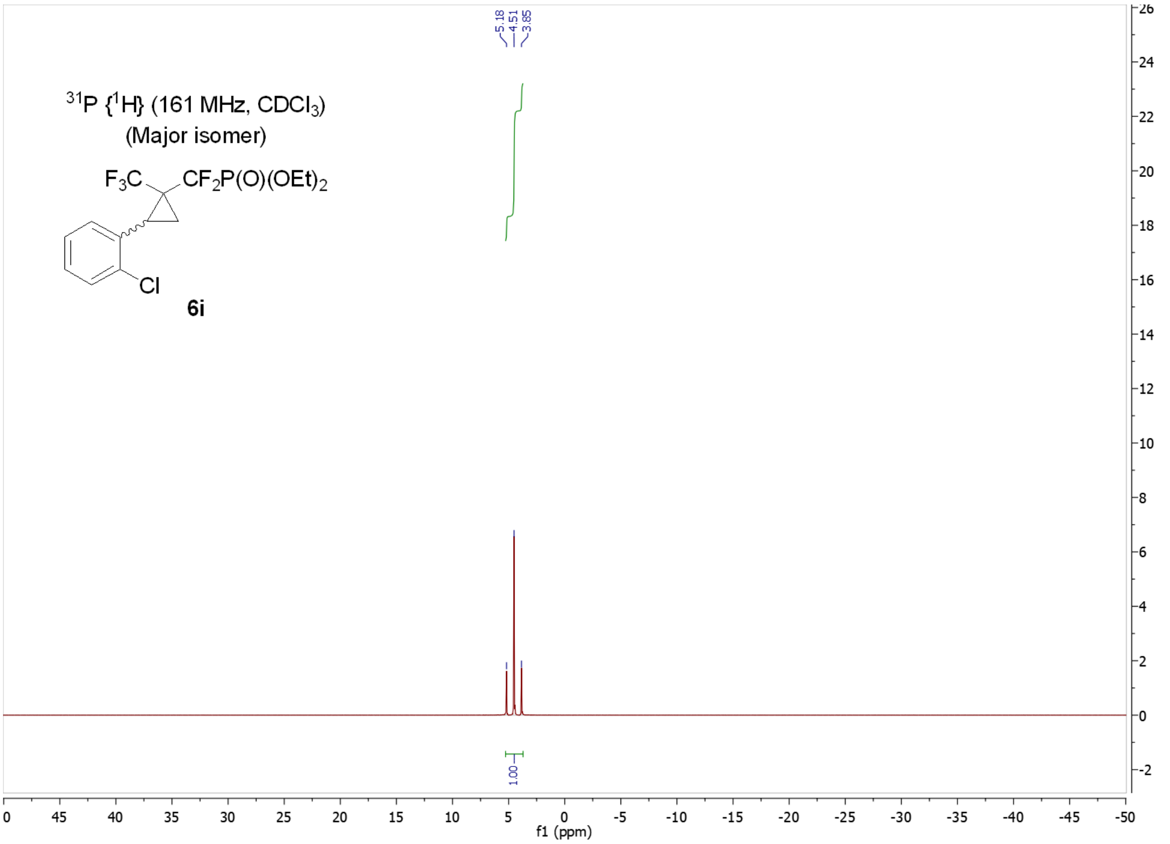

³¹P {¹H} (161 MHz, CDCl₃)
(Major isomer)
F₃C
CF₂P(O)(OEt)₂
Cl
6i
5.18
4.51
3.85
1.00
f1 (ppm)

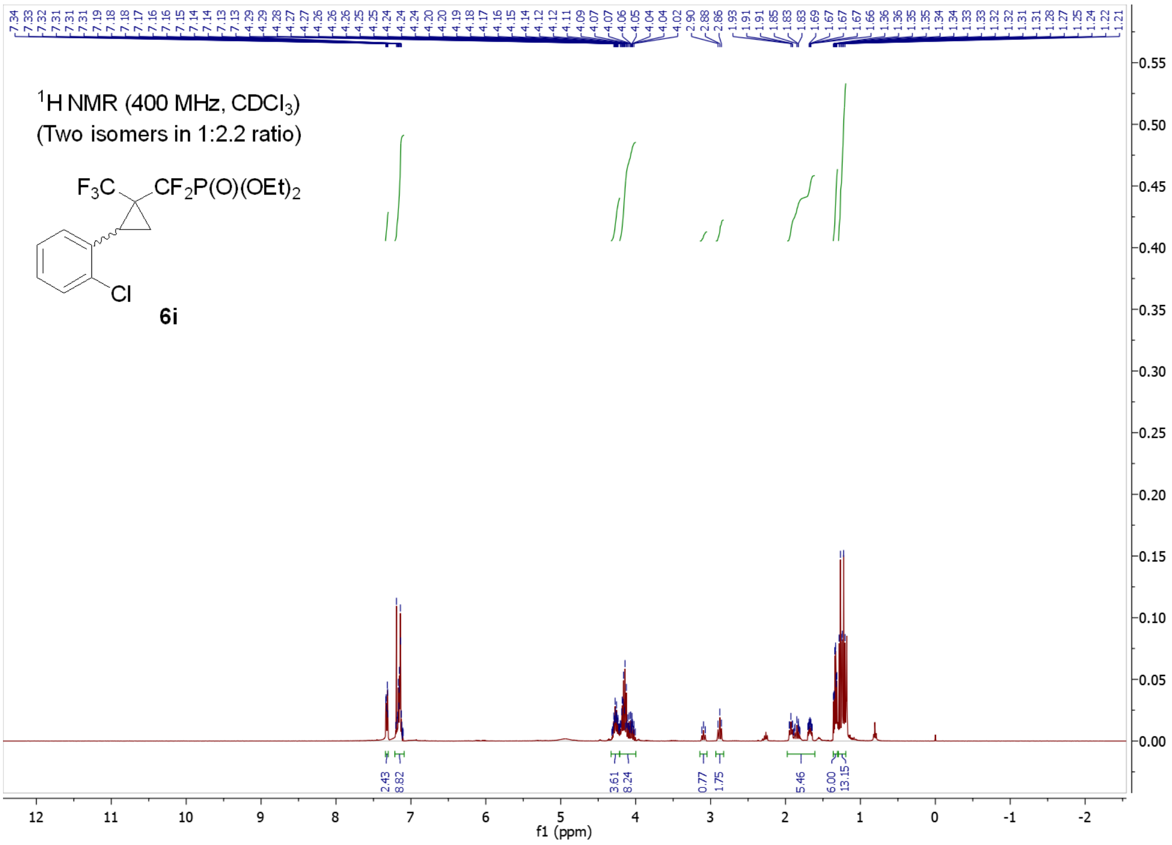
$^{1}$H NMR (400 MHz, CDCl$_3$)
(Two isomers in 1:2.2 ratio)
F$_3$C
CF$_2$P(O)(OEt)$_2$
Cl
6i
f1 (ppm)

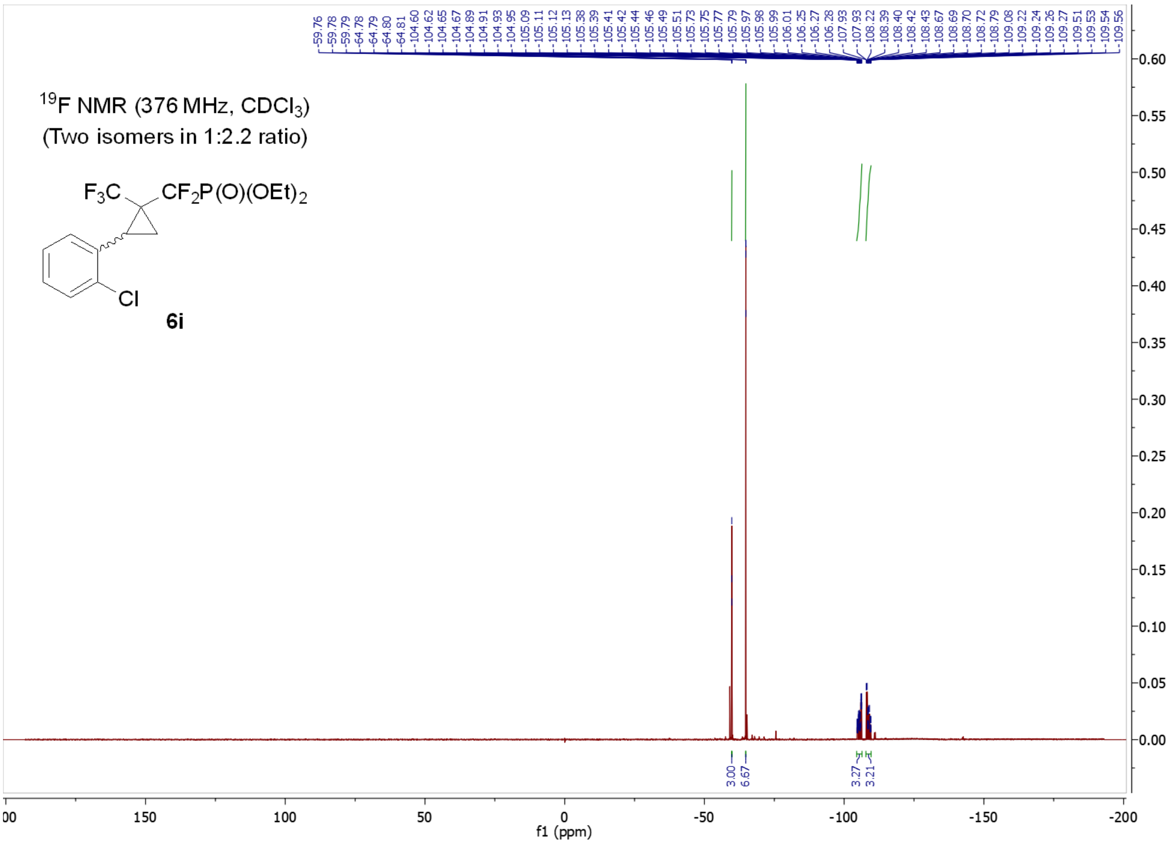
$^{19}$F NMR (376 MHz, CDCl$_3$)
(Two isomers in 1:2.2 ratio)
F$_3$C
CF$_2$P(O)(OEt)$_2$
Cl
6i
f1 (ppm)

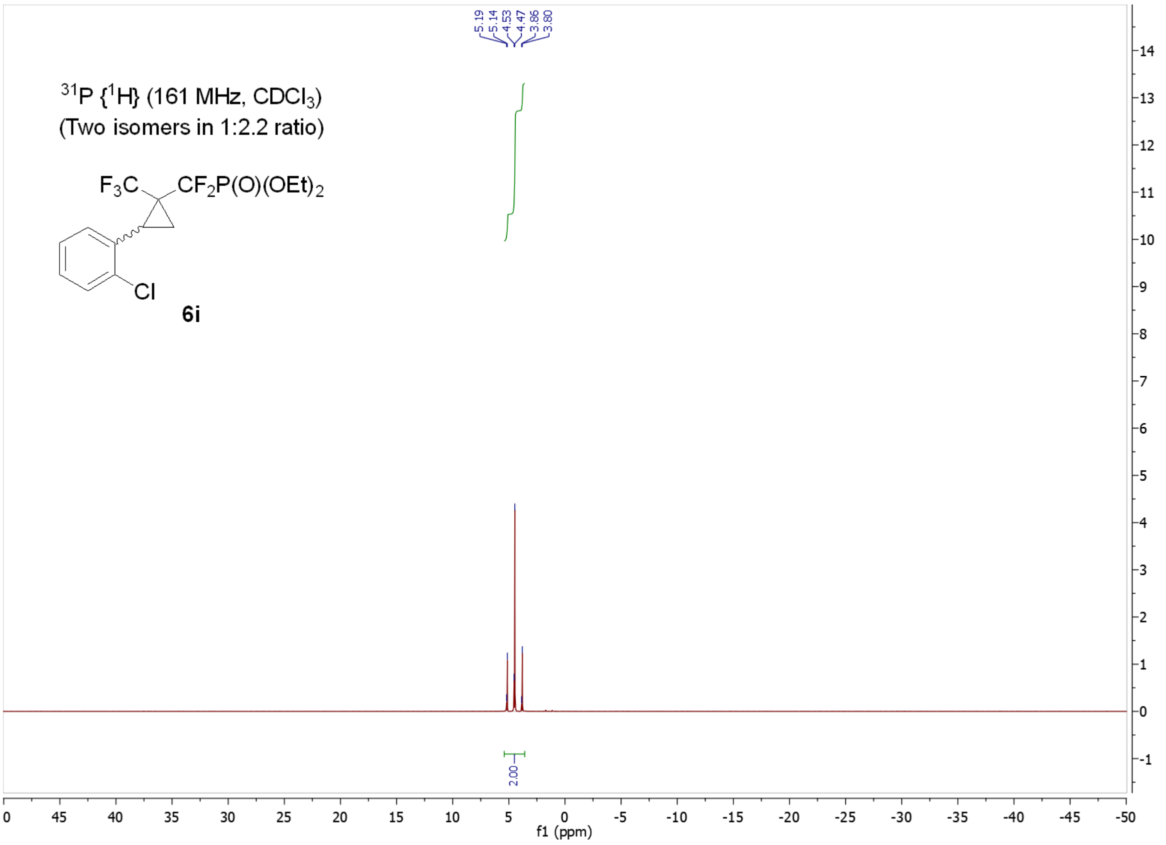

$^{31}P\ \{^1H\}$ (161 MHz, $CDCl_3$)
(Two isomers in 1:2.2 ratio)
$F_3C$ $CF_2P(O)(OEt)_2$
Cl
6i
5.19 5.14 4.53 4.47 3.86 3.80
2.00
f1 (ppm)


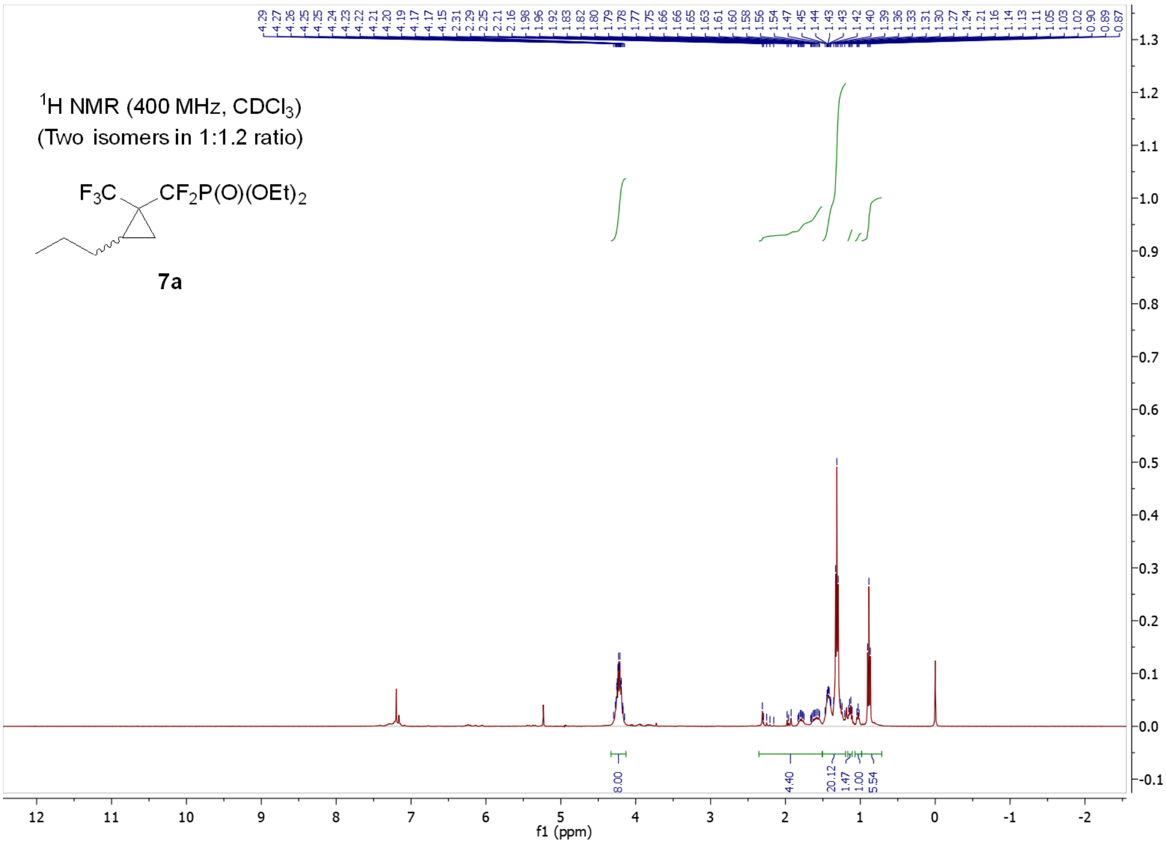

$^1H$ NMR (400 MHz, $CDCl_3$)
(Two isomers in 1:1.2 ratio)
$F_3C$ $CF_2P(O)(OEt)_2$
7a
4.29 4.27 4.26 4.25 4.24 4.23 4.22 4.21 4.20 4.19 4.17 4.15 2.31 2.29 2.25 2.21 2.16 1.98 1.96 1.92 1.83 1.82 1.80 1.79 1.78 1.77 1.75 1.66 1.65 1.63 1.61 1.60 1.58 1.56 1.54 1.47 1.45 1.44 1.43 1.43 1.42 1.40 1.39 1.36 1.33 1.31 1.30 1.27 1.24 1.21 1.16 1.14 1.13 1.11 1.05 1.03 1.02 0.90 0.89 0.87
8.00 4.40 20.12 1.47 1.00 5.54
f1 (ppm)

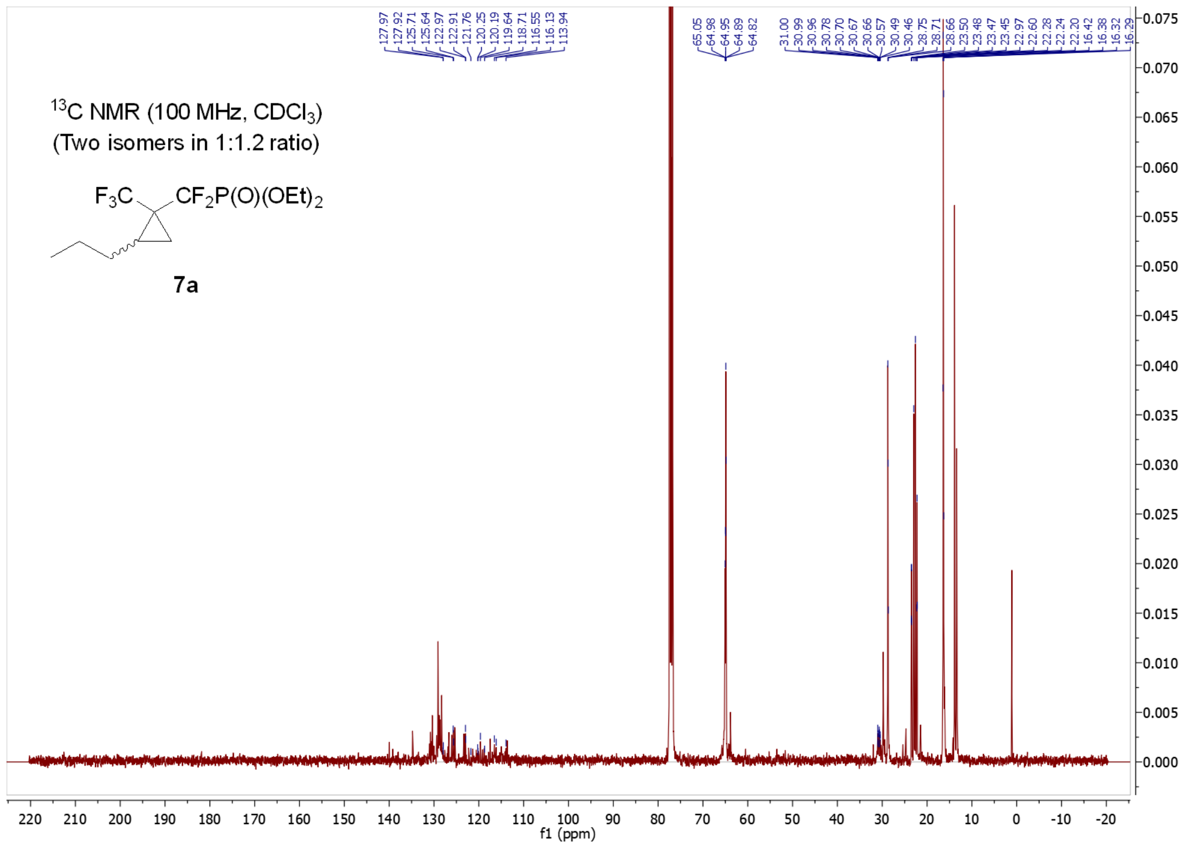

$^{13}C$ NMR (100 MHz, $CDCl_3$)
(Two isomers in 1:1.2 ratio)
$F_3C$
$CF_2P(O)(OEt)_2$
7a
f1 (ppm)


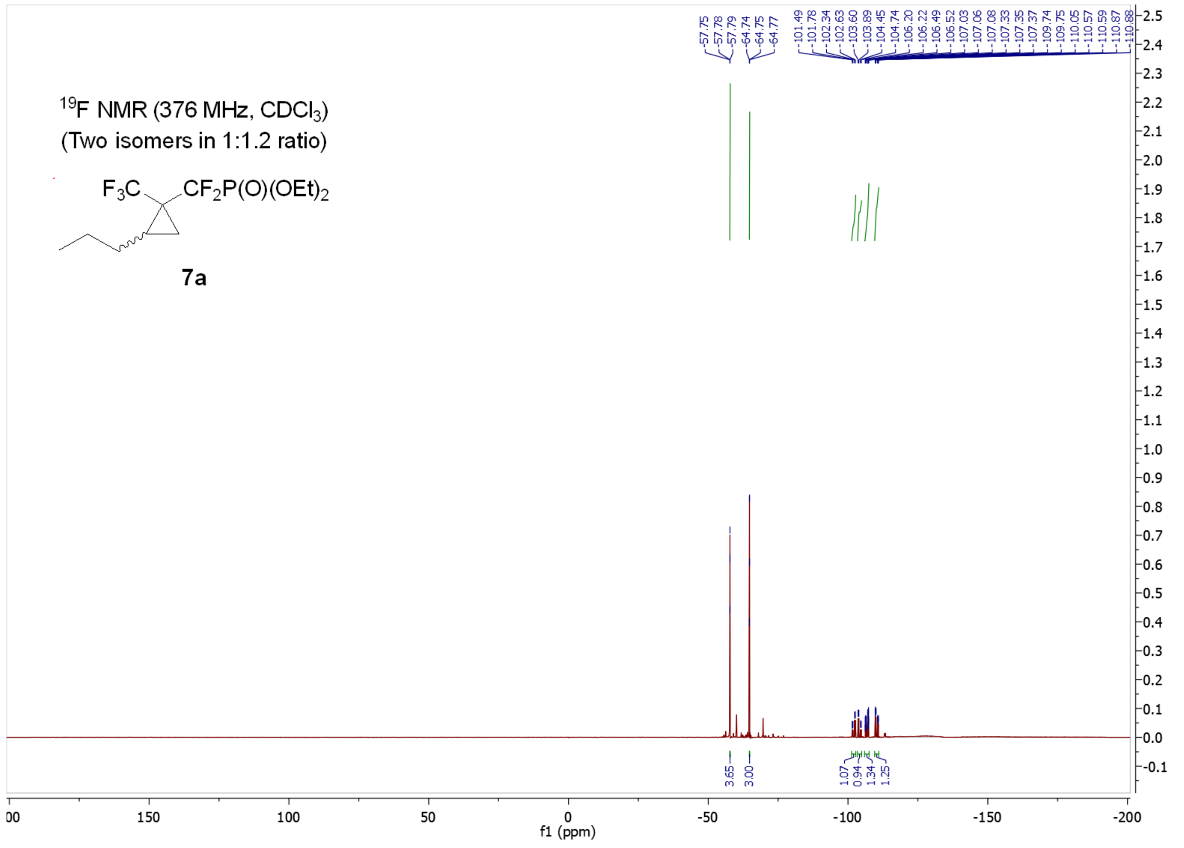

$^{19}F$ NMR (376 MHz, $CDCl_3$)
(Two isomers in 1:1.2 ratio)
$F_3C$
$CF_2P(O)(OEt)_2$
7a
f1 (ppm)

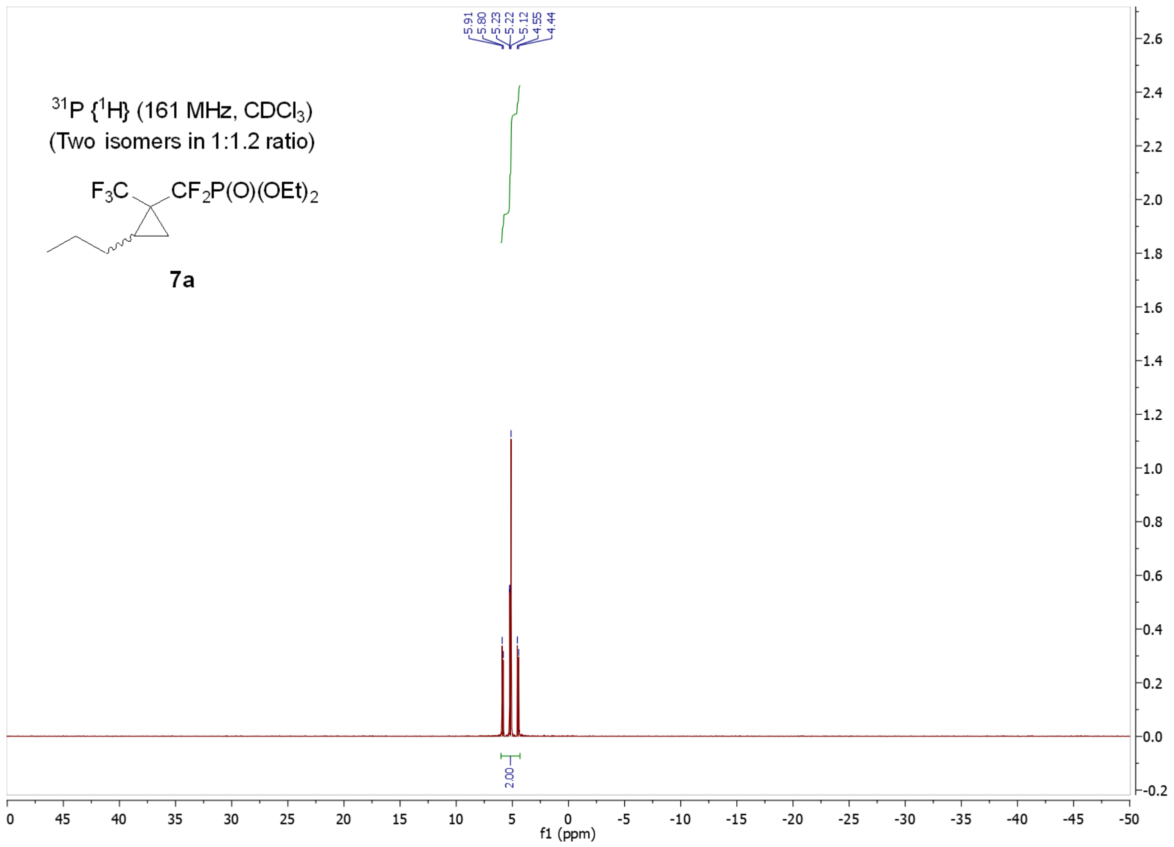
$^{31}P$ {$^{1}H$} (161 MHz, $CDCl_3$)
(Two isomers in 1:1.2 ratio)
$F_3C$
$CF_2P(O)(OEt)_2$
7a
f1 (ppm)

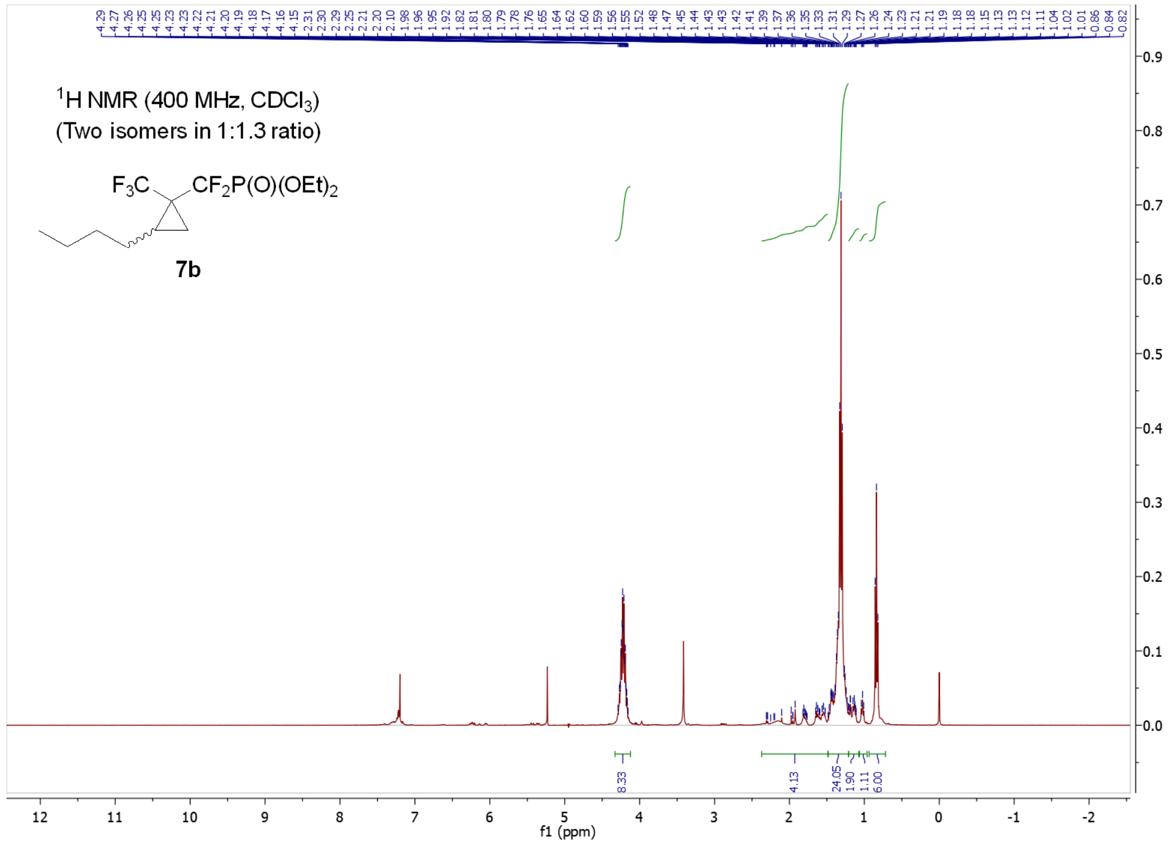
$^{1}H$ NMR (400 MHz, $CDCl_3$)
(Two isomers in 1:1.3 ratio)
$F_3C$
$CF_2P(O)(OEt)_2$
7b
f1 (ppm)

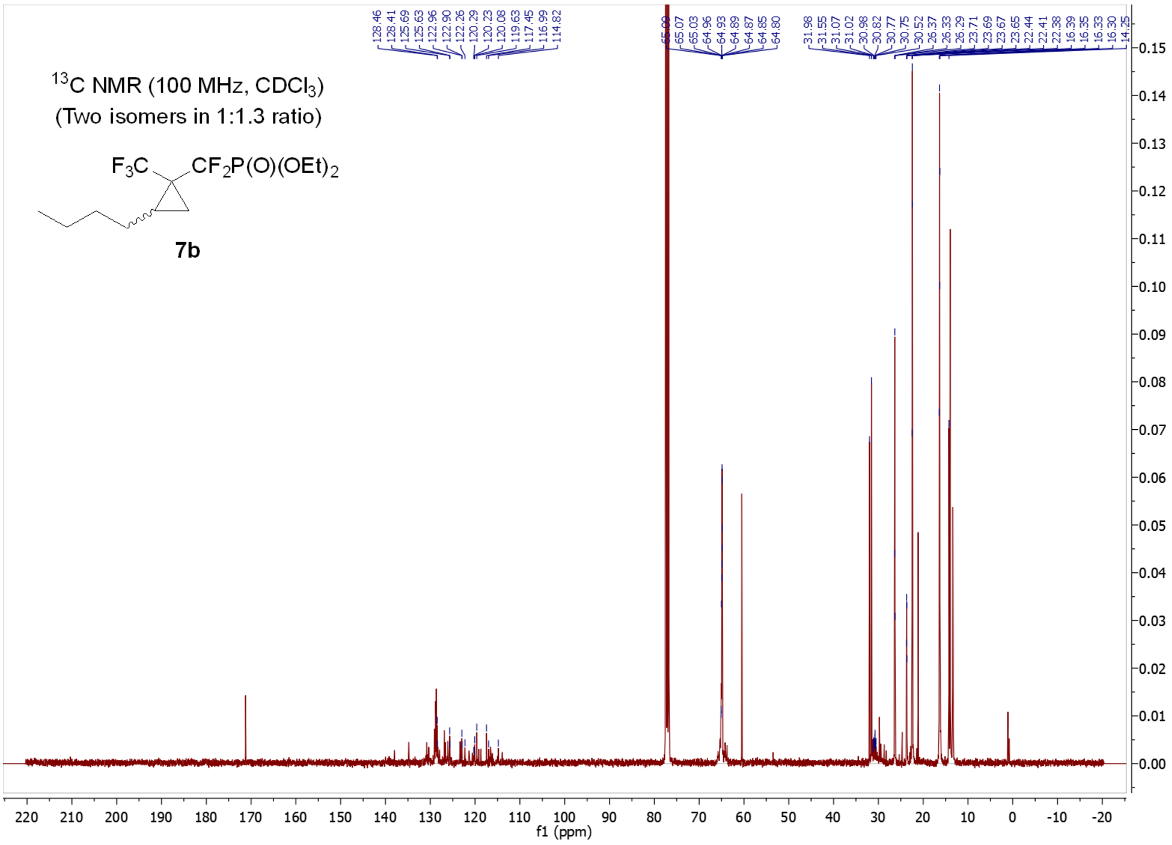

$^{13}$C NMR (100 MHz, CDCl$_3$)
(Two isomers in 1:1.3 ratio)
F$_3$C
CF$_2$P(O)(OEt)$_2$
7b
f1 (ppm)


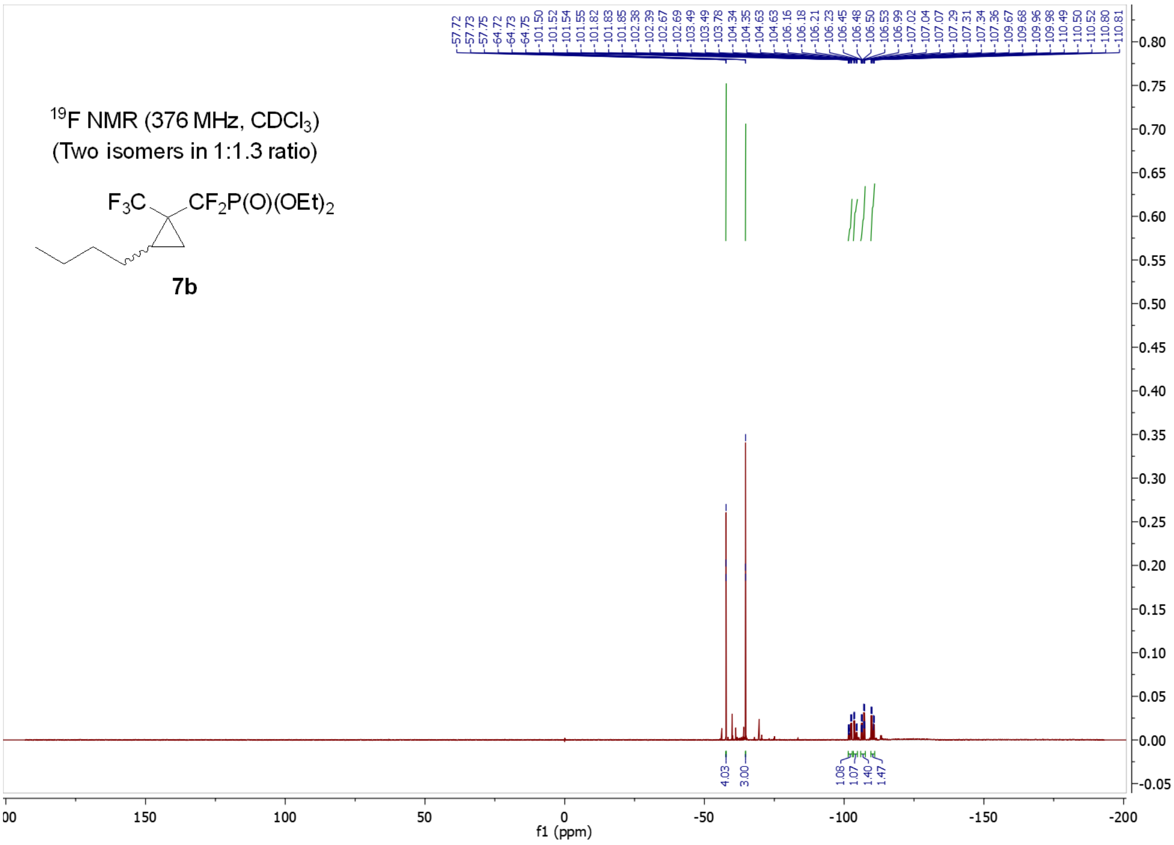

$^{19}$F NMR (376 MHz, CDCl$_3$)
(Two isomers in 1:1.3 ratio)
F$_3$C
CF$_2$P(O)(OEt)$_2$
7b
f1 (ppm)

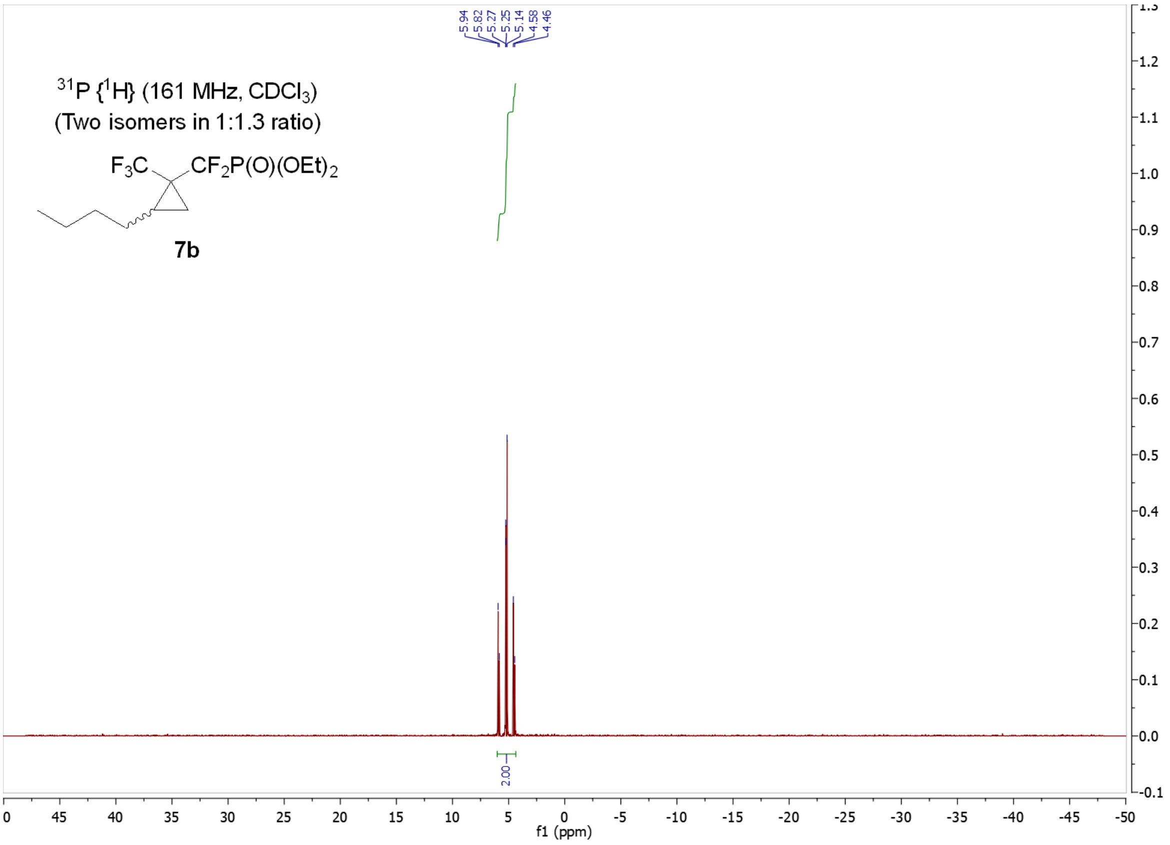
$^{31}$P {$^{1}$H} (161 MHz, CDCl$_3$)
(Two isomers in 1:1.3 ratio)
F$_3$C
CF$_2$P(O)(OEt)$_2$
7b
f1 (ppm)

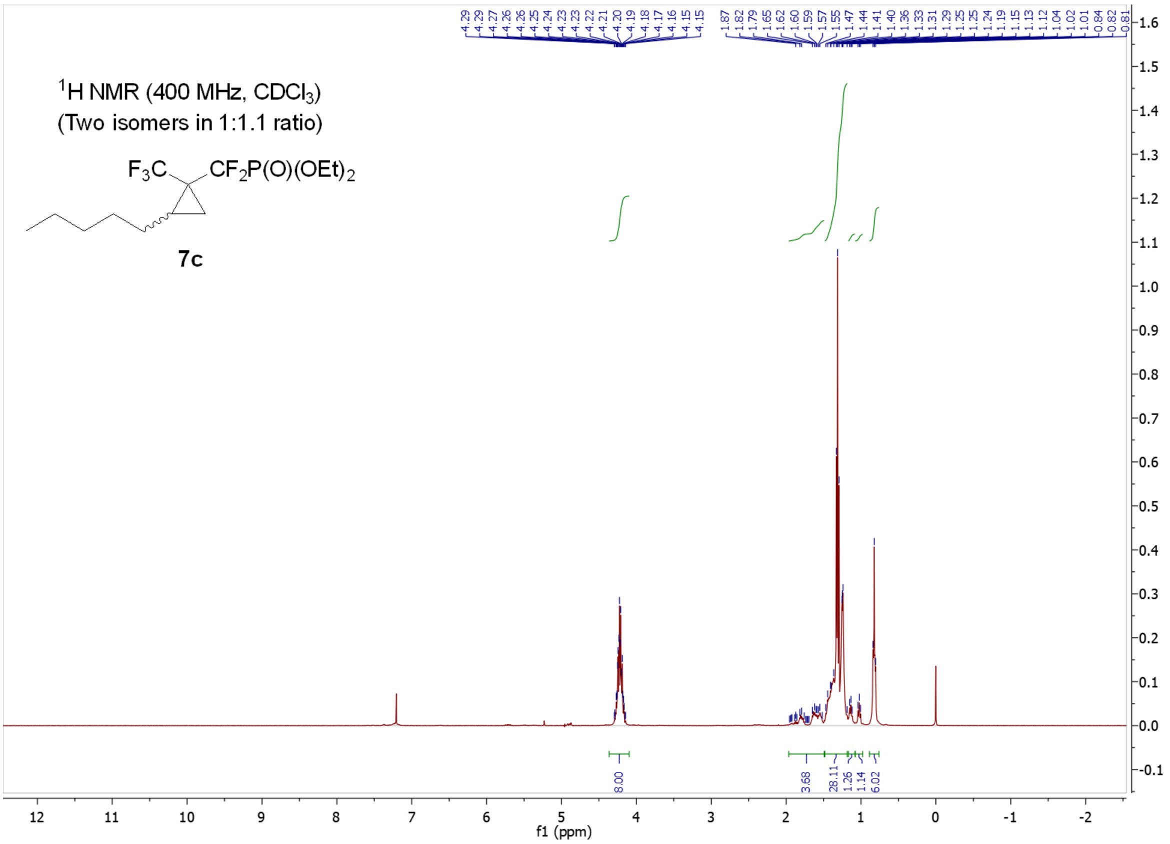
$^{1}$H NMR (400 MHz, CDCl$_3$)
(Two isomers in 1:1.1 ratio)
F$_3$C
CF$_2$P(O)(OEt)$_2$
7c
f1 (ppm)

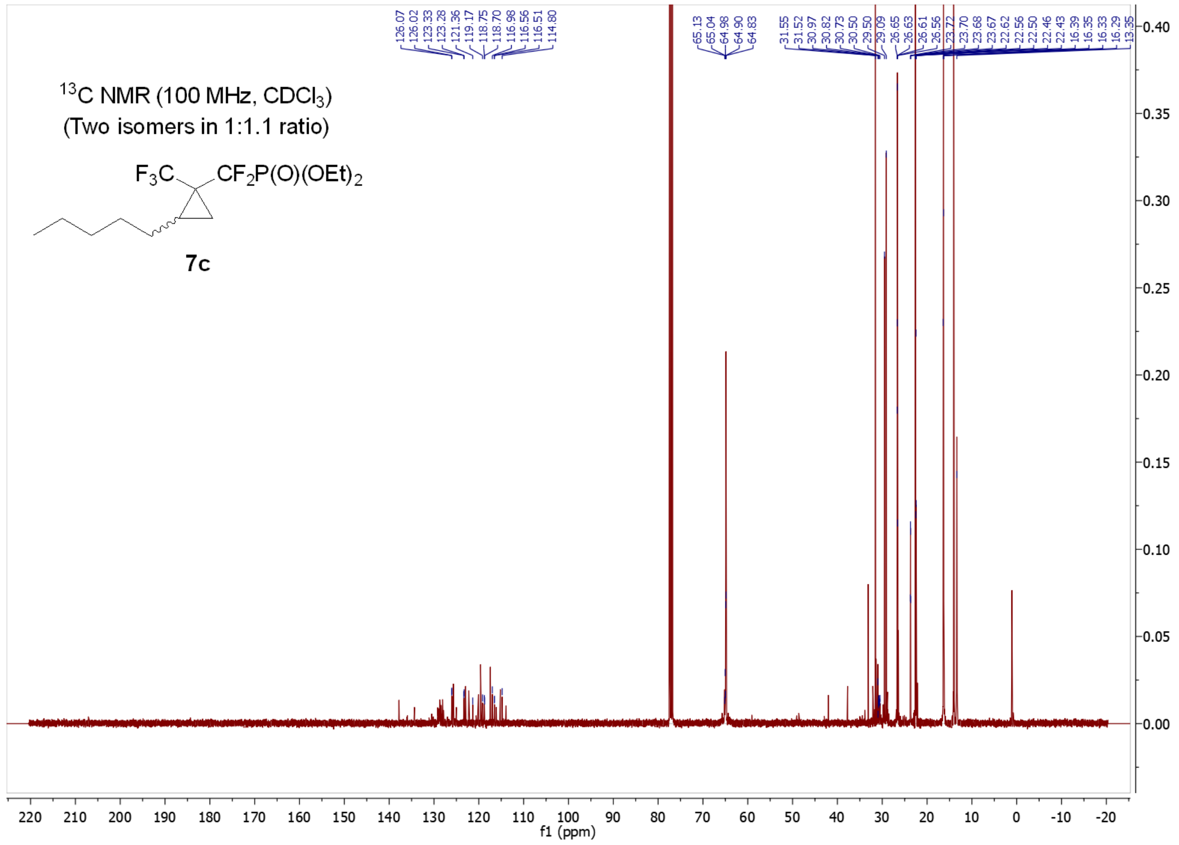

$^{13}$C NMR (100 MHz, CDCl$_3$)
(Two isomers in 1:1.1 ratio)
F$_3$C
CF$_2$P(O)(OEt)$_2$
7c
f1 (ppm)


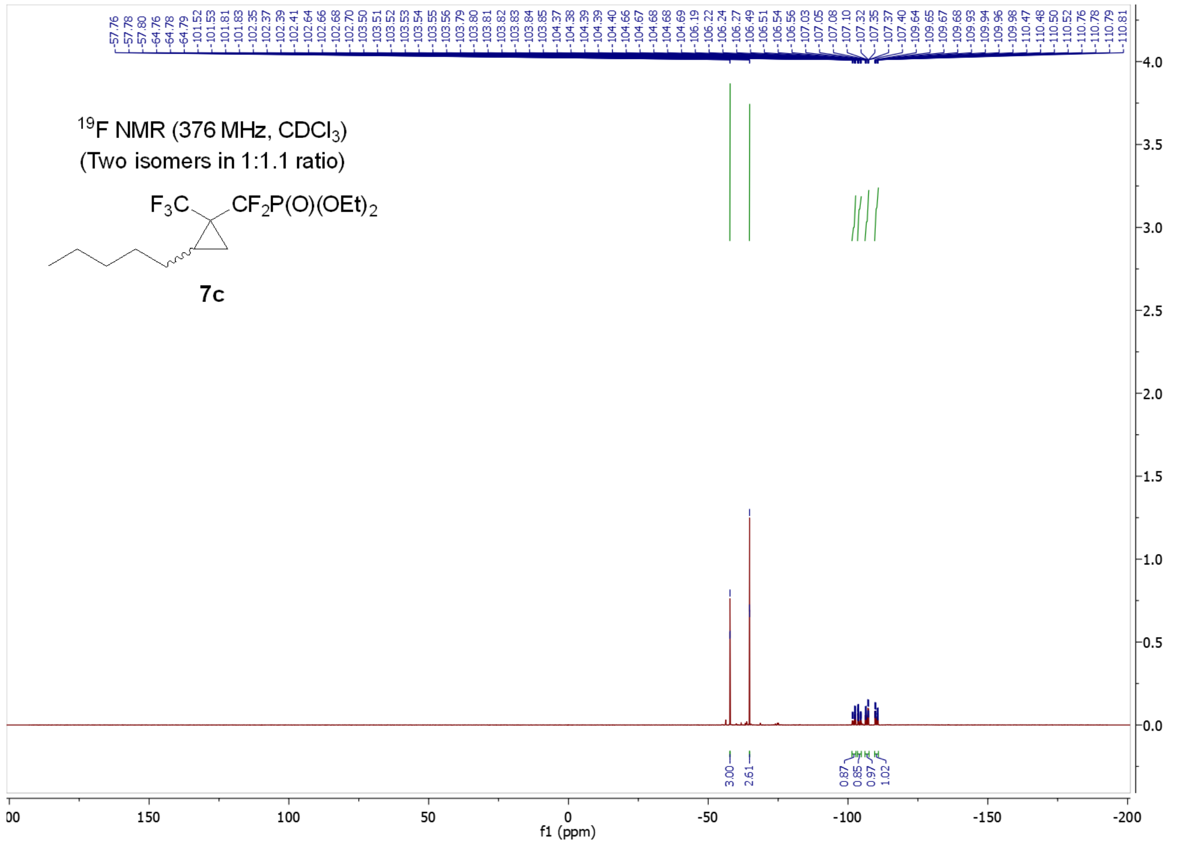

$^{19}$F NMR (376 MHz, CDCl$_3$)
(Two isomers in 1:1.1 ratio)
F$_3$C
CF$_2$P(O)(OEt)$_2$
7c
f1 (ppm)

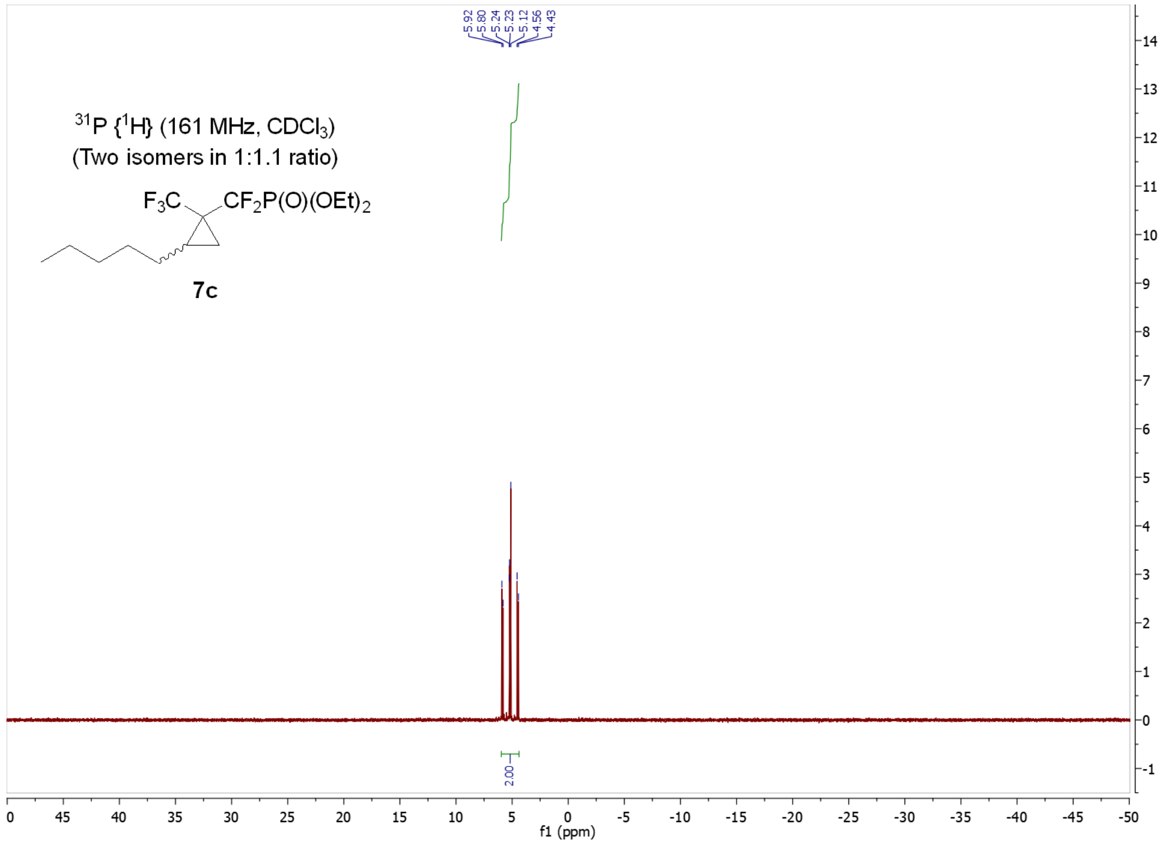

31P {1H} (161 MHz, CDCl3)
(Two isomers in 1:1.1 ratio)
F3C
CF2P(O)(OEt)2
7c
f1 (ppm)


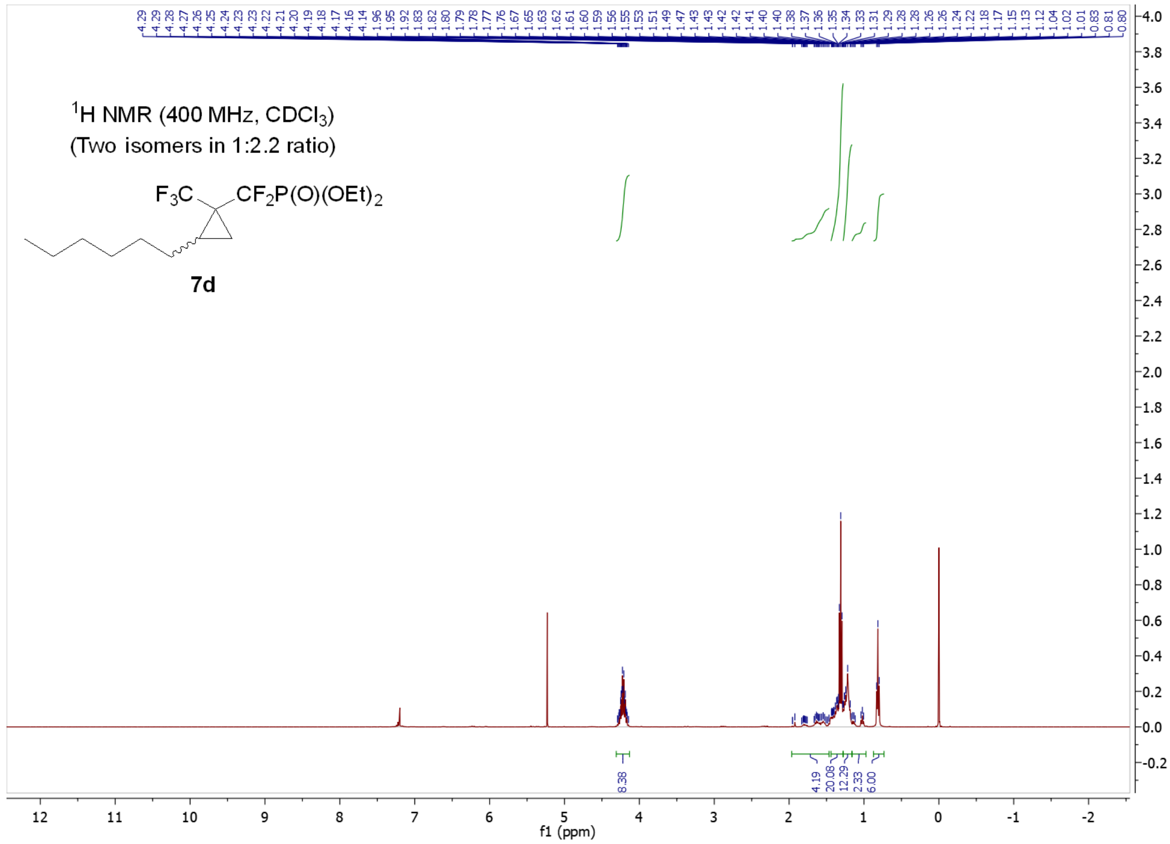

1H NMR (400 MHz, CDCl3)
(Two isomers in 1:2.2 ratio)
F3C
CF2P(O)(OEt)2
7d
f1 (ppm)

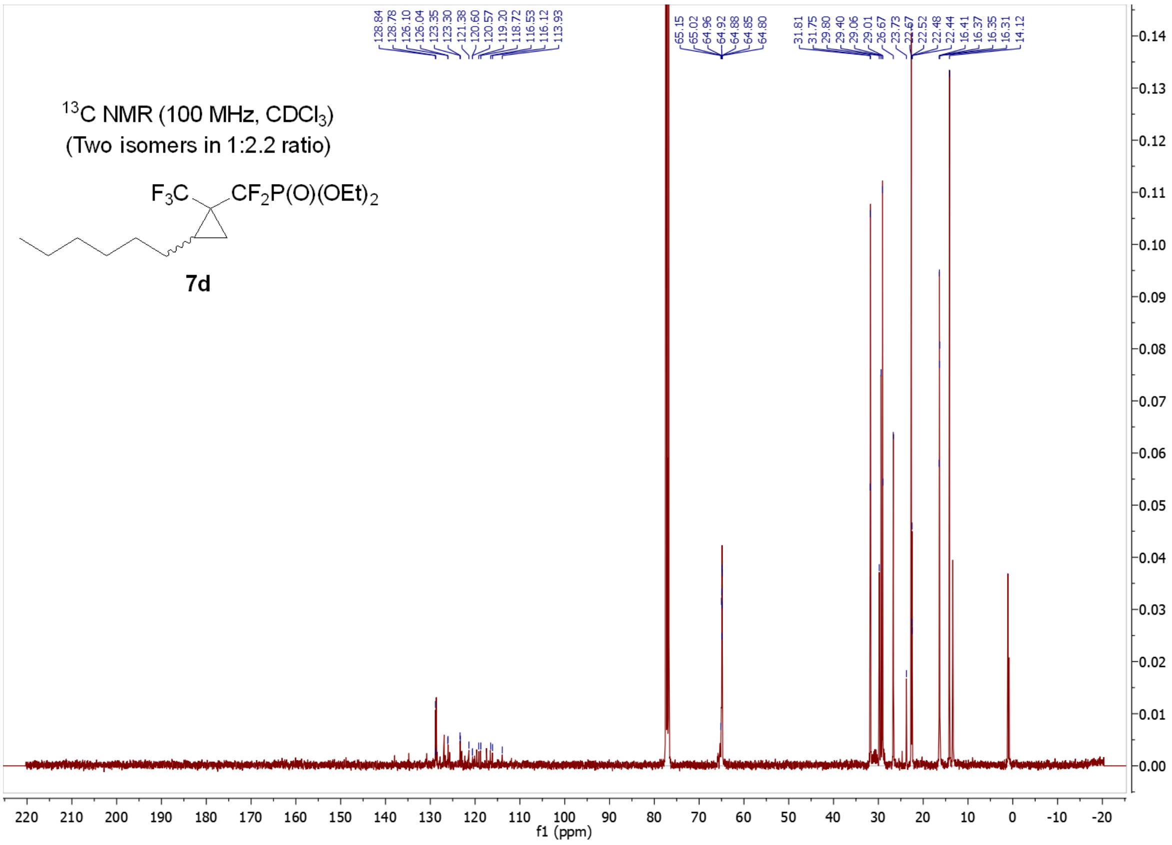
13C NMR (100 MHz, CDCl3)
(Two isomers in 1:2.2 ratio)
F3C
CF2P(O)(OEt)2
7d
f1 (ppm)

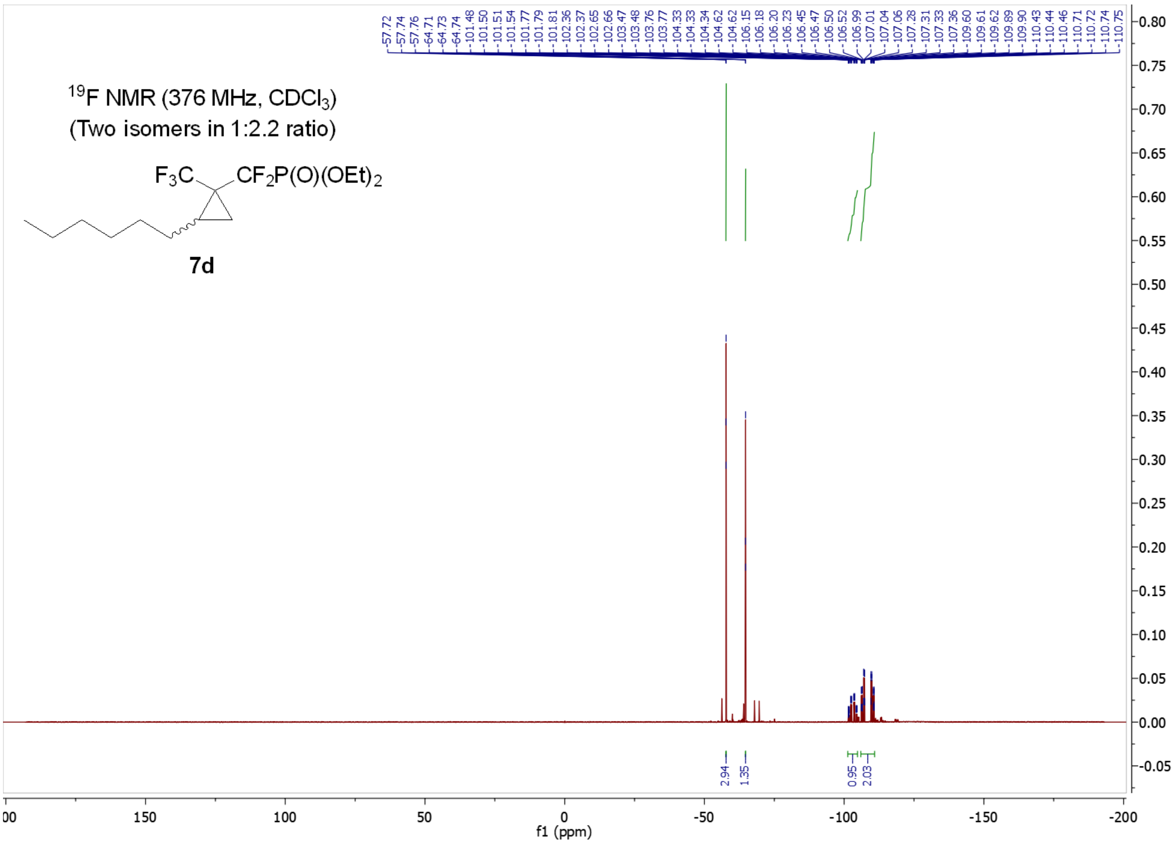
19F NMR (376 MHz, CDCl3)
(Two isomers in 1:2.2 ratio)
F3C
CF2P(O)(OEt)2
7d
f1 (ppm)

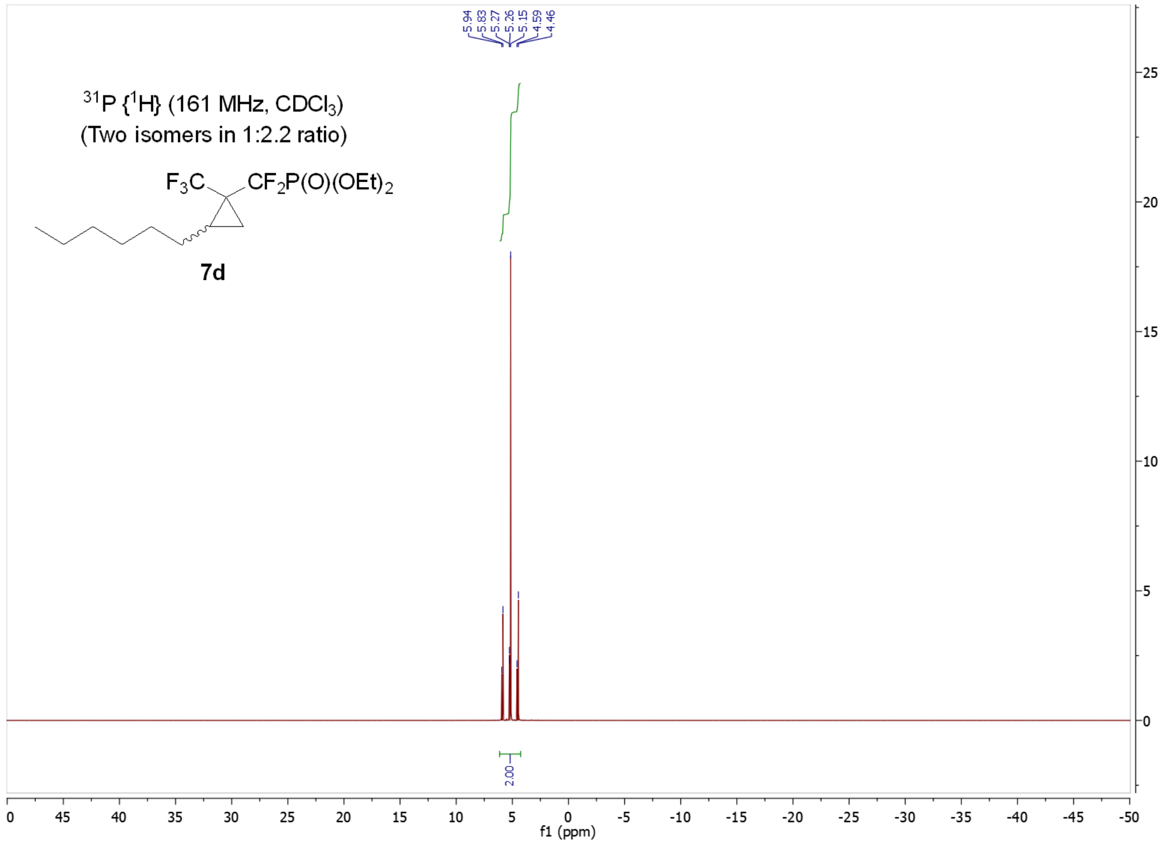
$^{31}P\{^1H\}$ (161 MHz, $CDCl_3$)
(Two isomers in 1:2.2 ratio)
$F_3C$
$CF_2P(O)(OEt)_2$
7d
f1 (ppm)

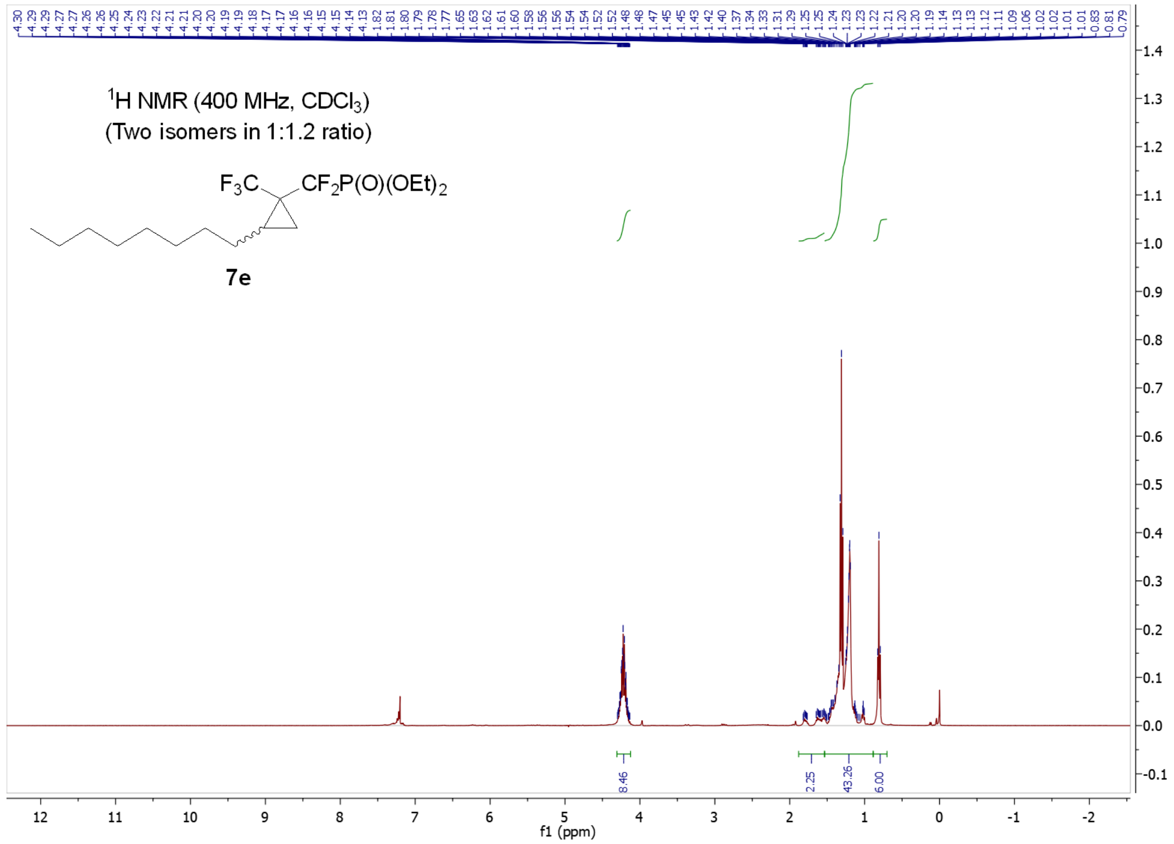
$^1H$ NMR (400 MHz, $CDCl_3$)
(Two isomers in 1:1.2 ratio)
$F_3C$
$CF_2P(O)(OEt)_2$
7e
f1 (ppm)

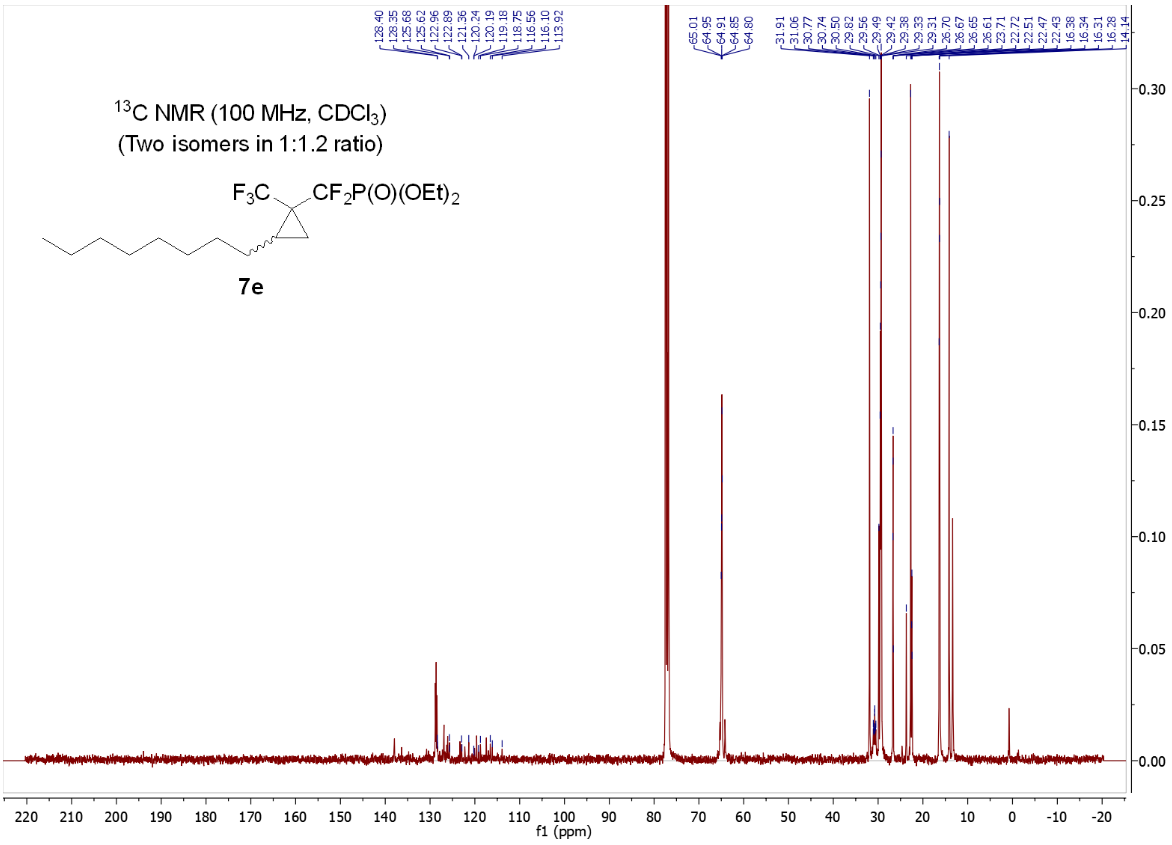
13C NMR (100 MHz, CDCl3)
(Two isomers in 1:1.2 ratio)
F3C
CF2P(O)(OEt)2
7e
128.40 128.35 125.68 125.62 122.96 122.89 121.36 120.24 120.19 119.18 118.75 116.56 116.10 113.92
65.01 64.95 64.91 64.85 64.80
31.91 31.06 30.77 30.74 30.50 29.82 29.56 29.49 29.42 29.38 29.33 29.31 26.70 26.67 26.65 26.61 23.71 22.72 22.51 22.47 22.43 16.38 16.34 16.31 16.28 14.14
f1 (ppm)

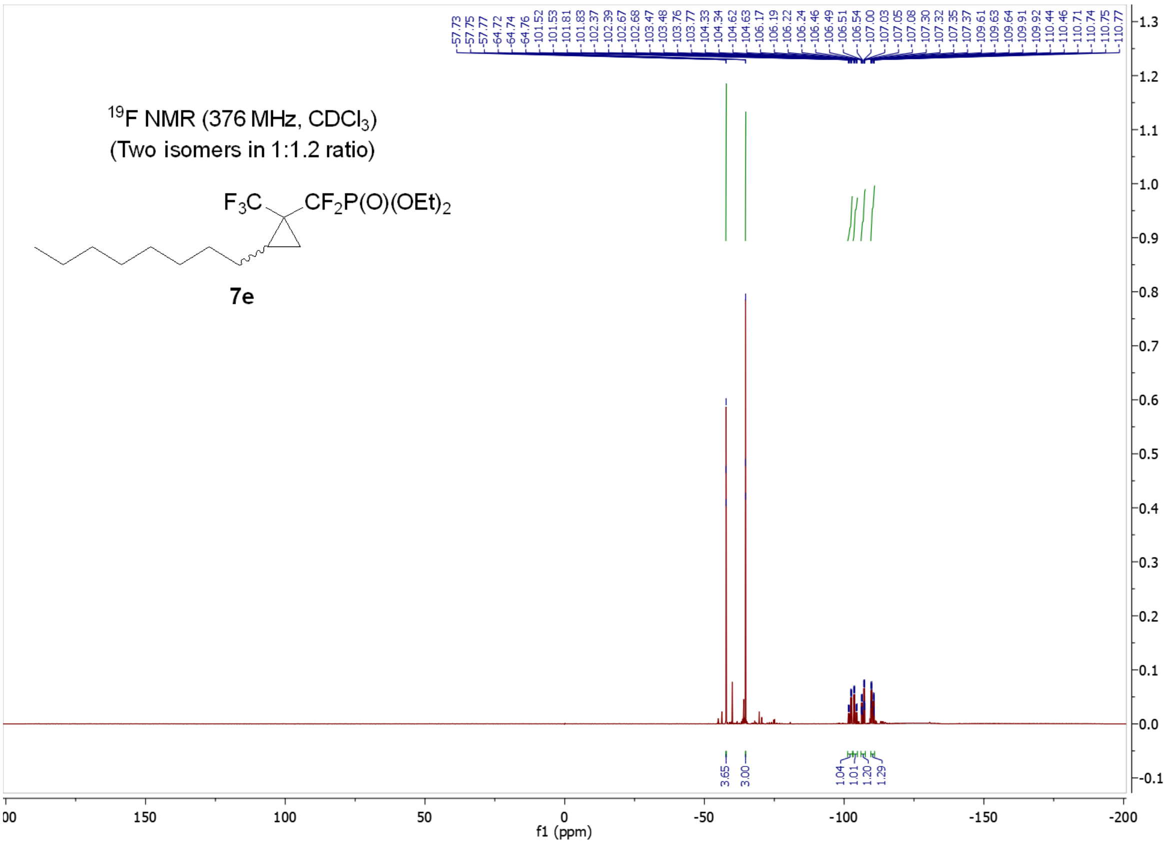
19F NMR (376 MHz, CDCl3)
(Two isomers in 1:1.2 ratio)
F3C
CF2P(O)(OEt)2
7e
-57.73 -57.75 -57.77 -64.72 -64.74 -64.76 -101.52 -101.53 -101.81 -101.83 -102.37 -102.39 -102.67 -102.68 -103.47 -103.48 -103.76 -103.77 -104.33 -104.34 -104.62 -104.63 -106.17 -106.19 -106.22 -106.24 -106.46 -106.49 -106.51 -106.54 -107.00 -107.03 -107.05 -107.08 -107.30 -107.32 -107.35 -107.37 -109.61 -109.63 -109.64 -109.91 -109.92 -110.44 -110.46 -110.71 -110.74 -110.75 -110.77
3.65 3.00 1.04 1.01 1.20 1.29
f1 (ppm)

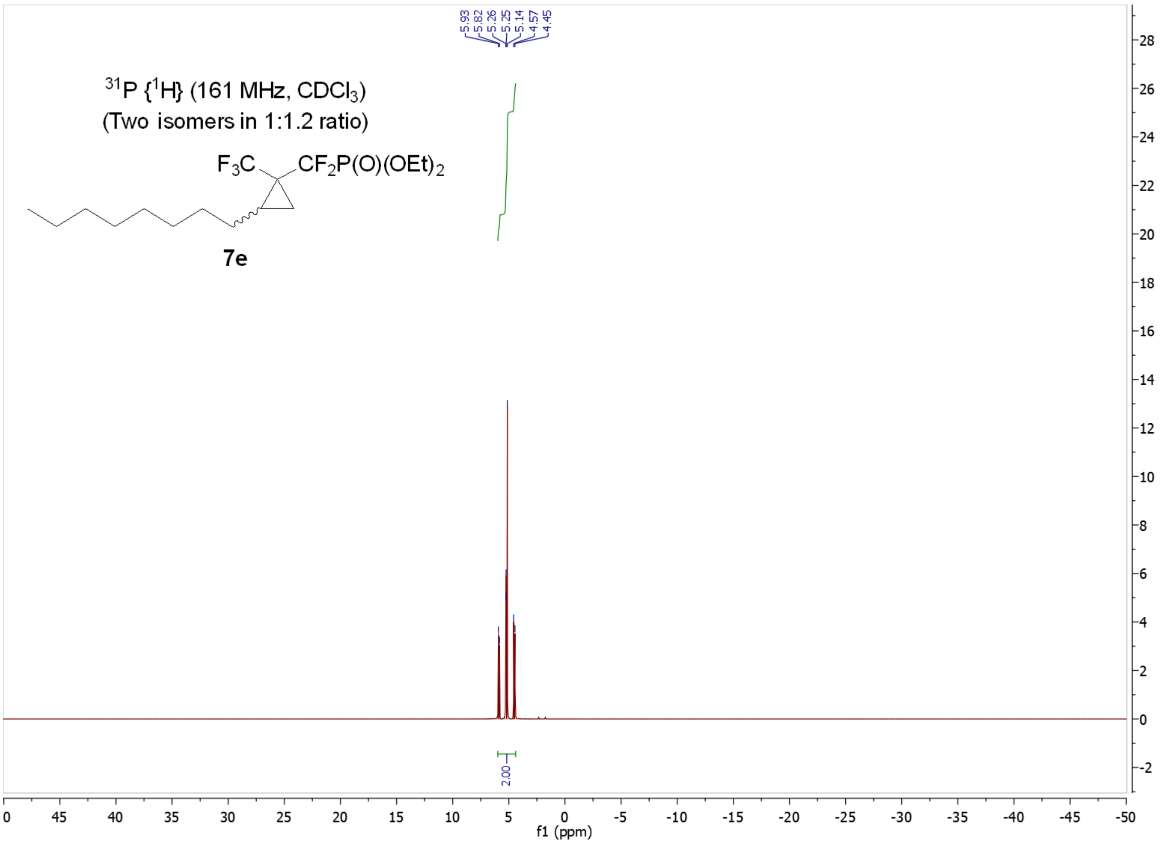
31P {1H} (161 MHz, CDCl3)
(Two isomers in 1:1.2 ratio)
F3C
CF2P(O)(OEt)2
7e
f1 (ppm)

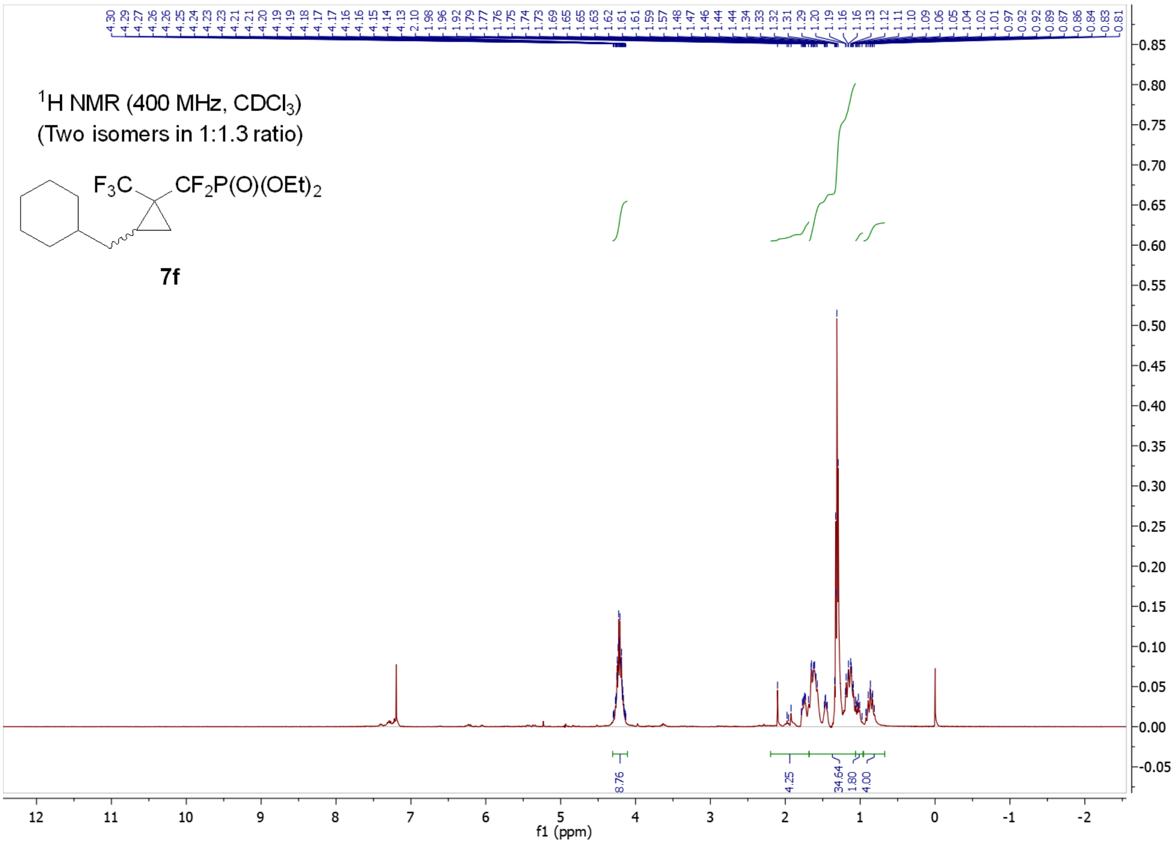
1H NMR (400 MHz, CDCl3)
(Two isomers in 1:1.3 ratio)
F3C
CF2P(O)(OEt)2
7f
f1 (ppm)

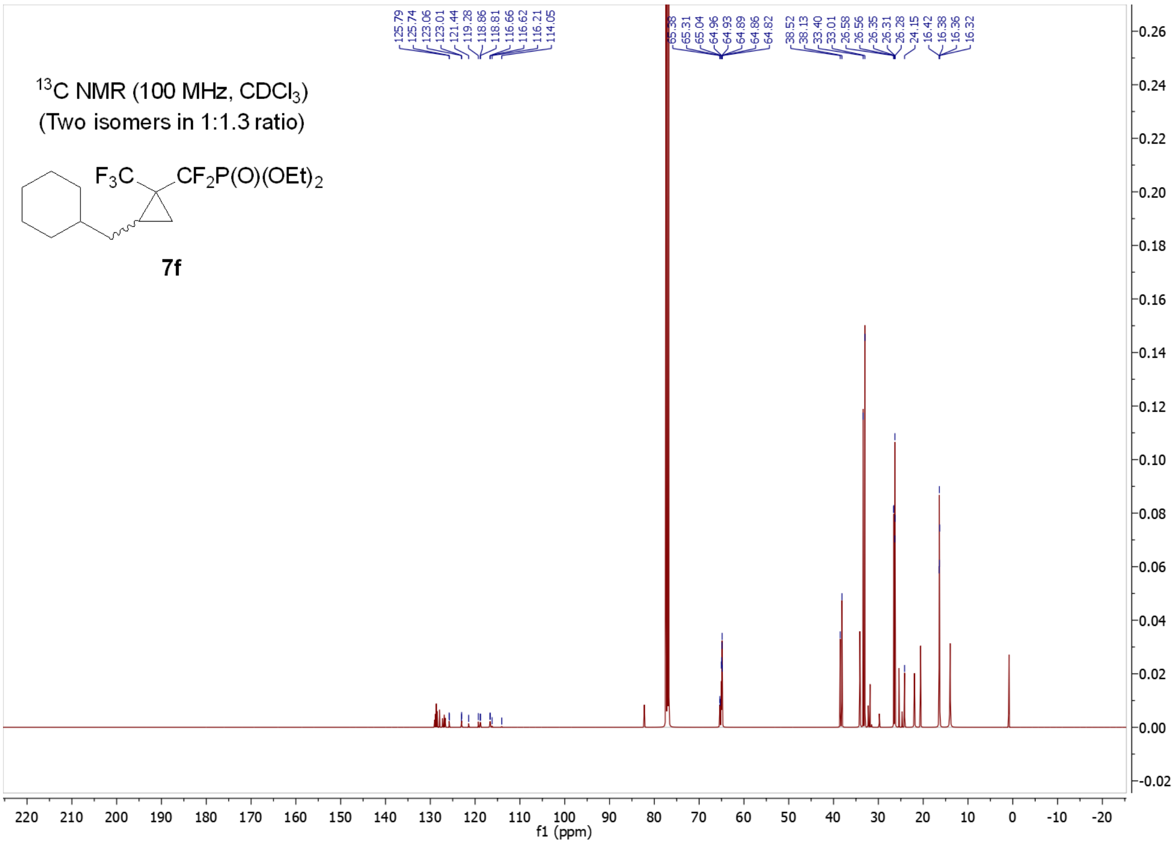
13C NMR (100 MHz, CDCl3)
(Two isomers in 1:1.3 ratio)
F3C
CF2P(O)(OEt)2
7f
f1 (ppm)

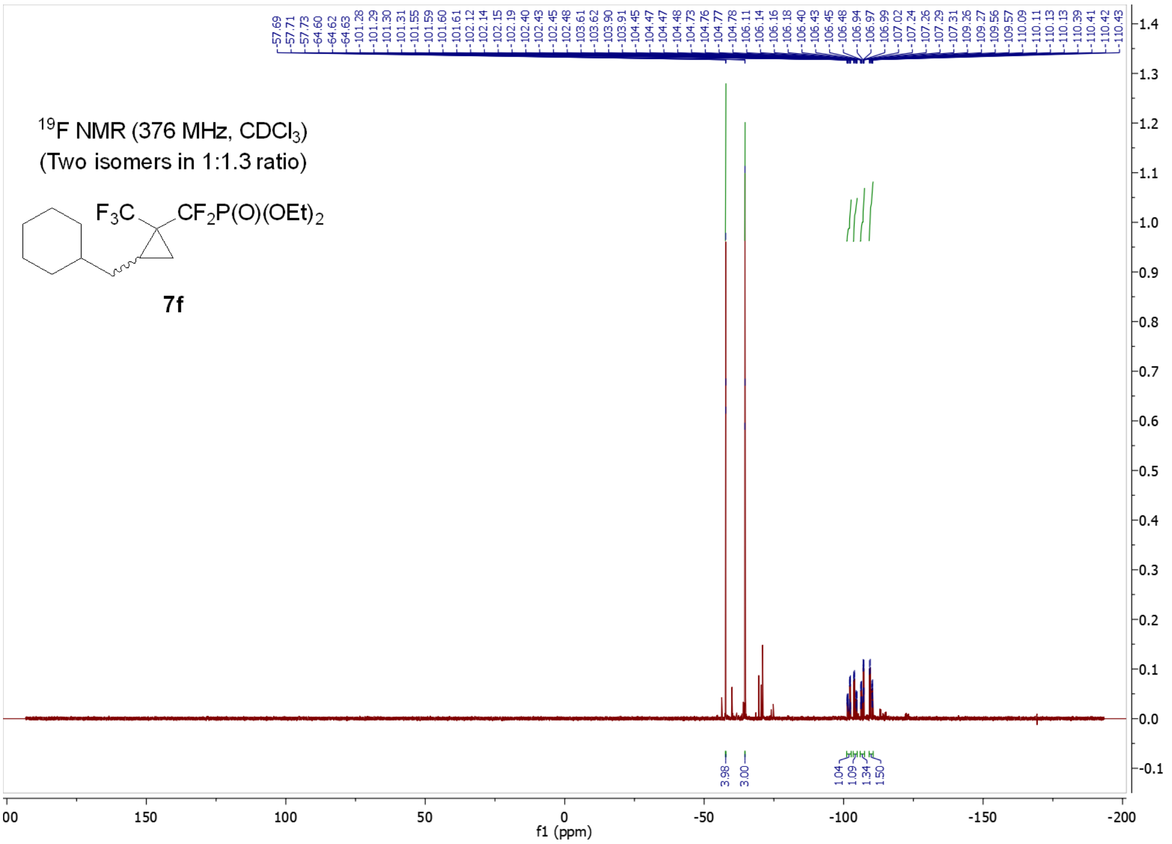
19F NMR (376 MHz, CDCl3)
(Two isomers in 1:1.3 ratio)
F3C
CF2P(O)(OEt)2
7f
f1 (ppm)

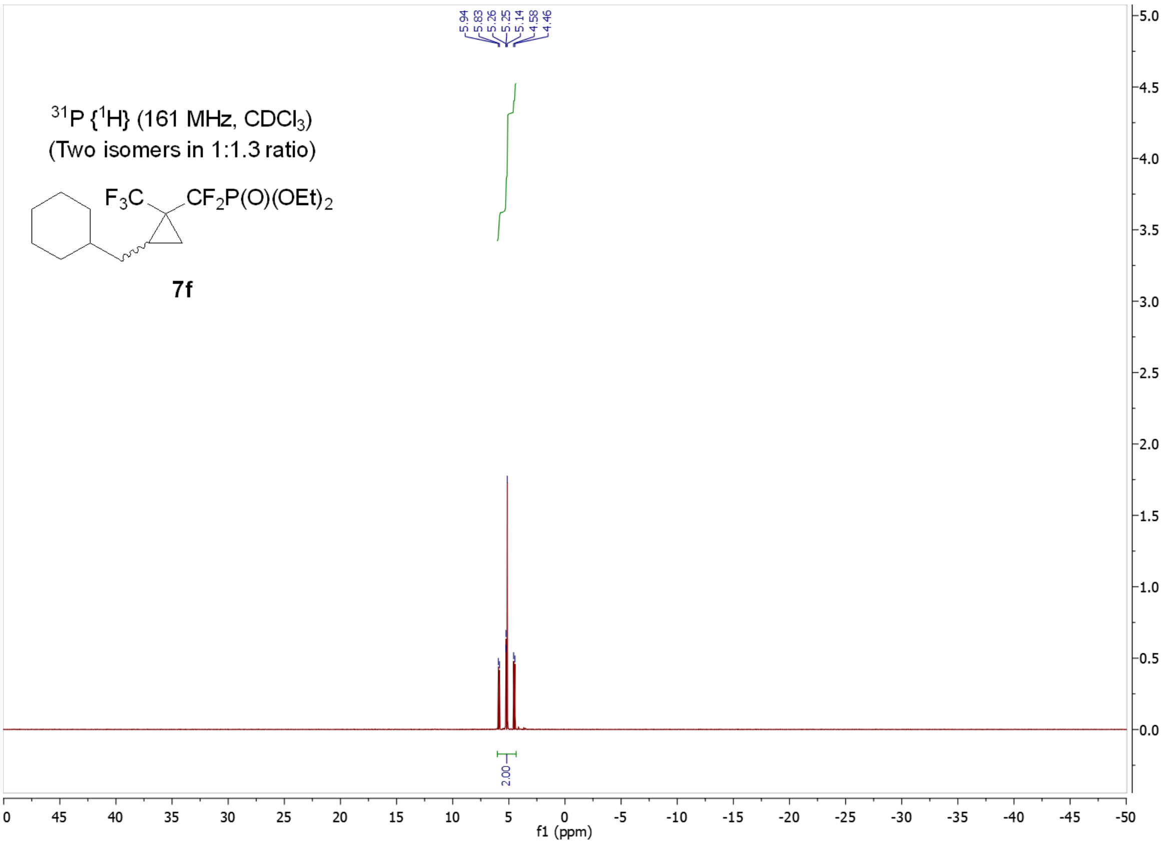

$^{31}P\{^1H\}$ (161 MHz, $CDCl_3$)
(Two isomers in 1:1.3 ratio)
$F_3C$
$CF_2P(O)(OEt)_2$
7f
f1 (ppm)


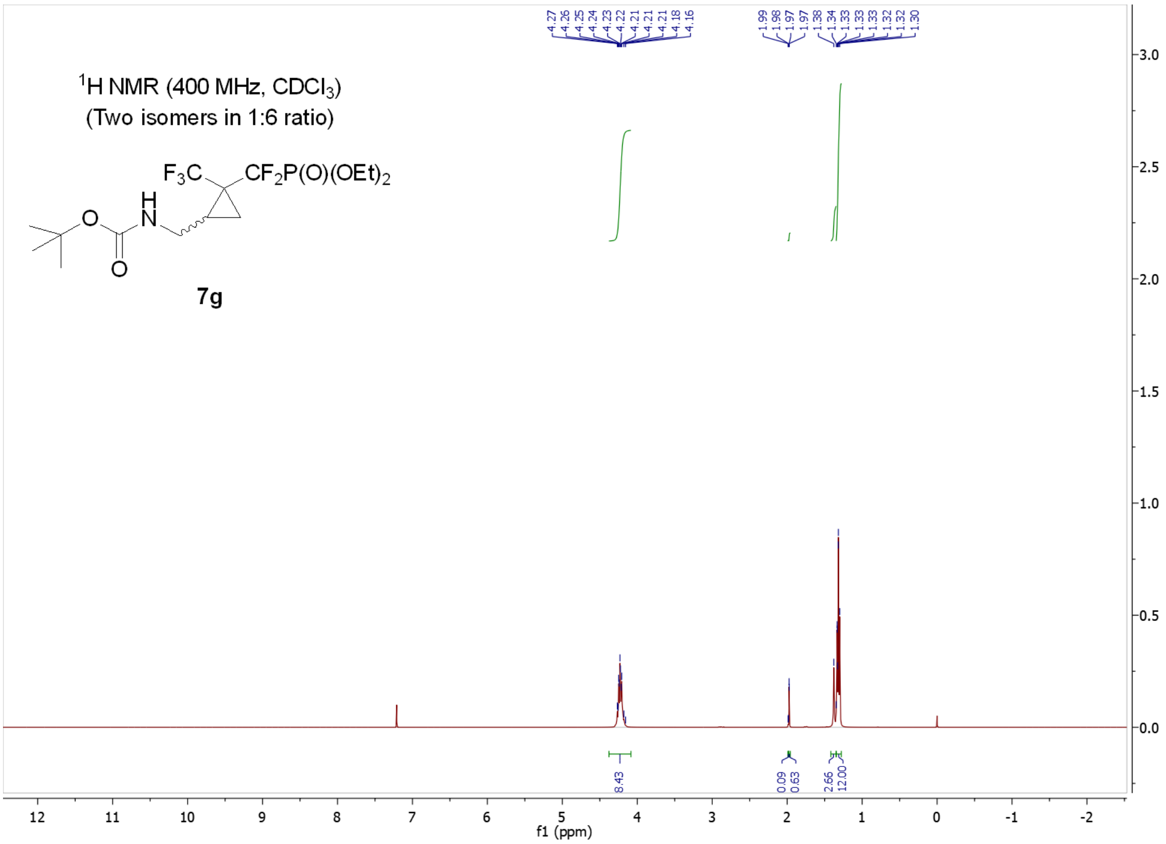

$^1H$ NMR (400 MHz, $CDCl_3$)
(Two isomers in 1:6 ratio)
$F_3C$
$CF_2P(O)(OEt)_2$
7g
f1 (ppm)

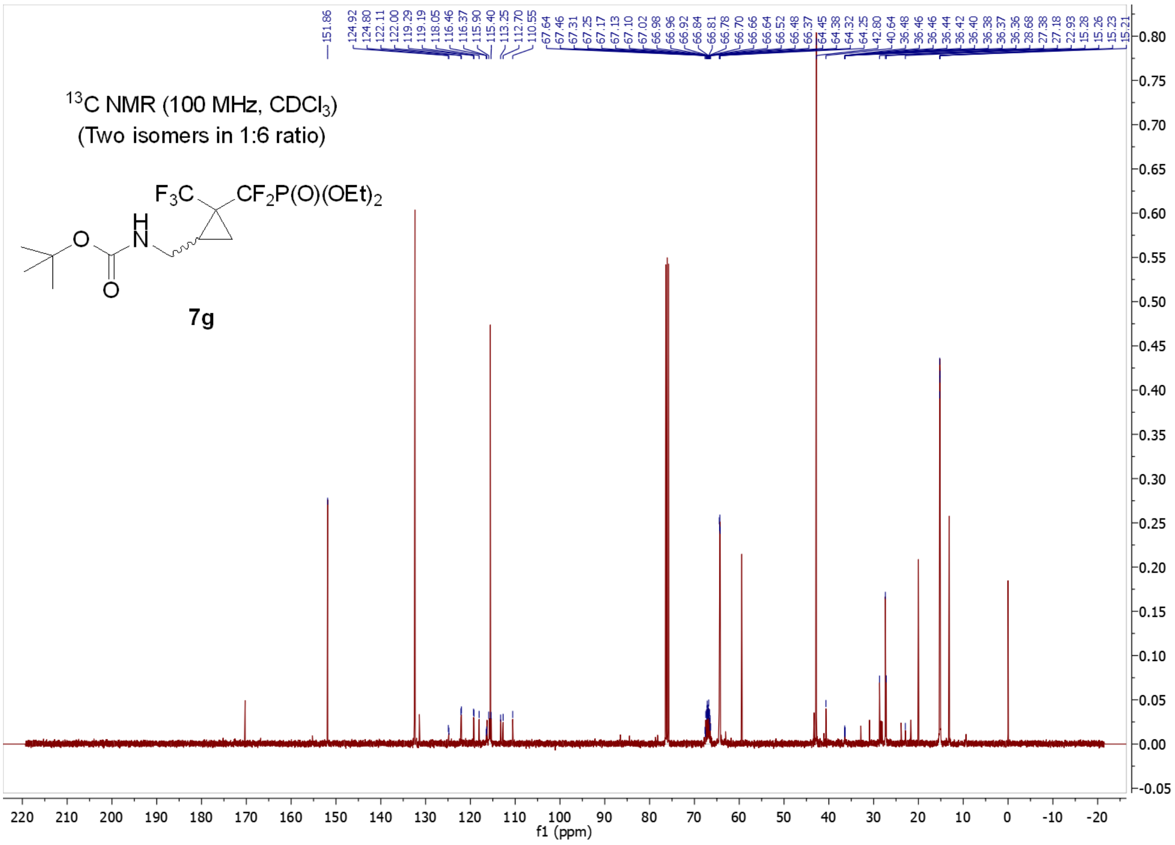

$^{13}$C NMR (100 MHz, $CDCl_3$)
(Two isomers in 1:6 ratio)
$F_3C$
$CF_2P(O)(OEt)_2$
7g
f1 (ppm)


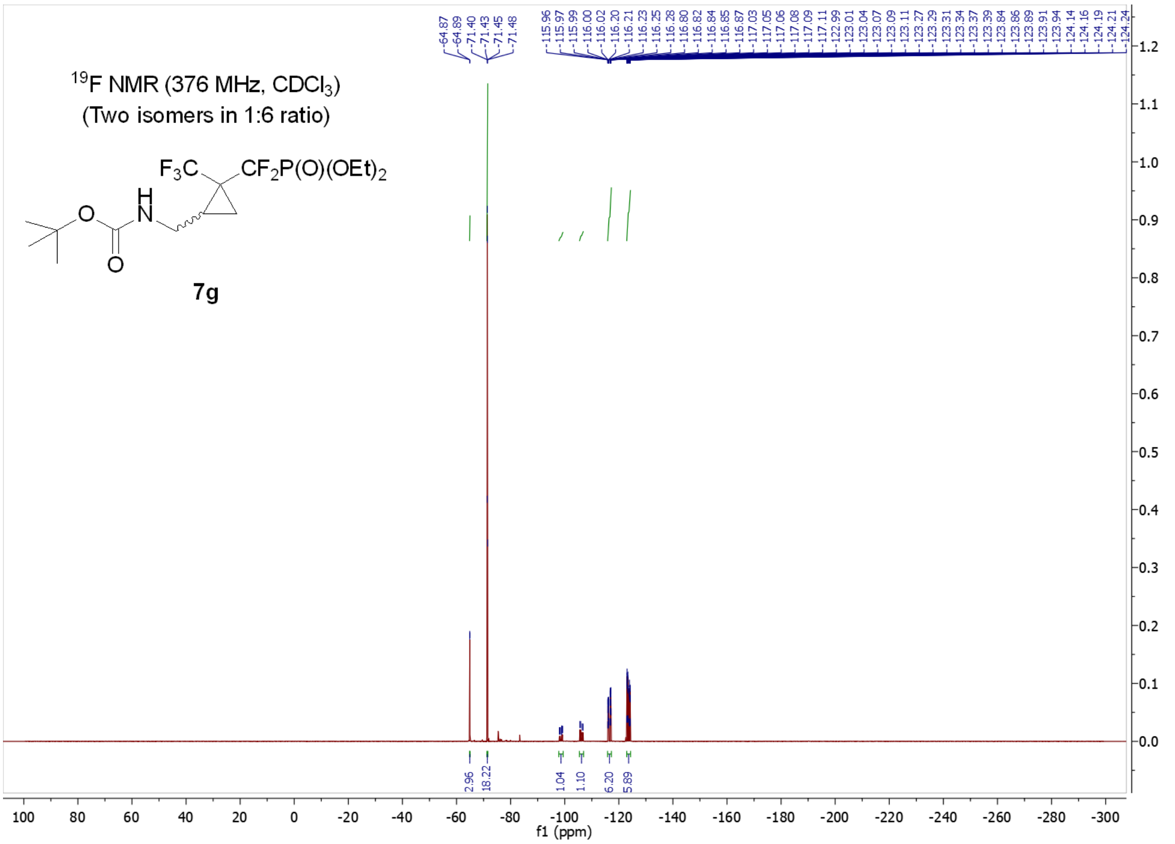

$^{19}$F NMR (376 MHz, $CDCl_3$)
(Two isomers in 1:6 ratio)
$F_3C$
$CF_2P(O)(OEt)_2$
7g
2.96
18.22
1.04
1.10
6.20
5.89
f1 (ppm)

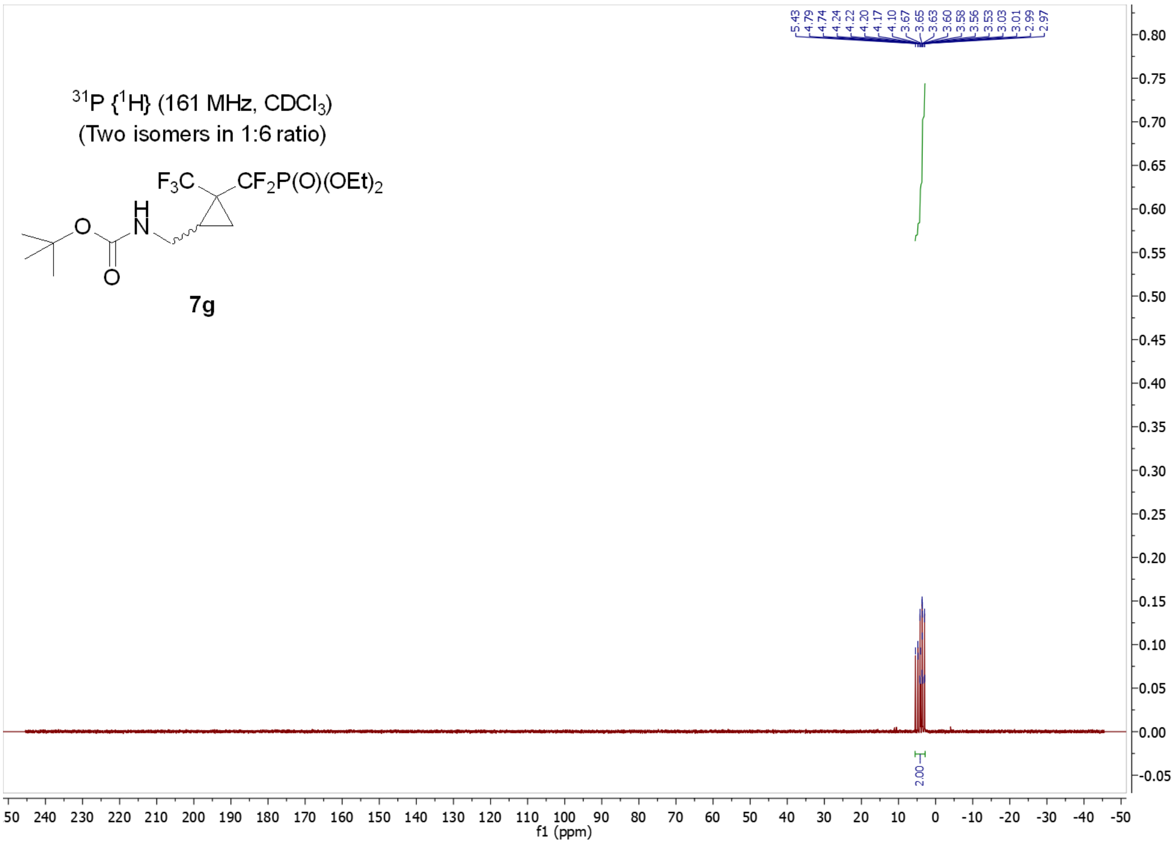

$^{31}P\{^1H\}$ (161 MHz, $CDCl_3$)
(Two isomers in 1:6 ratio)
$F_3C$
$CF_2P(O)(OEt)_2$
7g
5.43 4.79 4.74 4.24 4.22 4.20 4.17 4.10 3.67 3.65 3.63 3.60 3.58 3.56 3.53 3.03 3.01 2.99 2.97
2.00
f1 (ppm)

## 8. Copies of HOESY $^{19}F$, $^{1}H$ 2D NMR spectra

HOESY $^{19}F$-$^{1}H$ 2D NMR spectrum of **6c** (major isomer):

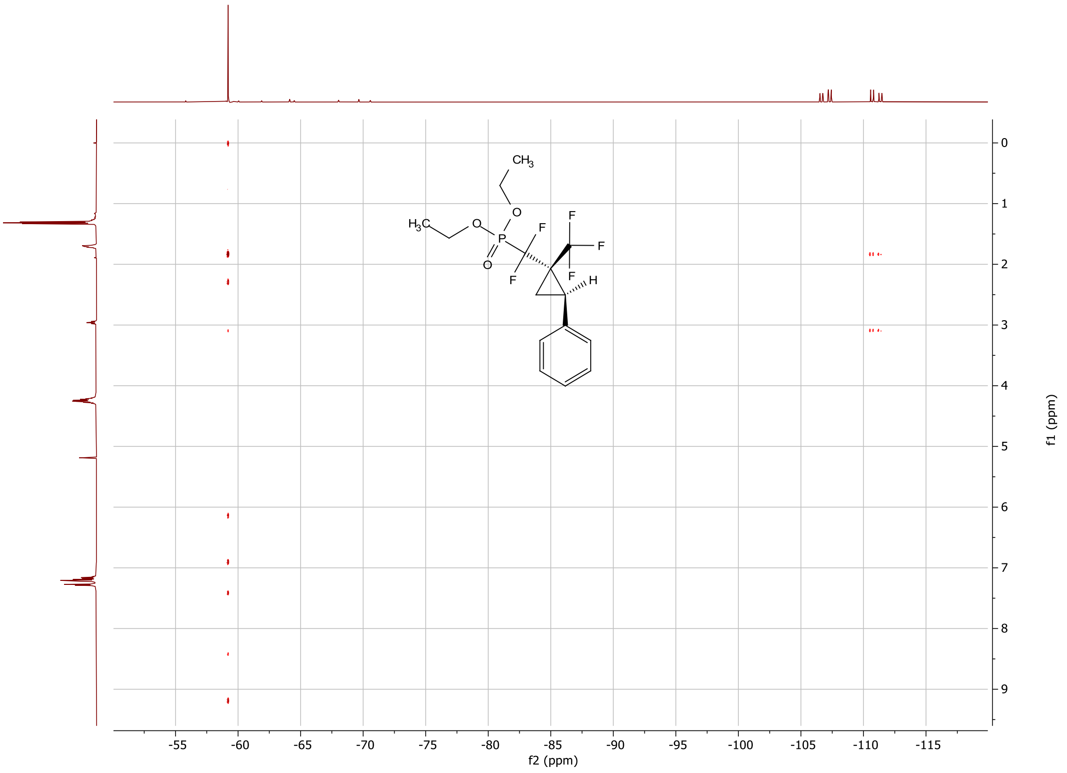


HOESY $^{19}F$, $^{1}H$ 2D NMR spectrum of **6d** (major isomer):

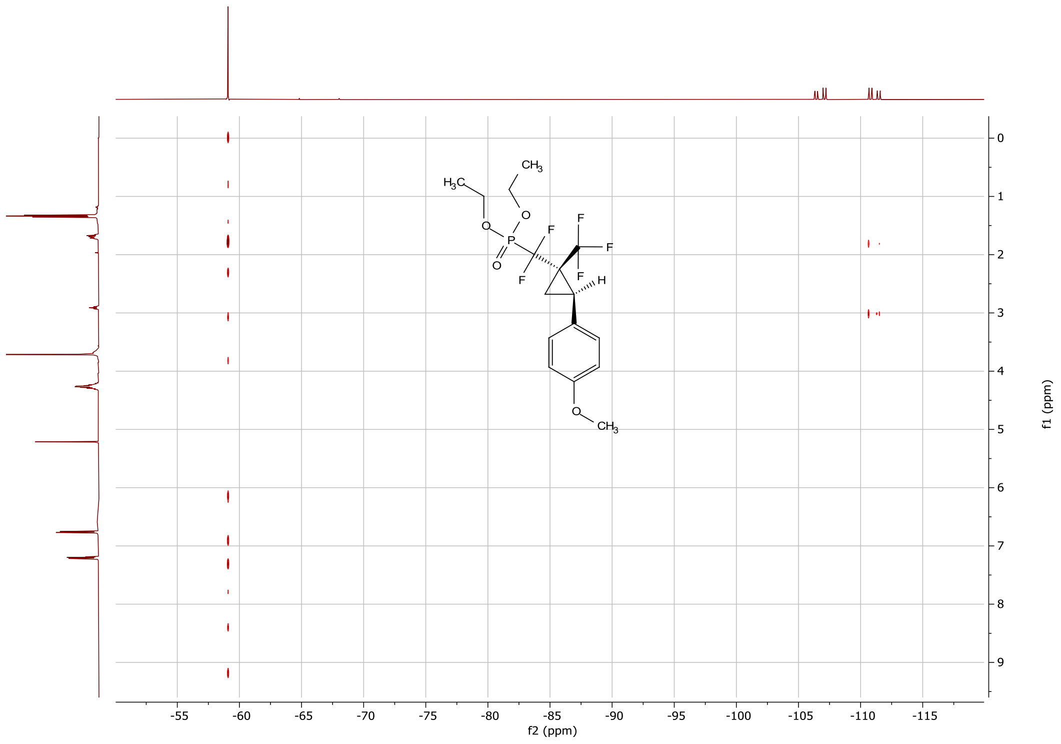


HOESY $^{19}$F, $^{1}$H 2D NMR spectrum of **6g** (major isomer):

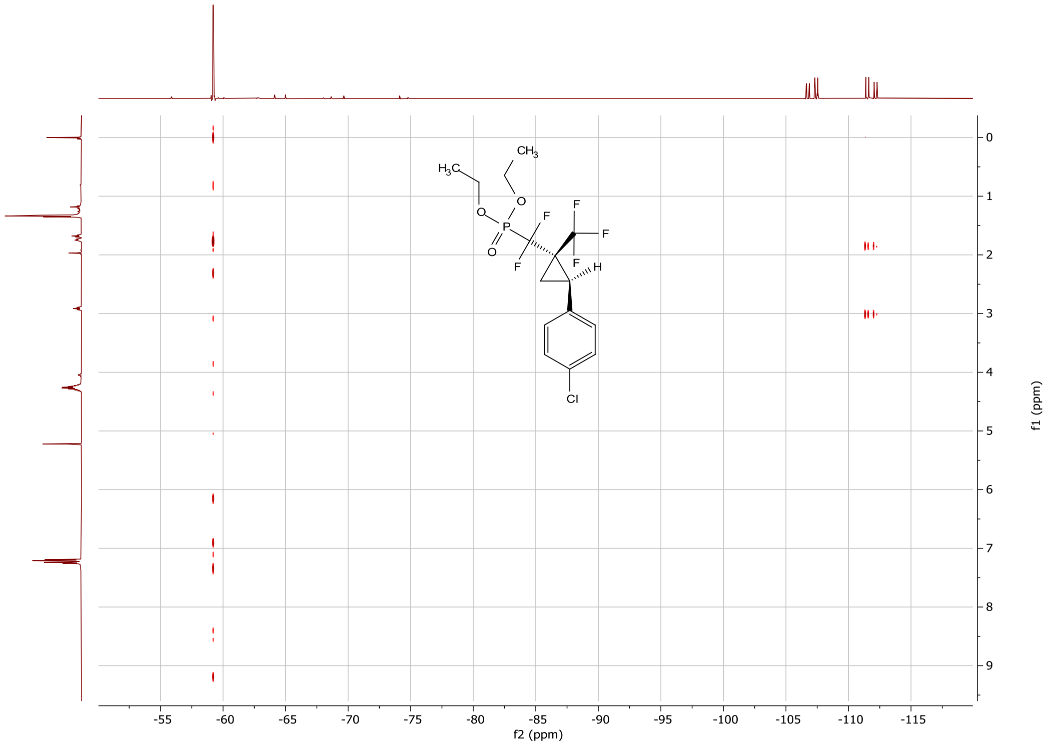

## 9. DFT calculations

Geometry optimizations were performed on the B3LYP/6-31G(d) (for H, C, F, N, O, P) and B3LYP/def2tzvp (for Cu and I) level of theory. More accurate energies were computed using 6-311+g(3d,p) instead of 6-31G(d). Solvation effects were considered by means of the default (SCRD) model of solvation with the solvent toluene. Thermochemical values refer to a temperature of 384 K. All calculations were carried out with an ultrafine integration grid (99,590). Transition structures were located either by a QST2 optimization or by directing the Berny geometry optimization algorithm towards a first order saddle point. Frequency calculations were conducted to characterize all stationary points.

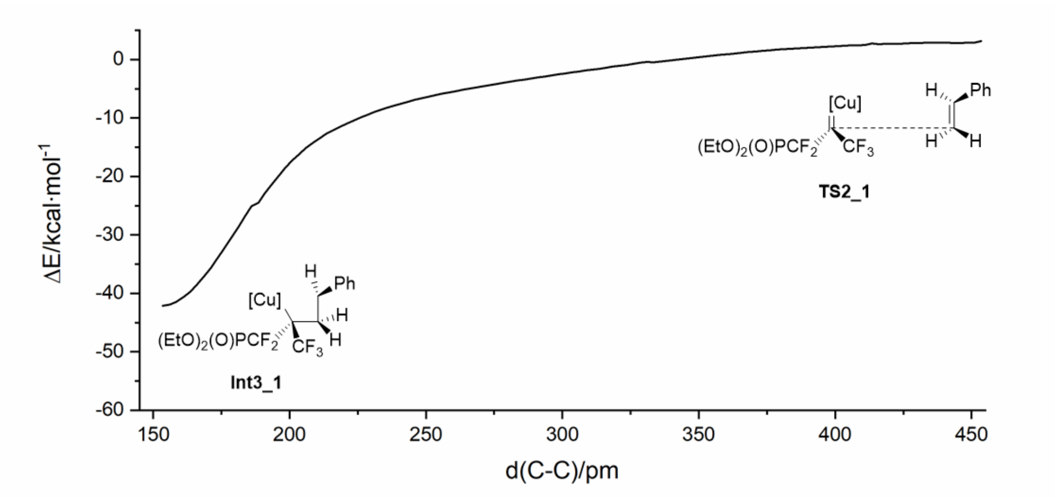


Figure 1. Change in electronic energy relative to **Int2** and styrene in dependence of the respective C-C bond that is formed along the reaction pathway leading to **Int3_1**.

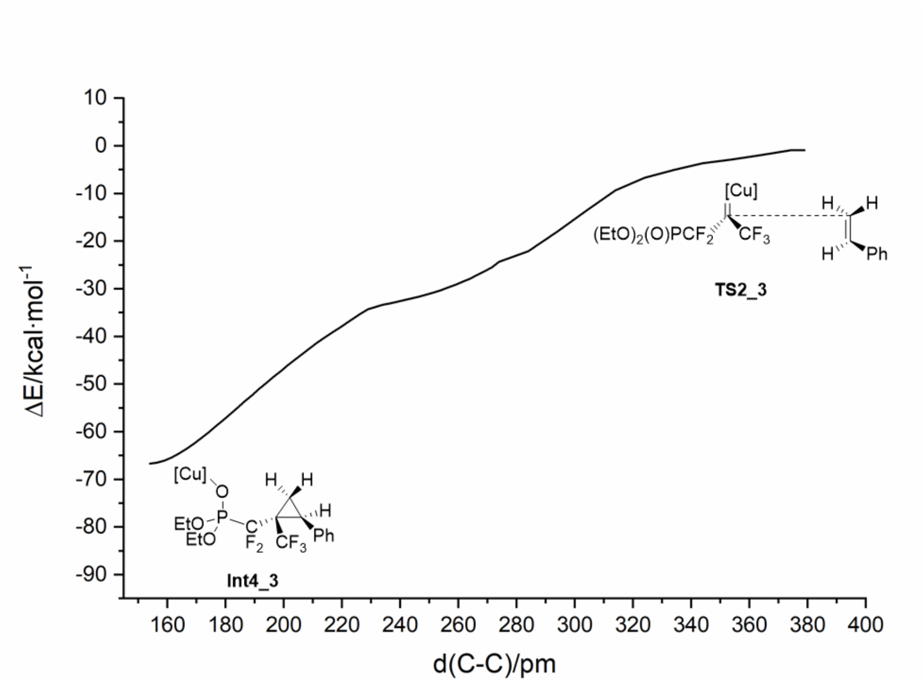


Figure 2. Change in electronic energy relative to **Int2** and styrene in dependence of the respective C-C bond that is formed along the reaction pathway leading directly to **Int4_4**.

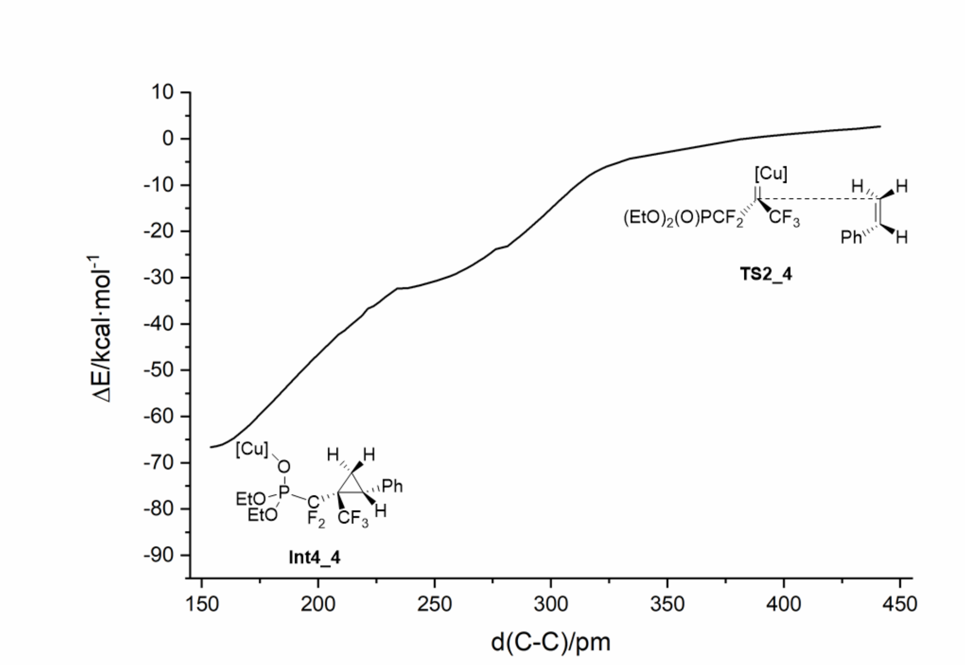


Figure 3. Change in electronic energy relative to Int2 and styrene in dependence of the respective C-C bond that is formed along the reaction pathway leading directly to Int4_4.

**Table S3.** Cartesian coordinates and calculated thermochemical values of substrates, catalyst, products, intermediates and transition states.

| Molecule | Coordinates | | | | Thermochemical values / Hartree |
|---|---|---|---|---|---|
| **Substrates and Catalyst** | | | | | |
| 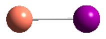 | Cu | 0.00000000 | 0.00000000 | -1.56964500 | Sum of electronic and zero-point Energies= |
| CuI | I | 0.00000000 | 0.00000000 | 0.85886200 | -1938.441800 |
| | | | | | Sum of electronic and thermal Energies= |
| | | | | | -1938.437992 |
| | | | | | Sum of electronic and thermal Enthalpies= |
| | | | | | -1938.436775 |
| | | | | | Sum of electronic and thermal Free Energies= |
| | | | | | -1938.475739 |
| 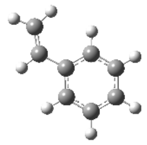 | C | 0.00000000 | 0.56045000 | 0.00000000 | Sum of electronic and zero-point Energies= |
| | C | 1.33856100 | 0.13047200 | 0.00000000 | -309.615611 |
| | C | 1.66266100 | -1.22604900 | 0.00000000 | Sum of electronic and thermal Energies= |
| | C | 0.65031400 | -2.18606800 | 0.00000000 | -309.604921 |
| | C | -0.68690200 | -1.77578000 | 0.00000000 | Sum of electronic and thermal Enthalpies= |
| Styrene | C | -1.00866300 | -0.42165000 | 0.00000000 | -309.603705 |
| | H | 2.13267900 | 0.87393700 | 0.00000000 | Sum of electronic and thermal Free Energies= |
| | H | 2.70575900 | -1.53168600 | 0.00000000 | -309.657859 |
| | H | 0.89764100 | -3.24429600 | 0.00000000 | |
| | H | -1.48235700 | -2.51673000 | 0.00000000 | |
| | H | -2.05330000 | -0.12369100 | 0.00000000 | |
| | C | -0.28181400 | 2.00610500 | 0.00000000 | |
| | H | 0.60685100 | 2.63777400 | 0.00000000 | |
| | C | -1.47930100 | 2.60565200 | 0.00000000 | |
| | H | -1.55872500 | 3.68913900 | 0.00000000 | |
| | H | -2.41768400 | 2.05676000 | 0.00000000 | |
| 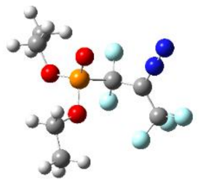 | P | 1.16959500 | 0.22765800 | 0.45381800 | Sum of electronic and zero-point Energies= |
| | O | 1.23405300 | -0.07076200 | 1.90832300 | -1448.747538 |
| | C | -0.11417800 | -0.84814800 | -0.38413700 | Sum of electronic and thermal Energies= |
| | C | -1.49104900 | -0.76415900 | 0.21056900 | -1448.717522 |
| | N | -1.69550200 | -1.27555800 | 1.39127700 | Sum of electronic and thermal Enthalpies= |
| | N | -1.86745400 | -1.73500300 | 2.41621600 | -1448.716306 |
| | C | -2.62837200 | -0.06396500 | -0.46231900 | Sum of electronic and thermal Free Energies= |
| Adduct 3 | F | -3.70645100 | -0.04732700 | 0.35039900 | -1448.818373 |
| | F | -2.32310600 | 1.21180600 | -0.77651600 | |
| | F | -2.99393400 | -0.66495400 | -1.61430200 | |
| | F | 0.35947500 | -2.14127400 | -0.26667200 | |
| | F | -0.20251500 | -0.55166300 | -1.71038800 | |
| | O | 0.64411500 | 1.65650700 | -0.02455300 | |
| | O | 2.52757800 | 0.04129700 | -0.38058400 | |
| | C | 3.57214400 | -0.90026500 | 0.02115300 | |
| | H | 4.49099800 | -0.30897600 | 0.06586900 | |
| | H | 3.35229700 | -1.27332500 | 1.02502300 | |
| | C | 1.28161300 | 2.88885300 | 0.43187100 | |
| | H | 2.30465000 | 2.90883700 | 0.04272100 | |
| | H | 1.31701600 | 2.87723900 | 1.52580100 | |
| | C | 3.67905300 | -2.01990100 | -0.99483400 | |
| | H | 3.88480600 | -1.62250400 | -1.99424400 | |
| | H | 4.50077300 | -2.68947200 | -0.71433900 | |
| | H | 2.75435600 | -2.60289100 | -1.03356200 | |
| | C | 0.46185000 | 4.04977600 | -0.08841100 | |
| | H | -0.56380900 | 4.00241300 | 0.29129800 | |
| | H | 0.91211100 | 4.99240600 | 0.24289800 | |

| | | | | | |
|---|---|---|---|---|---|
| | H | 0.43001700 | 4.04655900 | -1.18272300 | |
| Products | | | | | |
| 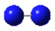 | N | 0.00000000 | 0.00000000 | 0.55259000 | Sum of electronic and zero-point Energies= |
| | N | 0.00000000 | 0.00000000 | -0.55259000 | -109.553048 |
| $N_2$ | | | | | Sum of electronic and thermal Energies= |
| | | | | | -109.550006 |
| | | | | | Sum of electronic and thermal Enthalpies= |
| | | | | | -109.548790 |
| | | | | | Sum of electronic and thermal Free Energies= |
| | | | | | -109.577887 |
| 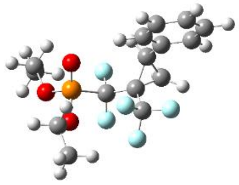 | P | -1.84936700 | 0.05289600 | -0.66611000 | Sum of electronic and zero-point Energies= |
| | O | -1.05378800 | -0.18913800 | -1.89845100 | -1648.891070 |
| | C | -1.03733100 | -0.67385300 | 0.86506100 | Sum of electronic and thermal Energies= |
| | C | 0.44905100 | -0.41984600 | 1.07773900 | -1648.853069 |
| | C | 0.83778500 | 1.03274100 | 1.24914900 | Sum of electronic and thermal Enthalpies= |
| | F | 2.09933600 | 1.17051000 | 1.70533100 | -1648.851853 |
| Pr1 | F | 0.77035400 | 1.71597400 | 0.07763300 | Sum of electronic and thermal Free Energies= |
| | F | 0.03048300 | 1.67554400 | 2.11968600 | -1648.969463 |
| | F | -1.22730000 | -2.04150700 | 0.73557200 | |
| | F | -1.74643900 | -0.29868500 | 1.97383300 | |
| | O | -2.11006500 | 1.55614200 | -0.20054700 | |
| | O | -3.32916800 | -0.56929400 | -0.63138800 | |
| | C | -3.65513400 | -1.83988500 | -1.27258200 | |
| | H | -4.30223500 | -1.59527200 | -2.12014400 | |
| | H | -2.74552200 | -2.30357000 | -1.66326800 | |
| | C | -2.01966500 | 2.67318700 | -1.13663500 | |
| | H | -2.92882700 | 2.67612900 | -1.74684500 | |
| | H | -1.15749000 | 2.51709000 | -1.78994400 | |
| | C | -4.36020300 | -2.73341700 | -0.27238400 | |
| | H | -5.26268500 | -2.24948400 | 0.11541800 | |
| | H | -4.65391200 | -3.67031100 | -0.76047500 | |
| | H | -3.70091600 | -2.96942900 | 0.56813600 | |
| | C | -1.88748000 | 3.94264100 | -0.32286400 | |
| | H | -0.98444200 | 3.91056800 | 0.29389400 | |
| | H | -1.82085200 | 4.80376200 | -0.99776900 | |
| | H | -2.75441300 | 4.08200700 | 0.33126100 | |
| | C | 1.22095900 | -1.45350000 | 1.87704300 | |
| | H | 1.99586300 | -1.06928300 | 2.53164300 | |
| | H | 0.65709200 | -2.28935800 | 2.27696900 | |
| | C | 1.42815500 | -1.37569900 | 0.39317500 | |
| | H | 0.90367300 | -2.14183400 | -0.17209500 | |
| | C | 2.70088600 | -0.92553800 | -0.26443900 | |
| | C | 2.64480300 | -0.34396000 | -1.54030200 | |
| | C | 3.94894700 | -1.12789100 | 0.33487900 | |
| | C | 3.81614600 | 0.03248900 | -2.19617900 | |
| | H | 1.67736000 | -0.18699800 | -2.01048600 | |
| | C | 5.12192300 | -0.75083300 | -0.32251600 | |
| | H | 4.00557700 | -1.58815300 | 1.31767700 | |
| | C | 5.05861500 | -0.16871200 | -1.58902500 | |
| | H | 3.75863100 | 0.48431900 | -3.18312700 | |
| | H | 6.08407100 | -0.91457500 | 0.15604400 | |
| | H | 5.97102500 | 0.12521100 | -2.10138200 | |
| 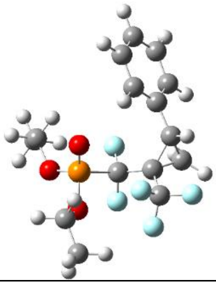 | P | -1.05149000 | 1.18418800 | 0.34887400 | Sum of electronic and zero-point Energies= |
| | O | -0.43842200 | 1.06146700 | 1.69611600 | -1648.888421 |
| | C | -0.29749800 | 0.04177500 | -0.94831600 | Sum of electronic and thermal Energies= |
| | C | -0.03881200 | -1.41928200 | -0.60675500 | -1648.850513 |
| | C | -1.25339100 | -2.18303700 | -0.12458400 | Sum of electronic and thermal Enthalpies= |
| | F | -1.00282100 | -3.50968900 | -0.03377900 | -1648.849297 |
| | F | -1.65100900 | -1.78273000 | 1.10900700 | Sum of electronic and thermal Free Energies= |
| | F | -2.31378800 | -2.03717200 | -0.94531100 | -1648.966130 |
| | F | 0.89742800 | 0.63166400 | -1.30232200 | |

| | | | | | |
|---|---|---|---|---|---|
| Pr2 | F | -1.09380400 | 0.10059700 | -2.06579200 | |
| | O | -2.60147100 | 0.83571000 | 0.18868400 | |
| | O | -0.95260700 | 2.62325900 | -0.35684300 | |
| | C | 0.21821300 | 3.48179400 | -0.19215800 | |
| | H | -0.09109600 | 4.29999400 | 0.46519400 | |
| | H | 1.01969100 | 2.92977500 | 0.30536900 | |
| | C | -3.50421500 | 0.84368300 | 1.33512300 | |
| | H | -3.75599900 | 1.88559900 | 1.55919500 | |
| | H | -2.98654000 | 0.41454800 | 2.19673200 | |
| | C | 0.64688400 | 3.98999900 | -1.55337700 | |
| | H | -0.16951200 | 4.53013400 | -2.04432900 | |
| | H | 1.49479800 | 4.67564400 | -1.43898800 | |
| | H | 0.95424100 | 3.15952600 | -2.19582600 | |
| | C | -4.73092500 | 0.04065900 | 0.95761100 | |
| | H | -4.45566200 | -0.99062900 | 0.71728800 | |
| | H | -5.43400300 | 0.02925400 | 1.79848200 | |
| | H | -5.23574900 | 0.47907400 | 0.09051300 | |
| | C | 0.91448600 | -2.16877500 | -1.51796900 | |
| | H | 0.63469200 | -3.16959100 | -1.82944600 | |
| | H | 1.39365600 | -1.56805700 | -2.28426200 | |
| | C | 1.31187600 | -1.92964800 | -0.09134000 | |
| | H | 1.18162000 | -2.79261900 | 0.55915800 | |
| | C | 2.48136600 | -1.07273900 | 0.30635400 | |
| | C | 2.47037000 | -0.37355100 | 1.52159300 | |
| | C | 3.63582700 | -1.03481400 | -0.48518500 | |
| | C | 3.58796200 | 0.35684900 | 1.92647500 | |
| | H | 1.57566100 | -0.37845200 | 2.13545000 | |
| | C | 4.75534200 | -0.30619400 | -0.07868400 | |
| | H | 3.66223600 | -1.58393800 | -1.42266100 | |
| | C | 4.73439400 | 0.39277100 | 1.12887600 | |
| | H | 3.56103300 | 0.89987200 | 2.86768600 | |
| | H | 5.64318400 | -0.28823700 | -0.70564900 | |
| | H | 5.60477800 | 0.96081100 | 1.44710400 | |
| 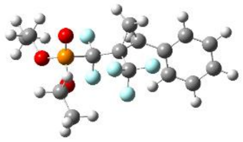 Pr3 | P | 2.40333500 | -0.01082900 | 0.32465600 | Sum of electronic and zero-point Energies= |
| | O | 2.57895500 | 0.06535200 | 1.79679300 | -1648.890124 |
| | C | 0.73554500 | -0.72023700 | -0.18264600 | Sum of electronic and thermal Energies= |
| | C | -0.54457900 | -0.22145700 | 0.47479000 | -1648.852113 |
| | C | -0.78903900 | 1.26523200 | 0.36121300 | Sum of electronic and thermal Enthalpies= |
| | F | -1.96069000 | 1.63420300 | 0.91436300 | -1648.850897 |
| | F | 0.17301700 | 1.97946800 | 1.00207400 | Sum of electronic and thermal Free Energies= |
| | F | -0.79507600 | 1.68400600 | -0.92250700 | -1648.968676 |
| | F | 0.85863900 | -2.07423900 | 0.09166100 | |
| | F | 0.60981400 | -0.61421400 | -1.54429700 | |
| | O | 2.45156900 | 1.33159400 | -0.53680800 | |
| | O | 3.43555200 | -0.94689400 | -0.47611000 | |
| | C | 3.97965300 | -2.17172100 | 0.10060100 | |
| | H | 5.03251800 | -1.96747900 | 0.31766400 | |
| | H | 3.47650000 | -2.39545600 | 1.04492900 | |
| | C | 3.07979600 | 2.54530400 | -0.02535600 | |
| | H | 4.16364300 | 2.43596200 | -0.13757000 | |
| | H | 2.84494800 | 2.64533300 | 1.03740500 | |
| | C | 3.82603500 | -3.29860300 | -0.90070700 | |
| | H | 4.32832800 | -3.05514400 | -1.84283100 | |
| | H | 4.27565900 | -4.21345100 | -0.49683000 | |
| | H | 2.76903100 | -3.49081600 | -1.10749600 | |
| | C | 2.54879900 | 3.71134800 | -0.83173100 | |
| | H | 1.46296600 | 3.78997800 | -0.72262000 | |
| | H | 3.00549700 | 4.64153500 | -0.47444800 | |
| | H | 2.78686400 | 3.59504600 | -1.89415100 | |
| | C | -1.06269700 | -0.91419900 | 1.71617400 | |
| | H | -0.43456100 | -1.68115000 | 2.15595300 | |

| | | | | | |
|---|---|---|---|---|---|
| | H | -1.57978300 | -0.28103300 | 2.43031500 | |
| | C | -1.72680100 | -1.19385700 | 0.39932000 | |
| | H | -1.42926500 | -2.13698100 | -0.05390500 | |
| | C | -3.12802800 | -0.78939400 | 0.04499100 | |
| | C | -4.16449500 | -0.86489600 | 0.98218200 | |
| | C | -3.42679900 | -0.39604200 | -1.26742900 | |
| | C | -5.47512600 | -0.54981500 | 0.61737900 | |
| | H | -3.94575600 | -1.17507900 | 2.00069100 | |
| | C | -4.73484700 | -0.08025200 | -1.63232400 | |
| | H | -2.62497600 | -0.32860700 | -1.99856300 | |
| | C | -5.76373400 | -0.15633200 | -0.69005300 | |
| | H | -6.26976700 | -0.61254800 | 1.35636100 | |
| | H | -4.95050600 | 0.22795000 | -2.65203400 | |
| | H | -6.78340600 | 0.09047300 | -0.97377000 | |
| 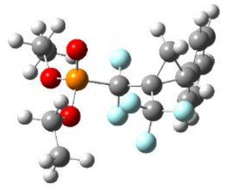 | P | 1.75184100 | 0.99571500 | -0.38663500 | Sum of electronic and zero-point Energies= |
| | O | 2.07346800 | 0.90683500 | -1.83348000 | -1648.889257 |
| | C | 0.09069200 | 0.22135500 | 0.04803900 | Sum of electronic and thermal Energies= |
| | C | -0.24303600 | -1.16749200 | -0.47684800 | -1648.851304 |
| | C | 0.74505700 | -2.25017500 | -0.10228000 | Sum of electronic and thermal Enthalpies= |
| | F | 0.30795800 | -3.47616500 | -0.47181000 | -1648.850088 |
| Pr4 | F | 1.94433500 | -2.08000800 | -0.71345400 | Sum of electronic and thermal Free Energies= |
| | F | 0.98080800 | -2.29301600 | 1.22484900 | -1648.967355 |
| | F | -0.82699000 | 1.11889100 | -0.46394300 | |
| | F | -0.05397100 | 0.24496700 | 1.41227000 | |
| | O | 2.71924900 | 0.28686900 | 0.66721800 | |
| | O | 1.62047800 | 2.47230800 | 0.23306000 | |
| | C | 1.07759900 | 3.59040600 | -0.53197000 | |
| | H | 1.91991200 | 4.25840500 | -0.73614700 | |
| | H | 0.68865800 | 3.23349700 | -1.48919500 | |
| | C | 4.10518000 | -0.02685800 | 0.33662400 | |
| | H | 4.68475700 | 0.90054600 | 0.39396200 | |
| | H | 4.14797200 | -0.40476700 | -0.68807800 | |
| | C | 0.00691500 | 4.27805900 | 0.29076400 | |
| | H | 0.41112900 | 4.62080400 | 1.24909300 | |
| | H | -0.37238300 | 5.15021400 | -0.25496300 | |
| | H | -0.82751800 | 3.59803500 | 0.48578100 | |
| | C | 4.59318000 | -1.05360700 | 1.33641900 | |
| | H | 3.98786100 | -1.96318800 | 1.27840000 | |
| | H | 5.63575200 | -1.31155900 | 1.11742500 | |
| | H | 4.54077000 | -0.66287000 | 2.35793100 | |
| | C | -0.91934700 | -1.30120600 | -1.82339700 | |
| | H | -1.11770700 | -0.37170200 | -2.34614100 | |
| | H | -0.60655100 | -2.12401800 | -2.45852800 | |
| | C | -1.71122500 | -1.60517600 | -0.58522600 | |
| | H | -1.82915900 | -2.66674900 | -0.37986700 | |
| | C | -2.87668200 | -0.78509300 | -0.11045900 | |
| | C | -3.11061500 | -0.61890500 | 1.26202000 | |
| | C | -3.79306200 | -0.24711300 | -1.02168000 | |
| | C | -4.23277700 | 0.07632700 | 1.71105700 | |
| | H | -2.40157500 | -1.02465500 | 1.97825000 | |
| | C | -4.91748700 | 0.44865600 | -0.57328600 | |
| | H | -3.62726500 | -0.37678500 | -2.08795800 | |
| | C | -5.14042900 | 0.61281600 | 0.79443600 | |
| | H | -4.39709200 | 0.20142400 | 2.77818000 | |
| | H | -5.61896800 | 0.86058000 | -1.29423400 | |
| | H | -6.01480900 | 1.15498100 | 1.14475100 | |

**Intermediates**

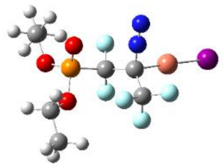

Int1

| | | | |
|---|---|---|---|
| P | -2.79870800 | 0.51772800 | 0.21971900 |
| O | -3.00354800 | 0.32181800 | 1.67895400 |
| C | -0.95504600 | 0.50597900 | -0.14276500 |
| C | -0.10151400 | -0.59187000 | 0.52782300 |
| N | -0.17174500 | -0.49570800 | 1.89775600 |
| N | -0.16859800 | -0.41091500 | 3.01458200 |
| C | -0.31404400 | -2.04889400 | 0.10499000 |
| F | 0.58486200 | -2.83834500 | 0.72287400 |
| F | -1.53781700 | -2.52175100 | 0.43464100 |
| F | -0.16737300 | -2.16701500 | -1.21619300 |
| F | -0.49590900 | 1.71014300 | 0.34896500 |
| F | -0.70827700 | 0.47027800 | -1.47271700 |
| O | -3.30486900 | -0.59396700 | -0.79774000 |
| O | -3.32834700 | 1.87605300 | -0.43187400 |
| C | -3.33194800 | 3.15464100 | 0.28681300 |
| H | -4.38523200 | 3.41255800 | 0.42526200 |
| H | -2.87844800 | 3.02242400 | 1.27244100 |
| C | -4.55038100 | -1.33078500 | -0.56136100 |
| H | -5.38233300 | -0.65807100 | -0.79127800 |
| H | -4.59559800 | -1.60552800 | 0.49611100 |
| C | -2.60313400 | 4.19070000 | -0.54345800 |
| H | -3.06413100 | 4.29509200 | -1.53104800 |
| H | -2.65098800 | 5.16151600 | -0.03647500 |
| H | -1.55132600 | 3.91915500 | -0.67362400 |
| C | -4.54362000 | -2.54670800 | -1.46148700 |
| H | -3.69403900 | -3.19600500 | -1.22860100 |
| H | -5.46866100 | -3.11430700 | -1.30901900 |
| H | -4.48635300 | -2.25522300 | -2.51512900 |
| Cu | 1.87696500 | -0.09720400 | 0.12637200 |
| I | 4.23586800 | 0.37185600 | -0.33343000 |

Sum of electronic and zero-point Energies=
-3387.204920
Sum of electronic and thermal Energies=
-3387.169262
Sum of electronic and thermal Enthalpies=
-3387.168046
Sum of electronic and thermal Free Energies=
-3387.285836

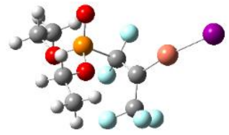

Int2

| | | | |
|---|---|---|---|
| P | -2.17621800 | -0.51848000 | -0.83276000 |
| O | -1.76876700 | -1.53224000 | -1.83378400 |
| C | -1.19941000 | -0.69644200 | 0.82864800 |
| C | -0.12810800 | 0.30653000 | 0.78832100 |
| C | -0.40243900 | 1.62854200 | 1.47251800 |
| F | 0.06722200 | 1.47981500 | 2.73392700 |
| F | 0.27340300 | 2.63354100 | 0.88798600 |
| F | -1.68872800 | 2.00736000 | 1.55204500 |
| F | -0.71078900 | -1.97225900 | 0.84531200 |
| F | -2.07374700 | -0.59464100 | 1.87520900 |
| O | -1.83259800 | 1.02168300 | -1.08679700 |
| O | -3.71412800 | -0.49188700 | -0.40299100 |
| C | -4.55507500 | -1.69328900 | -0.46165400 |
| H | -5.56525000 | -1.29765000 | -0.58716400 |
| H | -4.28261600 | -2.26366600 | -1.35325300 |
| C | -2.43918700 | 1.82559500 | -2.15337900 |
| H | -3.52084300 | 1.82908400 | -1.99304300 |
| H | -2.21391500 | 1.34667300 | -3.11041300 |
| C | -4.42984900 | -2.51944400 | 0.80349200 |
| H | -4.66815600 | -1.92198100 | 1.68864500 |
| H | -5.13343300 | -3.35857800 | 0.75160800 |
| H | -3.42137200 | -2.93114000 | 0.91730100 |
| C | -1.85158400 | 3.21732400 | -2.06218000 |
| H | -0.76920800 | 3.19852600 | -2.22448600 |
| H | -2.30533200 | 3.85040800 | -2.83288700 |
| H | -2.05431300 | 3.66220400 | -1.08294000 |
| Cu | 1.55729700 | -0.06936100 | 0.10023200 |
| I | 3.90882500 | -0.54156400 | -0.38386800 |

Sum of electronic and zero-point Energies=
-3277.653482
Sum of electronic and thermal Energies=
-3277.620988
Sum of electronic and thermal Enthalpies=
-3277.619772
Sum of electronic and thermal Free Energies=
-3277.730082

| | | | | | |
|---|---|---|---|---|---|
| 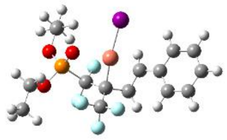 | P | -2.59523300 | -0.35283100 | 0.01961800 | Sum of electronic and zero-point Energies= |
| | O | -1.56411400 | -1.00090500 | -0.86708200 | -3587.327381 |
| | C | -1.67028300 | 0.76612200 | 1.20923400 | Sum of electronic and thermal Energies= |
| | C | -0.37790300 | 1.31589100 | 0.63816100 | -3587.283415 |
| | C | -0.55397900 | 2.36811300 | -0.42333100 | Sum of electronic and thermal Enthalpies= |
| Int3_1 | F | 0.62873800 | 2.70471200 | -0.99444700 | -3587.282199 |
| | F | -1.37373900 | 1.98238100 | -1.43320000 | Sum of electronic and thermal Free Energies= |
| | F | -1.07682800 | 3.51902400 | 0.07148100 | -3587.416179 |
| | F | -1.34340800 | -0.05726100 | 2.27727200 | |
| | F | -2.48737000 | 1.74100200 | 1.71792800 | |
| | O | -3.68604400 | 0.58507000 | -0.64684200 | |
| | O | -3.47374800 | -1.34932800 | 0.90301500 | |
| | C | -2.95112500 | -2.60782400 | 1.44759600 | |
| | H | -3.40851400 | -3.40355100 | 0.85387600 | |
| | H | -1.86919600 | -2.65223800 | 1.30303500 | |
| | C | -4.09192200 | 0.44323500 | -2.04920600 | |
| | H | -4.82589600 | -0.36684700 | -2.09674800 | |
| | H | -3.21570100 | 0.16605100 | -2.64040000 | |
| | C | -3.33530400 | -2.70027100 | 2.90904400 | |
| | H | -4.42165900 | -2.64119900 | 3.03311000 | |
| | H | -2.99363500 | -3.66001300 | 3.31385900 | |
| | H | -2.87132600 | -1.89363800 | 3.48454100 | |
| | C | -4.67817000 | 1.76546500 | -2.49316300 | |
| | H | -3.93029400 | 2.56141300 | -2.42501400 | |
| | H | -5.00664200 | 1.68324500 | -3.53559100 | |
| | H | -5.54233200 | 2.03903000 | -1.87901200 | |
| | Cu | 0.46306300 | -0.32384600 | -0.19080300 | |
| | I | 1.73461000 | -2.36450900 | -1.06466300 | |
| | C | 0.66401500 | 1.71921300 | 1.69096000 | |
| | H | 1.03704200 | 2.73603500 | 1.55759900 | |
| | H | 0.29793200 | 1.62903700 | 2.71646300 | |
| | C | 1.66126100 | 0.64840500 | 1.34274600 | |
| | H | 1.54108500 | -0.27479100 | 1.90895000 | |
| | C | 3.01398300 | 0.90651500 | 0.86442400 | |
| | C | 3.98487800 | -0.11026400 | 1.01951600 | |
| | C | 3.42489100 | 2.15296800 | 0.34230700 | |
| | C | 5.31555200 | 0.11975800 | 0.69440100 | |
| | H | 3.67995300 | -1.07859100 | 1.40349800 | |
| | C | 4.75644500 | 2.37244000 | 0.00474700 | |
| | H | 2.70147700 | 2.94308500 | 0.18220900 | |
| | C | 5.70588900 | 1.36119100 | 0.18155800 | |
| | H | 6.04796800 | -0.67044800 | 0.83089400 | |
| | H | 5.05588400 | 3.33529900 | -0.39920900 | |
| | H | 6.74452100 | 1.53792800 | -0.08391800 | |
| 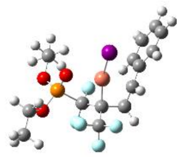 | P | 2.15561500 | 0.62425000 | -0.83511600 | Sum of electronic and zero-point Energies= |
| | O | 1.11538900 | -0.28692100 | -1.43336400 | -3587.328871 |
| | C | 1.49947100 | 1.19573900 | 0.82714200 | Sum of electronic and thermal Energies= |
| | C | 0.61370500 | 0.17444800 | 1.51523200 | -3587.284853 |
| | C | 1.34850900 | -0.99131100 | 2.13021200 | Sum of electronic and thermal Enthalpies= |
| Int3_2 | F | 0.48062800 | -1.90233400 | 2.63265600 | -3587.283637 |
| | F | 2.14450900 | -1.64895300 | 1.24967000 | Sum of electronic and thermal Free Energies= |
| | F | 2.14594200 | -0.61573600 | 3.16029800 | -3587.418112 |
| | F | 0.73601400 | 2.31269700 | 0.52091000 | |
| | F | 2.51407500 | 1.64174900 | 1.63209400 | |
| | O | 3.58858000 | 0.03066800 | -0.51489800 | |
| | O | 2.48776200 | 1.93535300 | -1.68093400 | |
| | C | 1.48595200 | 2.63761900 | -2.49055100 | |
| | H | 1.70711700 | 2.38444600 | -3.53075900 | |
| | H | 0.48766500 | 2.26391900 | -2.25109900 | |
| | C | 4.13276200 | -1.14227900 | -1.21027700 | |
| | H | 4.50253200 | -0.80426600 | -2.18292400 | |

| | | | | | |
|---|---|---|---|---|---|
| | H | 3.32511200 | -1.86171600 | -1.36466200 | |
| | C | 1.61144400 | 4.12369200 | -2.23074900 | |
| | H | 2.62079900 | 4.47854100 | -2.46326900 | |
| | H | 0.90080900 | 4.66444900 | -2.86664900 | |
| | H | 1.39016900 | 4.35485800 | -1.18427500 | |
| | C | 5.23978000 | -1.70912000 | -0.34907700 | |
| | H | 4.84904900 | -2.02488700 | 0.62304400 | |
| | H | 5.67576600 | -2.58113000 | -0.84975900 | |
| | H | 6.03176400 | -0.97023600 | -0.18898700 | |
| | Cu | -0.51824700 | -0.61383900 | 0.02526900 | |
| | I | -1.97998400 | -2.25587600 | -1.30302300 | |
| | C | -0.44793800 | 0.73932300 | 2.46618700 | |
| | H | -0.38372300 | 0.34574800 | 3.48402100 | |
| | H | -0.43350200 | 1.82736300 | 2.52052200 | |
| | C | -1.60742200 | 0.18052400 | 1.68744200 | |
| | H | -1.92715300 | -0.81279500 | 1.99492900 | |
| | C | -2.64167800 | 1.00517700 | 1.06207700 | |
| | C | -3.88534500 | 0.40398800 | 0.76048000 | |
| | C | -2.48871100 | 2.38846700 | 0.82221700 | |
| | C | -4.94050000 | 1.16015500 | 0.26580800 | |
| | H | -4.01008400 | -0.66205900 | 0.92196600 | |
| | C | -3.54500000 | 3.13758100 | 0.31261000 | |
| | H | -1.54004500 | 2.87403700 | 1.01781900 | |
| | C | -4.77330500 | 2.52972700 | 0.03575600 | |
| | H | -5.89115500 | 0.68124400 | 0.05042600 | |
| | H | -3.41088400 | 4.20029800 | 0.13123800 | |
| | H | -5.59487800 | 3.11920600 | -0.36161800 | |
| 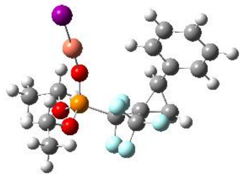 | P | 1.58348800 | -1.51381700 | -0.14650100 | Sum of electronic and zero-point Energies= |
| | O | 0.24661800 | -1.28649800 | -0.80634700 | -3587.365284 |
| | C | 2.70559800 | -0.02723200 | -0.36838000 | Sum of electronic and thermal Energies= |
| | C | 2.20707300 | 1.36299000 | -0.00491100 | -3587.321275 |
| | C | 1.72925600 | 1.51848500 | 1.42237000 | Sum of electronic and thermal Enthalpies= |
| | F | 1.57996000 | 2.80809900 | 1.77159900 | -3587.320059 |
| Int4_1 | F | 0.52664800 | 0.91358400 | 1.61206400 | Sum of electronic and thermal Free Energies= |
| | F | 2.58672400 | 0.96121000 | 2.30352600 | -3587.457358 |
| | F | 3.01395700 | -0.05388800 | -1.71465500 | |
| | F | 3.86813900 | -0.29868700 | 0.29821200 | |
| | O | 1.63291200 | -1.74347500 | 1.41153500 | |
| | O | 2.38588200 | -2.74298100 | -0.75842500 | |
| | C | 2.38412100 | -3.08922900 | -2.18640300 | |
| | H | 1.42693700 | -2.78831400 | -2.61967600 | |
| | H | 3.18834200 | -2.51580700 | -2.65364100 | |
| | C | 0.62661600 | -2.48387100 | 2.18542500 | |
| | H | 0.74152700 | -3.54503500 | 1.94718500 | |
| | H | -0.36708600 | -2.14852100 | 1.87244500 | |
| | C | 2.60908200 | -4.58076400 | -2.30313700 | |
| | H | 1.79543800 | -5.13951500 | -1.82950200 | |
| | H | 2.64613800 | -4.85622600 | -3.36335800 | |
| | H | 3.55595600 | -4.87324300 | -1.83793000 | |
| | C | 0.88046000 | -2.19053000 | 3.64686100 | |
| | H | 0.79483300 | -1.11761600 | 3.84319500 | |
| | H | 0.13530100 | -2.71763700 | 4.25301600 | |
| | H | 1.87706100 | -2.52788400 | 3.94901600 | |
| | Cu | -1.64467600 | -1.09508600 | -0.38632500 | |
| | I | -4.03736300 | -0.94629900 | 0.03253600 | |
| | C | 2.96835300 | 2.51633500 | -0.63131400 | |
| | H | 3.12816000 | 3.38193300 | 0.00265300 | |
| | H | 3.80336000 | 2.26207400 | -1.27589200 | |
| | C | 1.57842800 | 2.22306300 | -1.10962400 | |
| | H | 1.53201400 | 1.68833400 | -2.05511700 | |
| | C | 0.41032400 | 3.14179400 | -0.88399100 | |

| | | | | | |
|---|---|---|---|---|---|
| | C | -0.89165700 | 2.62085100 | -0.85717900 | |
| | C | 0.58457900 | 4.52576200 | -0.76216400 | |
| | C | -1.99294500 | 3.46121600 | -0.69887200 | |
| | H | -1.04765600 | 1.54904500 | -0.95811500 | |
| | C | -0.51696100 | 5.37001200 | -0.60658500 | |
| | H | 1.58509300 | 4.94814500 | -0.79565200 | |
| | C | -1.80786100 | 4.84033900 | -0.57170300 | |
| | H | -2.99221300 | 3.03534400 | -0.67299900 | |
| | H | -0.36367900 | 6.44202800 | -0.51391200 | |
| | H | -2.66492200 | 5.49695000 | -0.44834500 | |
| 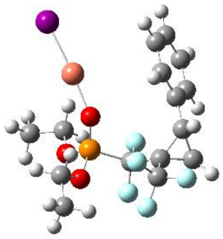 | P | -1.05278400 | -1.26852000 | 0.50043800 | Sum of electronic and zero-point Energies= |
| | O | -0.02124900 | -0.79752900 | -0.49345600 | -3587.363156 |
| | C | -2.19305000 | 0.12677100 | 1.03372300 | Sum of electronic and thermal Energies= |
| | C | -2.85540100 | 1.02371400 | 0.00173200 | -3587.319205 |
| | C | -3.67408400 | 0.29435900 | -1.04145100 | Sum of electronic and thermal Enthalpies= |
| | F | -4.42452400 | 1.14806600 | -1.77015500 | -3587.317989 |
| | F | -2.87861600 | -0.36942500 | -1.92098600 | Sum of electronic and thermal Free Energies= |
| | F | -4.51084700 | -0.61512900 | -0.50518700 | -3587.454622 |
| Int4_2 | F | -1.41971600 | 0.89202100 | 1.87537600 | |
| | F | -3.16024900 | -0.45071800 | 1.81579100 | |
| | O | -2.05742200 | -2.36757000 | -0.02494500 | |
| | O | -0.49661800 | -1.88791000 | 1.85355600 | |
| | C | 0.57043800 | -1.30343700 | 2.67963900 | |
| | H | 1.27377100 | -0.77337100 | 2.02885500 | |
| | H | 0.09550900 | -0.58867100 | 3.35488000 | |
| | C | -1.75769800 | -3.26806800 | -1.14652600 | |
| | H | -1.01775600 | -3.99503700 | -0.79984100 | |
| | H | -1.32859300 | -2.67644500 | -1.95806400 | |
| | C | 1.24983800 | -2.43072500 | 3.42428200 | |
| | H | 1.72479200 | -3.13089000 | 2.72972900 | |
| | H | 2.02591000 | -2.01142200 | 4.07452100 | |
| | H | 0.53456300 | -2.97803700 | 4.04700700 | |
| | C | -3.05708400 | -3.92770000 | -1.55133600 | |
| | H | -3.78862300 | -3.17681400 | -1.86440800 | |
| | H | -2.86957300 | -4.60621000 | -2.39122200 | |
| | H | -3.47836500 | -4.50820600 | -0.72438400 | |
| | Cu | 1.91318700 | -0.54551500 | -0.53594400 | |
| | I | 4.34616700 | -0.38916400 | -0.56763400 | |
| | C | -3.40203700 | 2.34146300 | 0.51754500 | |
| | H | -4.38742600 | 2.64420700 | 0.17932200 | |
| | H | -3.19548400 | 2.55466700 | 1.56123600 | |
| | C | -2.26327100 | 2.36976300 | -0.45536400 | |
| | C | -0.88161300 | 2.87471600 | -0.15539700 | |
| | C | -0.59347300 | 3.65199200 | 0.97286200 | |
| | C | 0.14273200 | 2.62722100 | -1.08235900 | |
| | C | 0.69377300 | 4.15121700 | 1.18286600 | |
| | H | -1.37774000 | 3.87900400 | 1.68885400 | |
| | C | 1.42973500 | 3.12437200 | -0.87416800 | |
| | H | -0.07208700 | 2.04187700 | -1.97288800 | |
| | C | 1.70991300 | 3.88598800 | 0.26366200 | |
| | H | 0.89929200 | 4.75262600 | 2.06450300 | |
| | H | 2.21199200 | 2.91978000 | -1.59944100 | |
| | H | 2.71192900 | 4.27257900 | 0.42700000 | |
| | H | -2.56405400 | 2.58468700 | -1.47828600 | |
| 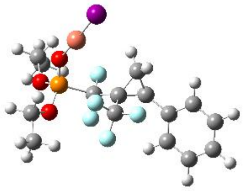 | P | 0.54038800 | 2.24366800 | 0.07423800 | Sum of electronic and zero-point Energies= |
| | O | 1.73353800 | 1.39361900 | -0.27278600 | -3587.363978 |
| | C | -1.07595500 | 1.53132300 | -0.56978300 | Sum of electronic and thermal Energies= |
| | C | -1.45088900 | 0.08011500 | -0.31903800 | -3587.320033 |
| | C | -1.40207700 | -0.35438300 | 1.12703400 | Sum of electronic and thermal Enthalpies= |
| | F | -1.80435100 | -1.62245900 | 1.29715600 | -3587.318817 |
| | F | -0.12825900 | -0.28653600 | 1.61408700 | Sum of electronic and thermal Free Energies= |

| | | | | | |
|---|---|---|---|---|---|
| Int4_3 | F | -2.15867100 | 0.43216000 | 1.91990900 | -3587.455471 |
| | F | -0.99265600 | 1.73674200 | -1.93382500 | |
| | F | -2.07560600 | 2.34696300 | -0.11109100 | |
| | O | 0.22700700 | 2.50461500 | 1.59805500 | |
| | O | 0.61376300 | 3.69765200 | -0.56136600 | |
| | C | 1.17751300 | 3.98081400 | -1.88932100 | |
| | H | 2.12188400 | 4.49883500 | -1.70515900 | |
| | H | 1.39192700 | 3.04234100 | -2.40527900 | |
| | C | 1.22638400 | 2.41726500 | 2.67236200 | |
| | H | 1.84794900 | 3.31536100 | 2.61644400 | |
| | H | 1.84899200 | 1.53671100 | 2.49713400 | |
| | C | 0.19720100 | 4.84037500 | -2.65781800 | |
| | H | -0.01825100 | 5.76776400 | -2.11740800 | |
| | H | 0.63021600 | 5.10029700 | -3.63071900 | |
| | H | -0.74131500 | 4.30369500 | -2.82628300 | |
| | C | 0.47281700 | 2.32392200 | 3.98011700 | |
| | H | -0.16495600 | 1.43512400 | 3.99334000 | |
| | H | 1.19118300 | 2.25188800 | 4.80448100 | |
| | H | -0.15064100 | 3.20950300 | 4.13949200 | |
| | Cu | 2.51029500 | -0.38785700 | -0.21545400 | |
| | I | 3.55475000 | -2.58923700 | -0.21439500 | |
| | C | -1.15769700 | -0.97048100 | -1.37182100 | |
| | H | -0.57281800 | -0.66701400 | -2.23344300 | |
| | H | -0.93310500 | -1.96372700 | -0.99600000 | |
| | C | -2.55794100 | -0.45126900 | -1.23856400 | |
| | H | -2.82876600 | 0.28641500 | -1.99133100 | |
| | C | -3.73020700 | -1.25237800 | -0.75211300 | |
| | C | -3.87053000 | -2.60723400 | -1.07297700 | |
| | C | -4.74195500 | -0.61755400 | -0.01683500 | |
| | C | -5.00133000 | -3.31767900 | -0.66376700 | |
| | H | -3.09507900 | -3.10731400 | -1.64703900 | |
| | C | -5.87066000 | -1.32671000 | 0.39244300 | |
| | H | -4.63481200 | 0.43342600 | 0.24056400 | |
| | C | -6.00332600 | -2.67996800 | 0.06949500 | |
| | H | -5.09733200 | -4.36968700 | -0.91892300 | |
| | H | -6.64535400 | -0.82379000 | 0.96521300 | |
| | H | -6.88248300 | -3.23330400 | 0.38862400 | |
| 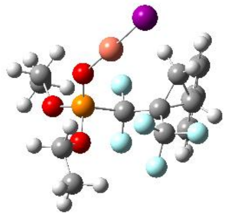 | P | -0.27445900 | 1.89525100 | -0.48617300 | Sum of electronic and zero-point Energies= |
| | O | 0.95434800 | 1.30388800 | -1.12997500 | -3587.363608 |
| | C | -1.51022800 | 0.59362100 | 0.08313800 | Sum of electronic and thermal Energies= |
| | C | -1.05484800 | -0.56027500 | 0.95960600 | -3587.319578 |
| | C | -0.32689800 | -0.15615500 | 2.22301800 | Sum of electronic and thermal Enthalpies= |
| | F | -0.09429500 | -1.21068300 | 3.02816400 | -3587.318362 |
| | F | 0.88990500 | 0.39130800 | 1.94839800 | Sum of electronic and thermal Free Energies= |
| Int 4_4 | F | -1.01296200 | 0.75842500 | 2.93663500 | -3587.455821 |
| | F | -2.00230200 | 0.09643300 | -1.10346900 | |
| | F | -2.53208700 | 1.27213600 | 0.69051000 | |
| | O | -0.08123600 | 2.78026100 | 0.80593000 | |
| | O | -1.09973100 | 2.82135700 | -1.47809200 | |
| | C | -1.26794500 | 2.54421700 | -2.91225900 | |
| | H | -0.70310100 | 3.32420100 | -3.42870900 | |
| | H | -0.82229900 | 1.57647700 | -3.15276700 | |
| | C | 1.13628400 | 3.54981900 | 1.09720600 | |
| | H | 1.15577100 | 4.40780200 | 0.41891800 | |
| | H | 2.00308700 | 2.91420100 | 0.89968000 | |
| | C | -2.74297300 | 2.59171100 | -3.24828900 | |
| | H | -3.17324600 | 3.56370300 | -2.98637500 | |
| | H | -2.87263600 | 2.43595000 | -4.32565800 | |
| | H | -3.28902600 | 1.80813600 | -2.71484400 | |
| | C | 1.06468000 | 3.96980700 | 2.54816100 | |
| | H | 1.01337100 | 3.09241900 | 3.19983600 | |

| | | | | | |
|---|---|---|---|---|---|
| | H | 1.96398800 | 4.54208400 | 2.80212200 | |
| | H | 0.18852000 | 4.59951400 | 2.73282800 | |
| | Cu | 2.36759400 | -0.00086400 | -0.80471800 | |
| | I | 4.14801400 | -1.62323900 | -0.44177400 | |
| | C | -0.63727400 | -1.86594200 | 0.31226800 | |
| | H | -0.67313900 | -1.89108200 | -0.77176700 | |
| | H | 0.20358100 | -2.39285600 | 0.75198200 | |
| | C | -1.93613400 | -1.81639700 | 1.05909100 | |
| | H | -1.89753900 | -2.25085900 | 2.05543700 | |
| | C | -3.28153400 | -1.95688100 | 0.40561500 | |
| | C | -4.39093700 | -1.26894400 | 0.91860600 | |
| | C | -3.46762500 | -2.83652300 | -0.66765600 | |
| | C | -5.65685500 | -1.45105900 | 0.36237700 | |
| | H | -4.25828900 | -0.58165800 | 1.74963500 | |
| | C | -4.73511500 | -3.02027600 | -1.22452200 | |
| | H | -2.61812600 | -3.38583600 | -1.06445600 | |
| | C | -5.83286000 | -2.32713700 | -0.71201800 | |
| | H | -6.50627700 | -0.90770700 | 0.76788500 | |
| | H | -4.86291300 | -3.70763700 | -2.05663200 | |
| | H | -6.81958200 | -2.46904600 | -1.14476000 | |
| **Transition states** | | | | | |
| 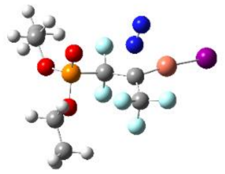 | P | -2.78393500 | 0.50023700 | 0.19884700 | Sum of electronic and zero-point Energies= |
| | O | -3.01075200 | 0.31963600 | 1.65608800 | -3387.187169 |
| | C | -0.94828100 | 0.53391600 | -0.21143600 | Sum of electronic and thermal Energies= |
| | C | 0.04005100 | -0.52869800 | 0.26848000 | -3387.151373 |
| | N | -0.29991000 | -0.38432300 | 2.19748800 | Sum of electronic and thermal Enthalpies= |
| | N | -0.08132300 | -0.23900200 | 3.27149000 | -3387.150157 |
| TS1 | C | -0.37317500 | -1.99152600 | 0.12587200 | Sum of electronic and thermal Free Energies= |
| | F | 0.55009300 | -2.79822000 | 0.67653400 | -3387.268033 |
| | F | -1.55672600 | -2.32001700 | 0.68400900 | |
| | F | -0.43995900 | -2.24675900 | -1.19197800 | |
| | F | -0.49851100 | 1.74207500 | 0.27180000 | |
| | F | -0.77922100 | 0.53141500 | -1.56791000 | |
| | O | -3.26877000 | -0.62144900 | -0.81955100 | |
| | O | -3.33095200 | 1.84574500 | -0.46870400 | |
| | C | -3.38178500 | 3.12118300 | 0.25149000 | |
| | H | -4.44379800 | 3.34632400 | 0.38138600 | |
| | H | -2.93317300 | 3.00245900 | 1.24113300 | |
| | C | -4.41986900 | -1.48118500 | -0.53974600 | |
| | H | -5.32607200 | -0.88526500 | -0.68640500 | |
| | H | -4.37034300 | -1.80236300 | 0.50399700 | |
| | C | -2.67867700 | 4.18198700 | -0.56981700 | |
| | H | -3.13283100 | 4.27332100 | -1.56189700 | |
| | H | -2.76312500 | 5.14988600 | -0.06191400 | |
| | H | -1.61764500 | 3.94389900 | -0.68975100 | |
| | C | -4.35599700 | -2.65205300 | -1.49656600 | |
| | H | -3.43215000 | -3.22043700 | -1.35057400 | |
| | H | -5.20751700 | -3.31743800 | -1.31442000 | |
| | H | -4.39840200 | -2.31201200 | -2.53631600 | |
| | Cu | 1.88646100 | -0.11342700 | 0.10634200 | |
| | I | 4.25385700 | 0.34605800 | -0.34036100 | |
| 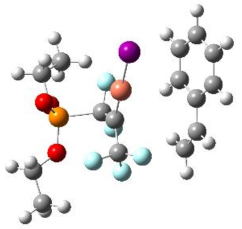 | P | -2.78702200 | -0.70809500 | 0.70132700 | Sum of electronic and zero-point Energies= |
| | O | -2.33557500 | -2.11905400 | 0.65815000 | -3587.266340 |
| | C | -1.33201200 | 0.54934100 | 0.50899400 | Sum of electronic and thermal Energies= |
| | C | -0.49334900 | 0.16556500 | -0.63432700 | -3587.221887 |
| | C | -0.98763700 | 0.51084500 | -2.02564400 | Sum of electronic and thermal Enthalpies= |
| | F | 0.05374100 | 0.73747200 | -2.84243900 | -3587.220671 |
| | F | -1.64997000 | -0.58437100 | -2.48376500 | Sum of electronic and thermal Free Energies= |
| | F | -1.81981400 | 1.55783800 | -2.12695400 | -3587.360569 |
| TS2_2 | F | -0.63437900 | 0.44374900 | 1.69109900 | |
| | F | -1.85930100 | 1.80729200 | 0.47774900 | |

| | | | | | |
|---|---|---|---|---|---|
| | O | -3.76019500 | -0.16316100 | -0.43053400 | |
| | O | -3.46488200 | -0.17786000 | 2.04574600 | |
| | C | -3.08912400 | -0.69012700 | 3.36862500 | |
| | H | -3.96128600 | -1.24563200 | 3.72250900 | |
| | H | -2.25450300 | -1.38719600 | 3.26405700 | |
| | C | -4.59944700 | -1.06896200 | -1.22516700 | |
| | H | -5.41771400 | -1.41021900 | -0.58496800 | |
| | H | -3.99408200 | -1.92863400 | -1.52114100 | |
| | C | -2.75515800 | 0.47823500 | 4.27419900 | |
| | H | -3.59988500 | 1.17028400 | 4.34511200 | |
| | H | -2.52795800 | 0.10493200 | 5.27892300 | |
| | H | -1.88372800 | 1.02064900 | 3.89792100 | |
| | C | -5.10327500 | -0.28555700 | -2.41916800 | |
| | H | -4.26565500 | 0.06535600 | -3.02797100 | |
| | H | -5.73783000 | -0.93236000 | -3.03473300 | |
| | H | -5.69427000 | 0.57809800 | -2.09967100 | |
| | Cu | 1.00420500 | -0.91123600 | -0.41166500 | |
| | I | 2.96634300 | -2.30788800 | -0.15003100 | |
| | C | 0.12631800 | 3.92193000 | -1.17772300 | |
| | H | -0.37967600 | 4.29789800 | -2.06150900 | |
| | H | -0.42414400 | 3.99160600 | -0.24414500 | |
| | C | 1.36105700 | 3.40601800 | -1.26248000 | |
| | H | 1.84299700 | 3.38842700 | -2.23983800 | |
| | C | 2.17854700 | 2.85043300 | -0.16882800 | |
| | C | 3.51228700 | 2.49322200 | -0.43233100 | |
| | C | 1.68462600 | 2.65188200 | 1.13568100 | |
| | C | 4.33006000 | 1.96526900 | 0.56779100 | |
| | H | 3.91002100 | 2.63370000 | -1.43467600 | |
| | C | 2.50006800 | 2.12337300 | 2.13433800 | |
| | H | 0.65369400 | 2.90006800 | 1.36847800 | |
| | C | 3.82708000 | 1.77956500 | 1.85696800 | |
| | H | 5.35743100 | 1.69657000 | 0.33862200 | |
| | H | 2.09736300 | 1.97399700 | 3.13252600 | |
| | H | 4.45892300 | 1.36441500 | 2.63687200 | |
| 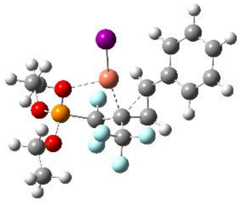 TS3_1 | P | -2.58010700 | -0.40116000 | 0.13704000 | Sum of electronic and zero-point Energies= |
| | O | -1.54817700 | -1.42778600 | -0.22051300 | -3587.319246 |
| | C | -1.70002100 | 1.02898500 | 0.95786200 | Sum of electronic and thermal Energies= |
| | C | -0.36403500 | 1.48426300 | 0.35905600 | -3587.275684 |
| | C | -0.42520100 | 1.97809600 | -1.07651300 | Sum of electronic and thermal Enthalpies= |
| | F | 0.76010800 | 2.47082300 | -1.48406800 | -3587.274468 |
| | F | -0.76742300 | 1.00307200 | -1.95587300 | Sum of electronic and thermal Free Energies= |
| | F | -1.33808400 | 2.96244600 | -1.21700700 | -3587.409239 |
| | F | -1.45006400 | 0.58498000 | 2.24767700 | |
| | F | -2.54223400 | 2.09762800 | 1.08218700 | |
| | O | -3.39325200 | 0.31067900 | -1.02206000 | |
| | O | -3.71071200 | -0.86329900 | 1.16028400 | |
| | C | -3.49568900 | -1.89348000 | 2.18325100 | |
| | H | -4.12990600 | -2.73325100 | 1.88984400 | |
| | H | -2.45393000 | -2.22059900 | 2.15350200 | |
| | C | -3.66254200 | -0.33990700 | -2.31083200 | |
| | H | -4.52167600 | -1.00111100 | -2.16821700 | |
| | H | -2.79006300 | -0.93720700 | -2.58536900 | |
| | C | -3.88677100 | -1.33436500 | 3.53678200 | |
| | H | -4.92841200 | -0.99885800 | 3.53352000 | |
| | H | -3.77742800 | -2.11530800 | 4.29754700 | |
| | H | -3.24568600 | -0.49082300 | 3.80709500 | |
| | C | -3.94547000 | 0.75257000 | -3.32069200 | |
| | H | -3.07929200 | 1.41242000 | -3.42183200 | |
| | H | -4.15660100 | 0.29919000 | -4.29518400 | |
| | H | -4.81208800 | 1.34921600 | -3.01992400 | |
| | Cu | 0.51535000 | -0.53186000 | -0.02976100 | |

| | | | | | |
|---|---|---|---|---|---|
| | I | 1.98953400 | -2.41829400 | -0.80145800 | |
| | C | 0.33435500 | 2.44670100 | 1.30449800 | |
| | H | 0.75764300 | 3.33314700 | 0.83999800 | |
| | H | -0.21087800 | 2.68366900 | 2.21490400 | |
| | C | 1.19630800 | 1.24492600 | 1.33181700 | |
| | H | 0.94689700 | 0.54888000 | 2.12893300 | |
| | C | 2.63019200 | 1.24890000 | 0.94916900 | |
| | C | 3.44870800 | 0.24717500 | 1.50458300 | |
| | C | 3.23474100 | 2.27292100 | 0.19928000 | |
| | C | 4.82804700 | 0.27107100 | 1.32292100 | |
| | H | 2.99694700 | -0.55313700 | 2.08239800 | |
| | C | 4.61441100 | 2.28430300 | 0.00541400 | |
| | H | 2.63743000 | 3.05994300 | -0.24333900 | |
| | C | 5.41602600 | 1.28712700 | 0.56629100 | |
| | H | 5.44056600 | -0.50964200 | 1.76364000 | |
| | H | 5.06421300 | 3.07928500 | -0.58231200 | |
| | H | 6.49144300 | 1.30208600 | 0.41428500 | |
| 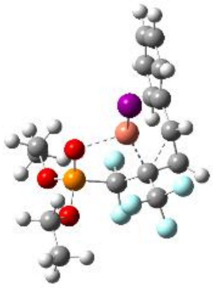 | P | 2.05751300 | 0.49582600 | -0.84361700 | Sum of electronic and zero-point Energies= |
| | O | 0.88066100 | -0.10608800 | -1.55780800 | -3587.323182 |
| | C | 1.49077500 | 1.07456800 | 0.85072700 | Sum of electronic and thermal Energies= |
| | C | 0.44421900 | 0.22714400 | 1.56854200 | -3587.280298 |
| | C | 0.94859900 | -1.10594200 | 2.09461300 | Sum of electronic and thermal Enthalpies= |
| | F | -0.05857200 | -1.82745900 | 2.63035000 | -3587.279082 |
| | F | 1.53208400 | -1.86817600 | 1.13958100 | Sum of electronic and thermal Free Energies= |
| | F | 1.87054900 | -0.93754600 | 3.06940200 | -3587.409991 |
| | F | 0.95152400 | 2.32341200 | 0.60699900 | |
| | F | 2.57345400 | 1.27806700 | 1.66380800 | |
| TS3_2 | O | 3.30616200 | -0.42583800 | -0.53246900 | |
| | O | 2.71010800 | 1.77464900 | -1.53703200 | |
| | C | 1.92877400 | 2.76632900 | -2.28548400 | |
| | H | 2.14806500 | 2.58689800 | -3.34122500 | |
| | H | 0.86219500 | 2.59684600 | -2.12098200 | |
| | C | 3.63144900 | -1.60541900 | -1.34805500 | |
| | H | 4.10009100 | -1.24680400 | -2.26935000 | |
| | H | 2.70218000 | -2.12385800 | -1.59578000 | |
| | C | 2.35698700 | 4.15024800 | -1.84577800 | |
| | H | 3.43042000 | 4.29735100 | -2.00362300 | |
| | H | 1.81611400 | 4.90133600 | -2.43329000 | |
| | H | 2.13251100 | 4.30804800 | -0.78650600 | |
| | C | 4.56671600 | -2.47471200 | -0.53763900 | |
| | H | 4.08260800 | -2.80667500 | 0.38584200 | |
| | H | 4.83585500 | -3.35889600 | -1.12660100 | |
| | H | 5.48497900 | -1.93556600 | -0.28288400 | |
| | Cu | -0.73112800 | -0.53581000 | -0.12207100 | |
| | I | -2.08108300 | -2.52773200 | -1.00546800 | |
| | C | -0.34716700 | 0.97706700 | 2.62651800 | |
| | H | -0.39871400 | 0.49133100 | 3.59943500 | |
| | H | -0.10094400 | 2.03086200 | 2.72356400 | |
| | C | -1.42344400 | 0.62157100 | 1.67054900 | |
| | H | -1.94508400 | -0.30059700 | 1.91309200 | |
| | C | -2.20922900 | 1.60021000 | 0.88075700 | |
| | C | -3.36496700 | 1.14181800 | 0.21269500 | |
| | C | -1.92773300 | 2.98026000 | 0.86186500 | |
| | C | -4.20207400 | 2.03031300 | -0.45683900 | |
| | H | -3.60386400 | 0.08271000 | 0.22513400 | |
| | C | -2.76729700 | 3.86390000 | 0.18777500 | |
| | H | -1.05894500 | 3.37204700 | 1.37618300 | |
| | C | -3.90366800 | 3.39447200 | -0.47657500 | |
| | H | -5.08692100 | 1.65406500 | -0.96175500 | |
| | H | -2.53660500 | 4.92563200 | 0.18824500 | |
| | H | -4.55495300 | 4.08875700 | -1.00020300 | |

| | | | | | |
|---|---|---|---|---|---|
| 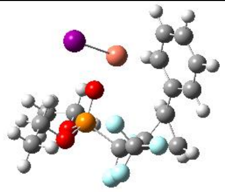 | P | 2.09734600 | -0.83797000 | -0.28771700 | Sum of electronic and zero-point Energies= |
| | O | 0.87901900 | -0.75957400 | -1.14808200 | -3587.353279 |
| | C | 2.70162800 | 0.87330000 | 0.21071000 | Sum of electronic and thermal Energies= |
| | C | 1.60425900 | 1.86657100 | 0.57466300 | -3587.310588 |
| | C | 0.82375100 | 1.52143700 | 1.82929900 | Sum of electronic and thermal Enthalpies= |
| | F | 0.07538800 | 2.55693700 | 2.26572000 | -3587.309372 |
| TS4_1 | F | -0.02872500 | 0.48449000 | 1.63030000 | Sum of electronic and thermal Free Energies= |
| | F | 1.63607300 | 1.16985500 | 2.84318800 | -3587.440543 |
| | F | 3.36995600 | 1.35156500 | -0.90312100 | |
| | F | 3.63496100 | 0.77610900 | 1.19921000 | |
| | O | 2.00065800 | -1.53899600 | 1.13210500 | |
| | O | 3.37589900 | -1.54013200 | -0.95516500 | |
| | C | 3.67444200 | -1.42901500 | -2.38019200 | |
| | H | 2.73535400 | -1.33575800 | -2.93294400 | |
| | H | 4.26392500 | -0.51858000 | -2.52169800 | |
| | C | 1.15242000 | -2.71220600 | 1.37438500 | |
| | H | 1.64311900 | -3.57538000 | 0.91384300 | |
| | H | 0.18307900 | -2.54991300 | 0.89657200 | |
| | C | 4.44504500 | -2.66432500 | -2.79710600 | |
| | H | 3.84355100 | -3.56784400 | -2.65332500 | |
| | H | 4.70796400 | -2.58864900 | -3.85865700 | |
| | H | 5.36996400 | -2.76319000 | -2.21931700 | |
| | C | 1.01723100 | -2.87015700 | 2.87278100 | |
| | H | 0.54869100 | -1.98332400 | 3.31078600 | |
| | H | 0.38430200 | -3.73826900 | 3.08826000 | |
| | H | 1.99358000 | -3.02434300 | 3.34438700 | |
| | Cu | -1.80265900 | -0.07965800 | -0.77927700 | |
| | I | -3.08165400 | -1.99124700 | 0.19083300 | |
| | C | 1.79352800 | 3.33456100 | 0.25213300 | |
| | H | 1.35653100 | 4.03793500 | 0.95243400 | |
| | H | 2.75037000 | 3.63710500 | -0.16007500 | |
| | C | 0.89486000 | 2.51100700 | -0.62476200 | |
| | H | 1.37209400 | 2.20502500 | -1.55017400 | |
| | C | -0.58835100 | 2.59578600 | -0.78892300 | |
| | C | -1.16297900 | 1.75249600 | -1.77942500 | |
| | C | -1.42566100 | 3.45833800 | -0.08204700 | |
| | C | -2.55299200 | 1.76542200 | -2.01127600 | |
| | H | -0.50525400 | 1.19441400 | -2.44307100 | |
| | C | -2.80898200 | 3.46696700 | -0.31624000 | |
| | H | -1.01068700 | 4.13720500 | 0.65455800 | |
| | C | -3.37915500 | 2.62056900 | -1.25972000 | |
| | H | -2.97204100 | 1.18227500 | -2.82680100 | |
| | H | -3.43781000 | 4.14859900 | 0.24953000 | |
| | H | -4.44990400 | 2.63289000 | -1.43785800 | |
| 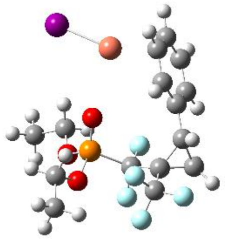 | P | -1.14512200 | -1.34031800 | 0.15554000 | Sum of electronic and zero-point Energies= |
| | O | -0.14896800 | -0.71740600 | -0.75884600 | -3587.353474 |
| | C | -2.11113800 | -0.06287200 | 1.13613300 | Sum of electronic and thermal Energies= |
| | C | -2.83868900 | 1.06476100 | 0.41435600 | -3587.310728 |
| | C | -3.92330200 | 0.62484700 | -0.54948000 | Sum of electronic and thermal Enthalpies= |
| | F | -4.71993300 | 1.66270500 | -0.89809300 | -3587.309512 |
| | F | -3.39326700 | 0.14843200 | -1.70462900 | Sum of electronic and thermal Free Energies= |
| | F | -4.71884400 | -0.33516900 | -0.04843600 | -3587.441052 |
| TS4_2 | F | -1.16932700 | 0.49179500 | 1.98373500 | |
| | F | -3.00574500 | -0.71880200 | 1.93969600 | |
| | O | -2.32791000 | -2.19483800 | -0.48578900 | |
| | O | -0.59078300 | -2.32906600 | 1.28621700 | |
| | C | 0.67712600 | -2.11057700 | 1.98314800 | |
| | H | 1.42297100 | -1.76362000 | 1.26343100 | |
| | H | 0.51383900 | -1.33419200 | 2.73635300 | |
| | C | -2.17142500 | -2.86189000 | -1.77551800 | |
| | H | -1.47664200 | -3.69773300 | -1.64526500 | |

| | | | | | |
|---|---|---|---|---|---|
| | H | -1.73934300 | -2.15256800 | -2.48639900 | |
| | C | 1.08832500 | -3.42272700 | 2.61645800 | |
| | H | 1.24636300 | -4.19143500 | 1.85308100 | |
| | H | 2.02866100 | -3.28098400 | 3.16118500 | |
| | H | 0.32827300 | -3.77675100 | 3.32122900 | |
| | C | -3.54130400 | -3.33530400 | -2.21270400 | |
| | H | -4.22665400 | -2.48831700 | -2.31455400 | |
| | H | -3.46049500 | -3.83962600 | -3.18246100 | |
| | H | -3.96162400 | -4.04168900 | -1.48936900 | |
| | Cu | 2.38757600 | 0.93098600 | -0.51368200 | |
| | I | 4.17155600 | -0.78744100 | -0.38468900 | |
| | C | -3.07253600 | 2.33337300 | 1.21031500 | |
| | H | -4.04233500 | 2.81198400 | 1.12886100 | |
| | H | -2.64144500 | 2.34495400 | 2.20542900 | |
| | C | -2.15483900 | 2.38306300 | 0.02467500 | |
| | C | -0.67836800 | 2.63927800 | 0.05536800 | |
| | C | 0.00152100 | 3.08299000 | 1.20263800 | |
| | C | 0.03933200 | 2.49066000 | -1.13559000 | |
| | C | 1.36868800 | 3.34137100 | 1.17156200 | |
| | H | -0.54526000 | 3.23048300 | 2.12845300 | |
| | C | 1.41669800 | 2.77315000 | -1.19270900 | |
| | H | -0.47395700 | 2.16439900 | -2.03503000 | |
| | C | 2.09206300 | 3.19258000 | -0.02423400 | |
| | H | 1.87633500 | 3.68344400 | 2.06849100 | |
| | H | 1.92643800 | 2.77860000 | -2.15419500 | |
| | H | 3.13742600 | 3.48415900 | -0.06999800 | |
| | H | -2.61547300 | 2.80302700 | -0.86664500 | |
| 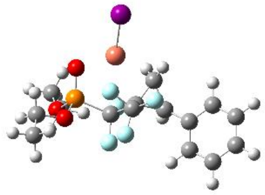 | P | -1.99075800 | 2.00105900 | -0.17907400 | Sum of electronic and zero-point Energies= |
| | O | -0.92973900 | 2.38014700 | -1.14826700 | -3587.339242 |
| | C | -2.42932100 | 0.17354500 | -0.27478600 | Sum of electronic and thermal Energies= |
| | C | -1.31728600 | -0.86583400 | -0.38039200 | -3587.296077 |
| | C | -0.31364900 | -0.85772900 | 0.73649700 | Sum of electronic and thermal Enthalpies= |
| | F | 0.62651900 | -1.80118000 | 0.62150300 | -3587.294861 |
| | F | 0.41581200 | 0.36236200 | 0.72954300 | Sum of electronic and thermal Free Energies= |
| TS4_3 | F | -0.85246100 | -0.92651900 | 1.95105400 | -3587.431380 |
| | F | -3.19549700 | 0.08195600 | -1.42170800 | |
| | F | -3.24724100 | -0.15269200 | 0.77141700 | |
| | O | -1.71442400 | 2.13492400 | 1.38707100 | |
| | O | -3.40385800 | 2.74068400 | -0.35007500 | |
| | C | -3.85705200 | 3.28339000 | -1.62840600 | |
| | H | -3.90598800 | 4.36731300 | -1.49292300 | |
| | H | -3.11147000 | 3.06771200 | -2.39774000 | |
| | C | -1.35351800 | 3.42196700 | 1.97636100 | |
| | H | -2.17783500 | 4.11969500 | 1.80003300 | |
| | H | -0.45676800 | 3.79407500 | 1.47186800 | |
| | C | -5.21635600 | 2.70049600 | -1.96641800 | |
| | H | -5.94220400 | 2.92247100 | -1.17783200 | |
| | H | -5.57960100 | 3.13781400 | -2.90327900 | |
| | H | -5.15210400 | 1.61581700 | -2.08779300 | |
| | C | -1.11519500 | 3.20039500 | 3.45626200 | |
| | H | -0.30106200 | 2.48680600 | 3.61672600 | |
| | H | -0.84147700 | 4.15013200 | 3.92828500 | |
| | H | -2.01782000 | 2.81782100 | 3.94248000 | |
| | Cu | 2.39943700 | 0.56956300 | 0.14397500 | |
| | I | 4.71580500 | 0.82745900 | -0.47042100 | |
| | C | -0.83710900 | -1.31805800 | -1.74550300 | |
| | H | -1.23075900 | -0.78471300 | -2.60317400 | |
| | H | 0.21110200 | -1.58965000 | -1.81995700 | |
| | C | -1.75183300 | -2.21763800 | -0.97223000 | |
| | H | -2.78973900 | -2.17905000 | -1.29524000 | |
| | C | -1.35810700 | -3.54593300 | -0.39676700 | |

| | | | | | |
|---|---|---|---|---|---|
| | C | -0.46739300 | -4.39358200 | -1.06479900 | |
| | C | -1.94309800 | -3.97845800 | 0.80239400 | |
| | C | -0.16033000 | -5.65126400 | -0.54058900 | |
| | H | -0.01633200 | -4.07210300 | -1.99963600 | |
| | C | -1.63588600 | -5.23359900 | 1.32670900 | |
| | H | -2.63546900 | -3.32319100 | 1.32552700 | |
| | C | -0.74245000 | -6.07349900 | 0.65589500 | |
| | H | 0.53357500 | -6.29948900 | -1.06853400 | |
| | H | -2.09244500 | -5.55538900 | 2.25864800 | |
| | H | -0.50210300 | -7.05118900 | 1.06418400 | |
| 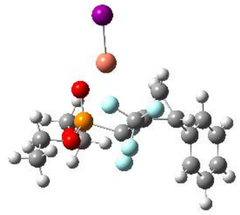 | P | -0.92831400 | 2.01486700 | -0.43558800 | Sum of electronic and zero-point Energies= |
| | O | 0.13263800 | 1.70773700 | -1.43111500 | -3587.338445 |
| | C | -1.86570300 | 0.47491600 | 0.11080100 | Sum of electronic and thermal Energies= |
| | C | -1.07200500 | -0.79005700 | 0.41873500 | -3587.295206 |
| | C | -0.02088900 | -0.64538000 | 1.48381300 | Sum of electronic and thermal Enthalpies= |
| | F | 0.58695500 | -1.80205100 | 1.78225200 | -3587.293989 |
| | F | 1.02975700 | 0.20199100 | 1.04862300 | Sum of electronic and thermal Free Energies= |
| TS4_4 | F | -0.46223300 | -0.08871100 | 2.60855200 | -3587.431319 |
| | F | -2.69942600 | 0.21305000 | -0.95390900 | |
| | F | -2.66027700 | 0.79068400 | 1.17832900 | |
| | O | -0.51800600 | 2.61507800 | 0.98555000 | |
| | O | -2.08712300 | 3.01377800 | -0.91547800 | |
| | C | -2.49742800 | 3.12888300 | -2.31297100 | |
| | H | -2.20177200 | 4.13220700 | -2.63169000 | |
| | H | -1.94580400 | 2.40243800 | -2.91482800 | |
| | C | 0.25327100 | 3.85205700 | 1.07929000 | |
| | H | -0.35433800 | 4.66239700 | 0.66514400 | |
| | H | 1.15997400 | 3.74238700 | 0.47666900 | |
| | C | -3.99841600 | 2.93020500 | -2.40803000 | |
| | H | -4.52759500 | 3.66284200 | -1.79056900 | |
| | H | -4.31983700 | 3.05835100 | -3.44784700 | |
| | H | -4.27620100 | 1.92619800 | -2.07614200 | |
| | C | 0.57506300 | 4.08442500 | 2.54166100 | |
| | H | 1.16694100 | 3.25719100 | 2.94561500 | |
| | H | 1.15379800 | 5.00847400 | 2.64621900 | |
| | H | -0.34190400 | 4.17994900 | 3.13119200 | |
| | Cu | 2.85227500 | -0.32264800 | 0.18605600 | |
| | I | 5.00133900 | -0.96315900 | -0.69752100 | |
| | C | -0.80319000 | -1.78433000 | -0.69384500 | |
| | H | -1.19133300 | -1.50433200 | -1.66672400 | |
| | H | 0.16488600 | -2.27429600 | -0.71533400 | |
| | C | -1.77845800 | -2.15948500 | 0.37931300 | |
| | H | -1.37839300 | -2.84225100 | 1.12615400 | |
| | C | -3.25177200 | -2.33756700 | 0.15051600 | |
| | C | -4.16980300 | -1.98703400 | 1.15035200 | |
| | C | -3.72170600 | -2.93935700 | -1.02268500 | |
| | C | -5.53249200 | -2.22609800 | 0.97451000 | |
| | H | -3.81521300 | -1.51357500 | 2.06150100 | |
| | C | -5.08591300 | -3.18019400 | -1.19896000 | |
| | H | -3.01721100 | -3.22574300 | -1.79888400 | |
| | C | -5.99474900 | -2.82359200 | -0.20126300 | |
| | H | -6.23371300 | -1.94451100 | 1.75528800 | |
| | H | -5.43605100 | -3.64812800 | -2.11499400 | |
| | H | -7.05640000 | -3.01015200 | -0.33762800 | |

## 10. References


1. Bergstrom, D. E.; Shum, P. W. *J. Org. Chem.* **1988**, *53*, 3953–3958. doi:10.1021/jo00252a014
2. Yang, S.; Zhu, S.; Lu, D.; Gong, Y. *Org. Lett.* **2019**, *21*, 2019–2024. doi:10.1021/acs.orglett.9b00128
3. Titaniuk, I. G.; Beletskaya, I. P.; Peregudov, A. S.; Osipov, S. N. J. Fluorine Chem. 2007, 128, 723-728.
   doi.org/10.1016/j.jfluchem.2007.02.003
